# Development of a new macrophage-specific TRAP mouse (Mac^TRAP^) and definition of the renal macrophage translational signature

**DOI:** 10.1038/s41598-020-63514-6

**Published:** 2020-05-05

**Authors:** Andreas Hofmeister, Maximilian C. Thomaßen, Sabrina Markert, André Marquardt, Mathieu Preußner, Martin Rußwurm, Ralph T. Schermuly, Ulrich Steinhoff, Hermann-Josef Gröne, Joachim Hoyer, Benjamin D. Humphreys, Ivica Grgic

**Affiliations:** 1 Department of Internal Medicine and Nephrology, University Hospital Giessen and Marburg, Philipps-University, Marburg, Germany; 2 Institute of Pathology, Julius-Maximilians-University Würzburg and Comprehensive Cancer Center Mainfranken, Würzburg, Germany; 3 Department of Internal Medicine, Universities of Giessen and Marburg Lung Center, Giessen, Germany; 4 Institute for Medical Microbiology and Hygiene, Philipps-University, Marburg, Germany; 5 Institute of Pharmacology, Philipps-University, Marburg, Germany; 6 Department of Cellular and Molecular Pathology, German Cancer Research Center, Heidelberg, Germany; 7 Division of Nephrology, Department of Medicine, Washington University in St. Louis School of Medicine, St. Louis, Missouri USA; 8 Department of Developmental Biology, Washington University in St. Louis School of Medicine, St. Louis, Missouri USA

**Keywords:** Gene ontology, Translational research, Kidney

## Abstract

Tissue macrophages play an important role in organ homeostasis, immunity and the pathogenesis of various inflammation-driven diseases. One major challenge has been to selectively study resident macrophages in highly heterogeneous organs such as kidney. To address this problem, we adopted a *Translational Ribosome Affinity Purification* (TRAP)- approach and designed a transgene that expresses an eGFP-tagged ribosomal protein (L10a) under the control of the macrophage-specific c-fms promoter to generate c-fms-eGFP-L10a transgenic mice (Mac^TRAP^). Rigorous characterization found no gross abnormalities in Mac^TRAP^ mice and confirmed transgene expression across various organs. Immunohistological analyses of Mac^TRAP^ kidneys identified eGFP-L10a expressing cells in the tubulointerstitial compartment which stained positive for macrophage marker F4/80. Inflammatory challenge led to robust eGFP-L10a upregulation in kidney, confirming Mac^TRAP^ responsiveness *in vivo*. We successfully extracted macrophage-specific polysomal RNA from Mac^TRAP^ kidneys and conducted RNA sequencing followed by bioinformatical analyses, hereby establishing a comprehensive and unique *in vivo* gene expression and pathway signature of resident renal macrophages. In summary, we created, validated and applied a new, responsive macrophage-specific TRAP mouse line, defining the translational profile of renal macrophages and dendritic cells. This new tool may be of great value for the study of macrophage biology in different organs and various models of injury and disease.

## Introduction

Resident monocyte-derived cells are an integral part of the mammalian innate immune system and are found in all solid organs including the kidney^1,2^. In the classical mononuclear phagocyte system (MPS) model, these cells originate from myeloid progenitor cells in the bone marrow and infiltrate the developing embryonal tissues via the vasculature to differentiate into tissue resident macrophages and dendritic cells^3,4^. However, this linear model has been challenged in part by recent studies, which have suggested that many tissue resident macrophages may in fact develop during embryonic development and persist into adulthood independently of blood monocyte input in the steady state^5^. Macrophages and dendritic cells share common monocytic ontogeny and are regarded by many as developmental intermediates^6^.

Macrophages are present at an early time point of development and contribute to organogenesis, angiogenesis, wound repair, inflammation and fibrosis^7^. They are highly heterogeneous immune effector cells that can be divided into subpopulations based on their distinct functions and anatomical location. Interestingly, during homeostasis and upon infection or injury, kidney-resident macrophage populations are replenished by a combination of local proliferation and recruitment of bone-marrow-derived precursors^6^. The canonical function of macrophages is the removal of apoptotic cells and microbes by phagocytosis, whereas dendritic cells mainly function as antigen-presenting cells and activators of T cells^8^. The marker molecules used to identify these cells largely overlap. For instance, dendritic cells also express the classical macrophage marker F4/80 in most non-lymphoid tissues, including the kidney^8^. The precise origin of kidney macrophages is still debated. Recent studies suggest that embryonic macrophage progenitors initially migrate from the yolk sac into the developing kidney where they mature and proliferate^9^. Key regulators of macrophage differentiation, proliferation and survival are the colony stimulating factor (CSF-1) and its receptor CSF-1R (CD115)^10^, that play important roles in the development of the innate immune system, inflammation, tissue repair as well as cancer biology^11^. CSF-1R is encoded by the c-fms proto-oncogene and is expressed specifically and at high levels in monocytes and tissue macrophages^12,13^, myeloid dendritic cells^14^, osteoclasts^15^, microglia, and Paneth cells^16^.

Our knowledge of the biological properties and functions of resident renal macrophages is still incomplete. Whole organ transcriptomic approaches to study macrophage biology in solid tissues have critical limitations that arise from the cellular complexity of many organs including kidney. For instance, more than two dozen distinct cell types can be found in the kidney^17^ and transcriptional analyses from whole tissue will thus only reflect a composite gene expression pattern of all cells, rather than of a single cell population. Laser-capture microdissection (LCM) and fluorescence-activated cell sorting (FACS) have been applied in solid tissues to enrich cell types of interest, but both require considerable, time-consuming manipulation of the tissue, which can significantly alter the naive expression profile of cells^18,19^. This is particularly true for inflammatory cells such as macrophages, that quickly respond to stress signals.

Herein, we present the development and application of a new tool to study *in vivo* expression profiles of macrophages in the kidney and other organs based on the novel Translational Ribosome Affinity Purification (TRAP) strategy^20–22^.

## Results

### Generation of a transgenic macrophage-specific TRAP mouse (Mac^TRAP^)

**Figure 1:**
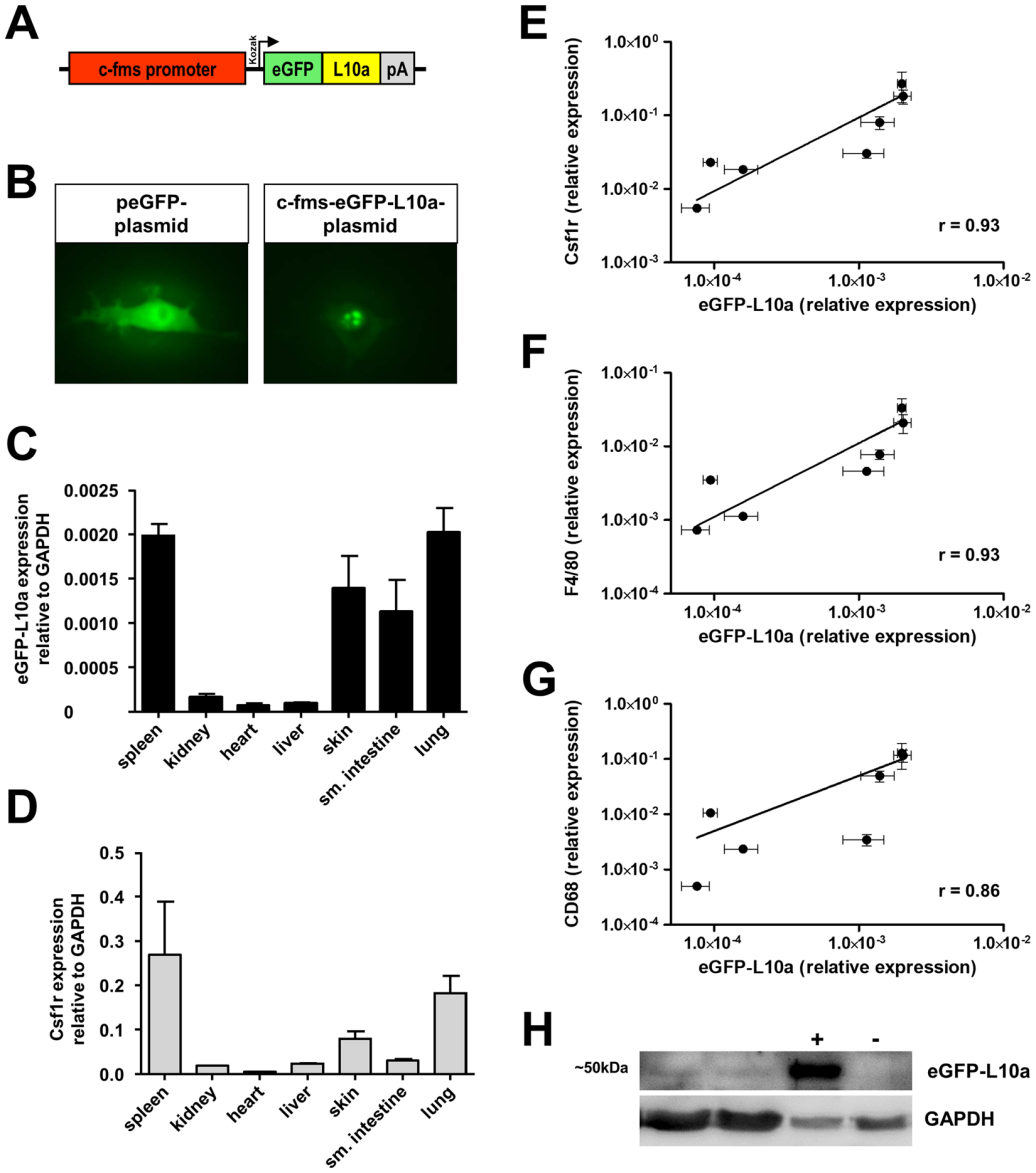
Design and validation of c-fms-eGFP-L10a construct and transgenic Mac^TRAP^ mice. (**A**) Transgene construction. A cassette encoding eGFP fused to the ribosomal protein L10a was inserted downstream of the c-fms promoter/enhancer element. **(B)** *In vitro* validation of the construct by fluorescence microscopy. RAW 264.7 cells were transfected with peGFP (control) or the c-fms-eGFP-L10a plasmid; latter reveal a nucleolar fluorescence signal which is in accordance with the site of ribosome assembly. By contrast, control cells show a homogenous fluorescence pattern throughout the cell body. **(C**,**D)** RT-qPCR of eGFP-L10a and endogenous Csf1r expression in various organs of Mac^TRAP^ mice. Mean ± SEM; n = 5. **(E–G)** Correlation between expression levels of the transgene c-fms-eGFP-L10a and endogenous Csf1r (r = 0.93, p < 0.01), F4/80 (r = 0.93, p < 0.01) and CD68 (r = 0.86, p < 0.05), respectively, across different organs of Mac^TRAP^ mice; data represent mean expression values ± SEM; n = 5. **(H)** Western Blot of spleen lysates from Mac^TRAP^ mice. Anti-GFP antibody detects a band at ~50 kDa, consistent with the expected size of the eGFP-L10a fusion protein. GAPDH served as loading control. Kidney lysates of Podo^TRAP^ mice^21^ and WT mice served as positive control and negative control, respectively (full-length blot is shown in Sup. Fig. S10).

**Figure 2:**
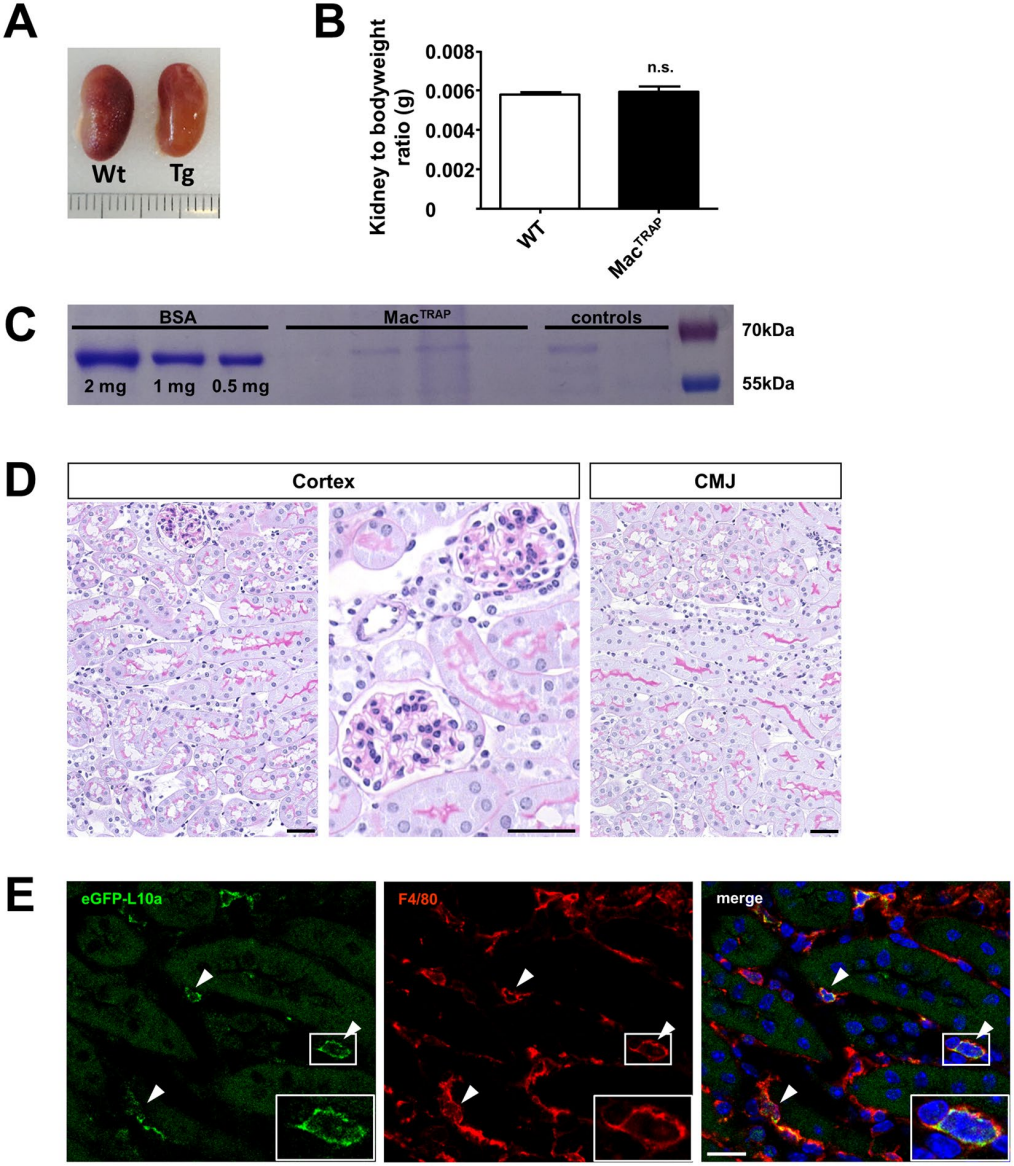
Anatomical and histological characterization of Mac^TRAP^ transgenic mice. (**A**) Kidneys extracted from Mac^TRAP^ mice revealed no gross macroscopical abnormalities when compared to C57BL/6 (WT) mice. **(B)** Kidney-to-body weight ratios were not significantly different in comparison to C57BL/6 (WT) mice; n = 20. **(C)** Coomassie gel loaded with urine samples of Mac^TRAP^ and WT control mice. No significant albuminuria was observed (full-length gel is shown in Sup. Fig. S10). **(D)** PAS staining of kidneys from 9 week old Mac^TRAP^ mice. Kidney architecture appears normal. CMJ = corticomedullary junction. Scale bar: 25 µm. **(E)** Co-staining of eGFP-L10a and macrophage-specific surface marker F4/80 in Mac^TRAP^ kidneys. Scale bar: 25 µm.

First, we engineered a construct containing the well characterized macrophage-specific c-fms promoter/enhancer element, a driver of Csf1r, followed by an eGFP-tagged ribosomal protein L10a (Fig. 1A, Sup. Fig. S1). For validation, we transfected RAW 246.7 macrophages with the c-fms-eGFP-L10a transgene *in vitro*, which demonstrated a distinct nucleolar and perinuclear fluorescence pattern consistent with known cellular sites of ribosomal assembly and localization (Fig. 1B). Next, we generated transgenic c-fms-eGFP-L10a mice (Mac^TRAP^) by using pronuclear injection of the linearized c-fms-eGFP-L10a transgene into F1-hybrid mouse embryos. Litters were of normal size and the transgene inherited in a Mendelian fashion. No developmental or behavioral abnormalities were observed. Next, we quantified eGFP-L10a and endogenous Csf1r expression levels across various organs via qPCR (Fig. 1C,D), demonstrating an excellent correlation with a calculated Spearman correlation coefficient (*r*) of 0.93 (Fig. 1E). Correlations with other classic macrophage-specific markers such as Adgre1 (F4/80) and CD68 were also robust (eGFP-L10a/F4/80: *r* = 0.93; eGFP-L10a/CD68: *r* = 0.86) (Fig. 1F,G). Moreover, the eGFP-L10a fusion protein was detectable by Western Blot in macrophage-rich organs such as spleen (Fig. 1H). Flow cytometry of leukocytes isolated from peripheral blood of healthy Mac^TRAP^ mice detected GFP-fluorescence specifically in CD115^+^ monocytes, but not in mature Ly6g^+^ neutrophils or lymphocytes (Sup. Fig. S2). Macroscopically, organs of Mac^TRAP^ mice revealed no gross anatomical abnormalities. Kidneys were of normal size and kidney-to-body weight ratios comparable to wild-type animals (Fig. 2A,B). No significant albuminuria was detected (Fig. 2C). Histologically, kidney architecture of Mac^TRAP^ mice appeared normal with unremarkable glomeruli and tubules (Fig. 2D).

### Validation of Mac^TRAP^ corroborates macrophage specificity and responsiveness of transgene expression

**Figure 3:**
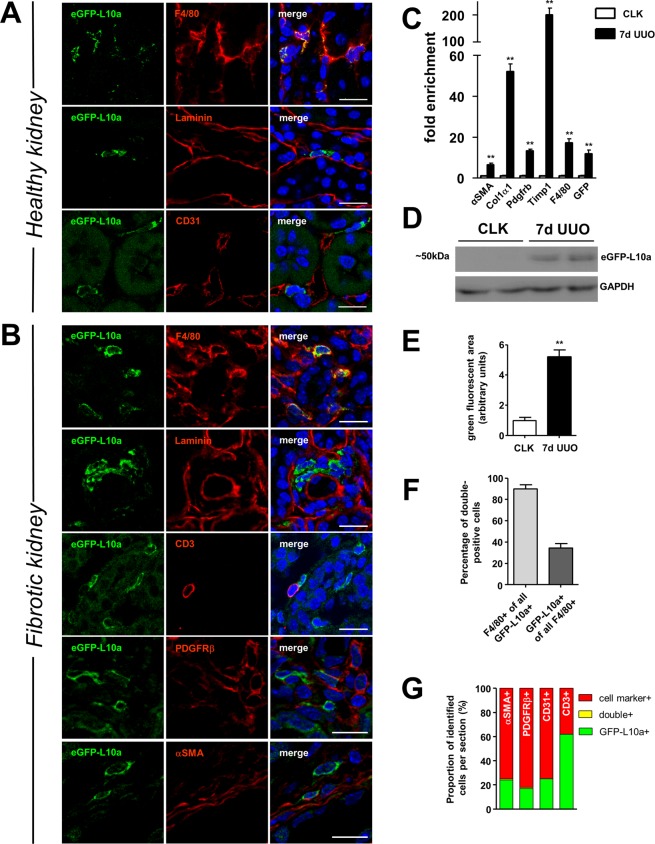
Cell type-specific expression of eGFP-L10a in healthy and fibrotic Mac^TRAP^ kidneys and transgene responsiveness *in vivo*. (**A**) Immunofluorescent staining of healthy Mac^TRAP^ kidneys demonstrates cell type-specific expression of the eGFP-L10a transgene. EGFP-L10a^+^ cells co-stain with macrophage surface marker F4/80 and reside in the tubulointerstitium as highlighted by anti-laminin staining. Endothelial cells (anti-CD31^+^) of peritubular capillaries are closely associated with eGFP-L10a^+^ cells, but no co-staining is observed. Scale bar: 25 µm. **(B)** In fibrotic kidneys (7d UUO), eGFP-L10a^+^ cells remain confined to the tubulointerstitial compartment as illustrated by anti-laminin staining. Co-staining with other interstitial cell markers is minimal (pericyte marker PDGFRβ, myofibroblast marker αSMA) or completely absent (T cell marker CD3). Scale bar: 25 µm. **(C)** RT-qPCR analysis shows strong upregulation of eGFP-L10a in fibrotic kidneys (7d UUO) along with macrophage- and fibrosis-related markers compared to CLK kidneys, indicating responsiveness of transgene expression upon proinflammatory challenge (**p < 0.01); data are shown as mean ± SEM; n = 5. (**D**) Western Blot of protein lysates from 7d UUO Mac^TRAP^ kidneys indicates robust upregulation of the eGFP-L10a protein compared to CLK controls when probed with an anti-GFP antibody. A protein band was detected at ~50 kDa corresponding to the expected molecular weight of the eGFP-L10a fusion protein. GAPDH antibody was used as internal loading control (full-length blot is shown in Sup. Fig. S10). (**E**) Quantitative analysis of green fluorescent area in Mac^TRAP^ kidney sections shows a significant increase in eGFP-L10a signals at 7 days of UUO compared to CLK controls (5.2 fold, **p < 0.01); data are shown as mean ± SEM; n = 3. (**F**) Proportion of cells with double-positivity for eGFP-L10a and macrophage-marker F4/80; a total of 1205 cells were counted; n = 5 mice. Data are shown as mean ± SEM. (**G**) Quantification of additional co-staining. Co-staining of eGFP-L10a^+^ cells (green) for other cell markers (red) was minimal (yellow) or absent. Bars show proportions based on all identified cells; n = 3–5 mice.

Next, we examined the identity of eGFP-L10a expressing cells in renal tissue by immunostaining. EGFP-L10a^+^ cells were confined to the tubulointerstitium and overwhelmingly positive for the macrophage marker F4/80 (Figs. 2E and 3A). Moreover, they showed a close association to tubular basement membranes and endothelial cells (Fig. 3A). Since macrophage expansion is a hallmark of many renal pathologies including acute kidney injury and tubulointerstitial fibrosis, we tested the hypothesis whether c-fms-eGFP-L10a transgene expression in Mac^TRAP^ was inducible and hence responsive to inflammatory challenge *in vivo*^7^. We subjected Mac^TRAP^ mice to unilateral ureteral obstruction (UUO) to precipitate kidney fibrosis and detected a robust induction of eGFP-L10a in UUO kidneys on a transcriptional as well as protein level when compared to uninjured contralateral kidneys (CLK) (Fig. 3B–D). This increase in eGFP-L10a expression was paralleled by a significant upregulation of classical markers of fibrosis including αSMA, collagen1α1 and F4/80 (Fig. 3C). In line with qPCR and Western blot data, immunofluorescence microscopy revealed a sharp increase in eGFP-L10a^+^ cells in UUO kidneys compared to CLK, thus validating the responsiveness of the c-fms-eGFP-L10a transgene in Mac^TRAP^ mice upon proinflammatory and profibrotic stimulation (Fig. 3D,E). Importantly, eGFP-L10a^+^ cells remained confined to the tubulointerstitial compartment during fibrosis. To further verify the exact identity of eGFP-L10a^+^ cells in the Mac^TRAP^ tubulointerstitium, we conducted a number of additional cell-specific immunostainings. Quantitative analyses showed that the vast majority of readily detectable eGFP-L10a^+^ cells were also positive for the macrophage marker F4/80 (92.4% ± 7.5%), suggesting a high level of specificity. Conversely, the eGFP-L10a^+^ fraction of all F4/80^+^ cells was lower (38.8% ± 8.1%) (Fig. 3F). Co-staining for other cell markers such as the pericyte marker PDGFRβ, myofibroblast marker αSMA, endothelial cell marker CD31 and T cell marker CD3 was minimal or completely absent (proportion of double-positive cells: 4.1% ± 1.3% for PDGFRβ, 4% ± 2.8% for αSMA, 0% for CD31 and 0% for CD3, respectively; relative proportions based on all identified cells are shown in Fig. 3G). Moreover, immunostaining for mature neutrophils in fibrotic kidney tissue and tail skin biopsies found that only a very small fraction of Ly6g^+^ neutrophils was also positive for eGFP-L10a as detected by fluorescence microscopy (double positive fraction: 0.42% and 0.98%, respectively) (Sup. Fig. S3). Of note, transgene expression was not restricted to kidney and eGFP-L10a^+^ cells were, as expected, also detected in other tissues of Mac^TRAP^ mice including spleen, lung, skin, liver, heart and aorta (Sup. Figs. S4 and S5).

### Capturing macrophage-specific polysomal mRNA from Mac^TRAP^ kidneys

**Figure 4:**
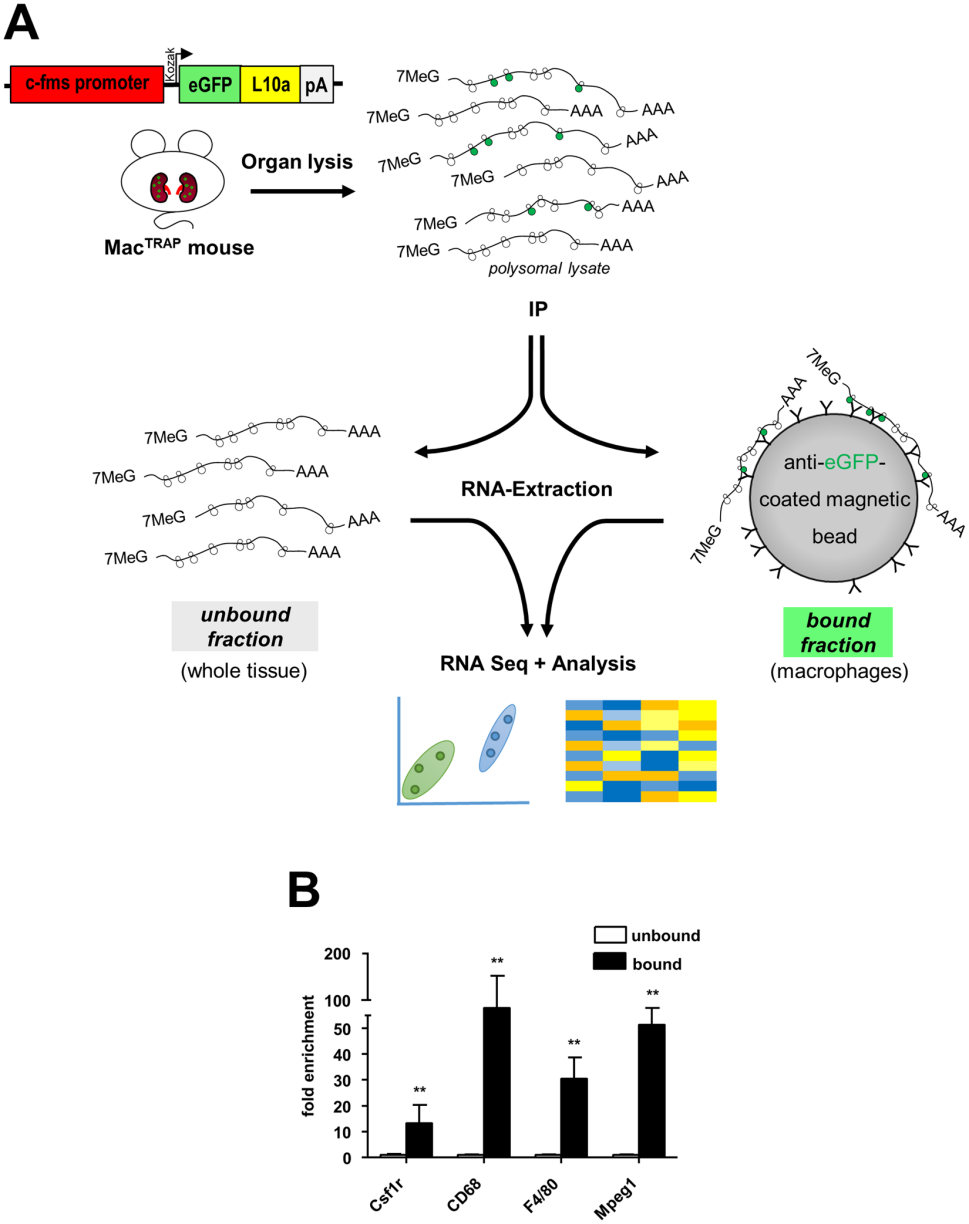
Methodology and validation of TRAP in Mac^TRAP^ mice. (**A**) Schematic illustrating the principle strategy and procedure of TRAP. Mac^TRAP^ kidneys are harvested, immediately homogenized and lysates subjected to immunoprecipitation (IP) with anti-eGFP-antibody-coated magnetic beads. Magnetic beads bind only to polysomes with the eGFP-tagged L10a protein (green). These bound polysomes represent the cell-specific (bound) fraction, which contains highly enriched RNA messages of renal macrophage origin. The unbound fraction represents RNA messages of whole kidney. Extracted RNA is then processed for downstream applications such as RNA-Seq and subsequent bioinformatical analyses. **(B)** Validation of macrophage-specific mRNA enrichment from Mac^TRAP^ kidneys. RT-qPCR analysis demonstrates strong enrichment of established macrophage marker genes in bound versus unbound kidney fractions (**p < 0.01); data are shown as mean ± SEM; n = 5.

Next, we aimed at capturing macrophage-specific RNA from whole kidney tissue of healthy Mac^TRAP^ mice by applying a modified TRAP protocol^23^ (Fig. 4A). IP-extracted polysomal mRNA was quantified and quality controlled (Sup. Fig. S6). We performed RT-qPCR analyses to verify the specificity of immunoprecipitated (bound) mRNA fractions screening for known macrophage marker genes as surrogates. We found a strong overrepresentation of *CD68* (97.8 fold), *Mpeg1* (45 fold), *Adgre1*(F4/80) (30.5 fold) and *Csfr1* (13.3 fold) in kidney bound fractions compared to whole kidney unbound fractions (Fig. 4B). Taken together, these data indicate a robust enrichment of macrophage-derived transcripts extracted from Mac^TRAP^ whole kidney tissue via TRAP technology.

### Establishing the translational profile of renal macrophages by RNA-Seq

**Figure 5:**
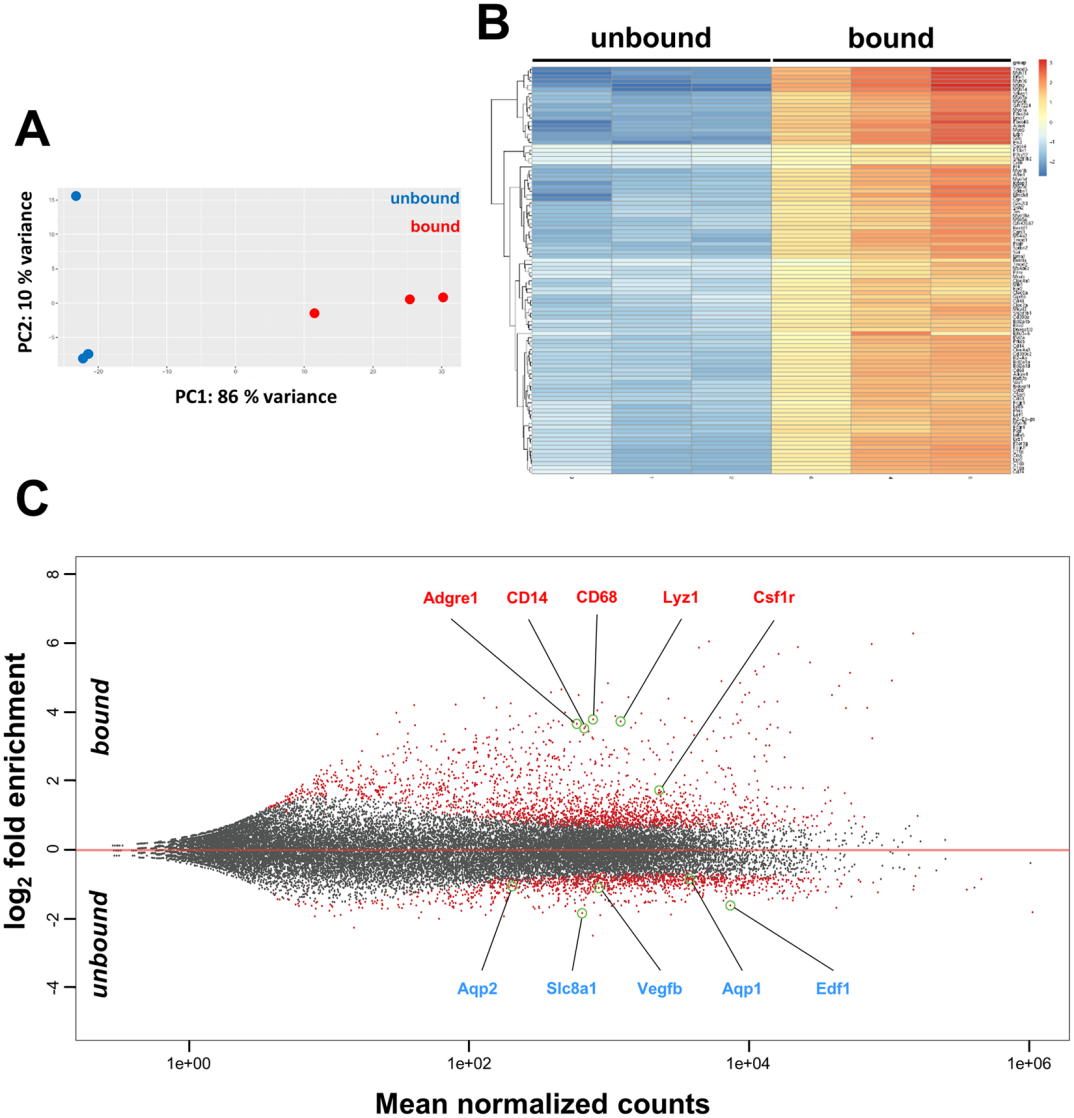
RNA-Seq data analysis of TRAP-extracted polysomal RNA from Mac^TRAP^ kidneys. (**A**) Principle component analysis (PCA) shows distinct clustering between unbound (blue dots) and bound (red dots) fractions extracted from kidneys of 9-week- old Mac^TRAP^ mice. **(B)** Heatmap of RNA-Seq data illustrating the 100 most differentially expressed genes between bound (renal macrophage-specific) vs. unbound (whole kidney) fractions. **(C)** MA plot of RNA-Seq data shows gene expression in relation to mean normalized counts. Macrophage-specific transcripts are robustly enriched in the bound vs. unbound fraction (selected genes are highlighted, *red font*), whereas transcripts representative of other cell types are depleted (*blue font*); red dots represent differentially expressed genes with p adj. < 0.05.

To establish an unbiased translational profile of resident renal macrophages and potentially dendritic cells, we next performed RNA-Seq (Illumina HiSeq) on TRAP-extracted polysomal RNA (bound fractions) as well as whole kidney RNA (unbound fractions) from Mac^TRAP^ renal tissue. First, we analyzed RNA-Seq data by Principle Component Analysis (PCA) and found a distinct clustering of both groups (Fig. 5A). Next, we performed hierarchical clustering analysis visualized by heatmap, highlighting the most differentially expressed genes between the two groups (Fig. 5B). Consistent with qPCR results, the MA plot demonstrated robust overrepresentation of classical macrophage markers including *Adgre1* (F4/80), *Csf1r* (c-fms), *CD14*, *CD68*, *Itgam* (CD11b) and *Lyz1*, but not of genes typically expressed in other kidney-resident cell types (Fig. 5C). To define the gene expression signature of resident renal macrophages and dendritic cells at homeostasis, we first filtered all transcripts that met a significant ≥2 fold enrichment (p < 0.05) in bound fractions compared to whole kidney, yielding a total of 1448 genes, which were hence considered macrophage-specific (Sup. Table 1). In this gene list, we readily identified many well characterized macrophage-specific and associated genes such as *Adgre1 (F4/80), CD68 (macrosialin), CD86, CD14, Lyz1 (lysozyme 1), Lyz2 (lysozyme 2), Msr1 (macrophage scavenger receptor 1), Mrc1 (mannose receptor C-type 1), C1q, Fcgr3 (CD16)*, as well as *Itgam (CD11b)* and *Itgb2 (Cd18)*, which together form the Macrophage-1 antigen complement receptor. The Mac^TRAP^ dataset also revealed other genes typically expressed in macrophages such as various chemokine ligands (e.g. *Ccl4, Ccl6, Ccl7, Ccl8, Ccl9, Ccl12, Cxcl4, Cxcl9*) and receptors including *Cx3cr1*, which has been used as a marker for yolk-sac derived macrophages^24–26^. Typical macrophage-determining transcription factors such as *PU.1 (Spi1), Runx1, Runx3, Irf5, Irf8, Pparg, Mafb* and *Batf3* were likewise significantly enriched in the bound fraction, as were transcription factors *Nfatc1* and *Irf9*, which have been recently reported to be presumably kidney macrophage-specific^27^.

For further assessment, we cross-referenced the translational kidney Mac^TRAP^ data with two recently published datasets on kidney macrophage expression^22,27^. Comparison of the Mac^TRAP^ dataset with the “core” macrophage gene list reported for P21 kidney macrophages by Mass *et al*. demonstrated a 70% overlap between significantly enriched genes^27^. Overall, we found more than one hundred genes shared among all three data sets including *Lyz2, C1q, Csf1r, CD68, CD86* and *CD14* (Sup. Fig. S7, Sup. Table 2).

However, we also identified a substantial number of additional transcripts that were exclusively enriched in TRAP-extracted polysomal RNA from Mac^TRAP^ kidneys (Sup. Table 3). For instance, we uncovered a number of genes with functions in actin cytoskeleton- and podosome-formation (e.g. *Myo1e, Svil*) as well as interferon inducibility (e.g. *Ifi204, Ifi211*). Other genes we identified are reportedly involved in directed processes such as phagocytosis (*Myo10*) and autophagy (e.g. *Atg7, Dctn4, Ambra1*).

### Systematic analysis of the renal macrophage translational profile underlines functions in immunity and actin dynamics

Next, we used DAVID (database for annotation, visualization and integrated discovery) bioinformatics resources to classify the predicted cell functions derived from the Mac^TRAP^ macrophage-specific signature. Using the gene functional classification tool with an input of all 1448 genes, we identified 59 gene clusters (Sup. Table 4). In a next step, we generated a gene functional annotation chart reporting 994 biological terms. Most robust enrichments were found for immune-related terms including “*immunity”* (5.1 fold, 115 genes, p = 7.4E-50), “*immune system response”* (4.6 fold, 109 genes, p = 1.7E-42), “*innate immunity”* (5.4 fold, 72 genes, p = 2E-32), “*innate immune response”* (3.3 fold, 82 genes, p = 1.0E-21) and “*actin-binding”* (5.9 fold, 83 genes, p = 6.6E-41) (Sup. Table 5). When testing for terms of tissue expression, the term *“macrophage”* yielded the highest confidence (122 genes, p = 3.3E-31). Of note, another term with substantial enrichment was *“dendritic cell”* (28 genes, p = 5.8E-6) (Sup. Tables 6 and 7). Next, we performed gene functional annotation clustering identifying 106 clusters with a group enrichment score (GES) of ≥1, with cluster “*immunity/immune system process”* ranking highest (GES: 35.9). Other high ranking annotation clusters included “*actin-binding”* (GES: 32.1), “*myosin complex”* (GES: 7.71) and “*phagosome”* (GES: 3.88) (Sup. Table 8, Sup. Fig. S8).

**Figure 6:**
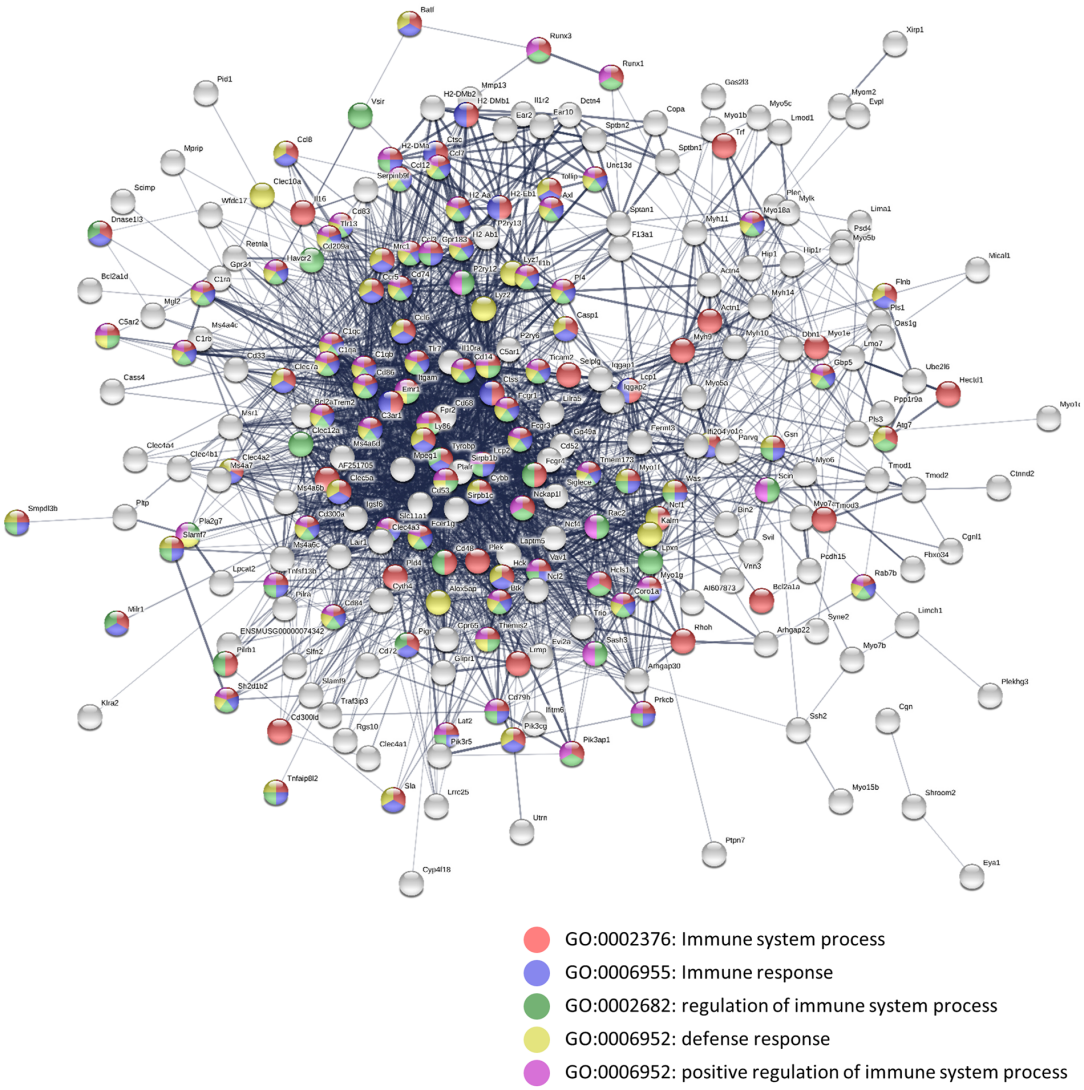
StringDB protein interaction network based on the top 300 overrepresented mRNAs in bound kidney fractions of Mac^TRAP^ mice. Colored nodes represent proteins individually assigned to the five most significantly enriched GO biological processes. Line thickness indicates the strength of confidence for each interaction. Minimum required interaction score for the network was set at 0.4.

We next sought to define pathways and biological processes likely to be active in resident kidney macrophages at homeostasis. To this end, we conducted gene ontology (GO) functional enrichment and pathway analyses using STRING^28^. We generated a macrophage interaction network by applying stringent criteria and including only the top 300 enriched genes (Fig. 6). The top five enriched biological processes turned out to associate with “*immune system response”* (111 genes, p = 2.18E-45), “*immune response”* (80 genes, p = 2.48E-39), “*regulation of immune response”* (80 genes, p = 2.12E-32), “*defense response”* (74 genes, p = 1.17E-29) and “*positive regulation of immune system process”* (62 genes, p = 8.14E-28) (Sup. Table 9, Sup. Fig. S9).

**Figure 7:**
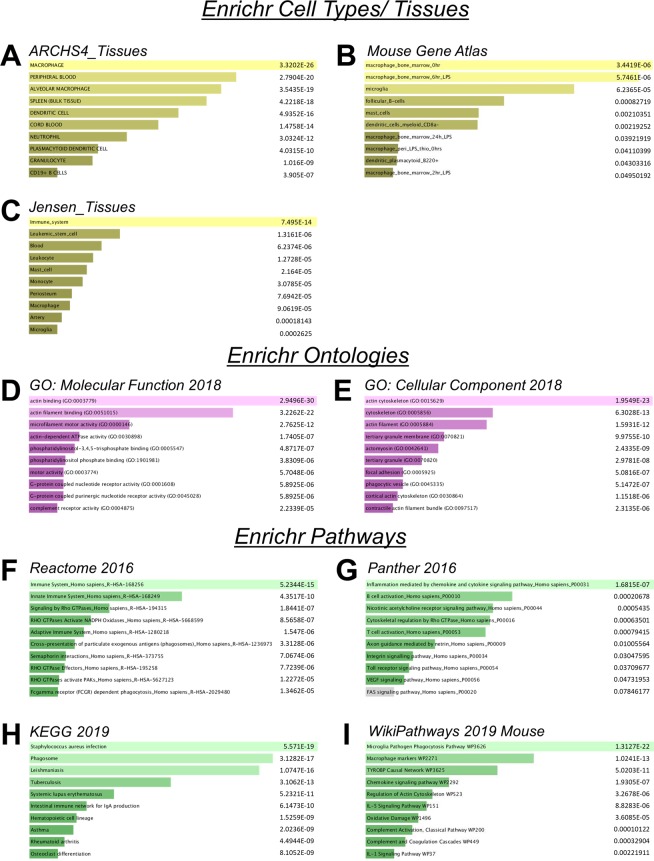
Comprehensive gene set enrichment analysis (GSEA) based on the top 300 overrepresented genes in kidney macrophages (Mac^TRAP^ bound fractions) using Enrichr. (**A**–**C**) GSEA for *cell types and tissue* reveals significant overrepresentation of immune-related terms including *“macrophage”, “alveolar macrophage”, “dendritic cell”, “macrophage_bone_marrow_0hr”* and *“monocyte”*. **(D** , **E)** GSEA for *molecular function* and *cellular component* gene ontologies demonstrate enrichment for terms such as “*actin binding”*, *“actin filament binding”, “phagocytic vesicle”* and “*microfilament motor activity*”. **(F–I)** GSEA for various *signaling and metabolic pathways* identifies terms such as “*immune system”, “microglia pathogen phagocytosis pathway”, “macrophage markers”, “phagosome*” and *“inflammation mediated by chemokine and cytokine signaling pathway”*. Bar graphs show enriched terms sorted by p value ranking.

Finally, we used Enrichr^29^ for comprehensive gene set enrichment analysis (GSEA) including the top 300 most strongly overrepresented genes in Mac^TRAP^ bound kidney fractions (Fig. 7). GSEA for *cell types and tissues* showed significant enrichment for distinct terms such as “macrophage” (p = 3.32E-26), “macrophage_bone marrow_0hr” (p = 3.44E-06), “alveolar macrophage” (p = 3.54E-19), “microglia” (p = 6.24E-05), “leukocyte” (p = 1.27E-05) and “dendritic cell” (p = 4.93E-16) across various catalogues (Fig. 7A–C). For *molecular function*, Enrichr GO implicated very strong associations with “actin binding” (p = 2.95E-30) and “microfilament motor activity” (p = 2.76E-12) (Fig. 7D). For *cellular component*, terms such as “actin cytoskeleton” (p = 1.95E-23) and “phagocytic vesicle” (p = 5.15E-07) were amongst the most prominent (Fig. 7E). Enrichr GSEA filtering for *signaling and metabolic pathways* suggested strong associations with “immune system” (p = 5.23E-15), “inflammation mediated by chemokine and cytokine signaling pathway” (p = 1.68E-0.7), “staphylococcus aureus infection” (p = 5.57E-19) and “phagosome” (p = 3.13E-17) (Fig. 7F–H). Importantly, based on the Wiki pathways 2019 mouse resource (Fig. 7I), the two most enriched terms were *“macrophage markers”* (p = 1.02E-13) and *“microglia pathogen phagocytosis pathway”* (p = 1.31E-22), further attesting to the validity and strength of Mac^TRAP^.

## Discussion

Resident macrophages and dendritic cells reside in various tissues and organs including kidney, and have been reported to play key roles in health and disease^30^. Our current understanding of these cells is incomplete and a better *in vivo* characterization is needed. However, to study single cell populations in complex organs such as kidney with its numerous unique cell types at different and at times varying densities is challenging. Several techniques including laser-capture microdissection, dynabead perfusion and FACS have been applied to enrich substrates of selective cell populations from whole tissues, most of which, however, come with considerable drawbacks due to the inherent necessity for long and cumbersome tissue preparation. Here, we report on the development of a novel macrophage-specific TRAP mouse line (Mac^TRAP^), that we have designed to express an eGFP-tagged ribosomal fusion protein, eGFP-L10a, under the control of the c-fms promoter, thus allowing enrichment of macrophage- and dendritic cell-derived messages in a one-step affinity purification procedure. This approach eliminates the risk of cellular transcriptional stress responses induced by tedious tissue processing. Moreover, expression profiles derived from polysomal RNA have been shown to more precisely reflect actual protein synthesis compared to RNA isolated by standard techniques^20,23,31^. Following the generation and validation of the Mac^TRAP^ mouse line, we extracted polysomal mRNA from healthy Mac^TRAP^ kidneys to establish a cell-specific and comprehensive gene expression profile of resident kidney macrophages, giving detailed insights into biological functions and pathway activities of these cells in steady-state.

Our immunohistological analyses showed that the vast majority of eGFP-L10a^+^ cells in Mac^TRAP^ kidneys stain positive for the macrophage marker F4/80. A small fraction of eGFP-L10a^+^ cells was found to be F4/80^−^, which may be explained by the reported existence of macrophage subsets that lack F4/80 expression^32^. Similarly, as visualized by fluorescence microscopy, we also found a fraction of F4/80^+^/eGFP-L10a^−^ cells, possibly reflecting either low and thus undetectable eGFP-L10a expression levels, mosaic expression of the transgene, and/or also macrophage heterogeneity. Importantly, co-staining for markers of other cell types including pericytes, myofibroblasts, endothelial cells, T cells and neutrophils indicated no or only minimal transgene expression in these cells, further supporting macrophage specificity of Mac^TRAP^. Interestingly, Sasmono *et al*. have previously reported on a *fms*-EGFP reporter mouse line, aka *MacGreen* mouse, which exhibits reporter expression not only in macrophages, but, unexpectedly, also in granulocytes^33–35^. This phenomenon is still not fully understood, especially since granulocytes, despite featuring a surprisingly complex transcriptome, have a comparatively simple proteome with only few abundant proteins and demonstrably lack protein expression of the endogenous CSF1R (CD115) surface receptor^35–37^. The authors have speculated, that the more stable eGFP reporter may be retained in progeny from the common myeloid precursor in *MacGreen* mice^34^. However, our data suggest that mature neutrophils from Mac^TRAP^ mice do not appear to express the transgene-encoded eGFP-L10a fusion protein at a substantial level – or at least not high enough to allow visual detection. Nonetheless, we cannot exclude the possibility of eGFP-L10a protein expression in neutrophils at a lower level that may not produce sufficient fluorescent intensity to pass the detection threshold. Also, we cannot completely rule out the possibility that with different stimulation Mac^TRAP^-derived neutrophils may acquire new characteristics that could potentially be associated with transgene induction.

Kidney macrophage profiling using the Mac^TRAP^ system identified several hundred significantly overrepresented genes, which comprised markers of both M1 (classically activated) and M2 (alternatively activated) macrophages. However, the M1-M2 paradigm remains controversial and may only reflect a simplified operational concept. There is now data to suggest that the increasingly recognized diversity and specialization of tissue resident macrophages may indeed be controlled by the environment in a tissue- and niche-specific manner, resulting in various subpopulations. For instance, a recent study suggests that the murine kidney may contain at least five discrete subpopulations, which might not be simply classified into conventional entities^38^.

Besides classical macrophage markers such as *CD68, CD11b* and *Adgre1(F4/80)*, we also identified a number of genes characteristic of dendritic cells. To date, there is no definitive cellular demarcation between macrophages and dendritic cells, and markers to identify either cell type overlap considerably^39^. Both cell types are closely related and share similar features, however macrophages seem to generally express higher levels of Csf1r when compared to dendritic cells^14^.

We have previously reported on a floxed TRAP mouse line that had been generated to allow induction of permanent eGFP-L10a expression following Cre-mediated recombination^22^. Generating Lyz2-Cre;CAG-eGFP-L10a^fl/fl^mice (Lyz2-Cre-L10a) led to constitutive, high-level eGFP-L10a expression in Lyz2-expressing cells, including macrophages, but irrespective of their subsequent cell fate. Comparison of the *in vivo* Mac^TRAP^ RNA-Seq dataset with the previous Lyz2-Cre-L10a microarray-based transcriptome showed a substantial overlap of hits including *Adgre1 (F4/80), CD68, C1qc* and *CD14*, but also important differences. Of note, a number of kidney macrophage-specific genes that we were able to identify exclusively with Mac^TRAP^ (Sup. Table 3), have attributed functions in podosome formation, phagocytosis and autophagocytosis. For instance, myosin Myo1e has been described as a component of macrophage podosomes that may be required for optimal MHC-II surface expression on macrophages^40,41^. Myosin Myo10 on the other hand has been shown to be recruited to phagocytic cups and to play a role in FcγR-mediated phagocytosis executed by macrophages^42^. The membrane-associated protein supervillin 1, encoded by *Svil1*, has been studied in primary human macrophages and demonstrated to be a key regulator of podosome turnover, podosomal matrix degradation and, interestingly, macrophage polarization^43^. Mac^TRAP^ also identified genes in resident kidney macrophages that are likely associated with (auto)phagocytotic processes. For example, there is evidence to suggest that autophagy related protein 7 *(Atg7)* and dynactin 4 *(Dctn4)* play a role in the autophagy-mediated clearance of *P. aeruginosa* by alveolar macrophages^44,45^. Atg7 has been described to have functions in LC3-dependent autophagy following TLR stimulation of macrophages, whereas the dynein-dependent motor Dctn4 has been shown to move autophagosomes along microtubules into lysosomes for degradation of damaged proteins and microbes. Moreover, we determined interferon-inducible gene 204 *(Ifi204)* to be an additional part of the renal macrophage translational signature, which has been reported to act as a potential regulator of the balance between macrophage proliferation and differentiation^46^. Interestingly, a very recent study has found that Ifi204 also plays a role in extracellular bactericidal activity of phagocytes through enhancing extracellular trap formation, thus promoting bacteria eradication and contributing to host defense against infection^47^.

A major strength of the Mac^TRAP^ approach is, that it captures macrophages with ongoing Csf1r-expression at the time of harvest quasi in real time, which may explain part of the distinctness of the herein reported translational profile when compared to the other models. Gene functional classification and annotation, GO term as well as GSEA analysis of the Mac^TRAP^ dataset revealed pronounced enrichment of immune-and actin/cytoskeleton-related terms. These findings are consistent with the assumed roles of macrophages in defensive mechanisms and immune responses as well as the growing body of evidence for the complexity and versatility of their actin filament cytoskeleton during migration, podosome formation and phagocytosis^48–52^. Of note, validation of the Mac^TRAP^ mouse included testing for responsiveness of transgene expression in kidney tissue, demonstrating a robust, *bona fide* eGFP-L10a induction upon inflammatory challenge *in vivo*. Furthermore, c-fms-eGFP-L10a expression is not restricted to macrophages of only the kidney of Mac^TRAP^ animals. We thus expect this TRAP line to be a valuable and universal tool for researchers interested in the study of macrophage biology as it may be applied in many different organs and disease models.

In summary, we have generated a novel macrophage-specific Mac^TRAP^ mouse line to facilitate the study of macrophages in complex tissues including the kidney. This tool will help to elucidate the macrophage biology during health and disease *in vivo* and may lead to the discovery of new therapeutic targets in inflammation-driven pathologies of the kidney and other organs.

## Methods

### Generation of transgenic c-fms-eGFP-L10a mice and maintenance

We first engineered an eGFP-L10a transgene under the control of the macrophage-specific promoter c-fms using standard cloning techniques. An adapter was added to the c-fms promoter (Topo cloning) to create a new Pac1 restriction site. The eGFP-L10a- cassette was integrated into the backbone of the c-fms plasmid following prior Pac1/Sal1 digestion. The construct (Sup. Fig. S1) was then sequenced and validated *in vitro*. Finally, Mlu1/Sal1 digestion produced the linearized complete transgene containing the c-fms promoter followed by the eGFP-L10a encoding sequence which was then successfully integrated into the genome of F1-hybrid mouse embryos (Gene Modification Facility, Harvard University) via pronuclear injection. All mouse studies were performed according to the animal experimental guidelines for laboratory mice issued by the Animal Care and Use Committee (IACUC) at Harvard University and Philipps-University Marburg. The experimental protocol was approved by the IACUC committee at Harvard University. Transgenic mice were maintained on a mixed C57BL/6JxDBA/2 background. Genomic DNA was obtained from tail biopsies and genomic integration of the c-fms-eGFP-L10a transgene verified via PCR-based genotyping using the following primers: sense: 5′-GGCATCGACTTCAAGGAGGA-3′, antisense: 3′-GGTCGTAGTTCTTCAGGCTGA-5′.

### Unilateral ureteral obstruction (UUO)

UUO surgery was performed as previously described^53^. In brief, mice were anesthetized with pentobarbital sodium i.p. (60 mg/kg) prior to surgery and placed in a prone position. The left kidney was accessed by retroperitoneal approach, the left ureter identified and ligated twice with a 4–0 silk suture. Seven days after UUO, mice were euthanized and organs harvested and processed for further studies.

### Mouse tissue preparation and histology

Mice were euthanized under deep isoflurane anesthesia and immediately perfused via the left ventricle with ice-cold PBS for 1 min. Kidneys were harvested, cut in transversal sections, fixed in freshly prepared 4% paraformaldehyde and 5 μm cryosections (Tissue Tek, Sakura) were stained using the following primary antibodies: rat anti-F4/80 (Abcam, 1:1000), rat anti-CD31 (eBioscience, 1:600), rabbit anti-CD3 (eBioscience, 1:300), rabbit anti-laminin (Sigma, 1:2000), rat anti-PDGFRβ (eBioscience, 1:800), mouse anti-αSMA (Sigma, 1:2500), rat anti-Ly6g (BD Pharmingen, 1:500). Sections were subsequently incubated with corresponding Cy3- or Cy5-conjugated secondary antibodies (Jackson ImmunoResearch, 1:400). For PAS staining, kidney sections were fixed in formalin. Images were captured using Nikon C1 confocal and Zeiss Axio Observer Z1 microscope, respectively.

### Western Blot

Western blotting was performed as previously described^23^. In brief, 10 mg of tissue was added to 500 µl ice cold radioimmunoprecipitation (RIPA) buffer containing a protease inhibitor (Roche) and homogenized on ice using a rotor-stator homogenizer (IKA) followed by centrifugation at 13,000 rpm at 4 °C for 30 min. Protein lysates were incubated in 4x Roti load sample buffer (Roth) for 5 min at 90 °C and resolved by SDS-PAGE (12% gels). Proteins were then transferred to PVDF membranes (Bio-Rad) using the Mini-Protean system (Bio-Rad). After blocking nonspecific binding, PVDF membranes were incubated with primary antibodies, including chicken anti-GFP (Aves Labs; 1:2000) for eGFP-L10a detection. Anti-GAPDH (Bethyl Labs, 1:5000) and anti-β-actin antibody (Santa Cruz, 1:10000) served as loading control. Membranes were subsequently probed with HRP-conjugated secondary antibody (Santa Cruz Biotechnology/ Bethyl Labs, 1:10000). Chemiluminescent detection of Proteins was performed using Clarity^TM^ ECL substrate (Bio-Rad) on the Fusion FX (Vilber) chemiluminescent imaging platform.

### RT-qPCR

RNA was extracted from tissue using standard techniques (RNAeasy kit; Qiagen). Purity was determined based on A_260/280_ ratios (Nano Drop, Thermo Fisher). 1 µg of RNA was reverse transcribed for each sample using iScript^TM^ reverse transcriptase (BioRad). Real-time quantitative PCR was performed on the ABI Prism 7500 Real-Time PCR System using iTaq universal SYBR^®^ Green reaction mix (Bio-Rad). The specific primers used for RT-qPCR are listed in Sup. Table 10. Expression values were normalized to the housekeeping gene GAPDH.

### Translational ribosome affinity purification (TRAP)

Polysomal RNA from whole organ lysates was extracted and purified as previously described^21^. In brief, mice were euthanized and perfused with ice-cold PBS under deep isoflurane (Baxter) anesthesia, kidneys removed and decapsulated, rapidly microdissected and transferred to 1 ml of ice-cold polysome extraction buffer. Samples were subsequently homogenized on ice using a Rotor-Stator homogenizer (IKA). Dynabeads (MyOne T1 Dynabeads, Invitrogen) coated with monoclonal anti-GFP antibodies (clones 19F7 and 19C8, 50 µg each per IP; Rockefeller) were added to the post-mitochondrial supernatant and incubated at 4 °C with orbital rotation for 4 hours. Beads were collected on a magnetic rack after incubation, washed repeatedly with ice-cold high-salt wash buffer and resuspended in Qiagen RNeasy lysis buffer. RNA was purified using RNeasy MinElute Cleanup Kit (Qiagen) including on-column DNase1 digestion (Qiagen).

### RNA quality control and RNA-Seq

For assessment of RNA quality and yield, purified RNA was measured using the Agilent 2100 Bioanalyzer^®^ system (Agilent Technologies) (Sup. Fig. S6). RNA samples with sufficient yield and RIN ≥ 9 were processed according to an ultra-low input protocol at DKFZ Heidelberg genomics core facility using Illumina HiSeq2000 RNA-Sequencing. Fast QC reports were analyzed after each run for quality control. FASTQ Mean Sequence Quality (Phred Score) was over 36 for each sample.

### Computational analysis of RNA-Seq data

The RNA-Seq data were mapped against the mouse reference genome GRCm38 using Hisat2^54^. The obtained SAM files were converted to BAM files using SAMtools “sort” command^55^ and subsequently made compatible for usage of the “.gtf” file of ENSEMBL (ftp://ftp.ensembl.org/pub/release-89/gff3/mus_musculus/). The resulting files were analyzed with DESeq2^56^. For the different representations the adjusted p value cut-offs as specified by DESeq2 were used. DAVID (database for annotation, visualization and integrated discovery) was used for functional interpretation and visualization of overrepresented genes. GO functional gene set enrichment and pathway analyses were performed using STRING database^28^ and Enrichr^29^.

### Statistics

Data are shown as mean ± SEM if not otherwise indicated. Statistical analysis was performed and graphs prepared using Prism software. The unpaired Student’s *t* test was used to determine differences between groups. P values of less than 0.05 were considered statistically significant.

## Supplementary information

Supplementary information .

## Data Availability

Raw data are deposited at GEO: GSE136265.
