# Supplementary material for: Development of a new macrophage-specific TRAP mouse (MacTRAP) and definition of the renal macrophage translational signature
Source: Sci Rep. 2020 May 5;10:7519. doi: 10.1038/s41598-020-63514-6 (PMC7200716; doi:10.1038/s41598-020-63514-6)
Supplement: Supplementary file 1 — Supplementary information . [file 41598_2020_63514_MOESM1_ESM.pdf]

Supplemental Figures

Development of a new macrophage-specific TRAP mouse (Mac^{TRAP}) and definition of the renal macrophage translational signature

Andreas Hofmeister, Maximilian C. Thomassen, Sabrina Markert, André Marquardt, Mathieu Preußner, Martin Rußwurm, Ralph Schermuly, Ulrich Steinhoff, Hermann-Josef Gröne, Joachim Hoyer, Benjamin D. Humphreys, Ivica Grgic

Correspondence: Ivica Grgic MD, Klinikum der Philips-Universität Marburg, Baldingerstrasse 1, 35043 Marburg. Phone: +4964215861736, email: grgic@med.uni-marburg.de

Sup. Fig. S1

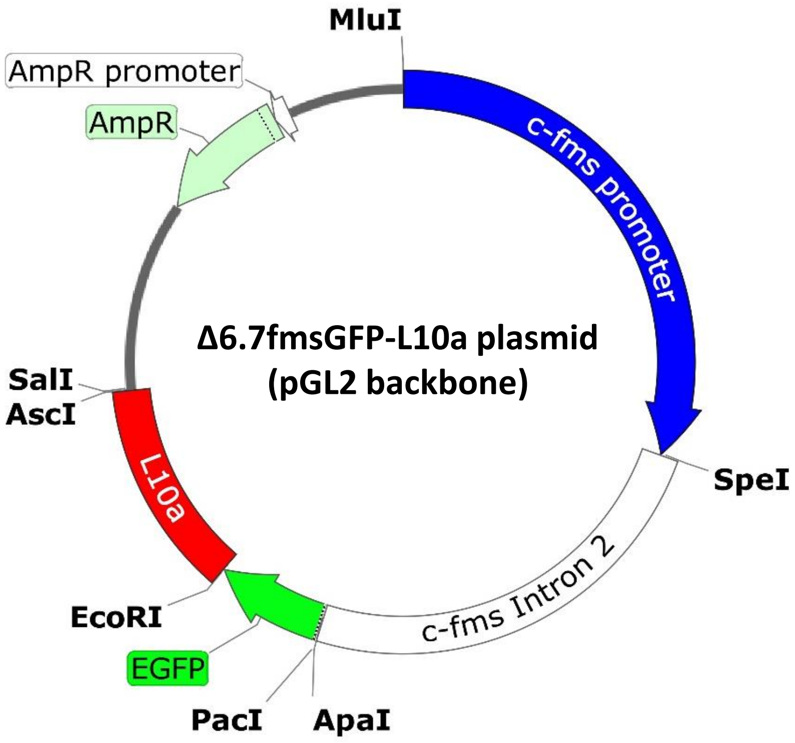

Sup. Fig. S1: Plasmid map of the engineered c-fms-eGFP-L10a expression vector. Mlu1/Sal1 digestion was used for linearization and extraction of the transgene.

Sup. Fig. S2

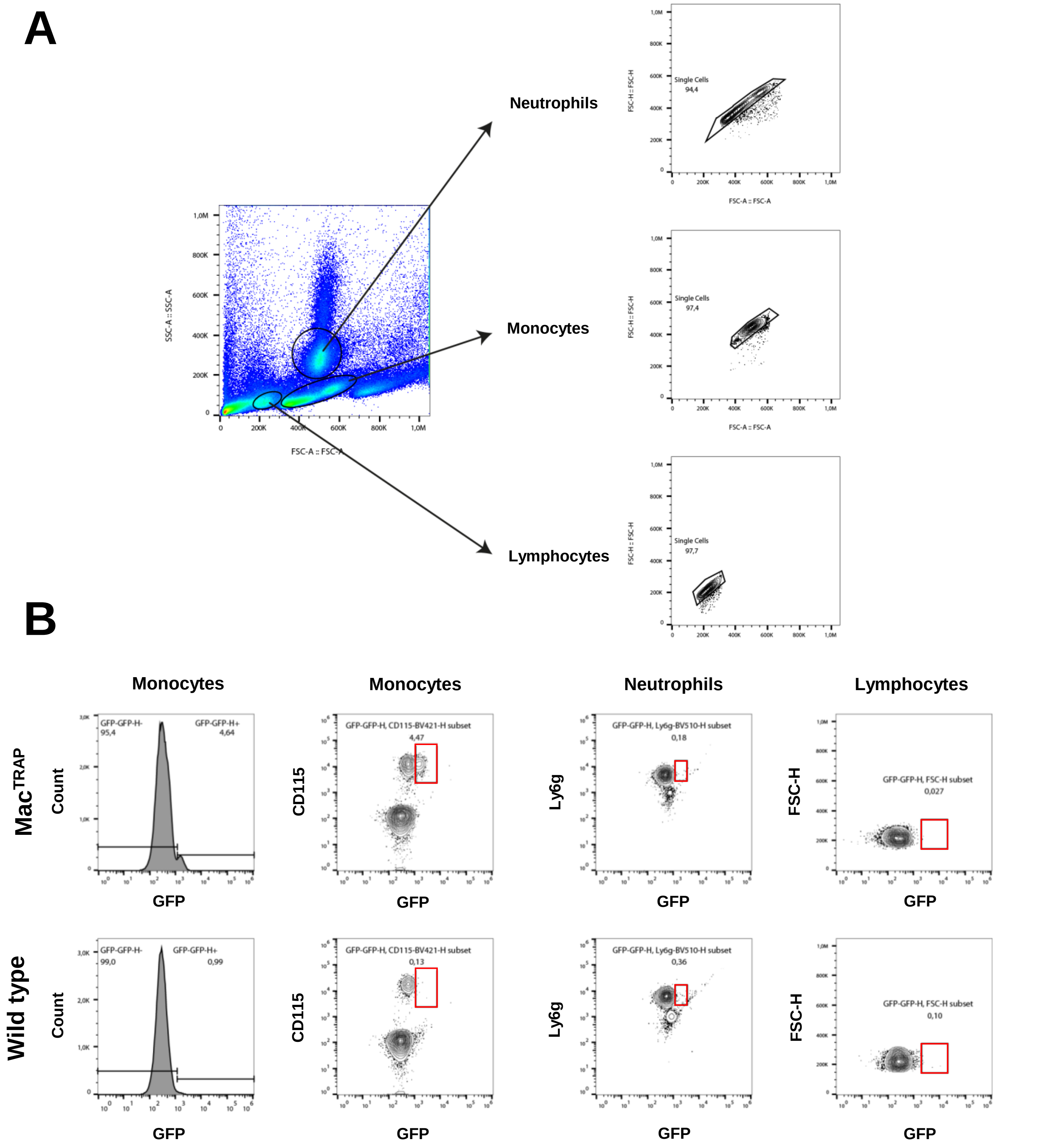

Sup. Fig. S2: FACS analysis detects eGFP-L10a signals in monocytes but not in neutrophils or lymphocytes isolated from peripheral blood of Mac^{TRAP} mice. **(A)** Gating strategy to define monocyte, neutrophil and lymphocyte populations. Only single cells contributed to the analysis. **(B)** GFP-fluorescence was specifically detected in $CD115^+$ monocytes, but not in $Ly6g^+$ neutrophils or lymphocytes of Mac^{TRAP} mice. Blood samples from wild-type mice served as negative controls. Representative plots are shown, n=6.

Sup. Fig. S3

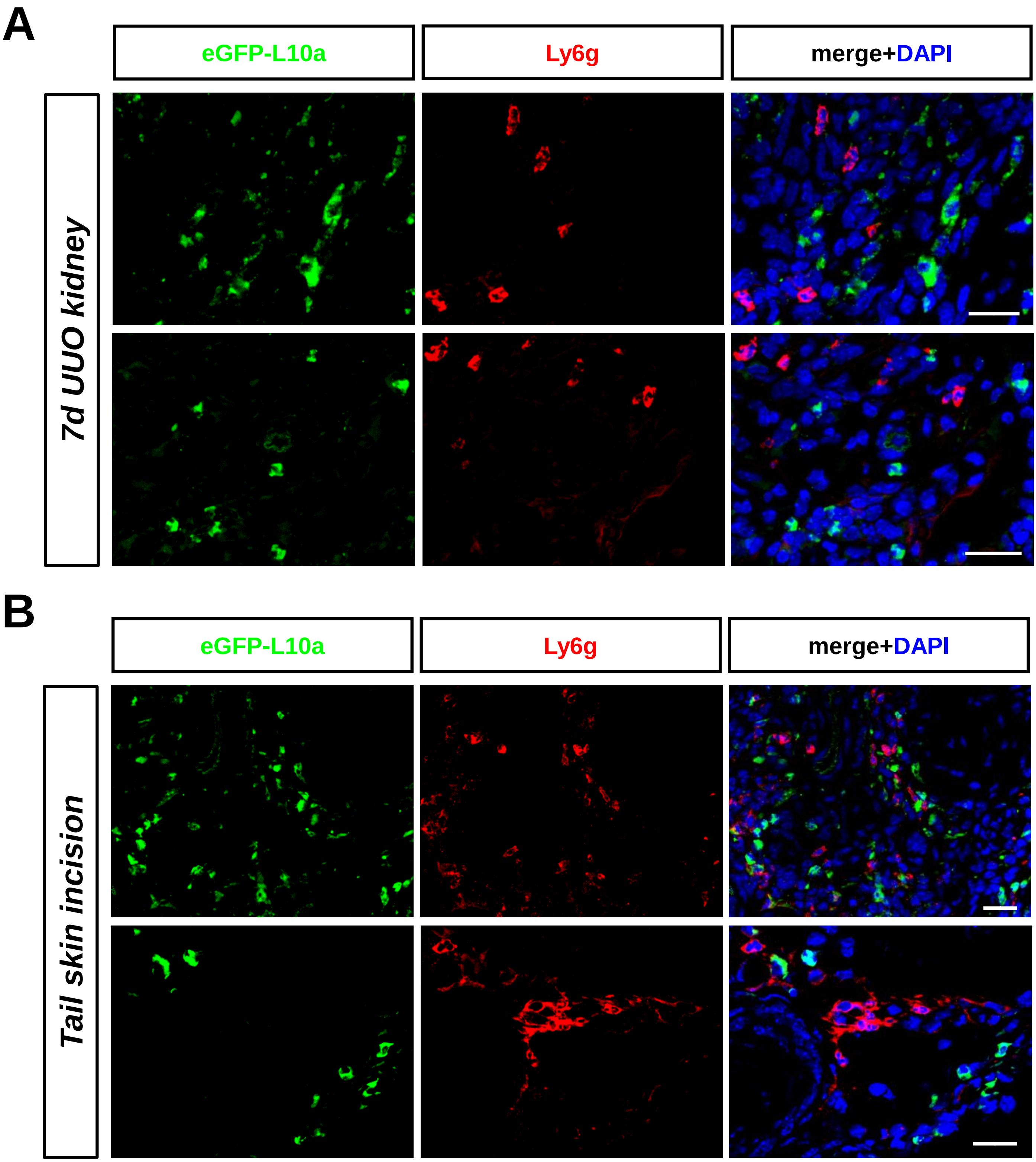

Sup. Fig. S3: Immunostaining for mature neutrophils in fibrotic kidney tissue and tail skin biopsies from Mac^{TRAP} mice. (A) Only a very small fraction of Ly6g^{+} neutrophils is positive for eGFP-L10a in fibrotic kidneys after 7d UUO (0.42% ± 0.29%; 468 cells counted, n=3). (B) Immunostaining of tail skin biopsies shows that only a very small fraction of Ly6g^{+} neutrophils is double-positive for eGFP-L10a (0.98% ± 0.52%; 517 cells counted, n=3). Scale bar: 20µm.

Sup. Fig. S4

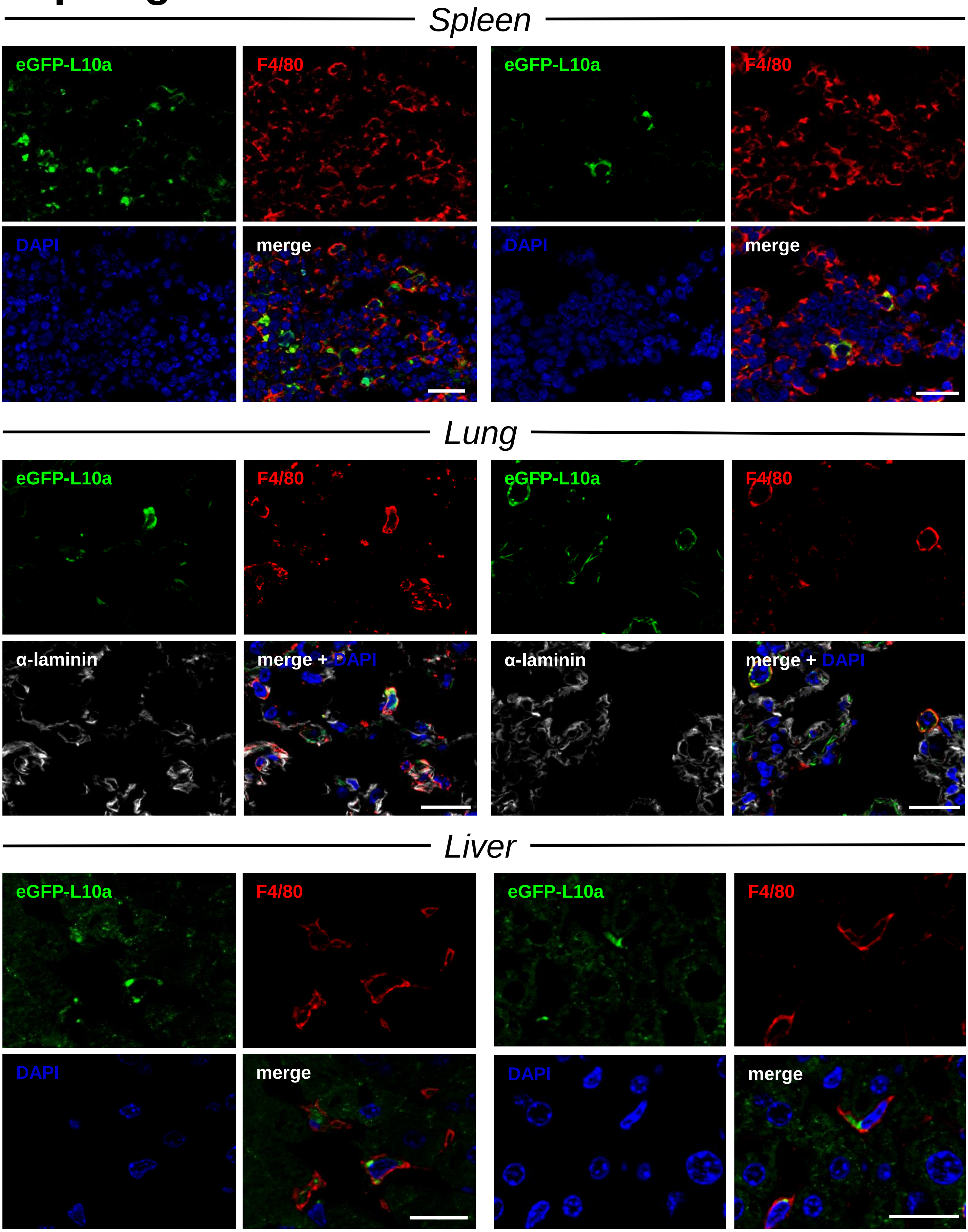
Spleen
eGFP-L10a
F4/80
eGFP-L10a
F4/80
DAPI
merge
DAPI
merge
Lung
eGFP-L10a
F4/80
eGFP-L10a
F4/80
α-laminin
merge + DAPI
α-laminin
merge + DAPI
Liver
eGFP-L10a
F4/80
eGFP-L10a
F4/80
DAPI
merge
DAPI
merge

Sup. Fig. S5

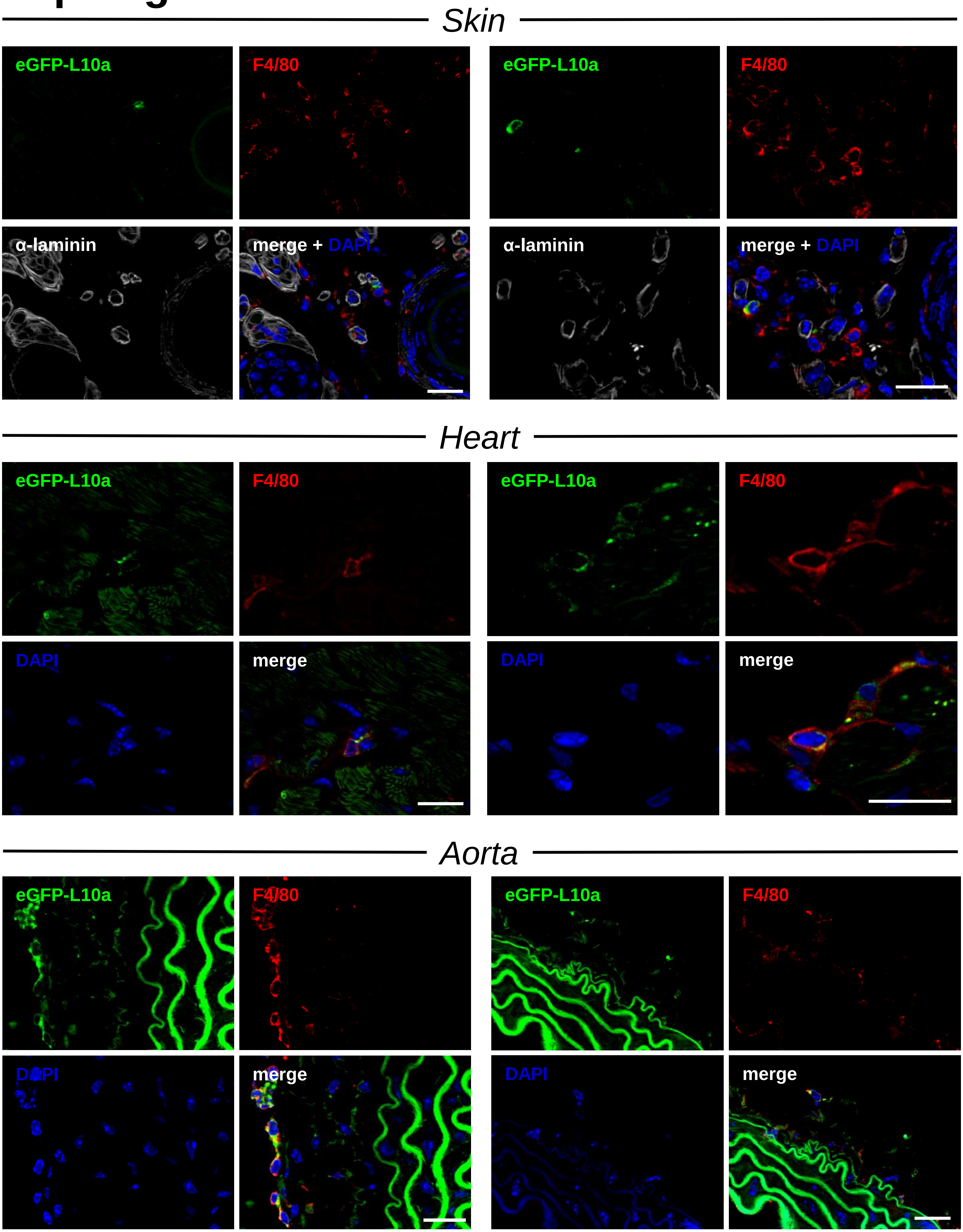
Skin
eGFP-L10a
F4/80
α-laminin
merge + DAPI
eGFP-L10a
F4/80
α-laminin
merge + DAPI
Heart
eGFP-L10a
F4/80
DAPI
merge
eGFP-L10a
F4/80
DAPI
merge
Aorta
eGFP-L10a
F4/80
DAPI
merge
eGFP-L10a
F4/80
DAPI
merge

Sup. Fig. S4+S5: Detection of eGFP-L10a$^+$ cells in various other organs and tissues of Mac^{TRAP} mice by fluorescence microscopy. EGFP-L10a expressing cells were also identified in other organs including spleen, lung, liver, skin, heart and aorta. Immunostaining confirmed macrophage-specificity (costaining with macrophage surface marker F4/80, *red*) and interstitial localization (highlighted by anti-laminin staining, *white*). Scale bar: 20µm.

Sup. Fig. S6

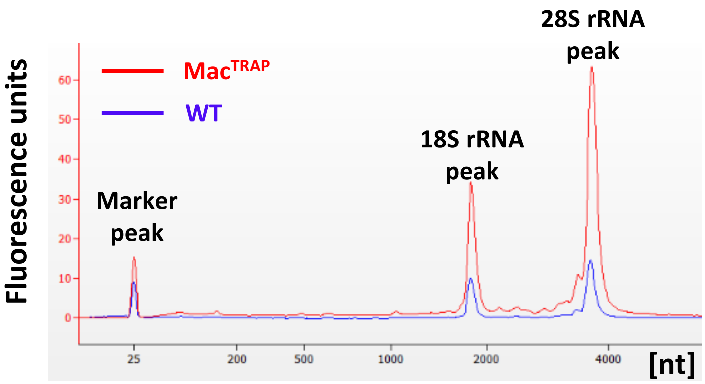

Sup. Fig. S6: Quality control of TRAP extracted RNA.
Representative Agilent 2100 Bioanalyzer® run of bound TRAP-RNA from a Mac^{TRAP} mouse kidney (red) and WT control (blue). Only TRAP-RNA samples with an RNA integrity number (RIN) >9 indicating excellent quality were used for downstream analyses including RNA-Seq; nt=nucleotides.

Sup. Fig. S7

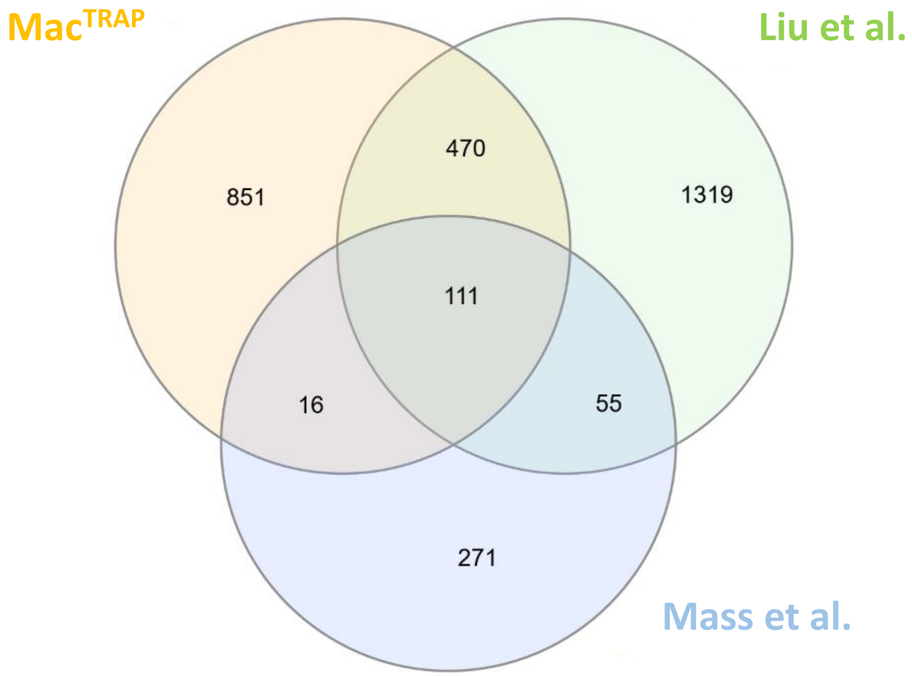

Sup. Fig. S7: Venn diagram between Mac^{TRAP}-generated translational profile of adult kidney macrophages and two published kidney macrophage-enriched expression profiles. We cross-referenced the Mac^{TRAP} dataset (≥ 2 fold enriched; $p < 0.05$) with data from Liu et al, 2014 (microarray analysis, ≥ 2 fold enriched, $p < 0.05$; kidneys from 7 week old Lyz2-L10a mice with Cre-mediated, irreversible recombination) and data published by Mass et al, 2016 (bulk RNA-Seq, top 453 enriched genes; FACS-based approach extracting CD45^{+}, CD11b^{+} and F4/80^{+} macrophages from p21 mouse kidneys. 111 genes were co-enriched among all datasets, featuring classical macrophage marker genes such as Csf1r, CD68, CD86, Cd14 and Lyz2 (see also Sup. Table 2).

Sup. Fig. S8

A

Cluster 1: Immunity

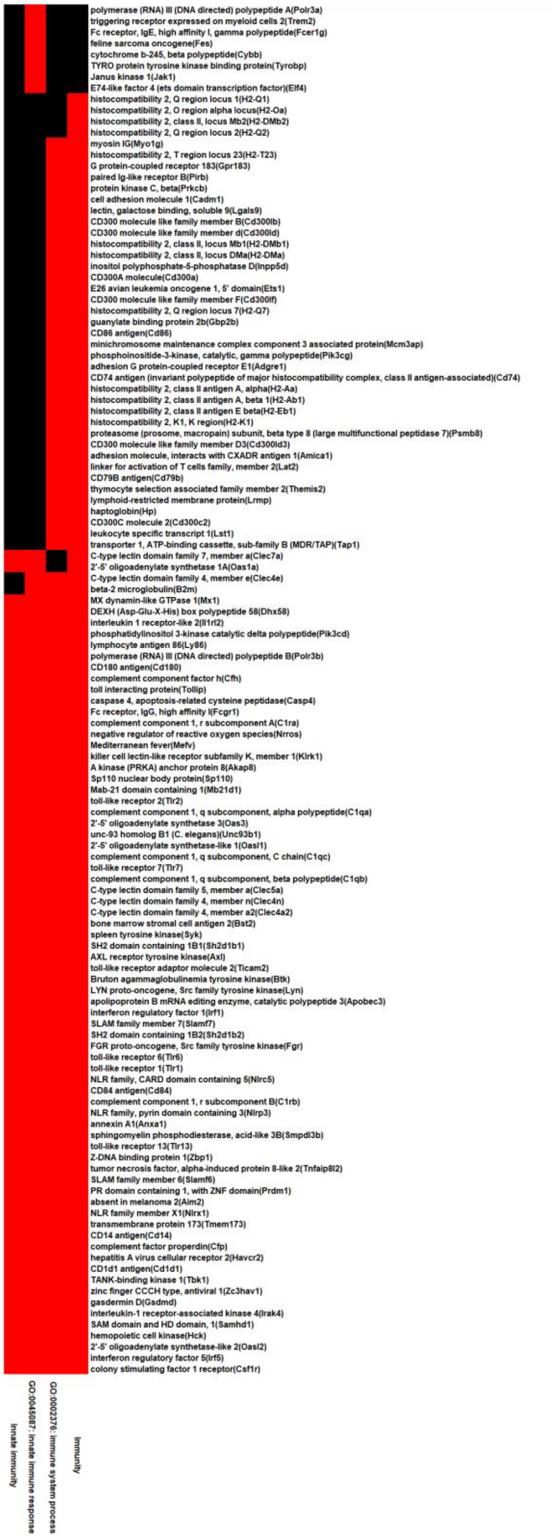

B

Cluster 2: Actin binding

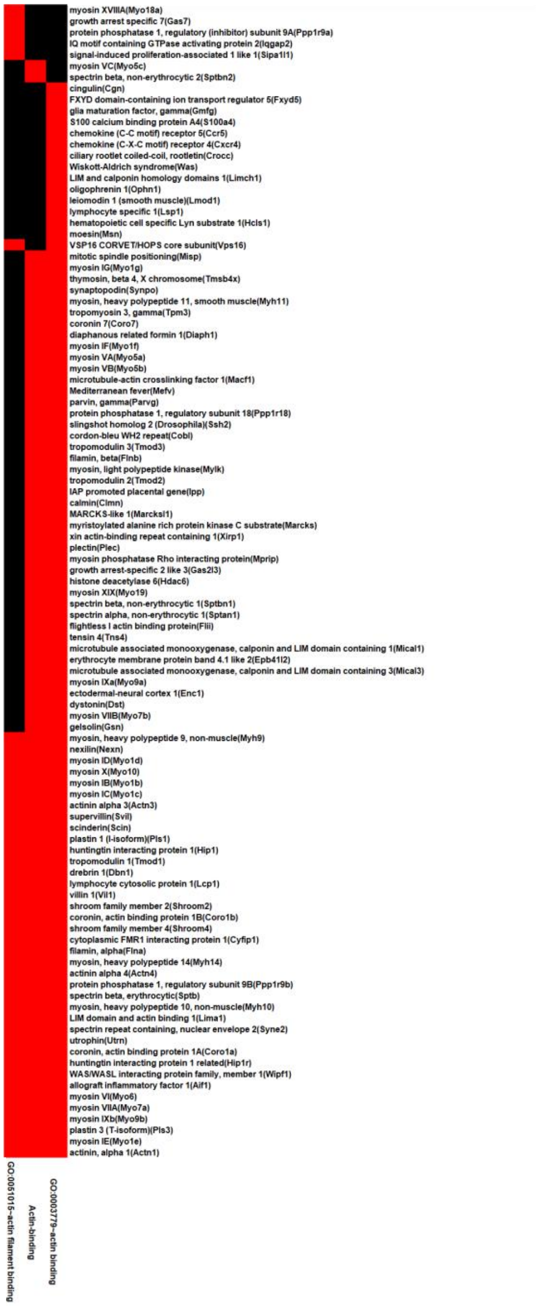

Sup. Fig. S8: DAVID Functional annotation clustering of renal Macrophage translational profile derived from Mac^{TRAP} and RNA Seq. Examples of 2D functional annotation clusters. A) Annotation cluster 1: Immunity B) Annotation cluster 2: Actin binding. Full analysis is presented in Supplemental Table 8.

Sup. Fig. S9

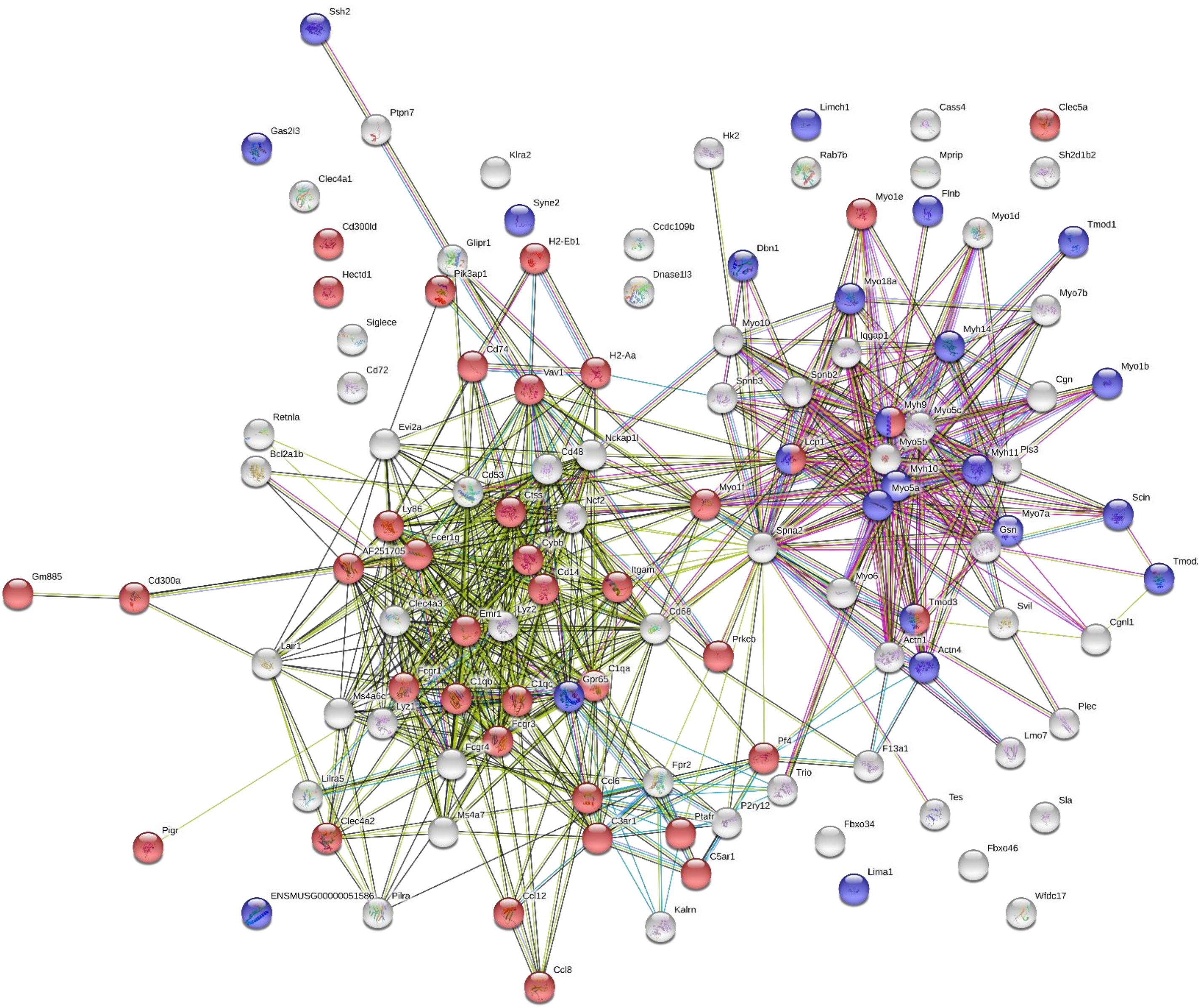

Sup. Fig. S9: Interaction network of genes with strongest enrichment in Mac^{TRAP} kidney macrophages (≥ 8 fold, $p < 0.05$) generated by StringDB. *Red nodes* represent genes enriched in immune system processes; *blue nodes* correspond to actin filament based processes.

Sup. Fig. S10

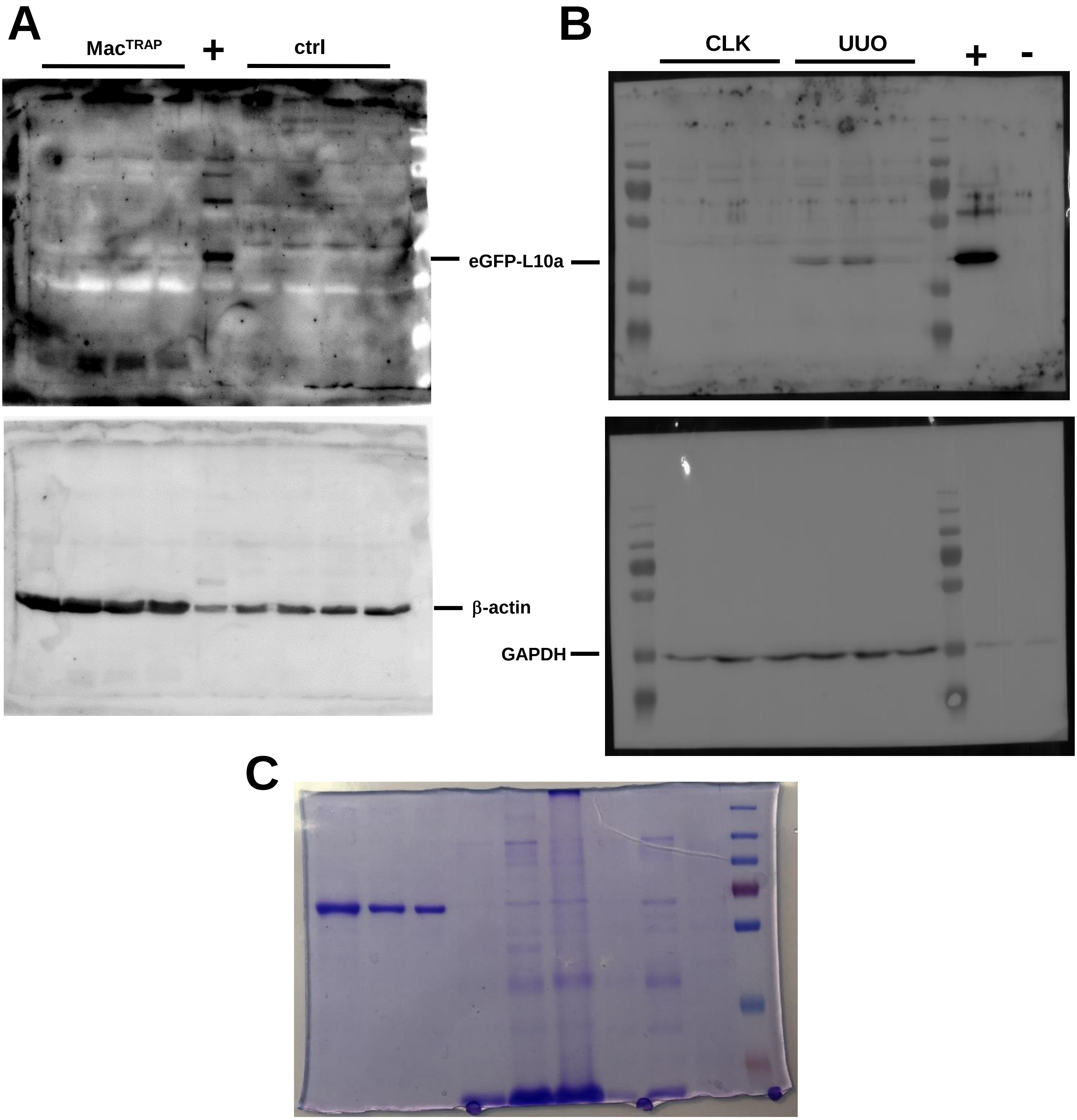

Sup. Fig. S10 (A) Full-length Western blots shown in Fig. 1H. Blots were loaded with spleen lysates from Mac^{TRAP} mice and lysates from WT controls. Upper blot was probed with an anti-GFP antibody (expected height of the eGFP-L10a band is indicated); lower blot was probed with an anti-β-actin antibody for loading control. **(B)** Full-length Western blots shown in Fig. 3D. Blots were loaded with protein lysates of 7d UUO and contralateral (CLK) controls kidneys from Mac^{TRAP} mice. Upper blot was probed with an anti-GFP antibody (expected height of the eGFP-L10a band is indicated); lower blot was probed with an anti-GAPDH antibody for loading control. Kidney lysates from Podo^{TRAP} (Grgic et al. 2014) and WT mice served as positive (+) and negative (-) controls for eGFP-L10a. **(C)** Full-length Coomassie gel depicted in Fig. 2C.

Supplementary Methods

FACS:

Peripheral blood from mice was obtained from the fascial vein and added to 1 ml HBSS prep (0.5% FCS [v/v], 20 mM HEPES in 1x PBS), supplemented with 80 µl heparin (25 I. E./ ml). For the following wash steps, the cells were centrifuged at 300g and 4 °C for 10 min. For erythrocyte lysis, the cell suspension was incubated with 5.0 ml NH_4Cl_2 at RT for 5 min. After washing with PBS containing 1% FCS, FcγR mouse blocking reagent (Miltenyi) was added according to manufacturers instructions to block unspecific antibody binding to surface FcγRs. For antibody staining, antibodies were diluted in PBS containing 1% FCS and incubated for 20 min at 4°C. Cells were washed with PBS containing 1% FCS for removal of unbound antibodies and subsequently sorted by flow cytometry (Attune NxT cytometer, Thermo Fisher). The data was analyzed using FlowJo software (BD). The following antibodies were used for FACS: rat anti-CD115-BV421 clone AFS98 (BioLegend, 1:300) and rat anti-Ly6g-BV510 clone 1A8 (Biolegend, 1:300).

Supplemental Tables

Development of a new macrophage-specific TRAP mouse (Mac^{TRAP}) and definition of the renal macrophage translational signature

Andreas Hofmeister, Maximilian C. Thomassen, Sabrina Markert, André Marquardt, Mathieu Preußner, Martin Rußwurm, Ralph Schermuly, Ulrich Steinhoff, Hermann-Josef Gröne, Joachim Hoyer, Benjamin D. Humphreys, Ivica Grgic

Correspondence: Ivica Grgic MD, Klinikum der Philips-Universität Marburg, Baldingerstrasse 1, 35043 Marburg. Phone: +4964215861736, email: grgic@med.uni-marburg.de

- Supplemental Table 1: Mac^{TRAP} Gene expression list (≥ 2x enriched, $p < 0.05$, 1448 Genes)
- Supplemental Table 2: Core macrophage transcripts (Venn diagramm)
- Supplemental Table 3: Genes exclusively identified in Mac^{TRAP} dataset
- Supplemental Table 4: DAVID functional classification chart
- Supplemental Table 5: DAVID functional annotation chart
- Supplemental Table 6: DAVID UP_TISSUE
- Supplemental Table 7: DAVID UP_TISSUE complete
- Supplemental Table 8: DAVID functional annotation clustering
- Supplemental Table 9: StringDB GO Biological Processes enrichment
- Supplemental Table 10: Primers used for RT-qPCR

Supplemental Tables

Development of a new macrophage-specific TRAP mouse (Mac^{TRAP}) and definition of the renal macrophage translational signature

Andreas Hofmeister, Maximilian C. Thomassen, Sabrina Markert, André Marquardt, Mathieu Preußner, Martin Rußwurm, Ralph Schermuly, Ulrich Steinhoff, Hermann-Josef Gröne, Joachim Hoyer, Benjamin D. Humphreys, Ivica Grgic

Correspondence: Ivica Grgic MD, Klinikum der Philips-Universität Marburg, Baldingerstrasse 1, 35043 Marburg. Phone: +4964215861736, email: grgic@med.uni-marburg.de

- Supplemental Table 1: Mac^{TRAP} Gene expression list (≥ 2x enriched, $p < 0.05$, 1448 Genes)
- Supplemental Table 2: Core macrophage transcripts (Venn diagramm)
- Supplemental Table 3: Genes exclusively identified in Mac^{TRAP} dataset
- Supplemental Table 4: DAVID functional classification chart
- Supplemental Table 5: DAVID functional annotation chart
- Supplemental Table 6: DAVID UP_TISSUE
- Supplemental Table 7: DAVID UP_TISSUE complete
- Supplemental Table 8: DAVID functional annotation clustering
- Supplemental Table 9: StringDB GO Biological Processes enrichment
- Supplemental Table 10: Primers used for RT-qPCR

Genes ≥ 2 fold enriched $p < 0.05$ in Mac^{TRAP} dataset
1448 Genes

external_gene_name	baseMean	log2FoldChange	fold change	stat	padj
Dbn1	5157.23463	6.055316763	66.5015823	19.7637668	1.3821E-82
Myh10	75200.1435	5.980047341	63.1209643	19.4296918	4.8991E-80
Myh9	148852.005	6.290649544	78.2842157	19.0511511	4.8486E-77
Tmod3	22247.4356	5.895186095	59.5151928	18.775043	6.8374E-75
Gsn	27175.7209	5.441014589	43.4418786	18.5999761	1.455E-73
Myh11	4382.93456	5.874074879	58.6506373	16.8272627	5.811E-60
Actn4	52596.2114	5.145304547	35.390851	16.800618	7.8082E-60
Lmo7	8615.91714	4.852990021	28.8998484	16.3444555	1.3477E-56
Myo1e	10004.7452	4.571404327	23.7755091	16.3154189	1.9281E-56
Pls3	12711.8538	5.324932592	40.0833891	16.2110656	9.5322E-56
Myh14	16222.1509	5.694693015	51.7932798	16.1928222	1.1659E-55
Lcp1	11300.5374	5.101589088	34.3345485	16.0622628	8.8472E-55
Sptan1	43873.4649	4.768767151	27.2610108	15.2937087	1.466E-49
Fbxo34	6351.8575	4.898944802	29.835226	15.271744	1.9071E-49
Myo6	104469.329	4.920486175	30.2840484	15.2100571	4.5757E-49
Gm7224	977.430089	4.838861122	28.6182018	15.0793781	3.1308E-48
Myo7a	9370.67606	4.504811479	22.7030068	14.9665292	1.6174E-47
Myo1d	11700.2108	4.160286072	17.8801393	14.8042324	1.7294E-46
Fbxo46	11073.8424	5.19436629	36.6150863	14.671817	1.1636E-45
Myo5b	39032.0959	4.498666686	22.6065148	14.6308736	2.0196E-45
Svil	1702.45855	3.964734242	15.6136317	14.2037106	9.3635E-43
Actn1	2654.00264	4.397072204	21.0693252	14.0848749	4.8399E-42
Lima1	8185.76447	3.940436263	15.3528678	13.9673479	2.4268E-41
Ptafr	384.767312	4.576293496	23.8562189	13.7344301	5.9549E-40
Pigr	13404.2588	3.5159375	11.4393843	13.7092779	8.0876E-40
Kalrn	1914.84826	3.175442607	9.03448652	13.4710285	2.016E-38
Iqgap1	15905.0079	4.306338815	19.7850501	13.3058401	1.7943E-37
Myo1b	1373.52174	4.436146078	21.6477635	13.0113465	8.5244E-36
Ms4a7	455.347035	4.660256692	25.2858206	12.8395983	7.6777E-35
Sptbn2	887.305064	4.283315224	19.4718118	12.8282479	8.5931E-35
Myo10	12861.4859	4.300630876	19.7069264	12.7963015	1.2553E-34
Cgnl1	49156.4098	4.124246719	17.4390158	12.6248212	1.0898E-33
Sptbn1	69674.4282	4.124811997	17.4458501	12.43903	1.1003E-32
H2-Aa	14151.6513	3.634694795	12.4208741	12.4345768	1.1291E-32
Tmod1	605.268829	4.501141807	22.6453323	12.061611	1.0892E-30
Hectd1	16033.9517	3.550082968	11.7133592	11.9523692	3.9667E-30
Ssh2	3885.86931	3.731844434	13.2860877	11.7101365	6.9176E-29
Gm43587	1104.08854	3.887340427	14.7981039	11.6940933	8.1376E-29
Cybb	1431.87622	3.556753983	11.7676471	11.6623917	1.1512E-28
Myo18a	19599.6452	3.417681502	10.6862332	11.5476656	4.2916E-28
Bcl2a1a	482.753177	3.989838816	15.8877048	11.5014007	7.1642E-28
Ctss	3468.24471	3.833934415	14.2603196	11.3318558	4.9169E-27
Myo5a	8649.83511	3.765090106	13.5958091	11.30893	6.2381E-27
Gas2l3	820.953014	4.054231144	16.6128898	11.2343873	1.4217E-26

Nckap1l	715.864421	3.72682827	13.2399729	11.2167346	1.6973E-26
Limch1	8919.09146	4.293271677	19.6066571	11.2013252	1.9762E-26
Vav1	299.14954	3.964905971	15.6154904	11.0737283	8.1015E-26
Trio	2627.1721	3.081873873	8.46713489	11.0552654	9.7466E-26
Cd68	765.842689	3.790291713	13.8353929	10.9542597	2.9279E-25
Lyz1	1207.87247	3.734233696	13.3081091	10.922678	4.0649E-25
Bcl2a1d	540.973026	3.737978151	13.3426946	10.9059233	4.6999E-25
Myo7b	9974.99523	3.388590594	10.472911	10.907203	4.6999E-25
Lyz2	8316.55564	3.688028505	12.8886433	10.7782927	1.8613E-24
Cgn	6183.25217	3.843631682	14.3564951	10.7295154	3.0442E-24
Sla	353.006918	3.060192495	8.34083891	10.7303688	3.0442E-24
C1qb	7664.31335	3.662026321	12.6584278	10.7247992	3.1464E-24
Flnb	7424.83307	3.280000163	9.71356017	10.686128	4.6931E-24
Dnase1l3	297.80564	3.422431401	10.7214743	10.6542173	6.5021E-24
C5ar1	270.761159	3.884245287	14.7663903	10.6169643	9.5323E-24
Fcer1g	2495.6439	3.646192118	12.5202557	10.5829346	1.3487E-23
Cd14	663.811914	3.532660305	11.5727539	10.5483449	1.9181E-23
Myo5c	904.263037	3.19405195	9.15177732	10.5393337	2.077E-23
Ctsc	1337.24625	2.915337949	7.54404326	10.5286186	2.2905E-23
Lilra5	674.963386	3.588304597	12.027831	10.4871823	3.4984E-23
Fcgr4	365.618016	3.661655424	12.6551739	10.4219355	6.8556E-23
Klra2	127.341933	4.410939021	21.2728146	10.3982741	8.6569E-23
Cd53	758.275409	3.269501538	9.64313027	10.3854738	9.7527E-23
Clec4a3	197.901838	4.25505931	19.0941568	10.3781141	1.0379E-22
C1qc	2333.45844	3.767607778	13.6195561	10.3617679	1.2137E-22
Plec	5004.12706	3.218131997	9.30581172	10.3474088	1.3901E-22
Syne2	13178.929	3.040819331	8.229583	10.3416286	1.4557E-22
Fcgr3	1538.65656	3.690512662	12.9108552	10.3098066	2E-22
Tes	5546.7393	3.453259807	10.9530428	10.268927	3.0158E-22
Mical3	4056.61177	3.334956264	10.0907134	10.249876	3.6239E-22
Adgre1	587.119493	3.664127863	12.6768805	10.2327101	4.2698E-22
Cd300c2	399.812953	3.762315809	13.5696895	10.1759957	7.5573E-22
Myo1c	10902.9851	2.709438639	6.54067096	10.151777	9.5633E-22
Rab7b	713.280784	3.437684748	10.8354318	10.0716834	2.1385E-21
Scin	1715.50732	3.161248122	8.94603326	10.0402817	2.9043E-21
BC021767	303.315107	2.952543104	7.74112424	10.0191712	3.5517E-21
Lair1	381.715607	3.55297655	11.736876	9.97150671	5.6752E-21
H2-Ea-ps	6617.5485	3.2557549	9.55168262	9.91643421	9.7464E-21
Myo1f	615.70499	3.152972699	8.89486493	9.82359553	2.4296E-20
Prkcb	462.768341	3.622638324	12.3175065	9.75248412	4.8497E-20
Cd300a	195.331202	3.891344297	14.8392297	9.69735895	8.2378E-20
Cd72	768.147925	3.22819615	9.37095545	9.66903165	1.0742E-19
Bcl2a1b	239.239778	3.424556767	10.7372807	9.62639492	1.6092E-19
Ppp1r9a	951.131678	2.985285071	7.91881782	9.57598005	2.5947E-19
Evi2a	436.908861	3.734698775	13.3123999	9.54473619	3.4698E-19
Laptm5	5822.74744	2.872273709	7.32218238	9.41480031	1.1919E-18
Ncf2	523.527219	3.139162715	8.81012639	9.28826865	3.9E-18
H2-Eb1	11199.339	3.215217472	9.28703114	9.24906678	5.5707E-18
Lmod1	371.648004	2.770947478	6.82556029	9.21997443	7.2315E-18
Evpl	1290.71775	2.954349151	7.75082109	9.19746225	8.8238E-18

Sh2d1b1	156.581193	4.137410557	17.5988659	9.14954726	1.362E-17
Clec7a	336.236446	3.447911143	10.9125105	9.08223551	2.5075E-17
Cyth4	1376.57714	2.799555595	6.96225954	9.0745446	2.6633E-17
Fcgr1	313.945274	3.557812015	11.7762803	9.05381952	3.188E-17
Shroom2	1087.52354	2.897208641	7.44983587	9.03682625	3.6869E-17
Ly86	654.878231	3.345486631	10.1646359	9.0350863	3.7086E-17
Igsf6	270.013581	2.965148005	7.80905517	8.97761913	6.1998E-17
Cd74	75832.3944	3.246535967	9.49084123	8.90010424	1.2379E-16
Mprip	13983.0709	3.039169853	8.22017925	8.88770701	1.3707E-16
C3ar1	393.273903	3.124562086	8.72141417	8.84120822	2.0605E-16
Plekhg3	6344.33305	2.370253185	5.17031861	8.81656723	2.5438E-16
Tollip	6523.92471	2.333783403	5.04125665	8.80254716	2.8555E-16
Clec4a1	206.993864	3.529592538	11.5481715	8.7880407	3.2189E-16
Iqgap2	2836.43618	2.838846717	7.15447903	8.78229498	3.3564E-16
Pf4	555.214618	3.717334761	13.1531347	8.7099169	6.3138E-16
Cd48	224.539666	3.62787532	12.3623004	8.70649409	6.4482E-16
Dctn4	7336.51668	2.368389186	5.16364273	8.60637087	1.5375E-15
Tifab	475.698413	2.728850827	6.62927374	8.59994172	1.6116E-15
Myo15b	7081.44642	2.381996053	5.21257434	8.59753844	1.6311E-15
Ifi204	420.585639	2.657428385	6.30907449	8.59445027	1.6609E-15
Gpr65	195.940328	3.489132845	11.2288077	8.56052846	2.211E-15
Lmtk2	4994.18436	2.247972684	4.75014872	8.55058286	2.3893E-15
Pld4	1012.97324	2.768629115	6.81460065	8.54197134	2.5523E-15
Ccl6	381.690436	3.164082209	8.96362447	8.54037583	2.5659E-15
Rac2	997.839902	2.704790013	6.51962965	8.50785999	3.3694E-15
Utrn	1763.59106	2.624488322	6.1666558	8.45496708	5.2213E-15
Lrch3	1173.0118	2.238577377	4.7193147	8.4557997	5.2213E-15
Hcls1	876.927021	2.781468743	6.87551959	8.45291867	5.2274E-15
Copa	13268.1521	2.57254809	5.94859142	8.45336173	5.2274E-15
Atg7	3845.64375	2.834859819	7.13473487	8.44772809	5.421E-15
Siglece	284.695031	3.10084192	8.57919284	8.42406386	6.5837E-15
C1qa	2888.98569	3.265196746	9.61439946	8.39396102	8.4423E-15
Cd83	405.724153	2.723568046	6.60504342	8.38516276	9.0267E-15
Fpr2	80.0899412	4.225430831	18.7060213	8.32735399	1.4616E-14
Axl	1004.20934	2.443370705	5.43911038	8.21330883	3.7749E-14
Mylk	4644.08825	2.589785028	6.02008989	8.20919823	3.8763E-14
Coro1a	2063.02051	2.842045683	7.17036064	8.2068588	3.9006E-14
Vil1	10978.9603	2.29753742	4.91617891	8.20661653	3.9006E-14
Tmem173	184.416001	2.63576537	6.21504729	8.13976714	6.7389E-14
Hip1r	2772.35782	2.616726553	6.13356798	8.11056316	8.5095E-14
Cd300ld	193.927157	3.123926849	8.71757487	8.07821146	1.1018E-13
Milr1	156.162846	3.480919868	11.1650659	8.05808132	1.2897E-13
Ms4a6c	134.024227	3.258728523	9.57139045	8.03526279	1.5425E-13
Gas2	17899.6477	1.812352018	3.51214405	8.02878243	1.6144E-13
Nr6a1	409.51397	2.651557882	6.28345426	7.97892722	2.4033E-13
Mcub	111.571908	3.331062272	10.0635141	7.97526444	2.458E-13
Pls1	6090.46676	2.447310685	5.45398482	7.96348463	2.6846E-13
Clec5a	138.779697	3.277365215	9.69583545	7.95339472	2.8921E-13
Ifi207	365.770592	2.723210113	6.60340491	7.9470625	3.0225E-13
Gm9025	137.015355	3.061629027	8.34914825	7.93384236	3.3391E-13

Pltp	307.407569	2.647342069	6.26511968	7.92149804	3.6626E-13
Myo1g	204.22984	2.321057686	4.9969843	7.90460371	4.1664E-13
Bin2	198.257181	2.7347981	6.65665824	7.87613966	5.1982E-13
Pou2f2	254.01866	2.850094174	7.21047436	7.86259335	5.7535E-13
Glipr1	112.265696	3.238917844	9.44085711	7.85881267	5.89E-13
Vsir	478.827748	2.934956562	7.64733229	7.85094767	6.2296E-13
Clec4a2	339.558017	3.137796135	8.80178502	7.8320519	7.1935E-13
P2ry6	522.887285	2.327253304	5.01848987	7.80808358	8.6451E-13
Ccl12	372.25539	3.195910099	9.16357214	7.79366824	9.628E-13
Cd52	2186.01112	2.75029053	6.72852618	7.76972175	1.1559E-12
Birc6	7956.10368	1.85586233	3.61968043	7.76174428	1.2231E-12
Il10ra	476.253228	2.88036152	7.36334613	7.7344661	1.5064E-12
Il1b	235.552291	2.969024001	7.83006346	7.70267197	1.9208E-12
Slamf7	152.553305	2.652806132	6.28889319	7.68756979	2.1479E-12
Parvg	314.858079	2.791678536	6.92434945	7.65026216	2.8548E-12
Tyrobp	1956.98143	2.946472505	7.70861943	7.64217179	3.0211E-12
Cd79b	153.791368	2.957217753	7.76624787	7.62498436	3.4307E-12
H2-DMb2	897.169217	2.63831853	6.2260559	7.60766998	3.8987E-12
Ighm	2284.73687	2.623864415	6.16398955	7.59463745	4.2854E-12
Dync2h1	1360.8813	2.164741125	4.48385966	7.58778164	4.4907E-12
Ptpn7	111.821413	3.142143146	8.82834583	7.58485182	4.5655E-12
Prpf8	7501.24021	2.180242609	4.53229764	7.5562377	5.6563E-12
C1ra	791.883292	2.491283807	5.6227808	7.53176763	6.7836E-12
Pilra	94.0763692	3.5163969	11.4430275	7.51827632	7.4767E-12
Arhgap30	361.226282	2.443822944	5.44081564	7.46636029	1.104E-11
Copb2	6792.19015	2.133533882	4.38790984	7.46346192	1.1219E-11
H2-Ab1	12823.3312	2.742582941	6.69267492	7.45874923	1.156E-11
Cd33	168.264393	2.777703886	6.85760062	7.44960449	1.2318E-11
H2-DMa	1341.04591	2.535510906	5.79782146	7.44740712	1.2453E-11
Fermt3	511.723924	2.431548336	5.39472095	7.44095323	1.3001E-11
Msn	3411.50946	1.63379859	3.10329015	7.40766746	1.6623E-11
Macf1	4271.11715	2.020405109	4.05697696	7.40113041	1.7363E-11
Pik3ap1	107.437813	3.135231864	8.78615447	7.34573419	2.6164E-11
Cd209a	109.246353	2.980745231	7.89393823	7.34225119	2.6554E-11
Phf11b	635.297685	2.501047225	5.66096194	7.34263509	2.6554E-11
Ift172	1582.95968	2.0574332	4.16245074	7.33721692	2.7419E-11
Sash3	203.46076	2.925815717	7.59903234	7.31694448	3.1719E-11
Runx1	137.415401	2.698315407	6.49043605	7.31480379	3.2051E-11
Itgam	63.151438	3.150751436	8.88118039	7.31162796	3.2639E-11
Sytl2	452.002691	2.041385436	4.11640644	7.28588601	3.9305E-11
Nfkbia	3456.74271	2.169800912	4.49961296	7.26853849	4.4454E-11
Unc93b1	1174.11944	1.906236954	3.74830137	7.25201069	4.9962E-11
Lpcat2	154.484229	2.971874408	7.84554902	7.24847535	5.0738E-11
Runx3	213.713669	2.564637158	5.91606194	7.24850129	5.0738E-11
Slamf9	1045.23358	2.768228703	6.81270956	7.24312512	5.2502E-11
Ppfia4	533.828007	2.541758	5.82298136	7.23597298	5.5053E-11
Slc11a1	305.647118	2.606629475	6.09079047	7.23413308	5.5513E-11
Mpeg1	736.330505	2.62133843	6.15320658	7.21912413	6.1354E-11
Myof	803.988641	1.860153149	3.63046199	7.21915711	6.1354E-11
H2-DMb1	1138.59397	2.671808904	6.37227666	7.1914103	7.4805E-11

Lilr4b	437.467724	2.611825679	6.11276743	7.18177308	7.9862E-11
Plek	431.663953	2.689314615	6.45006909	7.15856468	9.3656E-11
Ms4a6b	262.233963	2.474622968	5.55822011	7.15870491	9.3656E-11
Themis2	393.195587	2.700925684	6.50218987	7.15430474	9.6122E-11
Hip1	884.098426	2.464288878	5.51854858	7.14254552	1.0419E-10
Cd86	273.924493	2.78981699	6.91542055	7.13365656	1.1059E-10
Cep295	603.092418	1.957093385	3.8827892	7.06145865	1.8551E-10
Aass	19836.8377	1.818536941	3.52723315	7.05746919	1.8997E-10
Mrc1	319.4657	2.765332757	6.79904801	7.05570303	1.9145E-10
Cyp4f18	111.915897	2.950720234	7.73134938	7.03399045	2.2266E-10
Slfn2	800.3278	2.516749195	5.72291109	7.00764868	2.6755E-10
Ccr5	175.833369	2.558011147	5.88895295	6.99965745	2.8189E-10
Hk2	125.749963	3.097072518	8.55680681	6.9915681	2.9719E-10
Ddb1	11661.4625	1.748349023	3.35973868	6.98186253	3.1693E-10
Wfdc17	157.052299	3.214581347	9.28293713	6.97678148	3.2703E-10
Misp	746.029185	2.22878974	4.68740594	6.93862107	4.267E-10
Lat2	140.863763	2.887559793	7.40017708	6.93206912	4.4482E-10
Sdha	36714.3921	1.820062426	3.53096477	6.92404374	4.6856E-10
Neurl3	454.938566	2.052296797	4.14765759	6.91929171	4.8227E-10
Myom2	267.663833	2.518346793	5.72925199	6.89641853	5.6398E-10
Mical1	155.301865	2.671121029	6.36923909	6.89190017	5.7888E-10
Hck	393.133702	2.665693257	6.34532147	6.89138777	5.7888E-10
Dnmt1	1028.99386	1.865711574	3.64447644	6.87847965	6.309E-10
Fam111a	282.599277	2.310742614	4.96138397	6.86832553	6.7436E-10
Rassf4	600.126117	1.989186711	3.97013127	6.83879437	8.2153E-10
Hdac6	4192.42226	1.779107633	3.43213817	6.83903228	8.2153E-10
Plxna4os1	67.2594656	3.169290116	8.99604025	6.83353701	8.4837E-10
Snrnp200	5739.40562	1.972605168	3.92476199	6.81901921	9.3444E-10
Clec4b1	155.54214	2.891595844	7.42090863	6.79228653	1.1201E-09
Sec23b	3995.17948	1.583454276	2.99686537	6.78487593	1.1738E-09
C1rb	383.532355	2.424592006	5.36877151	6.76032725	1.3848E-09
Trf	202.48755	2.675604379	6.38906306	6.75406021	1.4396E-09
Cx3cr1	421.437564	2.196360018	4.58321517	6.74891791	1.485E-09
Eftud2	2343.1067	1.69821496	3.24499209	6.73992458	1.5729E-09
Ms4a4c	83.4714578	2.833384796	7.12744399	6.7347389	1.6229E-09
Rgs10	793.309925	2.573132923	5.95100332	6.73411103	1.6229E-09
Lcp2	232.158912	2.330155823	5.0285966	6.72924721	1.6708E-09
Ramp1	160.778633	2.763244011	6.78921141	6.72253722	1.7421E-09
Ighv3-6	159.06116	3.247121038	9.49469092	6.71273834	1.8552E-09
Pid1	200.692069	2.324837395	5.01009304	6.7066039	1.9266E-09
Ube2l6	303.377441	2.306320431	4.9461995	6.68929798	2.1595E-09
Epsti1	353.586553	2.23533107	4.70870738	6.68254131	2.2519E-09
Oas1g	473.33208	2.334774491	5.04472103	6.66579857	2.5136E-09
Crocc	886.832577	2.455285195	5.48421524	6.66309932	2.5494E-09
Tnfaip8l2	561.184736	2.714433322	6.56335433	6.65870818	2.6158E-09
Sec23ip	2302.91627	1.664676857	3.17042634	6.6413553	2.9308E-09
Alox5ap	437.281972	2.414976752	5.33310877	6.63798823	2.9861E-09
Selplg	278.710151	2.324739059	5.00975156	6.63170725	3.1031E-09
Zfp185	392.019583	2.187077515	4.55382078	6.59282183	4.019E-09
Ndufs1	14580.6008	1.844498457	3.59128079	6.58928407	4.0991E-09

Traf3ip3	58.6301167	2.907439865	7.50285598	6.57362239	4.536E-09
Fxyd5	1324.26856	2.147357845	4.43015705	6.57149953	4.5824E-09
Flna	1640.13171	1.995284886	3.98694827	6.55475607	5.1064E-09
Btk	99.0172038	2.556076449	5.88106098	6.53567524	5.7785E-09
Psd4	144.153919	2.36600794	5.15512689	6.5323707	5.8838E-09
1110037F02Rik	1000.74206	1.634074029	3.10388268	6.52553072	6.134E-09
Kdm5b	1420.07512	1.827371454	3.54889885	6.51777201	6.434E-09
Itgb2	347.755257	2.00272045	4.0075498	6.51500588	6.5277E-09
Casp1	138.672675	2.748221234	6.71888219	6.50775778	6.8234E-09
Csf2rb	226.065293	2.163777569	4.48086596	6.49651652	7.324E-09
Oas1a	670.901487	2.253363795	4.76793245	6.4855604	7.8456E-09
Fgd2	607.424313	1.798145026	3.47772782	6.48453524	7.8683E-09
Plcg2	939.806038	1.910088816	3.75832236	6.48291209	7.9225E-09
Gas7	917.709632	1.826282577	3.54622132	6.4585338	9.2736E-09
BC028528	156.438739	2.485829013	5.60156135	6.45619227	9.3818E-09
Pik3cg	178.624374	2.712362535	6.55394031	6.45364085	9.5045E-09
Ncf1	311.033513	2.42589332	5.37361633	6.44595473	9.9607E-09
Rassf2	392.320156	1.979070339	3.94238956	6.43786758	1.0466E-08
Pwp1	1113.67881	1.614503419	3.06206184	6.42424671	1.1404E-08
Rasa3	342.792506	2.030020915	4.08410771	6.42275765	1.1472E-08
Basp1	136.569862	2.757744997	6.76338274	6.4202412	1.1619E-08
Cad	503.923608	1.893619252	3.71566195	6.39156707	1.3969E-08
Pcx	34718.2544	1.740816532	3.34224277	6.38733871	1.4307E-08
Hpgd	887.464525	2.018115184	4.05054261	6.37918965	1.5033E-08
Alox15	1635.10433	1.657985642	3.15575596	6.37122084	1.5777E-08
Ston2	1150.71835	1.733614011	3.32559852	6.36560014	1.6305E-08
Smc1a	3803.90797	1.89049327	3.7076197	6.3449632	1.8579E-08
Tgfbi	778.751479	2.030117872	4.0843822	6.34399225	1.8628E-08
Lyn	1088.80756	1.504224986	2.83672242	6.34233312	1.8761E-08
Cep350	1840.78603	1.751578338	3.36726751	6.33275467	1.9891E-08
Mgl2	89.296599	2.903489327	7.48233898	6.32606958	2.0696E-08
Nrros	258.765095	2.178171314	4.52579524	6.32450388	2.0831E-08
Ncf4	348.799561	2.601618147	6.06967028	6.31637316	2.1877E-08
Tmod2	65.9572623	3.359874016	10.2665106	6.31567747	2.1897E-08
Ms4a6d	76.4498354	2.8981576	7.45473775	6.31456871	2.1975E-08
Zmynd15	175.329061	2.092496898	4.26485561	6.30532427	2.3245E-08
Wdr6	1193.26818	1.613476882	3.05988383	6.28837917	2.5836E-08
Trem2	114.647789	2.826638297	7.09419161	6.25914251	3.1065E-08
Gpr183	108.885584	2.540317704	5.81717096	6.25067938	3.2681E-08
Cttn	11425.6265	2.004470726	4.01241471	6.24004349	3.4859E-08
Mycbp2	3510.43788	1.667257575	3.17610273	6.23681635	3.546E-08
Havcr2	77.6371153	2.928455693	7.61295048	6.22668191	3.7699E-08
Ccr2	349.778657	2.119095111	4.34421381	6.21788565	3.9734E-08
Il16	315.568081	2.425729647	5.37300674	6.2151422	4.0294E-08
Gm6904	76.3808281	2.58252384	5.98986646	6.2133395	4.0619E-08
Ighv9-3	53.7065273	3.009087558	8.05055117	6.20725777	4.2076E-08
Plekho2	720.618142	1.900656448	3.73383053	6.20216036	4.3313E-08
Csf1r	2280.20389	1.735724415	3.33046683	6.19795205	4.4334E-08
Camk1d	315.95266	2.079198132	4.2257228	6.16999767	5.2747E-08
C5ar2	42.1479367	2.980383334	7.8919583	6.15661021	5.7014E-08

Pik3cd	376.299774	1.920871353	3.78651686	6.15683756	5.7014E-08
Clec10a	106.445625	2.52387461	5.75124625	6.14425326	6.1425E-08
Rhoh	91.8573801	2.917965847	7.55779741	6.13594479	6.4289E-08
F13a1	30.1641456	3.979313242	15.7722135	6.13083314	6.6167E-08
Was	85.0111795	2.819367581	7.05852912	6.11542396	7.2646E-08
Gbp2	386.135911	1.788163757	3.45375024	6.09935797	7.9805E-08
Gm13910	3967.19075	1.414897075	2.66640712	6.09395186	8.2276E-08
Tpm3-rs7	7609.18449	1.789810157	3.4576939	6.09119485	8.343E-08
AC159261,1	112.593788	2.575198365	5.95952921	6.09008212	8.3737E-08
Wdr72	1560.60192	1.510029507	2.84815864	6.08702756	8.5071E-08
Lpxn	269.814695	2.33044283	5.02959708	6.07656799	9.0511E-08
Oplah	8010.30608	1.60390126	3.03964167	6.07527549	9.0947E-08
Taf1	1472.16708	1.887282081	3.69937636	6.06614501	9.5956E-08
Slc15a3	200.537229	2.13986541	4.40720929	6.05900075	9.9992E-08
Nup205	547.2958	1.67209326	3.18676638	6.03801748	1.1353E-07
Ppl	129.502121	2.202081405	4.60142721	6.02501025	1.2266E-07
Smpdl3b	109.253662	2.546322855	5.84143513	6.02025201	1.2591E-07
Tpm3	6647.98095	1.768468928	3.40692202	6.00925914	1.339E-07
Adcy7	157.401527	2.071566637	4.2034288	6.00777737	1.347E-07
Ambra1	1613.4314	1.488932736	2.80681258	6.00193815	1.392E-07
Flii	2751.26252	1.583247643	2.99643617	5.99035551	1.4901E-07
Nup98	3180.86405	1.822151066	3.53608037	5.98696145	1.5168E-07
Stambp	1681.93455	1.807312546	3.4998972	5.9856468	1.5243E-07
Smu1	2589.91255	1.617712736	3.06888107	5.98232808	1.5508E-07
Fam105a	165.230318	2.171117413	4.50372086	5.97656071	1.6017E-07
Ctr9	2467.6749	1.684523228	3.21434153	5.96549171	1.7089E-07
Herc2	2259.14165	1.658551559	3.15699409	5.95953799	1.7668E-07
2900026A02Rik	1267.11926	1.748929141	3.36108992	5.95209544	1.8378E-07
Sh2d1b2	40.4115729	4.204920557	18.441966	5.94516585	1.911E-07
Tifa	299.686963	1.674750561	3.1926415	5.94469463	1.911E-07
Msr1	109.339005	2.924687581	7.59309249	5.93484156	2.0232E-07
Sf3b3	1942.15227	1.53458772	2.8970563	5.92779953	2.1055E-07
Suox	2909.53936	1.69202309	3.23109482	5.92609877	2.121E-07
Mmp13	192.068521	2.434344959	5.4051886	5.91823708	2.2182E-07
Tlr7	71.2246448	2.649911172	6.27628634	5.91657226	2.234E-07
Supt6	3230.84567	1.655794673	3.15096706	5.89689434	2.5098E-07
Dnajc13	7736.35679	1.694198439	3.23597046	5.89226096	2.5735E-07
I830077J02Rik	75.1929098	2.490952963	5.62149152	5.89059551	2.5918E-07
Ifi209	162.814804	2.182391373	4.53905312	5.88538082	2.667E-07
Itpr2	2437.33536	1.477775491	2.7851895	5.88437801	2.6752E-07
Elp2	1948.06816	1.525548935	2.87896238	5.86398249	3.0168E-07
Mcm3ap	975.53501	1.603686704	3.03918965	5.86207276	3.0427E-07
Mpdz	858.50501	1.848930375	3.60233006	5.85969723	3.0775E-07
Apobec1	559.726752	1.908028183	3.7529581	5.85614898	3.1347E-07
Dse	229.933675	2.123072627	4.35620735	5.84841715	3.2744E-07
Aplp2	9767.59488	1.478297425	2.7861973	5.84779312	3.2771E-07
Ppp1r18	266.087056	1.924379979	3.79573685	5.83746993	3.4766E-07
Pla2g7	443.709087	2.363069662	5.14463834	5.82884276	3.6506E-07
Ubr3	3788.25431	1.607864293	3.04800294	5.8224646	3.7818E-07
Ccl8	31.8652079	3.716710448	13.147444	5.82006665	3.8254E-07

Sptb	318.253295	1.965580478	3.90569821	5.81472945	3.9382E-07
Prex1	444.987674	1.932080252	3.81605047	5.79900258	4.3135E-07
Tmsb4x	24174.7536	1.80054269	3.48351238	5.79564517	4.3882E-07
Hexb	1162.3778	1.433725014	2.70143321	5.79506608	4.3908E-07
Map3k1	1794.82128	1.372582022	2.58933571	5.79055977	4.4975E-07
Oasl2	797.852041	1.753967375	3.37284817	5.77478608	4.9258E-07
BC147527	71.1780366	2.682908191	6.42149045	5.77219173	4.9742E-07
Gbp2b	98.1763747	2.388701624	5.2368585	5.77227192	4.9742E-07
Pfas	1182.74068	1.61126629	3.05519887	5.76894508	5.0567E-07
Mad1l1	653.464856	1.867785768	3.64971995	5.76449614	5.1775E-07
Ank3	9960.25829	1.911225722	3.76128525	5.7589139	5.3367E-07
Ppp1r9b	1521.20411	1.85100756	3.60752042	5.7324164	6.2241E-07
Dpysl2	3012.78106	1.949172025	3.86152852	5.71874577	6.7274E-07
Retnla	40.9543638	3.619378889	12.2897093	5.70122806	7.4362E-07
Batf	63.3548765	2.481431364	5.58451257	5.70037306	7.453E-07
Cep85	10622.6771	1.433964881	2.7018824	5.69775217	7.5476E-07
Lsp1	1046.02619	1.986482466	3.96269647	5.69583701	7.612E-07
Aifm1	9571.40014	1.543637099	2.91528534	5.69179965	7.7729E-07
Tnfsf13b	186.122411	2.410077342	5.31502818	5.68192127	8.2131E-07
Fam83h	777.446816	1.518264598	2.86446279	5.68090605	8.2396E-07
Plbd1	691.319744	2.168996151	4.4971037	5.67820669	8.348E-07
P2ry12	36.5012263	3.383917902	10.4390455	5.67177618	8.6441E-07
Mut	11489.5354	1.521139325	2.87017624	5.67065603	8.654E-07
Fli1	583.607259	1.503098363	2.83450804	5.6707848	8.654E-07
Hltf	1257.36554	1.589119976	3.0086577	5.66813068	8.759E-07
Polr2b	1839.62919	1.480774953	2.79098613	5.66710373	8.7881E-07
Plagl2	355.55617	1.781992803	3.43900879	5.66658633	8.7911E-07
Nup107	306.581501	1.794143111	3.46809426	5.66440784	8.8798E-07
Trappc9	4451.23609	1.491087995	2.81100885	5.65644511	9.2634E-07
Psmd2	9303.66449	1.444518233	2.7217192	5.65581925	9.2634E-07
Ikbkap	812.22943	1.36848938	2.58200068	5.6534015	9.3682E-07
Msh6	583.70121	1.553253243	2.9347818	5.65233475	9.4018E-07
Ascc3	1148.38562	1.69388589	3.23526949	5.63660745	1.0275E-06
D6Wsu163e	801.869279	1.498209687	2.82491936	5.6279964	1.0773E-06
Cxcl16	1882.05641	1.754945263	3.37513513	5.62626602	1.0853E-06
Cfp	152.005878	1.935594727	3.82535789	5.62548702	1.0874E-06
Rtp4	631.346677	1.82029858	3.5315428	5.62293287	1.1007E-06
Rapgef3	3257.63561	1.554129148	2.93656414	5.62031515	1.1146E-06
Arhgdib	1161.72433	2.006347676	4.01763827	5.61575541	1.1415E-06
Tmem229b	277.947205	1.775211607	3.42288212	5.6100634	1.1767E-06
Pwp2	670.108869	1.650066648	3.13848138	5.60803372	1.1875E-06
Gtf3c1	3328.21281	1.547921505	2.9239558	5.60204864	1.2261E-06
Gpr34	59.3276567	2.353342591	5.11006836	5.60100879	1.2303E-06
Sirpb1c	108.290704	2.543673474	5.8307177	5.59544237	1.2672E-06
Slamf8	232.036655	2.233949892	4.70420161	5.58465606	1.3447E-06
Lgals3bp	1126.65742	1.67620434	3.19586029	5.58381604	1.3447E-06
Dync1h1	7735.52174	1.667538892	3.17672211	5.58420215	1.3447E-06
Oasl1	109.642403	1.861703766	3.63436613	5.58235097	1.3527E-06
Sirpb1b	80.599524	2.550102607	5.85675931	5.5672167	1.4718E-06
Olfr286	38.1320423	2.821648581	7.06969796	5.56180982	1.5144E-06

Usp37	1572.89807	1.364628211	2.57509956	5.5592801	1.5326E-06
Nup133	446.69852	1.644428074	3.12623899	5.54161826	1.6913E-06
Helz2	217.314519	1.671471326	3.18539289	5.5400352	1.7025E-06
Fes	377.061925	1.858747588	3.6269267	5.53712381	1.7267E-06
Phf11a	271.131858	1.913552412	3.76735611	5.53447214	1.7487E-06
Pde4c	2054.97647	1.436707789	2.70702421	5.52529444	1.838E-06
Dpys	420.912186	1.68645997	3.21865952	5.50212995	2.0906E-06
Zc3hc1	1067.65602	1.327036599	2.50886805	5.50178086	2.0906E-06
Asb2	140.617223	2.001942757	4.00539009	5.49660043	2.1476E-06
Lrrc25	233.068065	2.362308502	5.14192477	5.49269911	2.1903E-06
Haus3	265.88107	1.780994089	3.43662894	5.49006128	2.207E-06
Mccc1	13316.8205	1.515341891	2.85866565	5.4906139	2.207E-06
Exoc4	2350.98753	1.449438445	2.73101728	5.49033972	2.207E-06
Tor3a	395.620664	1.49984732	2.82812781	5.4856355	2.2575E-06
Rab3gap1	2174.1058	1.490872223	2.81058846	5.48423268	2.27E-06
Gprasp1	619.586179	1.427537009	2.68987104	5.4830963	2.2792E-06
1700024P16Rik	374.854107	2.022426063	4.06266403	5.48083499	2.3029E-06
Tbk1	808.210336	1.359921071	2.56671137	5.47829746	2.3306E-06
Ighv4-1	36.2672432	3.135530069	8.78797076	5.47069922	2.427E-06
Pla2g15	318.295123	2.156424929	4.45808749	5.46456796	2.5063E-06
Eif3d	3496.37903	1.468631673	2.76759276	5.44373437	2.8114E-06
Cramp1l	1531.65279	1.212596129	2.31754304	5.43502653	2.9452E-06
Selenbp1	53510.4871	1.35392025	2.55605743	5.42640022	3.0837E-06
Epb41l2	549.867983	1.43882277	2.7109956	5.42245845	3.1451E-06
Znfx1	3420.77586	1.394378092	2.62875209	5.421054	3.1624E-06
Ccl7	35.0111822	2.82696473	7.09579697	5.41964399	3.1673E-06
Apoe	7778.11571	1.920162844	3.78465776	5.41961105	3.1673E-06
Ogdh	38197.5333	1.432270897	2.69871176	5.41951949	3.1673E-06
Zc3hav1	1519.21081	1.392700169	2.6256965	5.41847177	3.1784E-06
Nexn	271.397394	2.173132482	4.51001578	5.41241655	3.2649E-06
Maoa	394.651849	1.49418377	2.81704726	5.41322043	3.2649E-06
Tlr13	95.2969716	2.467864308	5.53224216	5.40503034	3.3852E-06
Mcm4	271.319894	1.652787322	3.1444056	5.40541363	3.3852E-06
Polr1b	692.351967	1.484438682	2.79808285	5.40469485	3.3852E-06
Selenbp2	35019.1724	1.347897382	2.54540881	5.4015615	3.437E-06
Nup160	318.688543	1.435580021	2.70490893	5.39964316	3.4659E-06
Kdm5a	3432.1292	1.895157044	3.71962465	5.39460766	3.5564E-06
Tnfrsf1b	160.008228	1.90725277	3.75094152	5.39252704	3.5896E-06
Lrmp	120.285959	2.313527355	4.97096986	5.38725733	3.688E-06
St5	1120.16692	1.370326283	2.58529029	5.37693406	3.8881E-06
Ptprd	4488.15089	1.570480402	2.97003597	5.37108662	4.0072E-06
Ifi206	154.513704	1.976395028	3.93508562	5.36863685	4.0528E-06
Hadhb	9523.5081	1.238147414	2.35895421	5.36241753	4.1855E-06
Fgl2	152.887841	2.001115701	4.00309458	5.35687743	4.3061E-06
Dapp1	142.72622	2.143453857	4.41818509	5.34262208	4.6382E-06
Pmpca	4393.78571	1.30359235	2.46842763	5.33796203	4.7483E-06
Inpp5d	440.511843	1.498101248	2.82470704	5.33745747	4.7509E-06
BC035044	118.674563	2.42948515	5.38701152	5.3369946	4.7525E-06
Gm42715	185.241369	1.71121367	3.27436164	5.33614884	4.7641E-06
Ranbp2	2331.72219	1.560934813	2.9504496	5.3338185	4.815E-06

Lonp2	5640.43355	1.355881173	2.559534	5.33284574	4.8302E-06
4933424M12Rik	34.2339391	3.012493729	8.06958079	5.32194135	5.1177E-06
Atp6v1a	48278.2167	1.65499065	3.1492115	5.31810409	5.2153E-06
Mef2c	831.564336	1.883996545	3.69096113	5.31603268	5.2634E-06
Cpsf1	2123.61437	1.380003931	2.6026908	5.30628346	5.5284E-06
Aco1	15745.8434	1.264382031	2.40224289	5.30644028	5.5284E-06
Slc14a1	37.2340941	2.935301546	7.64916117	5.29936331	5.7296E-06
Usp18	234.709146	1.487721297	2.80445668	5.29878365	5.7353E-06
Akap11	2113.66918	1.399677699	2.63842633	5.29806297	5.7455E-06
Rassf5	358.350161	1.815443079	3.5196771	5.29352223	5.8773E-06
Syk	221.159197	2.111802984	4.32231131	5.29188294	5.9175E-06
Clec12a	107.182378	2.380203699	5.20610244	5.2902528	5.9576E-06
H2-Q6	1511.69545	1.144435326	2.21059591	5.28710341	6.048E-06
Ncaph	131.122349	2.200689197	4.59698895	5.28664725	6.0501E-06
Dysf	516.955307	1.750242872	3.36415195	5.28496302	6.0929E-06
Arhgef17	1126.36614	1.570347087	2.96976153	5.28193946	6.1811E-06
Unc13d	52.3684572	2.35108189	5.10206716	5.28114259	6.1948E-06
Sesn1	800.291316	1.382006631	2.60630628	5.28062075	6.1993E-06
Sdc3	559.436246	1.998503601	3.99585325	5.27898736	6.2415E-06
Pdp2	16156.313	1.769605378	3.40960681	5.27045617	6.5249E-06
Nbas	989.833842	1.575444703	2.98027344	5.2677612	6.6074E-06
Zcchc3	357.240645	1.761607651	3.3907576	5.26260251	6.7812E-06
Coro7	1883.21677	1.375253844	2.59413551	5.26065265	6.8391E-06
Oas3	72.198744	2.139921203	4.40737973	5.25949148	6.8658E-06
Cobl	1969.54748	1.504076475	2.83643042	5.25916368	6.8658E-06
Mycn	110.540132	1.890406683	3.70739718	5.25799848	6.895E-06
Arhgef6	315.339127	1.812960989	3.51362686	5.2575765	6.8964E-06
Mcm7	1038.43859	1.316116126	2.48994888	5.23931015	7.5992E-06
Ptpro	304.114108	1.65598968	3.151393	5.22847121	8.0416E-06
Scimp	126.722485	2.444364416	5.44285807	5.21704502	8.5183E-06
Spn	167.523882	2.050125806	4.14142082	5.21709087	8.5183E-06
Acaca	992.312898	1.444634634	2.72193881	5.2115296	8.7574E-06
Dsp	392.480906	1.482023567	2.7934027	5.20721242	8.945E-06
Ccl9	114.376942	2.262399884	4.79788934	5.2035848	9.1027E-06
Jak1	6289.93169	1.344940654	2.54019747	5.19348068	9.5795E-06
Apeh	13532.7491	1.342792148	2.53641735	5.19332929	9.5795E-06
Myo19	2075.59791	1.416773004	2.66987649	5.1903938	9.7119E-06
Igha	17864.7463	2.689800373	6.45224121	5.1871256	9.8505E-06
Nup43	523.813428	1.364503397	2.57487679	5.18699342	9.8505E-06
Apbb1ip	109.000155	1.897602956	3.72593616	5.18568685	9.8997E-06
Vps16	1501.51261	1.393646694	2.62741974	5.18121141	1.012E-05
Dock9	2360.02006	1.36258563	2.57145629	5.17960893	1.0186E-05
Actr6	600.033512	1.313320756	2.48512902	5.17459034	1.0443E-05
Dock2	213.343852	2.015832859	4.04413977	5.17238639	1.0545E-05
Tbl3	1684.83783	1.319606703	2.49598057	5.16233047	1.1106E-05
40422	876.84162	1.396817508	2.63320074	5.1505428	1.1803E-05
Synpo	887.332666	1.518088257	2.86411269	5.14996368	1.1816E-05
Fnip1	1832.71016	1.568861403	2.96670485	5.13997873	1.2437E-05
Gm5547	44.9700745	2.332557146	5.03697352	5.13587249	1.2661E-05
Wdr36	1379.04658	1.397805216	2.63500412	5.13592188	1.2661E-05

Kcnk13	28.7553677	2.789269884	6.91279855	5.13406574	1.2757E-05
Ifitm6	30.8308682	2.953869177	7.74824288	5.12677538	1.3235E-05
Gm14548	61.6586734	2.272375884	4.83118093	5.11685204	1.3895E-05
Tnks1bp1	767.489846	1.609891111	3.05228803	5.11582602	1.3943E-05
Shroom4	1898.53446	1.263497211	2.40077002	5.11223462	1.4183E-05
Pik3r5	107.552736	2.445917761	5.44872153	5.10720783	1.452E-05
Abcg2	11673.5619	1.179206187	2.26452142	5.10706159	1.452E-05
Enc1	246.560411	1.813253494	3.51433932	5.10210467	1.4876E-05
Ctnnd2	45.1626701	2.572928895	5.95016178	5.09924613	1.5073E-05
Plat	460.436113	1.453053329	2.73786883	5.09651985	1.5262E-05
Btg2	1082.91841	1.495847997	2.82029876	5.09415924	1.5423E-05
Parn	842.69765	1.272352596	2.41555147	5.09096659	1.5655E-05
Rtn1	90.5090379	2.231973963	4.6977631	5.08751195	1.5912E-05
Itgax	146.60652	2.122839182	4.35550252	5.08368936	1.6203E-05
G530011O06Rik	186.795135	1.974332305	3.92946337	5.08333061	1.6203E-05
Lars	1119.22644	1.356529727	2.56068488	5.08195178	1.6258E-05
Hpgds	204.065579	2.184263106	4.54494585	5.08057447	1.6345E-05
Dock10	277.516733	1.677621138	3.19900032	5.06758152	1.7468E-05
Cep41	160.748222	1.653001653	3.14487277	5.06429051	1.7723E-05
Rtcb	6680.0063	1.277776313	2.42464967	5.06408456	1.7723E-05
Gm5131	562.270735	1.34339614	2.53747945	5.06203295	1.7881E-05
Ear2	49.5537838	2.450494346	5.46603367	5.06098071	1.7946E-05
Ptprs	430.370755	1.481475477	2.79234166	5.05646141	1.8341E-05
Gm5422	2190.54541	1.24176527	2.3648772	5.05026796	1.891E-05
Cd37	110.293685	1.940914822	3.83949035	5.04947378	1.8953E-05
H2-T-ps	774.764569	1.125519409	2.18180083	5.04445836	1.942E-05
Jag1	774.99479	1.525480634	2.87882608	5.04001779	1.9839E-05
Wipf1	193.195275	1.665390033	3.17199398	5.03871542	1.9937E-05
Irf9	709.826683	1.230427545	2.34636514	5.03616248	2.0167E-05
Cfh	2494.4635	1.361250876	2.56907833	5.03535877	2.0176E-05
Cluh	13353.9006	1.332467244	2.51832982	5.03548368	2.0176E-05
Parp14	445.176118	1.473651424	2.77723917	5.03251334	2.0439E-05
Man2c1	1464.19662	1.35440258	2.55691213	5.03000524	2.0664E-05
Utp4	1079.16561	1.181837995	2.2686562	5.02970251	2.0664E-05
Ciao1	3024.55028	1.355181534	2.55829305	5.02753075	2.0861E-05
Il1r2	35.774877	2.678289168	6.40096388	5.00952902	2.2783E-05
Lacc1	334.362855	1.424317674	2.68387536	5.00899367	2.2804E-05
Tns4	75.4240973	2.270584596	4.82518613	5.00491421	2.3207E-05
Dars	3156.35356	1.217475212	2.32539406	5.00503753	2.3207E-05
Dock11	96.8008599	2.016481153	4.04595746	4.9979641	2.4015E-05
Ptpre	270.376519	1.897738573	3.72628643	4.99601444	2.4214E-05
Tlr2	148.888116	2.181505078	4.53626549	4.9919907	2.4679E-05
Gfm1	5348.93135	1.370598821	2.58577872	4.99047415	2.4829E-05
H2-Q1	1462.20495	1.068651498	2.09747192	4.98992037	2.4855E-05
Dhx29	1145.29655	1.322871876	2.50163598	4.98211311	2.5785E-05
Notch2	1444.63243	1.102698031	2.14755939	4.98244342	2.5785E-05
Cep170	240.27887	1.467261126	2.76496482	4.97782591	2.6315E-05
Rgs19	262.003044	1.649609387	3.13748679	4.97394565	2.6751E-05
Fam129a	598.060543	1.509779376	2.84766488	4.97422652	2.6751E-05
Wdr81	2912.73155	1.419247017	2.67445887	4.97344065	2.6773E-05

2900097C17Rik	9323.22167	1.2546279	2.386056	4.95217591	2.9818E-05
Nedd4	8906.08264	1.231265604	2.34772854	4.94915736	3.023E-05
Rasa4	231.950127	1.612224905	3.0572296	4.94878433	3.0233E-05
Mcm5	378.189402	1.598520696	3.02832637	4.94744798	3.0387E-05
H2-Q5	351.512269	1.238737127	2.35991865	4.94626795	3.0517E-05
Gm8953	26.2512322	2.743371443	6.69633379	4.94236778	3.1079E-05
Arhgap22	33.8169312	2.546305242	5.84136381	4.93780144	3.1759E-05
Cd84	71.4324112	2.336781928	5.05174539	4.93703152	3.1828E-05
Cass4	22.0304733	3.359200268	10.2617172	4.93472048	3.2093E-05
Fgr	122.11109	1.955628449	3.87884856	4.9348581	3.2093E-05
Hhex	80.4822324	2.092842832	4.26587837	4.93424057	3.2116E-05
Ift122	3018.03225	1.222476319	2.33346903	4.92381362	3.3816E-05
Ighv1-53	35.4503118	2.601964032	6.07112565	4.92047592	3.4338E-05
Inpp4a	1323.42481	1.272119323	2.41516093	4.91103048	3.5971E-05
Plcg1	1029.34137	1.472569198	2.77515663	4.9022103	3.7559E-05
Ticam2	43.0945948	2.612553455	6.11585183	4.88088875	4.1711E-05
H2-Q2	632.087981	1.114397681	2.165046	4.87807611	4.2236E-05
Snd1	2818.15053	1.307363793	2.47488895	4.87754728	4.2256E-05
Inpp5b	2983.20569	1.128606197	2.18647401	4.8772981	4.2256E-05
Itpripl1	1522.64161	1.500146314	2.82871399	4.8740987	4.2872E-05
Pdcd11	690.274288	1.372749216	2.5896358	4.86949498	4.3807E-05
Hadha	7017.70482	1.308116552	2.47618062	4.86211638	4.5316E-05
Pilrb1	49.6829308	2.45398457	5.47927332	4.86114778	4.546E-05
AC160122,1	227.154637	1.689802681	3.22612576	4.85412307	4.702E-05
Dlec1	1413.62066	1.473675181	2.7772849	4.85350007	4.7087E-05
AB124611	112.073593	2.258626788	4.78535777	4.85136895	4.7514E-05
Zfp568	477.193238	1.334578374	2.52201765	4.84652477	4.8606E-05
Apobec3	512.982818	1.435427332	2.70462267	4.83959342	5.0246E-05
Hsd17b4	8713.28025	1.217070443	2.32474172	4.83727895	5.0748E-05
Ifi27l2a	1865.9203	1.924142372	3.79511176	4.83420106	5.1451E-05
Crnkl1	668.771276	1.265398741	2.40393642	4.83290232	5.17E-05
Ighv1-72	28.369803	2.521133535	5.74032944	4.82907491	5.2615E-05
Ubr5	4059.99684	1.28903942	2.44365297	4.80940428	5.787E-05
Ipp	499.442183	1.209266799	2.31220097	4.80853691	5.8024E-05
AC156572,1	910.32679	1.117903046	2.17031289	4.80675381	5.8445E-05
Atf3	113.740474	1.898217449	3.7275235	4.8060116	5.8563E-05
Mcmbp	1886.74596	1.137173492	2.19949678	4.79631055	6.1367E-05
Smc5	744.035467	1.213229136	2.31856012	4.79532838	6.1566E-05
Spg11	533.48753	1.216445644	2.32373515	4.79457525	6.1694E-05
St3gal6	592.445264	1.422177169	2.67989629	4.7922052	6.2323E-05
Gm3650	540.535994	1.427245981	2.68932848	4.79010592	6.2874E-05
Phka1	361.06742	1.477172013	2.7840247	4.78845623	6.3287E-05
Trim30a	476.966203	1.522773374	2.87342894	4.77657082	6.7032E-05
Dhtkd1	1089.41785	1.416483596	2.66934096	4.77556719	6.7256E-05
Hacl1	1411.98113	1.091697534	2.1312466	4.772559	6.8043E-05
Il21r	62.5950788	2.256380073	4.77791131	4.77104727	6.8442E-05
H2-Q7	1314.15114	1.049164508	2.06933111	4.76856941	6.9147E-05
Arhgef28	3661.53858	1.3147337	2.4875641	4.76622431	6.9755E-05
Mga	1419.38996	1.37640108	2.5961992	4.76550565	6.9889E-05
Efhd2	1147.4916	1.535269241	2.89842517	4.76377925	7.0375E-05

Acsl4	2583.45677	1.332965391	2.51919953	4.76344125	7.0378E-05
Xirp1	24.0985487	2.717795412	6.57866756	4.76209477	7.0618E-05
Ccdc88b	83.7992763	2.088204351	4.25218496	4.75057985	7.4398E-05
Pira2	71.2252464	2.001105916	4.00306743	4.74192122	7.7274E-05
Hk3	90.2458796	2.115814701	4.33434713	4.73612209	7.9262E-05
Kbtbd7	385.190964	1.280611533	2.42941934	4.73495465	7.9591E-05
Rgs2	884.913205	1.466903151	2.76427884	4.73408658	7.9805E-05
Pea15a	2554.21985	1.281915861	2.43161675	4.72973257	8.1405E-05
Fuk	591.48349	1.467901764	2.7661929	4.72300606	8.3743E-05
Bub3	1857.96953	1.171780098	2.25289503	4.72323922	8.3743E-05
Yod1	1765.95398	1.473929554	2.77777463	4.72115927	8.4373E-05
Gigyf1	1880.46261	1.557802884	2.94405145	4.71833084	8.5419E-05
Dst	4843.62881	1.166455893	2.24459614	4.71620142	8.6044E-05
Pcm1	1545.58908	1.176274847	2.25992493	4.71318753	8.7189E-05
Bbs2	1008.49087	1.357077257	2.5616569	4.7078931	8.9202E-05
Bckdha	1851.8317	1.27572779	2.42120929	4.70609377	8.9851E-05
Csf2rb2	92.501376	1.936082698	3.82665198	4.70382769	9.0606E-05
Vps13b	2326.89837	1.435252412	2.70429476	4.70083266	9.1765E-05
Irf8	1127.18936	1.158968221	2.23297674	4.69295397	9.5074E-05
Aim1	1011.95757	1.351136179	2.55112958	4.68471109	9.8827E-05
Zfp316	304.839983	1.564878209	2.95852525	4.6824742	9.9757E-05
Marcks	1757.32997	1.517749387	2.86344003	4.68013437	0.0001007
Gm8909	799.469337	1.05932908	2.08396216	4.6799075	0.0001007
Mkln1	2628.86452	1.153037186	2.22381563	4.6786893	0.00010114
Pex14	1929.27112	1.419563096	2.67504488	4.67533431	0.00010254
Ints9	792.68219	1.281456929	2.43084336	4.6732308	0.00010339
Amot	876.940866	1.351742569	2.55220209	4.67017773	0.00010462
Gys1	1358.51907	1.297625673	2.45823983	4.66694974	0.00010608
Stab1	615.629423	1.542312835	2.9126106	4.66408524	0.00010727
Tgm1	1879.96545	1.233397414	2.35120024	4.66221595	0.00010808
H2-Q4	1124.69091	1.035605393	2.04997369	4.66044759	0.00010885
Shprh	434.725693	1.302046285	2.46578376	4.65416531	0.00011188
Fto	4212.55062	1.143257789	2.20879234	4.63951821	0.00011975
Tbck	2659.77237	1.292007404	2.44868535	4.63403428	0.00012278
Man1c1	151.408832	1.822819731	3.53771966	4.63128056	0.00012423
Myo9b	962.182697	1.534906928	2.89769737	4.62884792	0.00012551
Kdm5c	1710.59392	1.388889997	2.61877116	4.62669583	0.00012644
Sp100	627.507515	1.173438934	2.25548694	4.62674531	0.00012644
Gm7292	1736.25022	1.004119165	2.00571854	4.621935	0.00012899
5430427O19Rik	57.9544672	2.238265543	4.71829474	4.61746017	0.00013161
Ciita	300.506113	1.605726909	3.0434906	4.61523917	0.00013282
Bend6	50.3665822	2.15826815	4.46378688	4.61260098	0.00013432
Amotl2	1591.53354	1.291199538	2.44731455	4.61150894	0.00013483
Stap1	223.20051	1.697617477	3.24364847	4.61006306	0.00013542
Gm4788	164.932228	1.572835346	2.97488798	4.60997548	0.00013542
Fut4	65.8617347	1.939619616	3.83604492	4.60710723	0.0001371
Ep400	1861.38829	1.295436917	2.45451319	4.60293702	0.00013967
Tcaf1	701.103779	1.237135059	2.35729949	4.60166974	0.00014031
Pip4k2a	571.603876	1.568757756	2.96649172	4.60079113	0.0001407
Jaml	62.5645916	1.847304435	3.59827247	4.59950977	0.00014136

Itpr3	151.293841	1.740897398	3.34243012	4.59516109	0.00014397
Mrip-ps	149.262125	1.654152036	3.14738145	4.59508302	0.00014397
Gm10499	630.070108	1.075275286	2.1071241	4.58854095	0.00014833
Ubr2	2090.27797	1.310588633	2.48042723	4.5874932	0.00014884
Hexa	1469.34628	1.262428738	2.39899265	4.58722127	0.00014884
Tbpl1	634.321416	1.145831671	2.21273652	4.58526599	0.00015002
Bcor	623.373943	1.410708385	2.65867676	4.58206589	0.00015211
Dhx15	3266.90256	1.206657028	2.30802208	4.58106468	0.00015262
Acadvl	6794.13314	1.390981523	2.62257044	4.57015905	0.00016031
Irf5	1195.15305	1.25537337	2.38728924	4.56168996	0.00016643
Ahctf1	1375.96162	1.237484033	2.35786976	4.56171107	0.00016643
Abcg1	503.95449	1.584528717	2.99909811	4.55978985	0.0001677
Samhd1	723.362524	1.083579013	2.11928706	4.55757146	0.00016924
Lyl1	70.2306629	1.851486085	3.60871719	4.55624148	0.00017007
Gm7848	60.7302572	1.955868286	3.87949344	4.54775802	0.0001763
Stxbp3	1533.80218	1.117209815	2.16927028	4.54660986	0.00017701
Ccl3	29.2760002	2.448658251	5.45908156	4.54453642	0.00017851
Gm15931	52.0413014	1.910402466	3.75913953	4.52694778	0.00019294
Srgn	533.852767	1.332637946	2.51862781	4.52746289	0.00019294
Skiv2l2	2788.83058	1.222608482	2.3336828	4.52662484	0.00019294
Gm7030	628.626333	1.051291717	2.07238453	4.52674353	0.00019294
Phf11c	277.713176	1.421816446	2.67922631	4.52510083	0.00019406
Pygb	1314.78912	1.25736666	2.3905899	4.51231417	0.00020439
Dpysl3	679.262128	1.372296382	2.58882309	4.51185192	0.00020455
Vnn3	12.3182842	2.509073706	5.69254467	4.51038436	0.00020556
Xpnpep1	9873.29243	1.066180621	2.0938827	4.5102155	0.00020556
Rnase6	92.4919359	1.745053454	3.35207273	4.5071738	0.00020794
Elmo1	105.432464	1.95301223	3.87182093	4.50146268	0.00021272
Cnot8	2762.02355	1.092547185	2.13250213	4.4991401	0.00021475
Dck	290.083507	1.227120373	2.3409926	4.49779528	0.00021582
Pikfyve	1536.07875	1.128181848	2.18583098	4.49360375	0.00021981
AC155249,1	158.896486	1.523930472	2.87573447	4.49138989	0.0002218
Mccc2	4532.09611	1.10324347	2.14837147	4.49072786	0.00022218
Il2rg	107.267438	1.642708055	3.12251403	4.48920737	0.00022315
Hps3	1280.57828	1.190068084	2.28163511	4.48486678	0.00022743
Hmgcs1	21072.566	1.188835457	2.27968653	4.48337927	0.00022839
Srpk3	46.6249584	2.198130904	4.58884445	4.47684902	0.00023484
H2-T23	2152.36303	1.021044078	2.02938709	4.47317852	0.00023859
Baz1a	678.882399	1.263173771	2.40023185	4.47265276	0.00023885
Ift140	755.89127	1.273432669	2.41736055	4.47026926	0.0002412
Tep1	1271.81368	1.31829241	2.49370777	4.46733301	0.0002442
Bhmt2	11411.2912	1.282605595	2.43277955	4.46683097	0.00024434
Skiv2l	2758.61099	1.265817863	2.4046349	4.46662094	0.00024434
Rbbp7	3143.78511	1.062095356	2.08796185	4.46611634	0.00024459
Cnp	442.284289	1.439044598	2.71141247	4.4640719	0.0002466
Plekho1	442.327738	1.390230981	2.62120644	4.46308156	0.00024741
Coro1b	5897.73479	1.05534493	2.07821502	4.46060667	0.00024994
Txnip	4016.76018	1.00490556	2.00681213	4.46002028	0.00025029
Plxnc1	77.6340204	2.175643911	4.51787361	4.45728428	0.00025282
Dok1	291.507858	1.641027099	3.11887795	4.45733248	0.00025282

Pilrb2	71.5133854	2.16951621	4.49872509	4.45270816	0.00025793
AU020206	345.466639	1.49039812	2.80966499	4.45078493	0.0002599
Irf1	892.393007	1.128689804	2.18660072	4.45049793	0.0002599
Mettl14	388.211809	1.124740784	2.18062363	4.44887944	0.00026151
Dppa3	17.143232	2.873957822	7.33073483	4.44844274	0.00026169
Ifi211	378.800331	1.522068121	2.87202463	4.4477675	0.00026217
Pik3c3	1133.79912	1.243204204	2.36723709	4.442263	0.00026861
4632427E13Rik	144.530045	1.504071028	2.83641971	4.44012956	0.00027092
Nfam1	179.215895	1.737495811	3.33455862	4.43579504	0.0002757
Cct3	6471.25846	1.111537279	2.16075766	4.43594133	0.0002757
Igsf3	1067.28929	1.348336477	2.54618364	4.43235168	0.0002794
Nup88	1976.41462	1.237829437	2.35843434	4.43241529	0.0002794
Hk1	2209.32982	1.147326628	2.2150306	4.43196645	0.00027953
Arrb2	408.40995	1.368041786	2.58119974	4.42608476	0.0002865
Nemf	3127.64673	1.324161414	2.50387304	4.42293373	0.00029033
Tubgcp2	541.135636	1.143462969	2.20910649	4.41866124	0.00029573
Col12a1	134.65284	1.482084704	2.79352107	4.41068961	0.00030603
Pirb	221.867182	1.785906201	3.44834997	4.40648063	0.00031162
Akap8	2207.07544	1.171925313	2.25312181	4.4056062	0.00031248
Pcca	5068.58306	1.182935317	2.27038241	4.4038416	0.00031421
Papss1	10243.9781	1.3790294	2.6009333	4.40239631	0.0003159
Anapc4	1175.66534	1.091086841	2.13034464	4.40114445	0.00031693
Gbp3	475.556692	1.146250112	2.21337839	4.39974837	0.00031853
Stk17b	349.472006	1.366278292	2.57804651	4.39588953	0.00032382
Sipa1l1	6152.914	1.197914908	2.29407874	4.39497326	0.00032477
Gm26917	830.246416	1.476975512	2.78364553	4.39230955	0.00032835
Hsdl2	3173.52571	1.057345818	2.08109932	4.38584893	0.00033781
A530040E14Rik	79.9686369	1.761469205	3.39043222	4.38005032	0.00034647
Gng2	304.491211	1.467483844	2.7653917	4.37977546	0.00034647
1600010M07Rik	27.8476463	2.246674465	4.74587619	4.37938518	0.00034664
Mdn1	586.245364	1.147664418	2.21554928	4.37789303	0.00034813
Skil	1360.16921	1.087784201	2.12547339	4.37391822	0.00035408
Sirpb1a	63.925901	2.0317144	4.08890459	4.37338177	0.00035449
Olfr787	10.1414872	2.454883695	5.48268921	4.3678096	0.00036273
Pfkm	2342.55963	1.044546415	2.06271774	4.36695421	0.00036369
Gm16332	211.645479	1.41430603	2.66531497	4.36547181	0.0003657
Rhpn2	370.054567	1.081641088	2.1164422	4.36287263	0.0003696
Calhm2	58.0155113	1.793910543	3.46753524	4.36034117	0.00037343
Atg2b	1350.35925	1.171654058	2.25269822	4.35858998	0.00037595
Gm8815	316.450297	1.100320301	2.14402288	4.35756925	0.00037723
Klhl9	6453.84284	1.395968827	2.63165219	4.35459926	0.00038141
Vcam1	214.926646	1.448994314	2.73017668	4.352005	0.00038546
Sec23a	1124.21094	1.093748118	2.13427802	4.34890688	0.00039046
Cdca7	70.2753105	1.715310279	3.28367258	4.34668946	0.00039393
Emp3	216.610976	1.581234479	2.9922578	4.3425323	0.00039945
Tnxa	45.391558	2.1098036	4.31632531	4.34208808	0.00039975
Polr1a	1090.85032	1.232102884	2.34909146	4.3344797	0.0004128
Vps13d	3635.98724	1.253656598	2.38445011	4.3294772	0.00042176
Bptf	3058.6831	1.319314968	2.49547589	4.3241194	0.00043107
Wdr90	219.654472	1.48194948	2.79325925	4.32310975	0.00043197

Cxcl9	89.1961119	1.548540598	2.92521081	4.31918397	0.00043918
Usp8	12826.1945	1.392585238	2.62548734	4.31647331	0.00044405
Cpsf2	1786.23612	1.20326566	2.30260295	4.31117204	0.00045427
Gm4951	215.897758	1.704334186	3.25878503	4.30993462	0.00045626
Syde2	628.442423	1.078655452	2.11206678	4.30912671	0.00045737
Nol6	1329.37632	1.146588393	2.21389744	4.30589491	0.00046295
Pepd	33091.2845	1.115909109	2.16731539	4.30330878	0.00046782
Pyroxd1	332.087038	1.184146443	2.27228917	4.30123552	0.00047164
Sacm1l	396.019751	1.234776742	2.35344925	4.29902401	0.00047462
Kdm4b	785.225831	1.348388388	2.54627526	4.29731262	0.0004769
Trpm2	63.5335199	1.717338244	3.28829161	4.29647921	0.00047776
Adck1	1200.39188	1.072325371	2.10282001	4.29303306	0.00048406
Bmp2k	2275.76195	1.110189583	2.15874013	4.29068325	0.00048862
Tnxb	227.935067	1.627076606	3.08886454	4.29022602	0.00048904
Itpka	78.4954348	2.084731654	4.24196187	4.28705167	0.00049431
Trpv2	192.101007	1.505652838	2.83953135	4.28703996	0.00049431
Ifi214	61.218149	1.83161131	3.55934385	4.28461766	0.00049857
Rhog	1215.51476	1.158773316	2.23267509	4.2845958	0.00049857
Mafb	530.441717	1.191819867	2.28440725	4.27589935	0.00051533
Chd8	1663.49861	1.147678077	2.21557026	4.27425791	0.00051853
Ighd	43.3804435	1.838981766	3.57757439	4.27344778	0.00051878
Ttc27	679.077394	1.270432489	2.41233871	4.27383871	0.00051878
Pfkp	3565.17807	1.288274476	2.44235765	4.26326296	0.00054084
Ythdc2	291.29139	1.326775425	2.50841391	4.26067219	0.00054521
Tmem71	66.4923303	2.209538659	4.62527344	4.25665287	0.00055379
Uqcrc2	14524.2708	1.066055584	2.09370123	4.25583054	0.00055518
Gm7839	49.2956376	1.908214847	3.75344371	4.25239969	0.00056243
Arhgap25	159.325366	1.904043939	3.74260798	4.2506204	0.00056625
Klhl6	454.633463	1.399315107	2.63776329	4.24728058	0.00057341
Wdr35	880.869292	1.078951319	2.11249997	4.24421182	0.00057928
Mcm2	532.739256	1.05210199	2.07354878	4.24430856	0.00057928
Mag	59.8234868	1.658897265	3.15775067	4.24008081	0.00058841
Wdr75	603.073699	1.24759682	2.37445567	4.23876527	0.00059075
Lpin1	3759.39484	1.154660654	2.22631951	4.23744504	0.00059355
Spag5	108.150032	1.630221456	3.09560513	4.2344391	0.00060015
Rap2b	511.217873	1.383065858	2.60822053	4.22993583	0.00061017
Smc3	1712.75216	1.382190779	2.60663897	4.22705825	0.0006166
Sec16a	3444.23764	1.080616457	2.11493959	4.22552723	0.00062009
Xrcc6	1119.88496	1.091710409	2.13126562	4.22418585	0.00062308
Entpd1	156.959012	1.285559012	2.43776492	4.22333466	0.00062472
Ptpn23	2049.08393	1.395284224	2.63040368	4.22001888	0.00063253
Las1l	2240.27057	1.098673037	2.14157624	4.2186501	0.00063566
Phf11d	382.569233	1.428456458	2.69158588	4.21817692	0.00063582
Gfm2	3104.69779	1.110838267	2.15971099	4.21807718	0.00063582
Dhx8	762.399466	1.12742323	2.1846819	4.21636939	0.0006392
Clec4a4	27.2462421	2.315646882	4.9782783	4.21352399	0.00064584
Gapt	32.2927887	2.016290908	4.04542397	4.2129663	0.00064612
Serpinb9f	9.61416506	2.304769698	4.94088576	4.20922321	0.00065602
Slfn4	47.0307421	1.822961209	3.53806661	4.20706554	0.00066082
B2m	10313.6408	1.213296685	2.31866868	4.20461653	0.00066651

Ctnnbl1	1061.19116	1.188269886	2.27879301	4.20196668	0.00067209
Nprl2	1981.49306	1.134866751	2.1959828	4.19756236	0.00068375
Adrb2	551.907578	1.527581927	2.88302216	4.19653508	0.00068609
Hmmr	139.870591	1.464513927	2.75970475	4.19482874	0.00069051
Zfp110	843.741435	1.32488131	2.50512277	4.19178744	0.00069827
Acly	5087.23989	1.020377156	2.02844918	4.18914545	0.00070488
Rubcnl	53.4314938	1.946243908	3.85369904	4.18872143	0.00070541
Acot2	4954.0441	1.271992006	2.4149478	4.18430766	0.00071687
Aqr	778.794806	1.126461646	2.18322625	4.18387586	0.00071743
Gm12250	78.4240016	1.608712707	3.04979592	4.18256845	0.00071998
Dhx58	148.495479	1.473842555	2.77760713	4.18276801	0.00071998
Tlr1	58.0908553	1.632726176	3.1009842	4.18224064	0.00072023
H2-K1	4527.68693	1.029873015	2.04184452	4.18185115	0.00072067
Atad2	146.89746	1.322055034	2.50021998	4.17962151	0.00072646
Polr1c	3220.59721	1.087340883	2.12482037	4.17952953	0.00072646
Eya1	8.74879992	2.321718553	4.99927383	4.17910261	0.00072703
Klf13	4184.12034	1.163973214	2.24073683	4.17453678	0.00074014
Cd180	47.5076259	1.942509627	3.843737	4.17299135	0.00074315
Rrm2	583.970744	1.138520401	2.20155121	4.17286469	0.00074315
Dusp6	1358.525	1.064449253	2.09137135	4.17329739	0.00074315
Lrsam1	520.208367	1.112216531	2.16177524	4.17139339	0.00074634
Gm16026	60.5139754	1.58543331	3.00097918	4.16388829	0.00076796
Mthfd1l	142.181642	1.401819755	2.64234667	4.16257297	0.00077073
Bms1	999.008469	1.25336091	2.38396146	4.15985161	0.00077913
Gm4907	19.8879297	2.413628623	5.32812756	4.1580247	0.00078285
Gsdmd	201.056443	1.389569198	2.62000433	4.158232	0.00078285
Akap8l	943.397997	1.135344106	2.19670952	4.15849558	0.00078285
Mex3d	62.2473856	1.769249043	3.40876476	4.15735934	0.00078353
Ighv5-17	87.7259286	1.819582766	3.52979101	4.15165856	0.00079978
Shmt2	10793.7069	1.041547744	2.05843479	4.15209237	0.00079978
Nudcd1	579.458813	1.275414349	2.42068331	4.15029378	0.0008037
Clmn	8593.46975	1.244914816	2.3700456	4.1475209	0.00081262
Bcl2a1c	16.7002141	2.460295824	5.50329561	4.14502449	0.00081978
F13b	529.900612	1.183562333	2.27136936	4.14419328	0.00082188
Plcd4	147.953308	1.478839935	2.78724522	4.1388049	0.00083862
Spi1	67.4344279	1.744783334	3.35144518	4.13731538	0.00084241
Ren1	2676.05615	1.020834793	2.02909272	4.13400685	0.00085014
Ankfy1	1983.33228	1.012484592	2.01738242	4.13077343	0.00085947
Plce1	171.605263	1.331521215	2.516679	4.12959557	0.00086297
Got1	4380.16318	1.035351459	2.0496129	4.12883718	0.00086492
Cd101	25.3218878	2.277979204	4.84998136	4.12755266	0.00086794
Pck2	1128.3021	1.307152681	2.47452682	4.12509363	0.00087544
Abcc3	81.0221407	1.546425954	2.92092629	4.12385904	0.00087923
Slfn1	55.5152374	1.904874253	3.74476258	4.12284803	0.00088126
Sh3tc2	2123.60106	1.097563358	2.13992963	4.12053347	0.00088923
Zfp85	111.867739	1.368467557	2.58196162	4.11833505	0.00089497
Tspyl3	29.6200357	2.05411303	4.15288243	4.11774926	0.00089632
Pcdh15	12.2927725	2.600475856	6.06486636	4.11737695	0.00089684
Vwa8	11632.8019	1.144145209	2.21015141	4.11594643	0.00090148
Cyfip1	1899.90769	1.0825169	2.11772741	4.11524967	0.00090328

Tspan32	67.4997082	1.804330674	3.49267081	4.11037275	0.00092067
Lrp4	522.346388	1.638661044	3.11376711	4.10478287	0.00093672
Tubgcp6	325.0282	1.130269746	2.18899665	4.1022222	0.00094496
Supt16	1096.36203	1.263220789	2.40031008	4.10083447	0.00094968
Ado	2986.80155	1.194147849	2.28809642	4.09704026	0.00096388
Frem2	198.769913	1.378045474	2.59916005	4.09266536	0.00097879
Cdk5rap1	531.86992	1.249238499	2.37715916	4.09206338	0.00098034
Ighv5-4	16.2634703	2.560485698	5.89906251	4.08227795	0.00101535
Gna15	70.2045011	1.823234948	3.53873799	4.07895137	0.00102896
Heatr1	633.927345	1.105455118	2.15166745	4.07608681	0.00103858
4933438K21Rik	16.0778462	2.414719383	5.33215745	4.07584531	0.00103861
Plcl1	592.225972	1.116792087	2.16864226	4.07444672	0.00104383
Ighv2-6	10.3605119	2.108033944	4.31103401	4.07392247	0.0010446
Cmtr2	223.027844	1.377040524	2.59735016	4.07315766	0.00104649
Engase	357.118658	1.226489839	2.33996968	4.06710117	0.00106872
Zfhx3	669.811632	1.206510145	2.30778711	4.06716305	0.00106872
Dus2	735.374388	1.187586498	2.27771383	4.06435196	0.00108032
Aif1	2128.90554	2.201949954	4.60100796	4.06312849	0.00108462
Lrch1	311.618603	1.105051504	2.15106557	4.06296484	0.00108462
Top3a	348.11648	1.187908193	2.27822177	4.06060233	0.00109349
Ifnar2	539.008967	1.177866846	2.26242011	4.05947369	0.00109662
Trerf1	192.34789	1.355387363	2.55865807	4.05525945	0.00111439
Slfn9	209.243203	1.52060795	2.86911928	4.05361478	0.00112005
Ermap	16.2400354	2.36581737	5.15444598	4.0509625	0.00113154
Fignl1	32.0195881	2.056091829	4.15858243	4.05076538	0.00113154
Serpine2	263.618756	1.213059819	2.31828803	4.05054069	0.00113154
Serpina3g	149.485106	1.310514147	2.48029917	4.04624639	0.00114911
Treml4	30.6675486	2.192333822	4.57044241	4.04277191	0.00116288
Egf	12790.5144	1.028127682	2.03937585	4.0424722	0.00116323
Smox	276.447745	1.173941521	2.25627282	4.04198228	0.00116454
Hacd4	177.524847	1.561412572	2.95142682	4.04160272	0.00116529
Hgd	18024.6032	1.121334538	2.17548118	4.03796691	0.0011812
Trim30d	159.026493	1.402328741	2.64327906	4.03430522	0.0011963
Lst1	421.654085	2.201244078	4.59875735	4.03366175	0.00119842
Ccl4	50.0628306	1.962231744	3.89664295	4.03213005	0.0012051
Arl4c	253.371917	1.36166255	2.56981152	4.02305368	0.00124346
Myo9a	1397.88011	1.082324118	2.11744444	4.02078085	0.0012538
Arid3b	219.968883	1.635109275	3.10611076	4.01642219	0.00127238
Tubb2a	1812.9107	1.051088684	2.0720929	4.01634414	0.00127238
Klhl22	2418.13005	1.010733213	2.01493488	4.0154559	0.00127514
Rngtt	564.325482	1.141720594	2.20644012	4.01349158	0.00128336
Ints1	1673.62142	1.256524801	2.38919532	4.01255459	0.00128725
Prdm1	66.0701127	1.873817962	3.6650121	4.01165277	0.00129095
Sipa1l2	359.441961	1.316121954	2.48995894	4.00896816	0.00130448
Uevld	446.947705	1.415573131	2.66765691	4.0062824	0.00131567
Fasn	3390.40245	1.092259	2.1320762	4.00427081	0.00132317
Ms4a4a	39.7001634	2.159811861	4.46856578	4.0034159	0.0013263
Wdr7	1078.31357	1.14370332	2.20947456	4.00326876	0.0013263
Ighv1-76	21.5032488	2.279124368	4.85383265	4.00122771	0.00133549
Sp110	231.818897	1.35503543	2.55803398	4.00097035	0.00133549

P2ry13	20.7414242	2.31927678	4.99081968	4.00031964	0.00133791
Sardh	18708.9643	1.261775267	2.39790627	3.99815024	0.00134897
Abi3	51.593483	1.814566043	3.51753808	3.99688297	0.00135495
Olfr1291-ps1	8.07123428	2.162250708	4.47612619	3.99514798	0.00136237
Gm5431	77.3736179	1.511900554	2.85185485	3.99217423	0.00137829
Ebi3	98.1408437	1.799481877	3.4809519	3.9919125	0.00137853
Cnbd2	7457.19003	1.072589974	2.10320573	3.98919078	0.00139185
Arhgap11a	179.113914	1.28182406	2.43146203	3.98864385	0.00139259
Kdm4c	621.006686	1.172811028	2.2545055	3.98840554	0.00139259
Gm5086	50.5063786	2.086444894	4.24700231	3.98818319	0.00139261
Acadm	39002.5925	1.063722338	2.09031786	3.98725049	0.0013968
Pla2g4a	128.453415	1.292271251	2.44913322	3.98655545	0.0013996
Rad50	976.381205	1.109043972	2.15702661	3.98200004	0.00142408
4930402H24Rik	3354.11888	1.368721703	2.5824165	3.98034961	0.00143269
Wdr61	4716.36019	1.009083279	2.01263182	3.97902828	0.00143935
Zbtb34	281.874276	1.246112194	2.37201346	3.97835677	0.00144209
Znrf3	1033.46242	1.106591888	2.15336352	3.97282141	0.00147016
Gm26880	10479.7826	1.004835741	2.00671501	3.97284909	0.00147016
Klrk1	58.8590768	1.740675361	3.34191574	3.96812925	0.00149305
Mcemp1	29.6648949	1.972518797	3.92452703	3.96582511	0.00150443
Cth	7039.12492	1.01260195	2.01754654	3.96512081	0.00150652
Actl6a	886.55254	1.193916233	2.28772911	3.96330516	0.00151665
Kdm3b	961.885344	1.053279227	2.07524149	3.96243415	0.00152082
Padi2	95.7599818	1.793234449	3.46591062	3.96014431	0.00153408
Gm11224	1106.59508	1.093471262	2.13386848	3.95975879	0.00153517
Efl1	623.973975	1.069704848	2.0990039	3.95818776	0.00154391
Selenon	207.081646	1.533086719	2.89404373	3.95531103	0.00155979
Capn7	2013.60938	1.167045474	2.24551362	3.95495032	0.00156001
Samsn1	40.0603088	2.081342753	4.23200917	3.95434667	0.00156187
Immt	15237.8612	1.004592058	2.00637609	3.95280236	0.00156916
Ighj1	21.8839095	2.192489393	4.57093528	3.95155286	0.00157596
Kyat3	3178.83824	1.016566414	2.02309829	3.95039747	0.00158076
Uhrf1	198.706837	1.413492181	2.66381184	3.94169013	0.00163488
Pik3c2a	1088.87351	1.086049551	2.12291933	3.93911948	0.00165102
Taf7	230.188447	1.060893289	2.08622287	3.93415031	0.00167955
Gm15800	1917.2997	1.101523818	2.1458122	3.93367958	0.00168135
Gm11787	80.3624923	1.39506033	2.6299955	3.93293262	0.00168509
Fam49b	317.621873	1.280652283	2.42948796	3.93098465	0.0016973
Gm9835	170.655568	1.300828267	2.46370286	3.92899999	0.00170834
Son	5041.89262	1.122283833	2.17691312	3.92146713	0.00175953
Mta3	889.03134	1.104090011	2.14963246	3.92096373	0.00176165
Bst2	739.156924	1.172556296	2.25410746	3.92070205	0.00176201
A630001G21Rik	44.544217	1.89963232	3.73118093	3.9192567	0.00177105
Csf3r	63.6817334	1.800943858	3.48448117	3.91340608	0.00180816
AI506816	820.025961	1.103815123	2.14922291	3.91342688	0.00180816
Gm42585	8.684761	2.031609673	4.08860778	3.912723	0.00181169
Lrrc40	686.717843	1.060950013	2.0863049	3.9111267	0.00182052
Hps5	2129.05945	1.02850663	2.03991159	3.90710647	0.00184622
Crlf3	430.015821	1.122234102	2.17683808	3.90275461	0.0018781
Ubr1	991.912328	1.240458497	2.36273609	3.89839177	0.00190226

Setx	1222.70484	1.022925598	2.03203549	3.89355726	0.00193391
Wdr33	1558.25052	1.020171122	2.02815951	3.89108276	0.00195037
2810474O19Rik	2878.41808	1.043659109	2.06144949	3.88837051	0.00197059
Adrb1	71.4454102	1.677845178	3.19949714	3.88304743	0.00200313
Ptpn6	2076.08819	1.141394944	2.20594213	3.88272056	0.00200313
Snx20	61.1062387	1.747238253	3.35715292	3.87851868	0.00202935
Ubr4	5720.82549	1.036773472	2.05163412	3.87632604	0.00204309
Gm42031	10665.9181	1.017185599	2.02396676	3.87535903	0.00204887
8030453O22Rik	14.9425401	2.298495309	4.91944413	3.87389988	0.00205943
Zbp1	209.799376	1.453810134	2.73930543	3.87288243	0.00206279
Gm45191	7.81694456	2.043785034	4.12325885	3.87146298	0.00207133
Kif21b	101.530876	1.606530038	3.04518534	3.86967072	0.00208309
Ptpn14	396.622499	1.032540207	2.04562289	3.86719383	0.00210258
Dhx38	1106.71277	1.024092171	2.03367927	3.8660394	0.00210899
Ighv5-9	14.862332	2.30442242	4.93969656	3.865749	0.00210973
Shq1	286.919679	1.116586314	2.16833297	3.86529447	0.00211188
Kif23	134.643784	1.385382102	2.61241139	3.86156868	0.00213896
Pdzrn4	36.4301759	2.058273755	4.16487661	3.86022186	0.00214538
Tkt	9172.61651	1.151701348	2.22175748	3.86040584	0.00214538
Mov10	903.699819	1.017009869	2.02372025	3.85791923	0.00216207
Spic	29.7231997	2.125569602	4.36375348	3.85631434	0.00217268
Ctps2	1353.92492	1.032682431	2.04582456	3.85651034	0.00217268
Gm6382	464.068608	1.063813357	2.09044974	3.8530614	0.00219626
Rab3b	246.675944	1.139091564	2.20242297	3.85213162	0.00220279
Arhgap45	205.718624	1.36239098	2.57110937	3.85039856	0.00221496
Aldh1b1	177.678971	1.595380146	3.02174129	3.8490919	0.00222475
Gm4873	29.2701725	1.939263766	3.83509886	3.8405416	0.00229798
Ighv1-55	20.7120386	2.225648582	4.67721122	3.84017489	0.00229951
Dhx36	932.848292	1.019579459	2.02732791	3.83739596	0.00231993
Elf4	183.614406	1.319009469	2.49494752	3.83490254	0.00233397
Pygl	549.473849	1.180396268	2.2663902	3.83115632	0.00236591
Nes	302.567496	1.343756513	2.53811337	3.82739565	0.00240037
Stom	186.939497	1.106161265	2.15272087	3.82404359	0.00242928
Lsg1	1059.90011	1.105931153	2.15237753	3.82212961	0.00244422
Ccnd2	1228.89464	1.143433185	2.20906089	3.81608225	0.00249673
Cdc42bpb	1274.74323	1.201955536	2.30051288	3.81576875	0.00249787
Pan2	705.513737	1.041174265	2.05790198	3.8126206	0.0025258
Sec16b	102.989869	1.255826444	2.38803908	3.80905425	0.00255835
Anxa1	265.870268	1.020617622	2.0287873	3.80914588	0.00255835
Maml2	453.895363	1.072680863	2.10333823	3.80858668	0.00256112
Cysltr1	47.5942511	1.79297734	3.465293	3.80789259	0.00256417
Ear10	13.2619565	2.330856664	5.03104001	3.80243937	0.00261265
Gfpt1	1388.5914	1.036525961	2.05128217	3.80225804	0.00261265
Lmbr1l	790.538767	1.106861843	2.15376649	3.79165436	0.0027224
Fam49a	198.864634	1.221796841	2.33237027	3.79100347	0.00272735
Gm13710	36.6068414	2.074960676	4.2133293	3.78915518	0.00274331
Cacna1f	8.33903242	1.909337783	3.75636638	3.78127743	0.0028158
Adamts15	699.943068	1.135384102	2.19677042	3.77799476	0.00284636
Cul7	738.122035	1.164867516	2.24212625	3.76625739	0.00297403
Gm42568	32.8745578	1.879466515	3.67938977	3.76148223	0.00302899

AC153954,1	100.88166	1.428386392	2.69145516	3.76120501	0.00302995
Pex5	2967.85757	1.09282078	2.13290658	3.75559809	0.00309615
Acvrl1	295.342064	1.296515811	2.45634944	3.75285184	0.00311795
Itfg2	742.046838	1.017758178	2.0247702	3.749288	0.00315265
Itga9	391.735777	1.228137342	2.34264337	3.74698512	0.00317424
Haus8	239.748599	1.076416719	2.10879188	3.7459618	0.00318222
Sirpa	1840.40149	1.055235278	2.07805707	3.74274724	0.00321819
Mctp1	26.6503017	1.938997715	3.83439168	3.73860512	0.00326147
Chm	744.296397	1.097207788	2.13940229	3.72672799	0.00340574
Klhl28	424.894291	1.112593558	2.16234026	3.72602626	0.00341258
Rrm1	518.549663	1.125777011	2.18219044	3.7250168	0.00342361
Ptchd1	20.5903869	2.055231517	4.15610331	3.72456963	0.00342703
Icam1	301.701972	1.15401689	2.22532629	3.72329306	0.00343909
Trim6	35.8707053	1.8738736	3.66515345	3.722543	0.00344666
Gdf3	16.8656868	2.149373812	4.43635191	3.7205402	0.00346876
Dpy19l1	203.714479	1.181202446	2.26765701	3.72019071	0.00347078
Kif1b	3218.31063	1.015337045	2.02137508	3.7200038	0.00347078
Nup37	378.959889	1.07738021	2.11020069	3.71705232	0.00350616
Gmfg	715.140398	1.089821283	2.12847668	3.71658488	0.00350726
Mtr	1510.43257	1.054982663	2.07769324	3.71676397	0.00350726
Tgs1	827.542687	1.099631278	2.14299915	3.71618087	0.00351018
Plch1	69.0842854	1.415199368	2.66696588	3.71574652	0.00351083
Tmem2	1049.9205	1.115133904	2.16615113	3.71591108	0.00351083
Hectd3	1509.61726	1.041738961	2.05870763	3.7131321	0.00354189
Nlrp3	18.2013492	2.055666278	4.15735596	3.7124438	0.00354634
Ikzf1	287.39343	1.102727567	2.14760336	3.71242781	0.00354634
Gm18860	275.286581	1.004937499	2.00685655	3.70503982	0.00363467
Rad21	5418.66008	1.022380723	2.03126818	3.70297068	0.00365612
Vps11	2446.17708	1.009075091	2.0126204	3.69917099	0.00370287
Polr3b	735.583989	1.018556382	2.02589076	3.69752636	0.0037185
Ttc21b	473.679408	1.022487865	2.03141903	3.69296682	0.00377728
Cnnm4	1317.25214	1.155626242	2.22781007	3.68736256	0.00385561
Rnf40	1865.31764	1.031791939	2.04456218	3.68575962	0.00387123
Slc9a9	65.0685974	1.907714	3.75214089	3.67993004	0.00395192
Ighv5-12	11.3417721	2.095200192	4.2728545	3.67607054	0.00400329
Nlrx1	629.031281	1.060259944	2.08530722	3.67605945	0.00400329
Emilin1	489.093671	1.263263681	2.40038144	3.67414106	0.00402152
Paf1	1767.28981	1.022161714	2.03095984	3.67202233	0.004052
Fbxw8	1468.69375	1.000309855	2.0004296	3.67172209	0.00405375
Marcksl1	279.549068	1.269062901	2.41004971	3.67094258	0.00406312
Helb	477.390332	1.043829427	2.06169287	3.6692315	0.00408434
Pik3c2b	180.28308	1.279391442	2.42736564	3.66386333	0.00415553
Lgals9	597.881327	1.155740848	2.22798705	3.66173624	0.00418092
Myom1	55.8338721	1.519523802	2.86696403	3.65978561	0.00420358
Ppp2r3a	3551.60052	1.028060698	2.03928116	3.65791689	0.00422811
Ripk3	85.9573236	1.461542756	2.7540271	3.65509166	0.00427181
Adgrl3	13.5944209	2.15417494	4.45114019	3.65181198	0.00431408
AC116589,3	9.59577201	2.218726605	4.65482395	3.65116512	0.0043218
H2-M2	76.4908496	1.308360784	2.47659984	3.65060843	0.00432801
Dennd2c	78.0416731	1.492765635	2.81427953	3.64740275	0.00437797

Nadsyn1	1377.92406	1.020541498	2.02868026	3.64447884	0.00441633
2010008C14Rik	7.91324269	1.789245155	3.45634003	3.64399308	0.00442146
Olfr57	6.47049773	1.900212051	3.73268056	3.64357426	0.00442544
Usp11	170.916449	1.043955576	2.06187315	3.63951668	0.00448923
Rnf213	1619.43202	1.077779482	2.11078477	3.6390952	0.00449332
Gm5640	19.9458542	2.042762299	4.12033688	3.63669609	0.00452533
Ap3m1-ps	187.114142	1.05358068	2.07567516	3.63622198	0.00452562
Clp1	708.475832	1.046704408	2.06580547	3.63613023	0.00452562
Slc2a3	37.5366613	1.887894131	3.70094612	3.63391119	0.00456145
Tubgcp5	326.550589	1.156605158	2.22932222	3.62620203	0.00466605
Aim2	43.1347727	1.550826518	2.92984942	3.62181298	0.00473915
Rps6ka5	83.9805081	1.412608192	2.66218013	3.62133051	0.00474122
Mefv	9.14383477	2.198652897	4.59050508	3.61817808	0.00478906
Polr3a	710.898205	1.054215671	2.07658895	3.61158476	0.00488463
Depdc1a	18.8151031	2.084468544	4.24118832	3.61008025	0.00490262
Chd7	398.422271	1.025936477	2.03628074	3.60942977	0.00490639
Fermt1	1396.11758	1.095440814	2.13678361	3.60830452	0.0049202
Sh3pxd2b	249.399876	1.198496284	2.29500339	3.60153891	0.00503097
Acox3	12210.5766	1.015006955	2.02091263	3.60013014	0.00504766
Gm16421	37.3348677	1.715516577	3.28414216	3.59684416	0.00510031
Lipt1	283.565447	1.197058218	2.29271689	3.59670263	0.00510031
Gm43715	11.0469457	2.251026102	4.76021291	3.5958247	0.00511397
Dnmt3a	1653.69509	1.004467139	2.00620237	3.59430821	0.00513666
Akna	283.370441	1.291434589	2.44771331	3.5912961	0.00518555
Lig1	261.4811	1.203410325	2.30283385	3.58954858	0.00521317
Galnt12	62.3502891	1.755024812	3.37532123	3.58633374	0.0052705
Cmtm3	403.789143	1.194754913	2.28905942	3.58589091	0.00527579
AC186674,1	6.34391881	1.8474855	3.5987241	3.58356823	0.00531557
Apobr	62.4434558	1.606751219	3.04565224	3.58186712	0.00534291
Fam46a	1144.19166	1.150287757	2.21958161	3.57988386	0.00536876
Napepld	265.652459	1.087922935	2.1256778	3.57899561	0.00538333
Aim1l	71.7597441	1.671734711	3.18597448	3.57832491	0.00538601
Gm15544	70.5266351	1.430225647	2.69488862	3.57792493	0.00539055
Tbc1d31	196.452844	1.134529365	2.19546931	3.57649904	0.00541258
Gm14221	14.3288446	2.100107362	4.2874129	3.57297439	0.00546347
Foxp2	14.6617681	2.073430703	4.20886345	3.56404719	0.00560298
Mcm3	340.22293	1.422245263	2.68002278	3.56381108	0.00560422
Gse1	243.981709	1.202968973	2.30212947	3.56310959	0.00561542
Adam8	32.855634	1.707741259	3.26649008	3.56143822	0.00564747
Pygm	159.765933	1.336299074	2.52502745	3.55428323	0.00578771
Casp12	50.0437329	1.594300073	3.01947991	3.54786107	0.0059187
Hdac4	1183.65689	1.065557165	2.09297803	3.54657149	0.00594374
Esyt1	232.894662	1.181064208	2.26743974	3.54497094	0.00597018
Klrb1b	20.2750081	1.959179768	3.88840844	3.54464357	0.00597131
Ehd4	1057.21231	1.039598506	2.0556555	3.54366317	0.00598954
Slfn8	280.910776	1.225193471	2.33786799	3.54104392	0.00604414
Gpd2	3179.37362	1.093366584	2.13371366	3.53538922	0.006135
Pnpt1	676.692436	1.008514729	2.01183882	3.53553871	0.006135
Vprbp	871.473567	1.004813308	2.00668381	3.5339883	0.00616351
Cuedc1	688.81298	1.132559903	2.19247426	3.53300911	0.00618227

Gm10440	8.48134701	2.138505116	4.40305576	3.53279444	0.00618318
Ighv1-62-2	19.3505629	1.954658069	3.87624046	3.53111876	0.00621423
Elac1	268.892333	1.021749121	2.0303791	3.5279259	0.00626887
Lrfn5	11.0899244	2.158662784	4.46500807	3.52706892	0.00628073
Ccdc126	215.375375	1.30537997	2.47148812	3.52640825	0.00628412
Fpr1	7.51510298	1.691120094	3.22907308	3.5249936	0.00630531
Gm4157	37.0878839	1.756679679	3.37919517	3.52357033	0.00632678
Szt2	796.168938	1.174559068	2.25723882	3.52242887	0.00634601
Arhgap23	185.370486	1.112042608	2.16151464	3.52042921	0.00637119
Csad	27938.7478	1.000811804	2.00112572	3.51961385	0.00637337
Rgsl1	6.38667393	1.770871945	3.41260147	3.5144916	0.00646365
Cspg4	466.099973	1.113539151	2.163758	3.51449098	0.00646365
Exph5	242.609704	1.047690879	2.06721849	3.51260174	0.0065041
Ighv2-9-1	10.7613183	2.175274292	4.51671628	3.50986866	0.00655857
Ier5	259.884746	1.263720143	2.40114103	3.5083824	0.00658256
Pald1	189.956108	1.00140537	2.00194921	3.50361342	0.00666288
Gm28875	866.116319	1.366092858	2.57771517	3.50057093	0.00672646
Fam26f	35.1420722	1.740531227	3.34158188	3.49921174	0.0067436
Diaph1	1218.48972	1.222297865	2.33318041	3.49944	0.0067436
Atad2b	418.057125	1.003188962	2.00442573	3.49817105	0.00676562
Espl1	54.7839329	1.619651774	3.07300854	3.49693065	0.00678908
Ube3a	2285.96453	1.109192027	2.15724798	3.49690774	0.00678908
Dpep2	24.7143379	1.812014809	3.51132323	3.49415484	0.00683768
Arsa	159.93536	1.048923695	2.06898573	3.49375474	0.00684359
Tbc1d4	351.020845	1.001332623	2.00184826	3.49332933	0.00685015
Pold1	294.908372	1.014870159	2.02072102	3.48653763	0.00700425
Gldc	2215.8288	1.176088346	2.2596328	3.48278897	0.00708961
Gm19617	709.670958	1.025813278	2.03610686	3.48094004	0.00711627
Gm15922	64.4560697	1.610332592	3.05322221	3.47964972	0.00714163
Rasgrp4	23.6799907	1.810295529	3.50714123	3.47910939	0.00714553
Pole	80.2496912	1.312533011	2.48377246	3.4790823	0.00714553
Irak4	1095.94941	1.27133416	2.41384687	3.47899882	0.00714553
Ighv9-1	14.6615001	1.987293334	3.96492433	3.4787802	0.00714688
Kif9	252.756379	1.211762857	2.31620486	3.47725128	0.00718326
Sbno1	4027.07215	1.080448324	2.11469313	3.47666342	0.00719001
Cfhr3	22.8920075	1.806528386	3.49799539	3.47512635	0.0072223
Exd1	59.2212208	1.414798302	2.66622457	3.47263543	0.00728056
Tap1	340.448997	1.017291369	2.02411515	3.47101002	0.00731564
Pld5	5.85427771	1.766241561	3.40166616	3.4659928	0.00743498
Gm18445	12.2415986	2.018815543	4.05250943	3.46517477	0.007453
Pld2	241.410711	1.203172305	2.30245395	3.46423695	0.00747439
Ighv8-12	33.7652547	1.144817831	2.21118208	3.46204597	0.00752615
Nkrf	149.811817	1.342168175	2.53532057	3.46183638	0.00752734
Igkv3-12	19.6452032	1.911890857	3.76301974	3.46150247	0.00753027
Scn2b	77.9350916	1.406675278	2.65125471	3.46094198	0.00753838
Pou5f2	8.34097435	2.057178584	4.16171619	3.45635328	0.00763482
E430014B02Rik	6.2011029	1.724680687	3.30506968	3.45414592	0.00769158
Ercc2	166.103063	1.039491351	2.05550282	3.4538583	0.00769158
Gm5936	20.8540828	1.915793705	3.77321342	3.44949614	0.00777866
Nrp1	2558.15322	1.122354079	2.17701912	3.44881206	0.00779362

Bbc3	29.8397106	1.636258719	3.10858649	3.44431179	0.00788593
Abca9	119.688407	1.31349439	2.48542814	3.43937588	0.00801164
Usp29	8.92343624	1.997048938	3.99182628	3.43738301	0.00805615
Tmem156	8.17314802	1.99609863	3.98919772	3.43334377	0.00815243
Cpne8	175.089445	1.250588536	2.37938468	3.42729956	0.00830098
Ighv1-71	17.9352452	1.897538533	3.72576979	3.42601697	0.00833415
Kntc1	87.1603517	1.204244477	2.30416571	3.41364019	0.00863496
Gm33370	6.93873001	1.61981351	3.07335306	3.41288945	0.00864653
Lilra6	39.341621	1.709153425	3.26968901	3.41215099	0.00865138
Rftn1	164.172494	1.330523419	2.51493902	3.40965833	0.00870501
Gm5118	5.76776062	1.713718059	3.28005057	3.40741497	0.00875617
Stk10	162.308775	1.277172014	2.42363428	3.40034505	0.00893911
AC155241,2	419.910369	1.010410201	2.0144838	3.40032033	0.00893911
Efna5	359.138083	1.435329424	2.70443913	3.3998683	0.00894676
Ch25h	99.8608039	1.578584005	2.98676557	3.39763126	0.00900629
Wif1	16.3360082	1.897768954	3.7263649	3.39738737	0.00900904
Slfn5	693.313929	1.022755537	2.03179597	3.39568056	0.00904228
4921527H02Rik	5.84289998	1.689235086	3.22485677	3.38988086	0.00921623
Brca2	182.506636	1.004964264	2.00689379	3.38838684	0.00924481
Creb5	96.1924969	1.347408089	2.54454568	3.38517779	0.00931054
AC153729,1	6.29874767	1.64371597	3.12469629	3.38483739	0.0093167
Prkdc	950.625992	1.083762207	2.11955618	3.38220414	0.00938478
Gm26510	39.2453289	1.630884222	3.09702756	3.37709677	0.0095016
Ddx3y	1382.64065	1.024685087	2.03451524	3.37748179	0.0095016
Iars2	3156.21923	1.088566402	2.1266261	3.37211948	0.00964074
Slc38a1	50.8176538	1.3324316	2.51826761	3.37055247	0.00966275
Glud-ps	1077.5136	1.060824026	2.08612271	3.37054403	0.00966275
Ppp2r2a	1408.30906	1.009530164	2.01325534	3.36979067	0.00967836
Ighv10-3	8.00907555	1.931160874	3.81361941	3.36942968	0.00968029
Guca1a	59.2692234	1.360109678	2.56704694	3.36536138	0.0097561
Cfhr2	209.9198	1.104589624	2.15037702	3.36081119	0.00989726
Zic4	8.25987913	1.94592334	3.85284284	3.35727005	0.00999117
Ets1	670.524903	1.119483609	2.1726919	3.35286524	0.0101198
5031425F14Rik	11.8991357	1.990334666	3.97329157	3.35125317	0.01015588
C230071H17Rik	14.2595885	1.951523337	3.86782719	3.35119845	0.01015588
Ighv4-2	11.2440874	1.99569066	3.9880698	3.3501683	0.01018237
Nphp3	335.142517	1.029766278	2.04169346	3.34445377	0.01034835
Stat1	724.068676	1.000886263	2.001229	3.341658	0.01043216
Epg5	674.231846	1.051718747	2.07299803	3.33540117	0.01060885
Zcchc24	177.137132	1.127331262	2.18454263	3.33388941	0.01066081
Gm15964	11.0200393	1.989717957	3.97159347	3.32651146	0.01086924
Pprc1	492.170214	1.017573559	2.02451111	3.32612365	0.01086924
Gm19938	5.36949587	1.662074196	3.16471196	3.32165393	0.01099429
Cd93	603.427089	1.018651551	2.0260244	3.32085039	0.01101404
Cd44	77.8651343	1.557177898	2.94277634	3.31965364	0.01104937
Ighj4	9.65016035	2.021250886	4.05935605	3.31502798	0.01117225
Gm11397	7.72973862	1.933116412	3.81879218	3.31475372	0.01117225
Ear-ps2	10.4768418	1.983058058	3.95330169	3.31285184	0.01121394
Gm10552	16.1680985	1.823623941	3.53969227	3.30601523	0.01145261
Neurl1a	36.0537554	1.539812195	2.90756651	3.3049095	0.0114856

Gm45697	5.45472883	1.636117678	3.1082826	3.30365192	0.01153109
Slc41a3	79.5510705	1.224146753	2.33617242	3.29877949	0.0117081
H2-M3	205.590717	1.194610106	2.28882967	3.29797239	0.01173555
Gm13205	78.4134916	1.400379381	2.63970989	3.29506507	0.01183244
Cdc25c	22.4605539	1.719491712	3.29320361	3.29147304	0.01193601
Rnf122	176.481714	1.091053977	2.13029611	3.28911754	0.01200244
Ccr1	22.766839	1.84611984	3.59531915	3.28617228	0.01208401
Olfr1205	9.76106228	1.987625823	3.96583821	3.28486855	0.01211459
Gm18360	5.77650756	1.586000893	3.00216005	3.2830739	0.01217282
Dsc2	44.9616344	1.574390321	2.97809613	3.28088894	0.01224826
Ntrk3	37.5054359	1.597858298	3.02693627	3.27807752	0.01233864
H2-Oa	16.8391445	1.830326448	3.55617531	3.27636852	0.01238993
Siglech	22.9138677	1.838528101	3.57644958	3.2739639	0.01244824
Gm4117	18.1586128	1.819206131	3.52886963	3.27084987	0.01256657
Isg15	221.303308	1.082692971	2.11798588	3.26985468	0.01257829
Pkib	115.562484	1.356051878	2.55983688	3.26597478	0.01268638
Olfr816	5.30808775	1.608446622	3.04923348	3.26264409	0.01278386
Top2a	334.685723	1.006020103	2.00836307	3.25406516	0.01311586
Ighv5-16	11.8343361	1.884810275	3.69304355	3.24825253	0.01332568
Cnr1	5.23307341	1.599908246	3.03124034	3.24735494	0.0133574
Actn3	24.1106024	1.821974232	3.53564697	3.24522383	0.01343408
Dbt	4041.41692	1.020189486	2.02818533	3.24269535	0.01351977
Strip2	150.370828	1.091884393	2.13152266	3.24050001	0.01360371
Nsun6	155.568141	1.029850397	2.04181251	3.23674767	0.01375613
Ank	135.932837	1.153552488	2.22461008	3.23654508	0.01375898
Tnip3	21.3208122	1.728616132	3.31409769	3.23336114	0.0138784
Ighv1-62	12.3537623	1.884714833	3.69279924	3.23156435	0.013938
Btbd9	1740.29254	1.126149937	2.18275459	3.23020871	0.01399026
Adam23	23.2512424	1.749298177	3.36194979	3.22745114	0.01407657
Acvr2b	418.592842	1.092618252	2.13260718	3.22756469	0.01407657
Pik3r4	944.780923	1.016532675	2.02305098	3.2258126	0.01415035
Iffo2	142.276388	1.015549874	2.02167329	3.22430913	0.01418955
Gys2	501.162121	1.135574228	2.19705994	3.22402621	0.01419653
Asgr2	25.5567988	1.825529573	3.54437088	3.22009184	0.0143359
Zfp282	390.981391	1.068842302	2.09774934	3.21787968	0.01442553
Lama4	177.029306	1.235881729	2.35525249	3.21691577	0.01446694
Cct3-ps1	97.5404918	1.106974717	2.153935	3.21343459	0.01460743
Ighmbp2	207.650031	1.048540534	2.06843631	3.20905994	0.01477335
Slco2b1	471.027373	1.022552355	2.03150984	3.20636728	0.01489041
Prokr1	9.09694146	1.918861395	3.78124517	3.20376193	0.01500804
Fam196a	24.2193216	1.724121831	3.30378964	3.20053638	0.01513574
Ighv1-26	17.2317855	1.810033715	3.50650483	3.19978803	0.0151677
Ighv1-82	11.6278528	1.85421453	3.6155485	3.19922744	0.01518243
Cep192	225.444444	1.126447748	2.18320522	3.19924138	0.01518243
Timd4	5.46732819	1.525234476	2.87833493	3.19619403	0.01532059
Il1rl2	36.8509826	1.381365833	2.6051489	3.19060295	0.01552205
Cd300lf	21.9263013	1.682100158	3.20894744	3.18923056	0.01558088
Kcnq1ot1	194.720174	1.083739796	2.11952326	3.18858059	0.01560089
Il6ra	124.564983	1.23808291	2.35884874	3.18789362	0.01562294
Emilin2	32.3624194	1.687923904	3.22192722	3.18443454	0.0157653

Ddx11	87.1539784	1.427469289	2.68974478	3.18230375	0.0158589
Capn8	5.04765463	1.546642291	2.92136433	3.17685616	0.01609805
Nrm	83.8166548	1.251778402	2.3813479	3.17409626	0.01619774
Agmo	22.1098916	1.727841494	3.31231871	3.17169507	0.01629347
Dtl	54.0122579	1.409188472	2.65587725	3.17071551	0.0163175
Cxcr4	41.3491504	1.398764451	2.63675669	3.17092385	0.0163175
Ankle1	13.194237	1.801803699	3.48655853	3.16964795	0.0163543
Clec4n	50.9173024	1.486901986	2.80286447	3.16827213	0.01639309
Gm43728	4.87480798	1.555254037	2.93885471	3.16770595	0.01641729
Gm45179	9.49793969	1.639511982	3.11560423	3.16389121	0.0165714
C130073E24Rik	8.78867919	1.894126439	3.71696844	3.16349076	0.01658642
38961	322.203755	1.00716377	2.00995579	3.16241723	0.01663987
Magee1	97.1525105	1.244080943	2.36867612	3.15854312	0.0168231
Cep290	626.142606	1.128937598	2.18697632	3.15418765	0.01701123
Gm21188	9.34852907	1.863634296	3.63923268	3.15241397	0.01708961
Atp8a1	242.702313	1.010171695	2.01415079	3.15089551	0.01711748
Nav3	27.8523316	1.523113805	2.87410706	3.15060733	0.01712634
Fam43a	161.868809	1.074222654	2.10558724	3.14635575	0.01731295
Ifi47	354.960919	1.252775216	2.38299383	3.14039623	0.0175877
AI662270	141.766627	1.383630402	2.60924136	3.13892655	0.01766801
Dstyk	943.661895	1.138254766	2.20114588	3.13827913	0.01768266
P2rx7	99.9062548	1.158008146	2.23149124	3.13538514	0.01779261
Hpse	13.447812	1.797822604	3.47695068	3.13489173	0.01781441
Cadm1	753.462855	1.018558152	2.02589324	3.13424299	0.01783304
Amer1	481.675182	1.026692405	2.03734797	3.13091907	0.01795031
Mx1	49.6819679	1.511854359	2.85176353	3.1307045	0.01795525
Eif3a	7527.43828	1.031198143	2.04372084	3.12977491	0.01799579
Pgbd5	12.1664097	1.824947312	3.54294068	3.1275139	0.01810874
Lilrb4a	33.2611887	1.499477148	2.82740225	3.11884876	0.01850007
Gli3	24.0601377	1.614090341	3.06118522	3.11296971	0.01881074
Ighv8-8	5.0700703	1.479873553	2.78924286	3.11085255	0.01891474
9330162B11Rik	5.22438897	1.462761305	2.75635423	3.11014892	0.01893803
Kank4	61.9587333	1.260867852	2.39639853	3.11000927	0.01893803
Gm44291	16.1204975	1.703740852	3.25744507	3.10557036	0.01909916
Adgrb3	8.84629819	1.817216642	3.52400664	3.10451022	0.01913638
Ighv5-9-1	21.5555133	1.750331135	3.36435778	3.09819927	0.01945371
Tpbgl	46.971429	1.474417923	2.7787151	3.09828013	0.01945371
Pou1f1	5.13270005	1.450961113	2.73390121	3.08885657	0.01989639
Cd300ld4	15.5006295	1.737556863	3.33469973	3.08806391	0.01992726
Cd300lb	16.5186392	1.762482025	3.39281326	3.08585116	0.01998879
Stxbp3-ps	156.14019	1.042444179	2.05971422	3.08039636	0.02030187
Mtbp	111.066462	1.036579769	2.05135868	3.07652716	0.02042908
Adgre4	8.96924427	1.838030969	3.57521739	3.07633711	0.0204333
Gm18752	22.6049549	1.632155916	3.09975871	3.07342938	0.02058913
A430093F15Rik	8.8483291	1.818797605	3.5278705	3.07053557	0.02075112
Ighg2b	835.252728	1.705375591	3.26113822	3.06785211	0.02088749
Slc38a11	6.63400714	1.684444101	3.21416524	3.06622943	0.02098323
Ear1	12.6109201	1.764467543	3.39748585	3.06261962	0.02118362
Pparg	34.7793897	1.691935396	3.23089843	3.06187469	0.0212183
A2ml1	4.92361675	1.444258663	2.72122955	3.05987389	0.02130607

Inafm2	2189.87956	1.088188782	2.12606953	3.05765943	0.0214459
Olfr1279	549.638559	1.057786674	2.08173535	3.05723366	0.02145815
Gm44224	9.05429416	1.818755028	3.52776639	3.05597014	0.02150315
Clec4e	5.1802872	1.413862765	2.66449618	3.05489529	0.02155297
Gm37629	4.86355527	1.443021018	2.71889609	3.05259962	0.0216544
Sh3kbp1	222.11852	1.023974334	2.03351317	3.0500009	0.02177836
Fetub	77.2513803	1.246243901	2.37223001	3.04897429	0.02182538
Gpr18	21.6593126	1.717962188	3.28971405	3.04688037	0.02191255
Hdac8	346.584341	1.046878909	2.06605536	3.04544927	0.02195377
Ptprz1	14.2307174	1.762591226	3.39307008	3.03882667	0.02233955
Gm42888	14.5347195	1.749698919	3.36288377	3.0367874	0.0224539
Gm37877	6.45831059	1.7164554	3.28627998	3.03387831	0.02261524
Batf3	132.069046	1.104242445	2.1498596	3.03335088	0.02262444
Gabra2	8.18779267	1.826575538	3.54694151	3.02971521	0.02280657
Ttc21a	9.1224574	1.787344706	3.45179002	3.02825681	0.02286595
Cd300ld5	16.9816244	1.699380039	3.24761371	3.02787004	0.02286595
Rasal3	35.1089074	1.440128111	2.7134496	3.02554749	0.02298273
Dnah8	17.6796663	1.629362043	3.09376163	3.02303538	0.02313511
Nwd2	84.8398124	1.473928665	2.77777292	3.02219548	0.02318993
Stag3	7.37775042	1.573174022	2.97558643	3.01847721	0.02339045
Rbl1	136.842545	1.116169431	2.1677065	3.01827481	0.02339656
Pla2g2d	33.7477779	1.640096458	3.1168667	3.01328595	0.02367874
Atg9b	13.9256239	1.737226998	3.33393736	3.0097041	0.02387611
Kif26a	87.1218433	1.011773995	2.01638901	3.00631255	0.02407271
A730062M13Rik	4.72337014	1.408820747	2.65520039	3.00340106	0.02427494
Gm21370	8.35780662	1.761197581	3.38979395	3.00212611	0.02434749
Gm4764	22.4155942	1.69240052	3.23194024	2.99927139	0.02451777
Ank2	123.055891	1.033024318	2.04630943	2.99649678	0.02463353
AI467606	55.7997646	1.211966398	2.31653166	2.98809233	0.02513086
Gm26624	22.9317305	1.594203706	3.01927822	2.98762778	0.02515913
Dnaaf3	22.5299006	1.602987907	3.03771792	2.98630426	0.02521843
Arhgap9	103.949423	1.264925357	2.40314776	2.9857429	0.02524635
Thbd	195.41524	1.067762787	2.09618026	2.98320095	0.02542539
Hp	13.3129514	1.733074051	3.32435407	2.98236011	0.02547527
Slfn10-ps	100.603245	1.115112477	2.16611896	2.97310132	0.02611264
Nrep	176.850001	1.037839244	2.0531503	2.96986782	0.02636843
Ophn1	136.788218	1.097241451	2.13945221	2.96934072	0.02639306
Iqgap3	51.1923535	1.36471993	2.57526328	2.96609787	0.02661057
Gm44436	4.57788816	1.384823294	2.6113997	2.96341678	0.02678084
Gm15753	55.7004479	1.178835965	2.26394038	2.96078466	0.02689915
Maats1	9.32546877	1.715264837	3.28356915	2.95431425	0.02729699
Ust	31.928384	1.441939395	2.71685844	2.94835442	0.02763313
AC133079,1	4.51021161	1.373661476	2.59127382	2.94335669	0.02794865
Brinp3	6.13264417	1.636258633	3.1085863	2.93860521	0.02829456
Rnf144b	92.1480835	1.121728346	2.1760751	2.93723177	0.02838789
M1ap	27.2374009	1.4911587	2.81114662	2.93623936	0.02845732
Eme1	16.1991889	1.655761159	3.15089386	2.92624422	0.02910149
Cd1d1	62.6782572	1.300869926	2.463774	2.92325307	0.02933855
Cpne2	134.389192	1.000905035	2.00125504	2.91976729	0.02959118
Gm15925	35.3773088	1.498497094	2.82548219	2.91858615	0.02965814

Grid2	7.01001761	1.755664923	3.37681917	2.9172687	0.02972941
Gm37868	7.48285801	1.451182821	2.73432138	2.91582475	0.02981189
Fam212a	96.1226056	1.155479978	2.22758422	2.91376771	0.02994231
Tbc1d9	111.334213	1.082762771	2.11808835	2.91328928	0.02995141
Cd300ld3	17.2508367	1.636047275	3.10813092	2.91099876	0.03010854
Gm15523	16.0562663	1.633522544	3.10269642	2.91120364	0.03010854
Gm10693	18.541282	1.558541022	2.94555813	2.91075966	0.03011114
Ccdc88c	250.577357	1.034227782	2.04801713	2.91016506	0.03011114
Nectin4	71.3648173	1.311735548	2.48239991	2.91003091	0.03011298
AC134249,1	4.2121952	1.366577701	2.5785816	2.90169366	0.03068377
Klhl13	142.74504	1.006425122	2.00892697	2.89958114	0.03080632
Dcaf12l1	230.010453	1.123744601	2.17911842	2.89186275	0.03135632
Myc	244.951039	1.06184344	2.0875973	2.88885265	0.03155529
Zfp712	30.1128135	1.389269697	2.61946048	2.88696343	0.03165406
Cdh23	18.3208401	1.571047136	2.97120291	2.88592767	0.03170142
Trim30e-ps1	4.30874338	1.339669592	2.53093348	2.88525163	0.03174432
Zfp382	57.4507213	1.198986015	2.29578257	2.88515102	0.03174432
C130026I21Rik	60.6405129	1.251257659	2.3804885	2.88482467	0.0317557
Psmb8	1324.53592	1.134492206	2.19541276	2.88270883	0.03194489
Ap4e1	187.783819	1.054797981	2.07742729	2.88021238	0.03210898
Zc3h12d	261.691035	1.065049339	2.09224143	2.87566156	0.03248276
Rad51	42.1551826	1.31267127	2.4840105	2.86674373	0.03318282
Ighv1-62-3	13.346066	1.606299096	3.04469792	2.86388965	0.03342449
Gm43027	6.48201039	1.521014992	2.86992889	2.86088478	0.03367631
Gcm1	23.2297128	1.532294246	2.89245446	2.8591718	0.03382297
Gm17575	7.19026904	1.68093009	3.20634594	2.85551658	0.03415467
Platr30	7.19026904	1.68093009	3.20634594	2.85551658	0.03415467
Igkv15-103	16.0512195	1.603161022	3.03808245	2.85027163	0.03457731
S100a4	19.2352866	1.533508512	2.89488996	2.84920924	0.03465662
Luzp2	7.82666187	1.389970613	2.62073342	2.8464761	0.03485809
Atp1a3	8.05770624	1.652956875	3.14477516	2.84561937	0.0349155
Dna2	217.312398	1.042528757	2.05983497	2.83985082	0.03538074
Socs3	72.5216682	1.27291202	2.41648832	2.8377216	0.03554373
Olfr1440	4.15200869	1.305299331	2.47134998	2.83054802	0.03605159
Abcd2	17.0713064	1.619475215	3.07263248	2.82931054	0.03615207
Cd300ld2	9.51192303	1.584472304	2.99898084	2.82682787	0.03632364
Gm17944	4.12326209	1.300898389	2.46382261	2.82330412	0.03660036
Gm43473	5.93239309	1.517364577	2.86267636	2.82185992	0.03671561
Batf2	64.6552084	1.096699297	2.13864837	2.82055119	0.0367722
Slamf6	8.82342652	1.640314864	3.1173386	2.81395786	0.0373229
Gm45414	4.10191579	1.294474263	2.45287593	2.81328795	0.03736608
Cdk1	150.933802	1.077067554	2.10974342	2.81231523	0.03744435
Tlr6	57.8663817	1.090519894	2.12950762	2.80930584	0.03772983
Rad54l	58.0471239	1.296041684	2.45554232	2.80846653	0.03781562
Gm15128	4.020508	1.297918691	2.45873916	2.80656451	0.03793771
Gm37706	8.55847491	1.647202179	3.13225611	2.80342733	0.03823197
Reln	33.3176775	1.431876287	2.6979737	2.80071179	0.03847801
Nuf2	44.7510015	1.352989343	2.55440865	2.79974038	0.03856238
Casc1	50.7459921	1.219646443	2.32889637	2.79933451	0.03857667
Angpt4	9.39255623	1.565184537	2.9591535	2.79639833	0.03881421

Olfr716	4.02690145	1.286016258	2.43853767	2.79552244	0.03886794
Gm37151	4.04696362	1.281942291	2.4316613	2.79359785	0.03906115
Gm35570	81.8092598	1.077671578	2.11062691	2.79299238	0.03910844
Gm11218	4.37742678	1.241967341	2.36520846	2.7895011	0.03944124
Gm45292	7.00261284	1.623658793	3.08155554	2.78711092	0.039675
Gm45910	73.9821332	1.153590575	2.22466881	2.78521835	0.0398345
Gm7823	8.47202032	1.635589853	3.10714561	2.78419629	0.03993134
Ighv1-81	27.042593	1.532184313	2.89223407	2.78404427	0.03993134
Ighv6-3	9.04131034	1.609456029	3.05136768	2.78257246	0.04008163
3110080O07Rik	7.30991344	1.646366325	3.1304419	2.77817964	0.04049459
Stx11	34.6802568	1.355587447	2.55901295	2.7775411	0.0405477
Hs3st3a1	49.675279	1.229156826	2.34429939	2.77323852	0.04095379
Il1rn	19.9282087	1.56652348	2.96190112	2.76987821	0.04133825
Gm15107	3.91405376	1.274184334	2.41862036	2.76779015	0.04150937
Dscc1	17.6906764	1.590965599	3.0125091	2.7663218	0.04160212
Olfr1090	4.9548066	1.15913224	2.23323062	2.76642071	0.04160212
Plcl2	326.177791	1.150565426	2.22000885	2.76563204	0.04165808
Aox1	16.9516938	1.511672141	2.85140337	2.76267832	0.04193335
Cxcl3	3.85903908	1.272361551	2.41556647	2.75914297	0.04223956
Gm5996	17.3387291	1.501508999	2.83138709	2.75561778	0.04258164
Gm18840	18.9851309	1.485381166	2.79991138	2.75457404	0.04264162
Gm43845	3.79891509	1.27435085	2.41889953	2.75381326	0.04269289
Olfr128	3.79885258	1.274334437	2.41887201	2.75376134	0.04269289
Wdr93	109.82285	1.123340905	2.17850875	2.75106671	0.04292198
Gm38259	4.03194825	1.241604498	2.36461367	2.74890464	0.04312351
Abcb4	17.304976	1.538902865	2.90573445	2.7464376	0.04339386
Gm9540	19.6208171	1.466773458	2.76403035	2.74578643	0.0434386
Mb21d1	69.148071	1.269872359	2.4114023	2.73739446	0.04426697
Ighv1-18	13.090035	1.579092852	2.9878192	2.73523244	0.04448853
Kif14	17.0074839	1.499547645	2.82754042	2.73500047	0.04450586
Elovl4	4.96355355	1.129142177	2.18728646	2.73424429	0.04455203
Klk7	3.82900834	1.251233972	2.38044941	2.73286805	0.04467497
Gm42482	5.17699927	1.496586129	2.82174209	2.7315638	0.04480322
Cfap46	7.8953874	1.599385253	3.03014168	2.72818238	0.04508106
Sis	3.83521425	1.235307777	2.35431568	2.71733789	0.04606657
Tmem150b	5.46015691	1.401371107	2.64152508	2.71657942	0.04614371
Ube2t	36.8157757	1.250077254	2.37854159	2.71540005	0.04627977
Hrh1	4.86220862	1.120188683	2.173754	2.71336266	0.04653655
AC161117,1	8.68220393	1.591832809	3.01432048	2.70946136	0.04693008
Gm6377	9.49238711	1.384243353	2.61035017	2.70889144	0.04696536
Cdc6	29.6691616	1.361226752	2.56903537	2.70555853	0.04736647
Casp4	77.7229438	1.045027172	2.06340522	2.70114351	0.04774172
AC110043,1	3.68242977	1.238450976	2.35945062	2.69978849	0.04788715
Nlrc5	148.288299	1.138500212	2.2015204	2.6966967	0.04808471
Card11	82.578875	1.231122299	2.34749535	2.69560657	0.04816927
Sorcs3	5.26865725	1.391402417	2.62333567	2.69064809	0.04869235
Gm11516	72.5977244	1.023455426	2.03278189	2.68872347	0.04887765
Gk2	47.3956038	1.107209231	2.15428516	2.68732165	0.04900949
Bhmt-ps1	66.8115032	1.091024856	2.13025311	2.68420953	0.04930501
Plg	104.813147	1.110744159	2.15957012	2.68092966	0.04962729

Ocstamp	9.16733929	1.525812396	2.87948817	2.68046532	0.04965838
Mmp25	5.15972448	1.389981371	2.62075297	2.6804203	0.04965838
Klra7	3.71374464	1.215624875	2.32241352	2.68002698	0.0496723
Hic2	337.61036	1.484730514	2.79864891	2.67984029	0.04968519

Genes overlapping in Venn Diagramm (Sup. Fig. S7) with Liu et al., Mass et al.
total: 111 Genes

ptpn6
flna
tgfbi
sdc3
tyrobp
klf13
slamf9
lyz2
hcls1
fgd2
p2ry6
selplg
cyfip1
ccnd2
c3ar1
siglece
pla2g7
h2-aa
pld4
plbd1
arhgdib
fermt3
cybb
slc11a1
laptm5
vav1
ncf2
ctsc
c1qc
c1qb
c1qa
ctss
axl
tubb2a
hpgd
lsp1
dnase1l3
rtp4
lcp1
lilra5
c5ar1
il10ra
ly86
h2-dma
cd93
cd74
cd72

cyth4
csf1r
coro1b
coro1a
txnip
efhd2
pea15a
h2-eb1
cd83
cd86
cd68
tlr2
tlr1
stab1
fam105a
lgals3bp
rgs10
ebi3
h2-dmb1
gsn
fxyd5
cxcl16
hexa
marcks
irf5
irf8
themis2
rac2
trf
cmtm3
myo1f
slco2b1
cfp
pf4
fes
mpeg1
lrrc25
h2-k1
bin2
mafb
nrros
gpr65
mrc1
rhog
adrb2
unc93b1
ms4a7
ehd4
clec4n
h2-ab1

apoe
sirpa
fcgr1
spi1
cx3cr1
tmsb4x
lpcat2
cd14
fcer1g
cd53
fcgr4
fcgr3
dusp6
mgl2

Genes exclusively expressed in Mac^{TRAP} dataset (Venn Diagramm Sup. Fig. S7)
total: 851 Genes

supt6
gm4873
olfr128
gm45910
ighv1-62
gm4907
plxna4os1
gm15931
myo15b
ube2t
casp12
dnajc13
ighv1-81
ighv1-82
nup205
paf1
ap4e1
ankle1
ighv6-3
ccdc88c
akap8l
cul7
usp8
ighv2-6
nes
selenbp1
selenbp2
jaml
gm42888
dctn4
gm44224
wdr7
wdr6
cxcl3
elac1
igkv15-103
adck1
pwp1
lrfn5
ighv8-12
fbxw8
1600010m07rik
mprip
stxbp3
bbc3
agmo

spag5
bms1
il1rn
gm26624
wdr93
gm5996
wdr90
flnb
aass
vnn3
kank4
hltf
kbtbd7
gm5422
cd1d1
efna5
rad50
ppp1r9a
ighmbp2
fetub
eya1
pkib
foxp2
fbxo46
serpinb9f
nup43
gm15922
gmfg
eif3d
crocc
ighv1-76
ighv1-71
ighv1-72
Sep 10
ptprs
myo18a
ptprd
ptpro
kif14
dst
dsp
gm7030
zfp282
h2-q1
h2-q2
nav3
ube3a
pygb
pygl
pygm

pfas
chd8
stx11
sipa1l1
sipa1l2
gm7292
slamf6
dnah8
arhgap23
mpdz
st5
mtbp
ai506816
ctps2
kalrn
aldh1b1
gm43027
olfr816
heatr1
cfap46
ac133079.1
olfr1291-ps1
dscc1
olfr1205
smu1
hk1
nlrx1
dhtkd1
ear1
gm11224
gm11787
klhl28
mettl14
epg5
gm9540
gm8815
gm17944
wdr81
olfr57
lama4
f13b
gfm1
gfm2
plcl1
casc1
ap3m1-ps
mut
utp4
hic2
ascc3

gm28875
anapc4
psmd2
tmem150b
nprl2
myh10
myh11
myh14
a730062m13rik
nexn
ttc21b
zic4
atg7
ermap
ptpn14
acot2
rad21
prkdc
gm42568
bbs2
fam83h
zfp185
klhl9
aifm1
nedd4
actn3
actn1
2900026a02rik
espl1
kcnq1ot1
virma
hmgcs1
bc035044
gm18860
klhl22
myom1
hrh1
coro7
dhx8
plekhg3
gm14548
gm8953
gm26510
sptan1
amot
znrf3
napepld
myo9a
aco1
hdac4

hdac6
hdac8
dlec1
akap8
dstyk
adgrb3
ighv1-62-3
ighv1-62-2
skiv2l2
son
mical3
tnks1bp1
pck2
nudcd1
rtcb
gm11516
ear-ps2
cfhr2
cfhr3
abcb4
pwp2
tns4
klk7
trio
hectd3
hectd1
lrp4
hectd4
h2-ea-ps
gm44436
zfp316
hadhb
hadha
gm13910
ppp2r2a
gm37868
mad1l1
tubgcp6
cmtr2
gm10499
pld5
ighv10-3
ocstamp
bhmt2
gm6382
scn2b
svil
yod1
atp6v1a
pou1f1

plg
cnnm4
cttn
vps16
vps11
ipp
fam196a
gm11218
cluh
cramp1l
fermt1
cad
nemf
ighv4-2
ighv4-1
rapgef3
ai662270
usp29
gm43587
lipt1
gm18445
ac110043.1
gm33370
gm37877
gm37706
apeh
gm18840
serpina3g
amotl2
tollip
fyb2
nbas
syde2
got1
nuf2
pik3c2b
pik3c2a
ctnnbl1
cobl
nwd2
plec
itpr2
itpr3
nup107
dpys
kif26a
acsl4
depdc1a
xpnpep1
myo7b

copa
gm21188
gm26880
wdr35
wdr36
wdr33
gm15964
oplah
cnr1
gm5640
gm5131
sec16b
gm13205
pcm1
cdc42bpb
ren1
eme1
gk2
rrm2
tubgcp2
usp37
smc1a
slc14a1
gm45292
synpo
gm4117
mycn
nphp3
amer1
gm4788
akap11
aox1
cd101
nr6a1
atad2b
d6wsu163e
pik3r4
gfpt1
ambra1
stambp
pole
thbd
ccdc126
gm7823
platr30
ppfia4
prpf8
skiv2l
elovl4
lmod1

ints1
arhgef17
c1rb
ints9
gse1
prokr1
gigyf1
frem2
pepd
gm43728
myo6
ctr9
olfr787
gm16332
fbxo34
plch1
dock9
rubcnl
jak1
cpne8
gm16421
phf11b
phf11c
dars
hsdl2
sec23ip
gm45191
isg15
pik3c3
gm45179
sh2d1b2
trappc9
maoa
btbd9
ston2
lonp2
crybg1
crybg2
mycbp2
helz2
tbck
ac186674.1
mkln1
igsf3
gm15544
stxbp3-ps
lilra6
lrsam1
4933438k21rik
nsun6

vps13d
vps13b
klra7
2900097c17rik
pdcd11
ranbp2
ptpn23
cdh23
reln
dysf
uevld
cct3-ps1
wif1
snd1
myo5c
myo5b
ift172
gm5936
eftud2
sirpb1c
4930402h24rik
nrep
hmmr
ighv9-3
ighv9-1
dppa3
gm5118
kif1b
mylk
lsg1
immt
2010008c14rik
shroom2
vil1
plce1
shroom4
srpk3
pgbd5
exoc4
tspyl3
pfkp
pfkm
papss1
taf7
tes
ighv5-9
msh6
ighv5-4
h2-q6
glud-ps

arhgap45
emilin2
emilin1
cnbd2
hsd17b4
myom2
inpp5b
ighg2b
hacl1
shmt2
gm37629
rhpn2
lima1
rbbp7
pcdh15
polr3b
c1ra
las1l
ntrk3
kdm4c
kdm4b
ddx11
c230071h17rik
shq1
c130073e24rik
gas2
gm6904
5031425f14rik
gm19617
top3a
lmtk2
pdp2
egf
inafm2
helb
pcx
fpr2
mefv
pan2
pdzrn4
ets1
cdk5rap1
sacm1l
cdc25c
clmn
plcd4
gm35570
icam1
suox
fto

taf1
elp2
rad54l
selenon
tmem2
misp
gm10440
pikfyve
ac134249.1
lrch3
kdm5a
kdm5b
kdm5c
pprc1
parn
syne2
fam43a
dtl
pde4c
crnkl1
dsc2
brca2
gys1
olfr1090
gys2
hgd
ighv5-9-1
plat
acaca
cspg4
ighv1-53
tpbgl
9330162b11rik
gm11397
pls1
pls3
nup98
olfr1279
clp1
wdr72
wdr75
bc021767
sec23a
sec23b
supt16
pira2
ighv1-18
gm13710
adam23
gm42715

fignl1
capn7
arhgef28
nectin4
capn8
plcg1
mccc2
mccc1
nup133
itpripl1
gm43715
gm42482
zfp110
gm18360
slc38a11
h2-dmb2
magee1
pmpca
gm15523
atg9b
fuk
birc6
sardh
xirp1
szt2
pyroxd1
nol6
smc3
smc5
exd1
gm37151
exph5
cuedc1
olfr1440
tbc1d4
ttc21a
ift140
ddx3y
igha
ighd
nup88
dcaf12l1
3110080o07rik
rab3gap1
a430093f15rik
wdr61
lars
ahctf1
cd300c2
ighj4

ighj1
tbl3
rngtt
ac155249.1
ighv1-26
cgn
pnpt1
gm4764
efl1
gtf3c1
spg11
acvr2b
ndufs1
zc3hc1
clec4a4
gcm1
zbtb34
ppl
gm43473
jag1
gm5547
cacna1f
fasn
man2c1
aqr
notch2
mdn1
itpka
mcm3ap
ighv5-17
ighv5-16
ighv5-12
zfp568
alox15
gm3650
cd300lb
ddb1
kdm3b
iqgap2
iqgap3
dhx15
shprh
gm10552
gm7848
4632427e13rik
cpsf2
cpsf1
mcub
polr3a
hip1r

acadvl
gm30400
au020206
timd4
mthfd1l
cfh
myo1e
myo1d
myo1c
myo1b
actl6a
fnip1
lpin1
zfp85
utrn
dync2h1
iffo2
myo19
ighv8-8
myo10
ppp2r3a
kntc1
sptbn1
bckdha
cth
serpine2
ifi209
ifi206
ifi207
ifi204
rgsl1
zcchc3
gm4157
4933424m12rik
itfg2
chm
gm14221
gdf3
pcca
xrcc6
tpm3-rs7
2810474o19rik
sptbn2
col12a1
ikbkap
ift122
sis
ighv3-6
ptprz1
slfn10-ps

pigr
a2ml1
lmbr1l
sh3tc2
ac161117.1
adgrl3
gabra2
iars2
herc2
atp1a3
dhx29
mta3
gm7839
nkrf
vwa8
gpd2
macf1
8030453o22rik
rnf40
ubr3
ubr2
ubr1
ubr5
ubr4
gm15107
gas2l3
sorcs3
ac160122.1
polr2b
copb2
brinp3
gm21370
snrnp200
dna2
cep295
phka1
cep290
ciao1
sbno1
limch1
cd300ld4
cd300ld5
cd300ld2
cd300ld3
ifi214
ifi211
gm38259
uqcrc2
ighv2-9-1
pex5

gm26917
gm42585
gprasp1
nup160
tnxb
tnxa
pex14
gm42031
gmfg-ps
mov10
diaph1
gm18752
kyat3
mcemp1
pou5f2
trim30e-ps1
dbt
tcaf1
gm19938
acox3
mga
clec4e
gm17575
atg2b
abcg2
gm7224
dhx36
zfp382
bptf
ythdc2
sec16a
igkv3-12
h2-t-ps
ac156572.1
polr1a
polr1b
polr1c
actr6
ogdh
cd300lf
gldc
ac159261.1
dpysl3
dbn1
nup37
lmo7
olfr286
ac116589.3
gm44291
cep41

cep350
ank2
ank3
mrip-ps
dus2
gm5086
slfn4
tmod2
arsa
ighv1-55
setx
vprbp
gm45697
sytl2
sptb
bhmt-ps1
lrrc40
adamts15
zfp712
olfr716
ado
evpl
ac153729.1
tgm1
scin
stag3
gm10693
dnmt3a
csad
ac153954.1
grid2
nadsyn1
e430014b02rik
mex3d
angpt4
kif9
ac155241.2
gli3
aplp2
gm43845
haus3
4921527h02rik
hp
cgnl1
gm15128
flii
cdc6
luzp2
gm8909
acly

trim6
hps3
maats1
ep400
hps5

Gene Functional Classification
Tool: DAVID
Input: 1448 macrophage translational signature genes
Classification stringency: Medium
Identified gene groups: 59
Comment: sorted by enrichment score

**Gene Group 1**		**Enrichment Score: 22.85**
1	ENSMUSG00000022439	parvin, gamma(Parvg)
2	ENSMUSG00000074570	Cas scaffolding protein family member 4(Cass4)
3	ENSMUSG00000034595	protein phosphatase 1, regulatory subunit 18(Ppp1r18)
4	ENSMUSG00000031351	zinc finger protein 185(Zfp185)
5	ENSMUSG00000039103	nexilin(Nexn)
6	ENSMUSG00000035852	mitotic spindle positioning(Misp)
7	ENSMUSG00000047945	MARCKS-like 1(Marcksl1)
8	ENSMUSG00000069662	myristoylated alanine rich protein kinase C substrate(Marcks)
9	ENSMUSG00000017607	tensin 4(Tns4)
**Gene Group 2**		**Enrichment Score: 21.66**
1	ENSMUSG00000057335	centrosomal protein 170(Cep170)
2	ENSMUSG00000037443	centrosomal protein 85(Cep85)
3	ENSMUSG00000035439	4HAUS augmin-like complex, subunit 8(Haus8)
4	ENSMUSG00000024603	dynactin 4(Dctn4)
5	ENSMUSG00000046111	centrosomal protein 295(Cep295)
**Gene Group 3**		**Enrichment Score: 21.51**
1	ENSMUSG00000027940	tropomyosin 3, gamma(Tpm3)
2	ENSMUSG00000034595	protein phosphatase 1, regulatory subunit 18(Ppp1r18)
3	ENSMUSG00000049775	thymosin, beta 4, X chromosome(Tmsb4x)
4	ENSMUSG00000058587	tropomodulin 3(Tmod3)
**Gene Group 4**		**Enrichment Score: 21.41**
1	ENSMUSG00000038736	NudC domain containing 1(Nudcd1)

2	ENSMUSG00000042834	neuronal regeneration related protein(Nrep)
3	ENSMUSG00000024691	family with sequence similarity 111, member A(Fam111a)
4	ENSMUSG00000043384	G protein-coupled receptor associated sorting protein 1(Gprasp1)
**Gene Group 5**		**Enrichment Score: 18.28**
1	ENSMUSG00000000631	myosin XVIIIA(Myo18a)
2	ENSMUSG00000020437	myosin IG(Myo1g)
3	ENSMUSG00000020900	myosin, heavy polypeptide 10, non-muscle(Myh10)
4	ENSMUSG00000033590	myosin VC(Myo5c)
5	ENSMUSG00000022443	myosin, heavy polypeptide 9, non-muscle(Myh9)
6	ENSMUSG00000032220	myosin IE(Myo1e)
7	ENSMUSG00000018830	myosin, heavy polypeptide 11, smooth muscle(Myh11)
8	ENSMUSG00000020527	myosin XIX(Myo19)
9	ENSMUSG00000004677	myosin IXb(Myo9b)
10	ENSMUSG00000024388	myosin VIIB(Myo7b)
11	ENSMUSG00000030761	myosin VIIA(Myo7a)
12	ENSMUSG00000035441	myosin ID(Myo1d)
13	ENSMUSG00000033577	myosin VI(Myo6)
14	ENSMUSG00000025885	myosin VB(Myo5b)
15	ENSMUSG00000034593	myosin VA(Myo5a)
16	ENSMUSG00000024300	myosin IF(Myo1f)
17	ENSMUSG00000039585	myosin IXa(Myo9a)
18	ENSMUSG00000017774	myosin IC(Myo1c)
19	ENSMUSG00000018417	myosin IB(Myo1b)
20	ENSMUSG00000030739	myosin, heavy polypeptide 14(Myh14)
21	ENSMUSG00000022272	myosin X(Myo10)

22	ENSMUSG00000034427	myosin XVB(Myo15b)
**Gene Group 6**		**Enrichment Score: 14.57**
1	ENSMUSG00000028328	tropomodulin 1(Tmod1)
2	ENSMUSG00000048096	leiomodin 1 (smooth muscle)(Lmod1)
3	ENSMUSG00000032186	tropomodulin 2(Tmod2)
4	ENSMUSG00000058587	tropomodulin 3(Tmod3)
**Gene Group 7**		**Enrichment Score: 13.74**
1	ENSMUSG00000002428	helicase-like transcription factor(Hltf)
2	ENSMUSG00000003660	small nuclear ribonucleoprotein 200 (U5)(Snrnp200)
3	ENSMUSG00000053754	chromodomain helicase DNA binding protein 8(Chd8)
4	ENSMUSG00000037993	DEAH (Asp-Glu-Ala-His) box polypeptide 38(Dhx38)
5	ENSMUSG00000029169	DEAH (Asp-Glu-Ala-His) box polypeptide 15(Dhx15)
6	ENSMUSG00000035455	fidgetin-like 1(Fignl1)
7	ENSMUSG00000020929	elongation factor Tu GTP binding domain containing 2(Eftud2)
8	ENSMUSG00000034653	YTH domain containing 2(Ythdc2)
9	ENSMUSG00000040383	aquarius(Aqr)
10	ENSMUSG00000042426	DEAH (Asp-Glu-Ala-His) box polypeptide 29(Dhx29)
11	ENSMUSG00000036875	DNA replication helicase/nuclease 2(Dna2)
12	ENSMUSG00000029505	E1A binding protein p400(Ep400)
13	ENSMUSG00000020850	pre-mRNA processing factor 8(Prpf8)
14	ENSMUSG00000017830	DEXH (Asp-Glu-X-His) box polypeptide 58(Dhx58)
15	ENSMUSG00000034931	DEAH (Asp-Glu-Ala-His) box polypeptide 8(Dhx8)
16	ENSMUSG00000022360	ATPase family, AAA domain containing 2(Atad2)
17	ENSMUSG00000027580	helicase with zinc finger 2, transcriptional coactivator(Helz2)
18	ENSMUSG00000029730	minichromosome maintenance complex component 7(Mcm7)
19	ENSMUSG00000027770	DEAH (Asp-Glu-Ala-His) box polypeptide 36(Dhx36)

20	ENSMUSG00000002227	Moloney leukemia virus 10(Mov10)
21	ENSMUSG00000005410	minichromosome maintenance complex component 5(Mcm5)
22	ENSMUSG00000069045	DEAD (Asp-Glu-Ala-Asp) box polypeptide 3, Y-linked(Ddx3y)
23	ENSMUSG00000022673	minichromosome maintenance complex component 4(Mcm4)
24	ENSMUSG00000041235	chromodomain helicase DNA binding protein 7(Chd7)
25	ENSMUSG00000038774	activating signal cointegrator 1 complex subunit 3(Ascc3)
26	ENSMUSG00000002870	minichromosome maintenance complex component 2(Mcm2)
27	ENSMUSG00000041859	minichromosome maintenance complex component 3(Mcm3)
28	ENSMUSG00000016018	superkiller viralicidic activity 2-like 2 (S. cerevisiae)(Skiv2l2)
29	ENSMUSG00000090112	SNF2 histone linker PHD RING helicase(Shprh)
30	ENSMUSG00000020228	helicase (DNA) B(Helb)
31	ENSMUSG00000024831	immunoglobulin mu binding protein 2(Ighmbp2)
32	ENSMUSG00000028702	RAD54 like (S. cerevisiae)(Rad54l)
33	ENSMUSG00000033732	splicing factor 3b, subunit 3(Sf3b3)
**Gene Group 8**		**Enrichment Score: 13.32**
1	ENSMUSG00000015745	pleckstrin homology domain containing, family O member 1(Plekho1)
2	ENSMUSG00000024013	FYVE, RhoGEF and PH domain containing 2(Fgd2)
3	ENSMUSG00000026786	amyloid beta (A4) precursor protein-binding, family B, member 1 interacting protein(Apbb1ip)
4	ENSMUSG00000021662	Rho guanine nucleotide exchange factor (GEF) 28(Arhgef28)
5	ENSMUSG00000027356	fermitin family member 1(Fermt1)
**Gene Group 9**		**Enrichment Score: 13.08**
1	ENSMUSG00000051586	microtubule associated monooxygenase, calponin and LIM domain containing 3(Mical3)
2	ENSMUSG00000037736	LIM and calponin homology domains 1(Limch1)
3	ENSMUSG00000029552	testis derived transcript(Tes)
4	ENSMUSG00000024696	leupaxin(Lpxn)
5	ENSMUSG00000023022	LIM domain and actin binding 1(Lima1)

6	ENSMUSG00000022439	parvin, gamma(Parvg)
7	ENSMUSG00000019823	microtubule associated monooxygenase, calponin and LIM domain containing 1(Mical1)
8	ENSMUSG00000031351	zinc finger protein 185(Zfp185)
**Gene Group 10**		**Enrichment Score: 12.16**
1	ENSMUSG00000039316	raftlin lipid raft linker 1(Rftn1)
2	ENSMUSG00000047945	MARCKS-like 1(Marcksl1)
3	ENSMUSG00000045763	brain abundant, membrane attached signal protein 1(Basp1)
4	ENSMUSG00000049866	ADP-ribosylation factor-like 4C(Arl4c)
**Gene Group 11**		**Enrichment Score: 12.14**
1	ENSMUSG00000041642	kinesin family member 21B(Kif21b)
2	ENSMUSG00000021294	kinesin family member 26A(Kif26a)
3	ENSMUSG00000033826	dynein, axonemal, heavy chain 8(Dnah8)
4	ENSMUSG00000063077	kinesin family member 1B(Kif1b)
5	ENSMUSG00000018707	dynein cytoplasmic 1 heavy chain 1(Dync1h1)
6	ENSMUSG00000032489	kinesin family member 9(Kif9)
7	ENSMUSG00000041498	kinesin family member 14(Kif14)
8	ENSMUSG00000032254	kinesin family member 23(Kif23)
**Gene Group 12**		**Enrichment Score: 12**
1	ENSMUSG00000006457	actinin alpha 3(Actn3)
2	ENSMUSG00000020315	spectrin beta, non-erythrocytic 1(Sptbn1)
3	ENSMUSG00000021061	spectrin beta, erythrocytic(Sptb)
4	ENSMUSG00000028649	microtubule-actin crosslinking factor 1(Macf1)
5	ENSMUSG00000026131	dystonin(Dst)
6	ENSMUSG00000057738	spectrin alpha, non-erythrocytic 1(Sptan1)
7	ENSMUSG00000040659	EF hand domain containing 2(Efhd2)
8	ENSMUSG00000024397	allograft inflammatory factor 1(Aif1)

9	ENSMUSG00000049493	plastin 1 (I-isoform)(Pls1)
10	ENSMUSG00000054808	actinin alpha 4(Actn4)
11	ENSMUSG00000021998	lymphocyte cytosolic protein 1(Lcp1)
12	ENSMUSG00000016382	plastin 3 (T-isoform)(Pls3)
13	ENSMUSG00000015143	actinin, alpha 1(Actn1)
**Gene Group 13**		**Enrichment Score: 11.83**
1	ENSMUSG00000031662	sorting nexin 20(Snx20)
2	ENSMUSG00000001150	minichromosome maintenance complex component 3 associated protein(Mcm3ap)
3	ENSMUSG00000068877	selenium binding protein 2(Selenbp2)
4	ENSMUSG00000068874	selenium binding protein 1(Selenbp1)
5	ENSMUSG00000022538	large 60S subunit nuclear export GTPase 1(Lsg1)
**Gene Group 14**		**Enrichment Score: 10.88**
1	ENSMUSG00000032344	Mab-21 domain containing 1(Mb21d1)
2	ENSMUSG00000032661	2'-5' oligoadenylate synthetase 3(Oas3)
3	ENSMUSG00000041827	2'-5' oligoadenylate synthetase-like 1(Oasl1)
4	ENSMUSG00000029561	2'-5' oligoadenylate synthetase-like 2(Oasl2)
5	ENSMUSG00000066861	2'-5' oligoadenylate synthetase 1G(Oas1g)
6	ENSMUSG00000052776	2'-5' oligoadenylate synthetase 1A(Oas1a)
7	ENSMUSG00000017830	DEXH (Asp-Glu-X-His) box polypeptide 58(Dhx58)
**Gene Group 15**		**Enrichment Score: 9.62**
1	ENSMUSG00000045374	WD repeat domain 81(Wdr81)
2	ENSMUSG00000066643	WD repeat domain 35(Wdr35)
3	ENSMUSG00000037474	denticleless E3 ubiquitin protein ligase(Dtl)
4	ENSMUSG00000028409	smu-1 suppressor of mec-8 and unc-52 homolog (C. elegans)(Smu1)
5	ENSMUSG00000032458	coatomer protein complex, subunit beta 2 (beta prime)(Copb2)
6	ENSMUSG00000032834	PWP2 periodic tryptophan protein homolog (yeast)(Pwp2)

7	ENSMUSG00000035351	nucleoporin 37(Nup37)
8	ENSMUSG00000022364	TBC1 domain family, member 31(Tbc1d31)
9	ENSMUSG00000006281	telomerase associated protein 1(Tep1)
10	ENSMUSG00000038299	WD repeat domain 36(Wdr36)
11	ENSMUSG00000025995	WD repeat domain 75(Wdr75)
12	ENSMUSG00000040560	WD repeat domain 7(Wdr7)
13	ENSMUSG00000040034	nucleoporin 43(Nup43)
14	ENSMUSG00000073434	WD repeat domain 90(Wdr90)
15	ENSMUSG00000041642	kinesin family member 21B(Kif21b)
16	ENSMUSG00000022052	protein phosphatase 2, regulatory subunit B, alpha(Ppp2r2a)
17	ENSMUSG00000039637	coronin 7(Coro7)
18	ENSMUSG00000031353	retinoblastoma binding protein 7(Rbbp7)
19	ENSMUSG00000090061	NACHT and WD repeat domain containing 2(Nwd2)
20	ENSMUSG00000001785	PWP1 homolog, endonuclein(Pwp1)
21	ENSMUSG00000066979	BUB3 mitotic checkpoint protein(Bub3)
22	ENSMUSG00000040688	transducin (beta)-like 3(Tbl3)
23	ENSMUSG00000045284	DDB1 and CUL4 associated factor 12-like 1(Dcaf12l1)
24	ENSMUSG00000026553	coatomer protein complex subunit alpha(Copa)
25	ENSMUSG00000024400	WD repeat domain 33(Wdr33)
26	ENSMUSG00000041438	cirrhosis, autosomal recessive 1A (human)(Cirh1a)
27	ENSMUSG00000066357	WD repeat domain 6(Wdr6)
28	ENSMUSG00000032867	F-box and WD-40 domain protein 8(Fbxw8)
29	ENSMUSG00000024271	elongator acetyltransferase complex subunit 2(Elp2)
30	ENSMUSG00000024835	coronin, actin binding protein 1B(Coro1b)
31	ENSMUSG00000044976	WD repeat domain 72(Wdr72)

32	ENSMUSG00000003662	cytosolic iron-sulfur protein assembly 1(Ciao1)
33	ENSMUSG00000061559	WD repeat domain 61(Wdr61)
**Gene Group 16**		**Enrichment Score: 9.56**
1	ENSMUSG00000063550	nucleoporin 98(Nup98)
2	ENSMUSG00000002055	sperm associated antigen 5(Spag5)
3	ENSMUSG00000029414	kinetochore associated 1(Kntc1)
4	ENSMUSG00000035852	mitotic spindle positioning(Misp)
5	ENSMUSG00000035351	nucleoporin 37(Nup37)
6	ENSMUSG00000040667	nucleoporin 88(Nup88)
7	ENSMUSG00000034906	non-SMC condensin I complex, subunit H(Ncaph)
8	ENSMUSG00000050379	septin 6(Sept6)
9	ENSMUSG00000051329	nucleoporin 160(Nup160)
10	ENSMUSG00000039130	zinc finger, C3HC type 1(Zc3hc1)
11	ENSMUSG00000079555	HAUS augmin-like complex, subunit 3(Haus3)
12	ENSMUSG00000040034	nucleoporin 43(Nup43)
13	ENSMUSG00000052798	nucleoporin 107(Nup107)
14	ENSMUSG00000026491	AT hook containing transcription factor 1(Ahctf1)
15	ENSMUSG00000066979	BUB3 mitotic checkpoint protein(Bub3)
16	ENSMUSG00000029554	MAD1 mitotic arrest deficient 1-like 1(Mad1l1)
17	ENSMUSG00000048170	minichromosome maintenance complex binding protein(Mcmbp)
18	ENSMUSG00000035439	4HAUS augmin-like complex, subunit 8(Haus8)
19	ENSMUSG00000019917	septin 10(Sept10)
20	ENSMUSG00000001150	minichromosome maintenance complex component 3 associated protein(Mcm3ap)
21	ENSMUSG00000039509	nucleoporin 133(Nup133)
22	ENSMUSG00000032254	kinesin family member 23(Kif23)

23	ENSMUSG00000026683	NUF2, NDC80 kinetochore complex component(Nuf2)
**Gene Group 17**		**Enrichment Score: 9.1**
1	ENSMUSG00000035697	histocompatibility (minor) HA-1(Hmha1)
2	ENSMUSG00000030047	Rho GTPase activating protein 25(Arhgap25)
3	ENSMUSG00000040345	Rho GTPase activating protein 9(Arhgap9)
4	ENSMUSG00000063506	Rho GTPase activating protein 22(Arhgap22)
5	ENSMUSG00000004952	RAS p21 protein activator 4(Rasa4)
6	ENSMUSG00000048865	Rho GTPase activating protein 30(Arhgap30)
7	ENSMUSG00000036863	synapse defective 1, Rho GTPase, homolog 2 (C. elegans)(Syde2)
8	ENSMUSG00000031214	oligophrenin 1(Ophn1)
9	ENSMUSG00000052142	RAS protein activator like 3(Rasal3)
10	ENSMUSG00000031453	RAS p21 protein activator 3(Rasa3)
11	ENSMUSG00000028175	DEP domain containing 1a(Depdc1a)
12	ENSMUSG00000049807	Rho GTPase activating protein 23(Arhgap23)
13	ENSMUSG00000041219	Rho GTPase activating protein 11A(Arhgap11a)
**Gene Group 18**		**Enrichment Score: 8.78**
1	ENSMUSG00000032571	phosphatidylinositol 3 kinase, regulatory subunit, polypeptide 4, p150(Pik3r4)
2	ENSMUSG00000022263	triple functional domain (PTPRF interacting)(Trio)
3	ENSMUSG00000028874	FGR proto-oncogene, Src family tyrosine kinase(Fgr)
4	ENSMUSG00000022221	receptor-interacting serine-threonine kinase 3(Ripk3)
5	ENSMUSG00000026094	serine/threonine kinase 17b (apoptosis-inducing)(Stk17b)
6	ENSMUSG00000027296	inositol 1,4,5-trisphosphate 3-kinase A(Itpka)
7	ENSMUSG00000042228	LYN proto-oncogene, Src family tyrosine kinase(Lyn)
8	ENSMUSG00000021754	mitogen-activated protein kinase kinase kinase 1(Map3k1)
9	ENSMUSG00000020115	TANK-binding kinase 1(Tbk1)
10	ENSMUSG00000019942	cyclin-dependent kinase 1(Cdk1)

11	ENSMUSG00000003283	hemopoietic cell kinase(Hck)
12	ENSMUSG00000042046	dual serine/threonine and tyrosine protein kinase(Dstyk)
13	ENSMUSG00000038970	lemur tyrosine kinase 2(Lmtk2)
14	ENSMUSG00000021279	CDC42 binding protein kinase beta(Cdc42bpb)
15	ENSMUSG00000029366	deoxycytidine kinase(Dck)
16	ENSMUSG00000021044	aarF domain containing kinase 1(Adck1)
17	ENSMUSG00000034663	BMP2 inducible kinase(Bmp2k)
18	ENSMUSG00000039145	calcium/calmodulin-dependent protein kinase ID(Camk1d)
19	ENSMUSG00000000530	activin A receptor, type II-like 1(Acvrl1)
20	ENSMUSG00000061393	activin receptor IIB(Acvr2b)
21	ENSMUSG00000026737	phosphatidylinositol-5-phosphate 4-kinase, type II, alpha(Pip4k2a)
22	ENSMUSG00000033703	fucokinase(Fuk)
23	ENSMUSG00000053158	feline sarcoma oncogene(Fes)
24	ENSMUSG00000002007	serine/arginine-rich protein specific kinase 3(Srpk3)
25	ENSMUSG00000031264	Bruton agammaglobulinemia tyrosine kinase(Btk)
26	ENSMUSG00000050553	glycerol kinase 2(Gk2)
27	ENSMUSG00000022836	myosin, light polypeptide kinase(Mylk)
28	ENSMUSG00000061751	kalirin, RhoGEF kinase(Kalrn)
29	ENSMUSG00000059883	interleukin-1 receptor-associated kinase 4(Irak4)
30	ENSMUSG00000021180	ribosomal protein S6 kinase, polypeptide 5(Rps6ka5)
31	ENSMUSG00000028530	Janus kinase 1(Jak1)
32	ENSMUSG00000021457	spleen tyrosine kinase(Syk)
33	ENSMUSG00000020272	serine/threonine kinase 10(Stk10)
**Gene Group 19**		**Enrichment Score: 7.7**
1	ENSMUSG00000026879	gelsolin(Gsn)

2	ENSMUSG00000026175	villin 1(Vil1)
3	ENSMUSG00000002565	scinderin(Scin)
4	ENSMUSG00000024236	supervillin(Svil)
**Gene Group 20**		**Enrichment Score: 7.41**
1	ENSMUSG00000035247	HECT domain containing 1(Hectd1)
2	ENSMUSG00000025326	ubiquitin protein ligase E3A(Ube3a)
3	ENSMUSG00000046861	HECT domain containing 3(Hectd3)
4	ENSMUSG00000042744	predicted gene 15800(Gm15800)
**Gene Group 21**		**Enrichment Score: 7.26**
1	ENSMUSG00000051786	tubulin, gamma complex associated protein 6(Tubgcp6)
2	ENSMUSG00000033790	tubulin, gamma complex associated protein 5(Tubgcp5)
3	ENSMUSG00000057335	centrosomal protein 170(Cep170)
4	ENSMUSG00000025474	tubulin, gamma complex associated protein 2(Tubgcp2)
5	ENSMUSG00000035439	4HAUS augmin-like complex, subunit 8(Haus8)
6	ENSMUSG00000074802	growth arrest-specific 2 like 3(Gas2l3)
**Gene Group 22**		**Enrichment Score: 7.06**
1	ENSMUSG00000019971	centrosomal protein 290(Cep290)
2	ENSMUSG00000029790	centrosomal protein 41(Cep41)
3	ENSMUSG00000031755	Bardet-Biedl syndrome 2 (human)(Bbs2)
4	ENSMUSG00000031592	pericentriolar material 1(Pcm1)
**Gene Group 23**		**Enrichment Score: 7.04**
1	ENSMUSG00000078763	schlafen 1(Slfn1)
2	ENSMUSG00000035208	schlafen 8(Slfn8)
3	ENSMUSG00000054404	schlafen 5(Slfn5)
4	ENSMUSG00000069793	schlafen 9(Slfn9)
**Gene Group 24**		**Enrichment Score: 6.94**
1	ENSMUSG00000039621	phosphatidylinositol-3,4,5-trisphosphate-dependent Rac exchange factor 1(Prex1)

2	ENSMUSG00000034116	vav 1 oncogene(Vav1)
3	ENSMUSG00000031133	Rac/Cdc42 guanine nucleotide exchange factor (GEF) 6(Arhgef6)
4	ENSMUSG00000021662	Rho guanine nucleotide exchange factor (GEF) 28(Arhgef28)
5	ENSMUSG00000052609	pleckstrin homology domain containing, family G (with RhoGef domain) member 3(Plekhg3)
**Gene Group 25**		**Enrichment Score: 6.93**
1	ENSMUSG00000026792	leucine rich repeat and sterile alpha motif containing 1(Lrsam1)
2	ENSMUSG00000070327	ring finger protein 213(Rnf213)
3	ENSMUSG00000038068	ring finger protein 144B(Rnf144b)
4	ENSMUSG00000030816	ring finger protein 40(Rnf40)
5	ENSMUSG00000047180	neuralized E3 ubiquitin protein ligase 3(Neurl3)
6	ENSMUSG00000041961	zinc and ring finger 3(Znrf3)
7	ENSMUSG00000039328	ring finger protein 122(Rnf122)
8	ENSMUSG00000027272	ubiquitin protein ligase E3 component n-recognin 1(Ubr1)
9	ENSMUSG00000044308	ubiquitin protein ligase E3 component n-recognin 3(Ubr3)
10	ENSMUSG00000066036	ubiquitin protein ligase E3 component n-recognin 4(Ubr4)
11	ENSMUSG00000023977	ubiquitin protein ligase E3 component n-recognin 2(Ubr2)
**Gene Group 26**		**Enrichment Score: 6.8**
1	ENSMUSG00000036894	RAP2B, member of RAS oncogene family(Rap2b)
2	ENSMUSG00000028268	guanylate binding protein 3(Gbp3)
3	ENSMUSG00000049866	ADP-ribosylation factor-like 4C(Arl4c)
4	ENSMUSG00000003411	RAB3B, member RAS oncogene family(Rab3b)
5	ENSMUSG00000033220	RAS-related C3 botulinum substrate 2(Rac2)
6	ENSMUSG00000052688	RAB7B, member RAS oncogene family(Rab7b)
7	ENSMUSG00000000386	MX dynamin-like GTPase 1(Mx1)
8	ENSMUSG00000029204	ras homolog family member H(Rhoh)
9	ENSMUSG00000022538	large 60S subunit nuclear export GTPase 1(Lsg1)

10	ENSMUSG00000028270	guanylate binding protein 2(Gbp2)
11	ENSMUSG00000040264	guanylate binding protein 2b(Gbp2b)
12	ENSMUSG00000073982	ras homolog family member G(Rhog)
**Gene Group 27**		**Enrichment Score: 6.61**
1	ENSMUSG00000019960	dual specificity phosphatase 6(Dusp6)
2	ENSMUSG00000037926	slingshot homolog 2 (Drosophila)(Ssh2)
3	ENSMUSG00000031506	protein tyrosine phosphatase, non-receptor type 7(Ptpn7)
4	ENSMUSG00000026604	protein tyrosine phosphatase, non-receptor type 14(Ptpn14)
**Gene Group 28**		**Enrichment Score: 6.48**
1	ENSMUSG00000054889	desmoplakin(Dsp)
2	ENSMUSG00000034282	envoplakin(Evpl)
3	ENSMUSG00000022565	plectin(Plec)
4	ENSMUSG00000039457	periplakin(Ppl)
**Gene Group 29**		**Enrichment Score: 6.38**
1	ENSMUSG00000025558	dedicator of cytokinesis 9(Dock9)
2	ENSMUSG00000031093	dedicator of cytokinesis 11(Dock11)
3	ENSMUSG00000038608	dedicator of cytokinesis 10(Dock10)
4	ENSMUSG00000020143	dedicator of cyto-kinesis 2(Dock2)
**Gene Group 30**		**Enrichment Score: 6.33**
1	ENSMUSG00000024943	structural maintenance of chromosomes 5(Smc5)
2	ENSMUSG00000022314	RAD21 cohesin complex component(Rad21)
3	ENSMUSG00000028702	RAD54 like (S. cerevisiae)(Rad54l)
4	ENSMUSG00000024974	structural maintenance of chromosomes 3(Smc3)
5	ENSMUSG00000041133	structural maintenance of chromosomes 1A(Smc1a)
6	ENSMUSG00000020380	RAD50 double strand break repair protein(Rad50)
**Gene Group 31**		**Enrichment Score: 6.23**
1	ENSMUSG00000024169	intraflagellar transport 140(Ift140)

2	ENSMUSG00000066643	WD repeat domain 35(Wdr35)
3	ENSMUSG00000029790	centrosomal protein 41(Cep41)
4	ENSMUSG00000030323	intraflagellar transport 122(Ift122)
**Gene Group 32**		**Enrichment Score: 6.12**
1	ENSMUSG00000038910	phospholipase C-like 2(Plcl2)
2	ENSMUSG00000034330	phospholipase C, gamma 2(Plcg2)
3	ENSMUSG00000016933	phospholipase C, gamma 1(Plcg1)
4	ENSMUSG00000024998	phospholipase C, epsilon 1(Plce1)
5	ENSMUSG00000036834	phospholipase C, eta 1(Plch1)
6	ENSMUSG00000026173	phospholipase C, delta 4(Plcd4)
7	ENSMUSG00000038349	phospholipase C-like 1(Plcl1)
**Gene Group 33**		**Enrichment Score: 6.09**
1	ENSMUSG00000026979	pleckstrin and Sec7 domain containing 4(Psd4)
2	ENSMUSG00000028159	dual adaptor for phosphotyrosine and 3-phosphoinositides 1(Dapp1)
3	ENSMUSG00000050721	pleckstrin homology domain containing, family O member 2(Plekho2)
4	ENSMUSG00000018008	cytohesin 4(Cyth4)
**Gene Group 34**		**Enrichment Score: 5.98**
1	ENSMUSG00000052384	negative regulator of reactive oxygen species(Nrros)
2	ENSMUSG00000038179	SLAM family member 7(Slamf7)
3	ENSMUSG00000049988	leucine rich repeat containing 25(Lrrc25)
4	ENSMUSG00000022801	leucine-rich repeats and calponin homology (CH) domain containing 3(Lrch3)
5	ENSMUSG00000035653	leucine rich repeat and fibronectin type III domain containing 5(Lrfn5)
6	ENSMUSG00000044583	toll-like receptor 7(Tlr7)
7	ENSMUSG00000068015	leucine-rich repeats and calponin homology (CH) domain containing 1(Lrch1)
8	ENSMUSG00000021624	CD180 antigen(Cd180)
9	ENSMUSG00000002812	flightless I actin binding protein(Flii)

10	ENSMUSG00000051439	CD14 antigen(Cd14)
11	ENSMUSG00000027995	toll-like receptor 2(Tlr2)
12	ENSMUSG00000063052	leucine rich repeat containing 40(Lrrc40)
13	ENSMUSG00000033777	toll-like receptor 13(Tlr13)
14	ENSMUSG00000051498	toll-like receptor 6(Tlr6)
15	ENSMUSG00000070942	interleukin 1 receptor-like 2(Il1rl2)
16	ENSMUSG00000044827	toll-like receptor 1(Tlr1)
**Gene Group 35**		**Enrichment Score: 5.76**
1	ENSMUSG00000024030	ATP-binding cassette, sub-family G (WHITE), member 1(Abcg1)
2	ENSMUSG00000037321	transporter 1, ATP-binding cassette, sub-family B (MDR/TAP)(Tap1)
3	ENSMUSG00000042476	ATP-binding cassette, sub-family B (MDR/TAP), member 4(Abcb4)
4	ENSMUSG00000020865	ATP-binding cassette, sub-family C (CFTR/MRP), member 3(Abcc3)
5	ENSMUSG00000029802	ATP-binding cassette, sub-family G (WHITE), member 2(Abcg2)
6	ENSMUSG00000055782	ATP-binding cassette, sub-family D (ALD), member 2(Abcd2)
7	ENSMUSG00000041797	ATP-binding cassette, sub-family A (ABC1), member 9(Abca9)
**Gene Group 36**		**Enrichment Score: 5.74**
1	ENSMUSG00000024621	colony stimulating factor 1 receptor(Csf1r)
2	ENSMUSG00000059146	neurotrophic tyrosine kinase, receptor, type 3(Ntrk3)
3	ENSMUSG00000061393	activin receptor IIB(Acvr2b)
4	ENSMUSG00000038970	lemur tyrosine kinase 2(Lmtk2)
5	ENSMUSG00000002602	AXL receptor tyrosine kinase(Axl)
**Gene Group 37**		**Enrichment Score: 5.52**
1	ENSMUSG00000024045	A kinase (PRKA) anchor protein 8(Akap8)
2	ENSMUSG00000008496	POU domain, class 2, transcription factor 2(Pou2f2)
3	ENSMUSG00000053007	cAMP responsive element binding protein 5(Creb5)
4	ENSMUSG00000032035	E26 avian leukemia oncogene 1, 5' domain(Ets1)

5	ENSMUSG00000040829	zinc finger, MYND-type containing 15(Zmynd15)
6	ENSMUSG00000070034	Sp110 nuclear body protein(Sp110)
7	ENSMUSG00000070691	runt related transcription factor 3(Runx3)
8	ENSMUSG00000002325	interferon regulatory factor 9(Irf9)
9	ENSMUSG00000034041	lymphoblastomic leukemia 1(Lyl1)
10	ENSMUSG00000052040	Kruppel-like factor 13(Klf13)
11	ENSMUSG00000039699	basic leucine zipper transcription factor, ATF-like 2(Batf2)
12	ENSMUSG00000071359	TATA box binding protein-like 1(Tbpl1)
13	ENSMUSG00000029771	interferon regulatory factor 5(Irf5)
14	ENSMUSG00000004842	POU domain, class 1, transcription factor 1(Pou1f1)
15	ENSMUSG00000004661	AT rich interactive domain 3B (BRIGHT-like)(Arid3b)
16	ENSMUSG00000039158	AT-hook transcription factor(Akna)
17	ENSMUSG00000093668	POU domain class 5, transcription factor 2(Pou5f2)
18	ENSMUSG00000002111	spleen focus forming virus (SFFV) proviral integration oncogene(Spi1)
19	ENSMUSG00000074622	v-maf musculoaponeurotic fibrosarcoma oncogene family, protein B (avian)(Mafb)
20	ENSMUSG00000026628	activating transcription factor 3(Atf3)
21	ENSMUSG00000027580	helicase with zinc finger 2, transcriptional coactivator(Helz2)
22	ENSMUSG00000044149	NF-kappaB repressing factor(Nkrf)
23	ENSMUSG00000034266	basic leucine zipper transcription factor, ATF-like(Batf)
24	ENSMUSG00000041515	interferon regulatory factor 8(Irf8)
25	ENSMUSG00000063972	nuclear receptor subfamily 6, group A, member 1(Nr6a1)
26	ENSMUSG00000033943	MAX gene associated(Mga)
27	ENSMUSG00000026630	basic leucine zipper transcription factor, ATF-like 3(Batf3)
28	ENSMUSG00000023333	glial cells missing homolog 1 (Drosophila)(Gcm1)
29	ENSMUSG00000031103	E74-like factor 4 (ets domain transcription factor)(Elf4)

30	ENSMUSG00000018899	interferon regulatory factor 1(Irf1)
31	ENSMUSG00000016087	Friend leukemia integration 1(Fli1)
32	ENSMUSG00000055491	peroxisome proliferative activated receptor, gamma, coactivator-related 1(Pprc1)
33	ENSMUSG00000026222	nuclear antigen Sp100(Sp100)
34	ENSMUSG00000002625	A kinase (PRKA) anchor protein 8-like(Akap8l)
35	ENSMUSG00000004359	Spi-C transcription factor (Spi-1/PU.1 related)(Spic)
**Gene Group 38**		**Enrichment Score: 4.93**
1	ENSMUSG00000041650	propionyl-Coenzyme A carboxylase, alpha polypeptide(Pcca)
2	ENSMUSG00000024892	pyruvate carboxylase(Pcx)
3	ENSMUSG00000020532	acetyl-Coenzyme A carboxylase alpha(Acaca)
4	ENSMUSG00000027709	methylcrotonoyl-Coenzyme A carboxylase 1 (alpha)(Mccc1)
**Gene Group 39**		**Enrichment Score: 4.85**
1	ENSMUSG00000003865	glycogen synthase 1, muscle(Gys1)
2	ENSMUSG00000030244	glycogen synthase 2(Gys2)
3	ENSMUSG00000021069	liver glycogen phosphorylase(Pygl)
4	ENSMUSG00000032648	muscle glycogen phosphorylase(Pygm)
5	ENSMUSG00000033059	brain glycogen phosphorylase(Pygb)
**Gene Group 40**		**Enrichment Score: 4.77**
1	ENSMUSG00000030107	ubiquitin specific peptidase 18(Usp18)
2	ENSMUSG00000027363	ubiquitin specific peptidase 8(Usp8)
3	ENSMUSG00000046404	YOD1 deubiquitinase(Yod1)
4	ENSMUSG00000051527	ubiquitin specific peptidase 29(Usp29)
5	ENSMUSG00000033364	ubiquitin specific peptidase 37(Usp37)
6	ENSMUSG00000031066	ubiquitin specific peptidase 11(Usp11)
**Gene Group 41**		**Enrichment Score: 4.75**
1	ENSMUSG00000020929	elongation factor Tu GTP binding domain containing 2(Eftud2)
2	ENSMUSG00000038563	elongation factor like GPTase 1(Efl1)

3	ENSMUSG00000021666	G elongation factor, mitochondrial 2(Gfm2)
4	ENSMUSG00000027774	G elongation factor, mitochondrial 1(Gfm1)
**Gene Group 42**		**Enrichment Score: 4.62**
1	ENSMUSG00000043263	interferon activated gene 209(Ifi209)
2	ENSMUSG00000026536	interferon activated gene 211(Ifi211)
3	ENSMUSG00000070501	interferon activated gene 214(Ifi214)
4	ENSMUSG00000073490	interferon activated gene 207(Ifi207)
5	ENSMUSG00000073489	interferon activated gene 204(Ifi204)
**Gene Group 43**		**Enrichment Score: 4.29**
1	ENSMUSG00000030214	phospholipase B domain containing 1(Plbd1)
2	ENSMUSG00000028885	sphingomyelin phosphodiesterase, acid-like 3B(Smpdl3b)
3	ENSMUSG00000023913	phospholipase A2, group VII (platelet-activating factor acetylhydrolase, plasma)(Pla2g7)
4	ENSMUSG00000031903	phospholipase A2, group XV(Pla2g15)
**Gene Group 44**		**Enrichment Score: 4.09**
1	ENSMUSG00000019122	chemokine (C-C motif) ligand 9(Ccl9)
2	ENSMUSG00000009185	chemokine (C-C motif) ligand 8(Ccl8)
3	ENSMUSG00000035373	chemokine (C-C motif) ligand 7(Ccl7)
4	ENSMUSG00000018927	chemokine (C-C motif) ligand 6(Ccl6)
5	ENSMUSG00000018930	chemokine (C-C motif) ligand 4(Ccl4)
6	ENSMUSG00000000982	chemokine (C-C motif) ligand 3(Ccl3)
7	ENSMUSG00000029417	chemokine (C-X-C motif) ligand 9(Cxcl9)
8	ENSMUSG00000035352	chemokine (C-C motif) ligand 12(Ccl12)
9	ENSMUSG00000029379	chemokine (C-X-C motif) ligand 3(Cxcl3)
10	ENSMUSG00000029373	platelet factor 4(Pf4)
**Gene Group 45**		**Enrichment Score: 3.86**
1	ENSMUSG00000000628	hexokinase 2(Hk2)
2	ENSMUSG00000025877	hexokinase 3(Hk3)

3	ENSMUSG00000037012	hexokinase 1(Hk1)
4	ENSMUSG00000021196	phosphofructokinase, platelet(Pfkp)
**Gene Group 46**		**Enrichment Score: 3.84**
1	ENSMUSG00000026589	SEC16 homolog B (S. cerevisiae)(Sec16b)
2	ENSMUSG00000020986	SEC23 homolog A, COPII coat complex component(Sec23a)
3	ENSMUSG00000027429	SEC23 homolog B, COPII coat complex component(Sec23b)
4	ENSMUSG00000001998	adaptor-related protein complex AP-4, epsilon 1(Ap4e1)
**Gene Group 47**		**Enrichment Score: 3.76**
1	ENSMUSG00000039899	fibrinogen-like protein 2(Fgl2)
2	ENSMUSG00000033880	lectin, galactoside-binding, soluble, 3 binding protein(Lgals3bp)
3	ENSMUSG00000055172	complement component 1, r subcomponent A(C1ra)
4	ENSMUSG00000024053	elastin microfibril interfacer 2(Emilin2)
5	ENSMUSG00000035493	transforming growth factor, beta induced(Tgfbi)
6	ENSMUSG00000029163	elastin microfibril interfacer 1(Emilin1)
7	ENSMUSG00000030713	kallikrein related-peptidase 7 (chymotryptic, stratum corneum)(Klk7)
8	ENSMUSG00000036896	complement component 1, q subcomponent, C chain(C1qc)
9	ENSMUSG00000026365	complement component factor h(Cfh)
10	ENSMUSG00000036905	complement component 1, q subcomponent, beta polypeptide(C1qb)
11	ENSMUSG00000098470	complement component 1, r subcomponent B(C1rb)
12	ENSMUSG00000036887	complement component 1, q subcomponent, alpha polypeptide(C1qa)
13	ENSMUSG00000031538	plasminogen activator, tissue(Plat)
**Gene Group 48**		**Enrichment Score: 3.73**
1	ENSMUSG00000025240	SAC1 suppressor of actin mutations 1-like (yeast)(Sacm1l)
2	ENSMUSG00000037318	TRAF3 interacting protein 3(Traf3ip3)
3	ENSMUSG00000043832	C-type lectin domain family 4, member a3(Clec4a3)
4	ENSMUSG00000074743	thrombomodulin(Thbd)

5	ENSMUSG00000032911	chondroitin sulfate proteoglycan 4(Cspg4)
6	ENSMUSG00000030737	solute carrier organic anion transporter family, member 2b1(Slco2b1)
7	ENSMUSG00000059639	C-type lectin domain family 4, member a4(Clec4a4)
8	ENSMUSG00000057447	olfactory receptor 1205(Olfr1205)
9	ENSMUSG00000030745	interleukin 21 receptor(Il21r)
10	ENSMUSG00000025044	macrophage scavenger receptor 1(Msr1)
11	ENSMUSG00000059089	Fc receptor, IgG, low affinity IV(Fcgr4)
12	ENSMUSG00000033355	receptor transporter protein 4(Rtp4)
13	ENSMUSG00000036353	purinergic receptor P2Y, G-protein coupled 12(P2ry12)
14	ENSMUSG00000055546	T cell immunoglobulin and mucin domain containing 4(Timd4)
15	ENSMUSG00000027276	jagged 1(Jag1)
16	ENSMUSG00000035653	leucine rich repeat and fibronectin type III domain containing 5(Lrfn5)
17	ENSMUSG00000060205	olfactory receptor 57(Olfr57)
18	ENSMUSG00000096606	trophoblast glycoprotein-like(Tpbgl)
19	ENSMUSG00000031497	tumor necrosis factor (ligand) superfamily, member 13b(Tnfsf13b)
20	ENSMUSG00000079298	killer cell lectin-like receptor subfamily B member 1B(Klrb1b)
21	ENSMUSG00000031494	CD209a antigen(Cd209a)
22	ENSMUSG00000058818	paired Ig-like receptor B(Pirb)
23	ENSMUSG00000037016	Fras1 related extracellular matrix protein 2(Frem2)
24	ENSMUSG00000049409	prokineticin receptor 1(Prokr1)
25	ENSMUSG00000037605	adhesion G protein-coupled receptor L3(Adgrl3)
26	ENSMUSG00000028859	colony stimulating factor 3 receptor (granulocyte)(Csf3r)
27	ENSMUSG00000030579	TYRO protein tyrosine kinase binding protein(Tyrobp)
28	ENSMUSG00000039774	UDP-N-acetyl-alpha-D-galactosamine:polypeptide N-acetylgalactosaminyltransferase 12(Galnt12)
29	ENSMUSG00000071714	colony stimulating factor 2 receptor, beta 2, low-affinity (granulocyte-macrophage)(Csf2rb2)

30	ENSMUSG00000071713	colony stimulating factor 2 receptor, beta, low-affinity (granulocyte-macrophage)(Csf2rb)
31	ENSMUSG00000079293	C-type lectin domain family 7, member a(Clec7a)
32	ENSMUSG00000059498	Fc receptor, IgG, low affinity III(Fcgr3)
33	ENSMUSG00000030427	leukocyte immunoglobulin-like receptor, subfamily A (with TM domain), member 6(Lilra6)
34	ENSMUSG00000041341	autophagy related 2B(Atg2b)
35	ENSMUSG00000089942	paired-Ig-like receptor A2(Pira2)
36	ENSMUSG00000015947	Fc receptor, IgG, high affinity I(Fcgr1)
37	ENSMUSG00000032076	cell adhesion molecule 1(Cadm1)
38	ENSMUSG00000058715	Fc receptor, IgE, high affinity I, gamma polypeptide(Fcer1g)
39	ENSMUSG00000081665	paired-Ig-like receptor A1(Pira1)
40	ENSMUSG00000029547	integrator complex subunit 1(Ints1)
41	ENSMUSG00000039497	dermatan sulfate epimerase(Dse)
42	ENSMUSG00000079017	interferon, alpha-inducible protein 27 like 2A(Ifi27l2a)
43	ENSMUSG00000031875	CKLF-like MARVEL transmembrane domain containing 3(Cmtm3)
44	ENSMUSG00000004730	adhesion G protein-coupled receptor E1(Adgre1)
45	ENSMUSG00000040212	epithelial membrane protein 3(Emp3)
46	ENSMUSG00000074785	plexin C1(Plxnc1)
47	ENSMUSG00000030789	integrin alpha X(Itgax)
48	ENSMUSG00000048779	pyrimidinergic receptor P2Y, G-protein coupled, 6(P2ry6)
49	ENSMUSG00000028581	lysosomal-associated protein transmembrane 5(Laptm5)
50	ENSMUSG00000023992	triggering receptor expressed on myeloid cells 2(Trem2)
51	ENSMUSG00000070873	leukocyte immunoglobulin-like receptor, subfamily A (with TM domain), member 5(Lilra5)
52	ENSMUSG00000044811	CD300C molecule 2(Cd300c2)
53	ENSMUSG00000026417	polymeric immunoglobulin receptor(Pigr)
54	ENSMUSG00000075172	olfactory receptor 1090(Olfr1090)

55	ENSMUSG00000023349	C-type lectin domain family 4, member n(Clec4n)
56	ENSMUSG00000030142	C-type lectin domain family 4, member e(Clec4e)
57	ENSMUSG00000026548	SLAM family member 9(Slamf9)
58	ENSMUSG00000069607	CD300 molecule like family member D3(Cd300ld3)
59	ENSMUSG00000053063	C-type lectin domain family 12, member a(Clec12a)
60	ENSMUSG00000046456	transmembrane protein 150B(Tmem150b)
61	ENSMUSG00000026113	inositol polyphosphate-4-phosphatase, type I(Inpp4a)
62	ENSMUSG00000031996	amyloid beta (A4) precursor-like protein 2(Aplp2)
63	ENSMUSG00000001995	signal-induced proliferation-associated 1 like 2(Sipa1l2)
64	ENSMUSG00000031461	myomesin 2(Myom2)
65	ENSMUSG00000027670	osteoclast stimulatory transmembrane protein(Ocstamp)
66	ENSMUSG00000096497	olfactory receptor 787(Olfr787)
67	ENSMUSG00000039328	ring finger protein 122(Rnf122)
68	ENSMUSG00000041961	zinc and ring finger 3(Znrf3)
69	ENSMUSG00000066684	paired immunoglobin-like type 2 receptor beta 1(Pilrb1)
70	ENSMUSG00000042286	stabilin 1(Stab1)
71	ENSMUSG00000033033	calcium homeostasis modulator 2(Calhm2)
72	ENSMUSG00000061171	solute carrier family 38, member 11(Slc38a11)
73	ENSMUSG00000009687	FXYD domain-containing ion transport regulator 5(Fxyd5)
74	ENSMUSG00000026712	mannose receptor, C type 1(Mrc1)
75	ENSMUSG00000042641	regulator of G-protein signaling like 1(Rgsl1)
76	ENSMUSG00000035004	immunoglobulin superfamily, member 6(Igsf6)
77	ENSMUSG00000025743	syndecan 3(Sdc3)
78	ENSMUSG00000070942	interleukin 1 receptor-like 2(Il1rl2)
79	ENSMUSG00000015314	SLAM family member 6(Slamf6)

80	ENSMUSG00000036634	myelin-associated glycoprotein(Mag)
81	ENSMUSG00000033569	adhesion G protein-coupled receptor B3(Adgrb3)
82	ENSMUSG00000045404	potassium channel, subfamily K, member 13(Kcnk13)
83	ENSMUSG00000040592	CD79B antigen(Cd79b)
84	ENSMUSG00000038179	SLAM family member 7(Slamf7)
85	ENSMUSG00000022971	interferon (alpha and beta) receptor 2(Ifnar2)
86	ENSMUSG00000036362	purinergic receptor P2Y, G-protein coupled 13(P2ry13)
87	ENSMUSG00000046031	family with sequence similarity 26, member F(Fam26f)
88	ENSMUSG00000030474	sialic acid binding Ig-like lectin E(Siglece)
89	ENSMUSG00000022901	CD86 antigen(Cd86)
90	ENSMUSG00000038147	CD84 antigen(Cd84)
91	ENSMUSG00000015396	CD83 antigen(Cd83)
92	ENSMUSG00000046006	Grb2-binding adaptor, transmembrane(Gapt)
93	ENSMUSG00000052821	cysteinyl leukotriene receptor 1(Cysltr1)
94	ENSMUSG00000095788	signal-regulatory protein beta 1A(Sirpb1a)
95	ENSMUSG00000028459	CD72 antigen(Cd72)
96	ENSMUSG00000092243	predicted gene 7030(Gm7030)
97	ENSMUSG00000018774	CD68 antigen(Cd68)
98	ENSMUSG00000021596	multiple C2 domains, transmembrane 1(Mctp1)
99	ENSMUSG00000073409	histocompatibility 2, Q region locus 6(H2-Q6)
100	ENSMUSG00000040747	CD53 antigen(Cd53)
101	ENSMUSG00000015355	CD48 antigen(Cd48)
102	ENSMUSG00000071424	glutamate receptor, ionotropic, delta 2(Grid2)
103	ENSMUSG00000013974	mast cell expressed membrane protein 1(Mcemp1)
104	ENSMUSG00000037306	mannosidase, alpha, class 1C, member 1(Man1c1)

105	ENSMUSG00000050350	G protein-coupled receptor 18(Gpr18)
106	ENSMUSG00000059030	olfactory receptor 128(Olfr128)
107	ENSMUSG00000022265	progressive ankylosis(Ank)
108	ENSMUSG00000046805	macrophage expressed gene 1(Mpeg1)
109	ENSMUSG00000000386	MX dynamin-like GTPase 1(Mx1)
110	ENSMUSG00000050103	alkylglycerol monooxygenase(Agmo)
111	ENSMUSG00000030798	CD37 antigen(Cd37)
112	ENSMUSG00000038295	autophagy related 9B(Atg9b)
113	ENSMUSG00000004609	CD33 antigen(Cd33)
114	ENSMUSG00000032777	general transcription factor III C 1(Gtf3c1)
115	ENSMUSG00000078771	ecotropic viral integration site 2a(Evi2a)
116	ENSMUSG00000003153	solute carrier family 2 (facilitated glucose transporter), member 3(Slc2a3)
117	ENSMUSG00000021624	CD180 antigen(Cd180)
118	ENSMUSG00000046718	bone marrow stromal cell antigen 2(Bst2)
119	ENSMUSG00000022747	ST3 beta-galactoside alpha-2,3-sialyltransferase 6(St3gal6)
120	ENSMUSG00000018920	chemokine (C-X-C motif) ligand 16(Cxcl16)
121	ENSMUSG00000000560	gamma-aminobutyric acid (GABA) A receptor, subunit alpha 2(Gabra2)
122	ENSMUSG00000028076	CD1d1 antigen(Cd1d1)
123	ENSMUSG00000028644	erythroblast membrane-associated protein(Ermap)
124	ENSMUSG00000000244	tetraspanin 32(Tspan32)
125	ENSMUSG00000024679	membrane-spanning 4-domains, subfamily A, member 6D(Ms4a6d)
126	ENSMUSG00000027435	CD93 antigen(Cd93)
127	ENSMUSG00000057191	cDNA sequence AB124611(AB124611)
128	ENSMUSG00000021087	reticulon 1(Rtn1)
129	ENSMUSG00000021097	calmin(Clmn)

130	ENSMUSG00000037913	transmembrane protein 156(Tmem156)
131	ENSMUSG00000030149	killer cell lectin-like receptor subfamily K, member 1(Klrk1)
132	ENSMUSG00000041552	patched domain containing 1(Ptchd1)
133	ENSMUSG00000049988	leucine rich repeat containing 25(Lrrc25)
134	ENSMUSG00000037405	intercellular adhesion molecule 1(Icam1)
135	ENSMUSG00000021886	G-protein coupled receptor 65(Gpr65)
136	ENSMUSG00000074825	inositol 1,4,5-triphosphate receptor interacting protein-like 1(Itpripl1)
137	ENSMUSG00000049307	fucosyltransferase 4(Fut4)
138	ENSMUSG00000047712	uronyl-2-sulfotransferase(Ust)
139	ENSMUSG00000027994	coiled-coil domain containing 109B(Ccdc109b)
140	ENSMUSG00000067599	killer cell lectin-like receptor, subfamily A, member 7(Klra7)
141	ENSMUSG00000049037	C-type lectin domain family 4, member a1(Clec4a1)
142	ENSMUSG00000030187	killer cell lectin-like receptor, subfamily A, member 2(Klra2)
143	ENSMUSG00000044288	cannabinoid receptor 1 (brain)(Cnr1)
144	ENSMUSG00000047759	heparan sulfate (glucosamine) 3-O-sulfotransferase 3A1(Hs3st3a1)
145	ENSMUSG00000056888	GLI pathogenesis-related 1 (glioma)(Glipr1)
146	ENSMUSG00000046650	olfactory receptor 1440(Olfr1440)
147	ENSMUSG00000095028	signal-regulatory protein beta 1B(Sirpb1b)
148	ENSMUSG00000053004	histamine receptor H1(Hrh1)
149	ENSMUSG00000034641	CD300 molecule like family member d(Cd300ld)
150	ENSMUSG00000040229	G protein-coupled receptor 34(Gpr34)
151	ENSMUSG00000063193	CD300 molecule like family member B(Cd300lb)
152	ENSMUSG00000032915	adhesion G protein-coupled receptor E4(Adgre4)
153	ENSMUSG00000034652	CD300A molecule(Cd300a)
154	ENSMUSG00000063715	olfactory receptor 816(Olfr816)

155	ENSMUSG00000048534	adhesion molecule, interacts with CXADR antigen 1(Amica1)
156	ENSMUSG00000073412	leukocyte specific transcript 1(Lst1)
157	ENSMUSG00000037646	vacuolar protein sorting 13B(Vps13b)
158	ENSMUSG00000079227	chemokine (C-C motif) receptor 5(Ccr5)
159	ENSMUSG00000068748	protein tyrosine phosphatase, receptor type Z, polypeptide 1(Ptprz1)
160	ENSMUSG00000049103	chemokine (C-C motif) receptor 2(Ccr2)
161	ENSMUSG00000035131	bone morphogenetic protein/retinoic acid inducible neural specific 3(Brinp3)
162	ENSMUSG00000013236	protein tyrosine phosphatase, receptor type, S(Ptprs)
163	ENSMUSG00000020101	V-set immunoregulatory receptor(Vsir)
164	ENSMUSG00000025804	chemokine (C-C motif) receptor 1(Ccr1)
165	ENSMUSG00000045382	chemokine (C-X-C motif) receptor 4(Cxcr4)
166	ENSMUSG00000030223	protein tyrosine phosphatase, receptor type, O(Ptpro)
167	ENSMUSG00000024349	transmembrane protein 173(Tmem173)
168	ENSMUSG00000045551	formyl peptide receptor 1(Fpr1)
169	ENSMUSG00000052613	protocadherin 15(Pcdh15)
170	ENSMUSG00000036944	transmembrane protein 71(Tmem71)
171	ENSMUSG00000079419	membrane-spanning 4-domains, subfamily A, member 6C(Ms4a6c)
172	ENSMUSG00000058099	Nfat activating molecule with ITAM motif 1(Nfam1)
173	ENSMUSG00000006411	nectin cell adhesion molecule 4(Nectin4)
174	ENSMUSG00000030263	lymphoid-restricted membrane protein(Lrmp)
175	ENSMUSG00000052270	formyl peptide receptor 2(Fpr2)
176	ENSMUSG00000041836	protein tyrosine phosphatase, receptor type, E(Ptpre)
177	ENSMUSG00000028399	protein tyrosine phosphatase, receptor type, D(Ptprd)
178	ENSMUSG00000027962	vascular cell adhesion molecule 1(Vcam1)
179	ENSMUSG00000074677	signal-regulatory protein beta 1-like(LOC100038947)

180	ENSMUSG00000037902	signal-regulatory protein alpha(Sirpa)
181	ENSMUSG00000086564	CD101 antigen(Cd101)
182	ENSMUSG00000024737	solute carrier family 15, member 3(Slc15a3)
183	ENSMUSG00000057135	SLP adaptor and CSK interacting membrane protein(Scimp)
184	ENSMUSG00000059791	nurim (nuclear envelope membrane protein)(Nrm)
185	ENSMUSG00000027947	interleukin 6 receptor, alpha(Il6ra)
186	ENSMUSG00000028599	tumor necrosis factor receptor superfamily, member 1b(Tnfrsf1b)
187	ENSMUSG00000045165	expressed sequence AI467606(AI467606)
188	ENSMUSG00000030147	C-type lectin domain family 4, member b1(Clec4b1)
189	ENSMUSG00000101389	membrane-spanning 4-domains, subfamily A, member 4A(Ms4a4a)
190	ENSMUSG00000067212	histocompatibility 2, T region locus 23(H2-T23)
191	ENSMUSG00000024331	desmocollin 2(Dsc2)
192	ENSMUSG00000031304	interleukin 2 receptor, gamma chain(Il2rg)
193	ENSMUSG00000029915	C-type lectin domain family 5, member a(Clec5a)
194	ENSMUSG00000034353	receptor (calcitonin) activity modifying protein 1(Ramp1)
195	ENSMUSG00000047798	CD300 molecule like family member F(Cd300lf)
196	ENSMUSG00000031129	solute carrier family 9 (sodium/hydrogen exchanger), member 9(Slc9a9)
197	ENSMUSG00000026073	interleukin 1 receptor, type II(Il1r2)
198	ENSMUSG00000022999	limb region 1 like(Lmbr1l)
199	ENSMUSG00000046245	paired immunoglobin-like type 2 receptor alpha(Pilra)
200	ENSMUSG00000000682	CD52 antigen(Cd52)
201	ENSMUSG00000040950	macrophage galactose N-acetyl-galactosamine specific lectin 2(Mgl2)
202	ENSMUSG00000020399	hepatitis A virus cellular receptor 2(Havcr2)
203	ENSMUSG00000046157	transmembrane protein 229B(Tmem229b)
204	ENSMUSG00000074342	RIKEN cDNA I830077J02 gene(I830077J02Rik)

205	ENSMUSG00000060550	histocompatibility 2, Q region locus 7(H2-Q7)
206	ENSMUSG00000055413	histocompatibility 2, Q region locus 5(H2-Q5)
207	ENSMUSG00000030148	C-type lectin domain family 4, member a2(Clec4a2)
208	ENSMUSG00000030089	solute carrier family 41, member 3(Slc41a3)
209	ENSMUSG00000035929	histocompatibility 2, Q region locus 4(H2-Q4)
210	ENSMUSG00000089722	CD300 molecule like family member D5(Cd300ld5)
211	ENSMUSG00000000318	C-type lectin domain family 10, member A(Clec10a)
212	ENSMUSG00000091705	histocompatibility 2, Q region locus 2(H2-Q2)
213	ENSMUSG00000069609	CD300 molecule like family member D4(Cd300ld4)
214	ENSMUSG00000074417	predicted gene 14548(Gm14548)
215	ENSMUSG00000073896	olfactory receptor 716(Olfr716)
216	ENSMUSG00000024672	membrane-spanning 4-domains, subfamily A, member 7(Ms4a7)
217	ENSMUSG00000033192	lysophosphatidylcholine acyltransferase 2(Lpcat2)
218	ENSMUSG00000079507	histocompatibility 2, Q region locus 1(H2-Q1)
219	ENSMUSG00000024677	membrane-spanning 4-domains, subfamily A, member 6B(Ms4a6b)
220	ENSMUSG00000032089	interleukin 10 receptor, alpha(Il10ra)
221	ENSMUSG00000051212	G protein-coupled receptor 183(Gpr183)
222	ENSMUSG00000035283	adrenergic receptor, beta 1(Adrb1)
223	ENSMUSG00000024334	histocompatibility 2, O region alpha locus(H2-Oa)
224	ENSMUSG00000053318	SLAM family member 8(Slamf8)
225	ENSMUSG00000037548	histocompatibility 2, class II, locus Mb2(H2-DMb2)
226	ENSMUSG00000079547	histocompatibility 2, class II, locus Mb1(H2-DMb1)
227	ENSMUSG00000037649	histocompatibility 2, class II, locus DMa(H2-DMa)
228	ENSMUSG00000036908	unc-93 homolog B1 (C. elegans)(Unc93b1)
229	ENSMUSG00000040751	linker for activation of T cells family, member 2(Lat2)

230	ENSMUSG00000051457	sialophorin(Spn)
231	ENSMUSG00000052384	negative regulator of reactive oxygen species(Nrros)
232	ENSMUSG00000059108	interferon induced transmembrane protein 6(Ifitm6)
233	ENSMUSG00000056529	platelet-activating factor receptor(Ptafr)
234	ENSMUSG00000016206	histocompatibility 2, M region locus 3(H2-M3)
235	ENSMUSG00000016283	histocompatibility 2, M region locus 2(H2-M2)
236	ENSMUSG00000070304	sodium channel, voltage-gated, type II, beta(Scn2b)
237	ENSMUSG00000025964	a disintegrin and metallopeptidase domain 23(Adam23)
238	ENSMUSG00000059336	solute carrier family 14 (urea transporter), member 1(Slc14a1)
239	ENSMUSG00000048163	selectin, platelet (p-selectin) ligand(Selplg)
240	ENSMUSG00000024754	transmembrane protein 2(Tmem2)
241	ENSMUSG00000020010	vanin 3(Vnn3)
242	ENSMUSG00000066682	paired immunoglobin-like type 2 receptor beta 2(Pilrb2)
243	ENSMUSG00000024675	membrane-spanning 4-domains, subfamily A, member 4C(Ms4a4c)
244	ENSMUSG00000061232	histocompatibility 2, K1, K region(H2-K1)
245	ENSMUSG00000055214	phospholipase D family, member 5(Pld5)
246	ENSMUSG00000060586	histocompatibility 2, class II antigen E beta(H2-Eb1)
247	ENSMUSG00000063434	sortilin-related VPS10 domain containing receptor 3(Sorcs3)
248	ENSMUSG00000040528	mast cell immunoglobulin like receptor 1(Milr1)
249	ENSMUSG00000052336	chemokine (C-X3-C motif) receptor 1(Cx3cr1)
250	ENSMUSG00000051504	sialic acid binding Ig-like lectin H(Siglech)
251	ENSMUSG00000073402	predicted gene 8909(Gm8909)
252	ENSMUSG00000053687	dipeptidase 2(Dpep2)
253	ENSMUSG00000073421	histocompatibility 2, class II antigen A, beta 1(H2-Ab1)
254	ENSMUSG00000036594	histocompatibility 2, class II antigen A, alpha(H2-Aa)

255	ENSMUSG00000051682	triggering receptor expressed on myeloid cells-like 4(Treml4)
256	ENSMUSG00000023169	solute carrier family 38, member 1(Slc38a1)
257	ENSMUSG00000040963	asialoglycoprotein receptor 2(Asgr2)
258	ENSMUSG00000049130	complement component 5a receptor 1(C5ar1)
259	ENSMUSG00000052337	inner membrane protein, mitochondrial(Immt)
260	ENSMUSG00000040552	complement component 3a receptor 1(C3ar1)
261	ENSMUSG00000042035	immunoglobulin superfamily, member 3(Igsf3)
262	ENSMUSG00000043067	dpy-19-like 1 (C. elegans)(Dpy19l1)
263	ENSMUSG00000074361	complement component 5a receptor 2(C5ar2)
264	ENSMUSG00000037408	cyclin M4(Cnnm4)
265	ENSMUSG00000055541	leukocyte-associated Ig-like receptor 1(Lair1)
**Gene Group 49**		**Enrichment Score: 3.63**
1	ENSMUSG00000025332	lysine (K)-specific demethylase 5C(Kdm5c)
2	ENSMUSG00000035021	bromodomain adjacent to zinc finger domain 1A(Baz1a)
3	ENSMUSG00000024201	lysine (K)-specific demethylase 4B(Kdm4b)
4	ENSMUSG00000038773	KDM3B lysine (K)-specific demethylase 3B(Kdm3b)
5	ENSMUSG00000030180	lysine (K)-specific demethylase 5A(Kdm5a)
6	ENSMUSG00000028397	lysine (K)-specific demethylase 4C(Kdm4c)
7	ENSMUSG00000042207	lysine (K)-specific demethylase 5B(Kdm5b)
8	ENSMUSG00000068245	PHD finger protein 11D(Phf11d)
**Gene Group 50**		**Enrichment Score: 2.78**
1	ENSMUSG00000029250	polymerase (RNA) II (DNA directed) polypeptide B(Polr2b)
2	ENSMUSG00000049553	polymerase (RNA) I polypeptide A(Polr1a)
3	ENSMUSG00000025280	polymerase (RNA) III (DNA directed) polypeptide A(Polr3a)
4	ENSMUSG00000027395	polymerase (RNA) I polypeptide B(Polr1b)
5	ENSMUSG00000067148	polymerase (RNA) I polypeptide C(Polr1c)

6	ENSMUSG00000034453	polymerase (RNA) III (DNA directed) polypeptide B(Polr3b)
**Gene Group 51**		**Enrichment Score: 2.62**
1	ENSMUSG00000090881	predicted gene 6904(Gm6904)
2	ENSMUSG00000091649	PHD finger protein 11B(Phf11b)
3	ENSMUSG00000068245	PHD finger protein 11D(Phf11d)
4	ENSMUSG00000091144	PHD finger protein 11C(Phf11c)
5	ENSMUSG00000044703	PHD finger protein 11A(Phf11a)
**Gene Group 52**		**Enrichment Score: 2.43**
1	ENSMUSG00000038543	cDNA sequence BC028528(BC028528)
2	ENSMUSG00000061100	resistin like alpha(Retnla)
3	ENSMUSG00000063297	leucine zipper protein 2(Luzp2)
4	ENSMUSG00000050786	coiled-coil domain containing 126(Ccdc126)
5	ENSMUSG00000021423	lymphocyte antigen 86(Ly86)
6	ENSMUSG00000039899	fibrinogen-like protein 2(Fgl2)
**Gene Group 53**		**Enrichment Score: 2.32**
1	ENSMUSG00000025964	a disintegrin and metallopeptidase domain 23(Adam23)
2	ENSMUSG00000025473	a disintegrin and metallopeptidase domain 8(Adam8)
3	ENSMUSG00000033453	a disintegrin-like and metallopeptidase (reprolysin type) with thrombospondin type 1 motif, 15(Adamts15)
4	ENSMUSG00000023903	matrix metallopeptidase 25(Mmp25)
**Gene Group 54**		**Enrichment Score: 2.23**
1	ENSMUSG00000021880	ribonuclease, RNase A family, 6(Rnase6)
2	ENSMUSG00000090166	eosinophil-associated, ribonuclease A family, member 10(Ear10)
3	ENSMUSG00000072596	eosinophil-associated, ribonuclease A family, member 2(Ear2)
4	ENSMUSG00000072601	eosinophil-associated, ribonuclease A family, member 1(Ear1)
**Gene Group 55**		**Enrichment Score: 1.61**
1	ENSMUSG00000021087	reticulon 1(Rtn1)
2	ENSMUSG00000028497	3-hydroxyacyl-CoA dehydratase 4(Hacd4)

3	ENSMUSG00000003484	cytochrome P450, family 4, subfamily f, polypeptide 18(Cyp4f18)
4	ENSMUSG00000032262	elongation of very long chain fatty acids (FEN1/Elo2, SUR4/Elo3, yeast)-like 4(Elovl4)
5	ENSMUSG00000050370	cholesterol 25-hydroxylase(Ch25h)
6	ENSMUSG00000050103	alkylglycerol monooxygenase(Agmo)
**Gene Group 56**		**Enrichment Score: 1.61**
1	ENSMUSG00000102037	B cell leukemia/lymphoma 2 related protein A1a(Bcl2a1a)
2	ENSMUSG00000099974	B cell leukemia/lymphoma 2 related protein A1d(Bcl2a1d)
3	ENSMUSG00000053820	B cell leukemia/lymphoma 2 related protein A1c(Bcl2a1c)
4	ENSMUSG00000089929	B cell leukemia/lymphoma 2 related protein A1b(Bcl2a1b)
**Gene Group 57**		**Enrichment Score: 1.41**
1	ENSMUSG00000045629	SH3 domain and tetratricopeptide repeats 2(Sh3tc2)
2	ENSMUSG00000024078	tetratricopeptide repeat domain 27(Ttc27)
3	ENSMUSG00000034848	tetratricopeptide repeat domain 21B(Ttc21b)
4	ENSMUSG00000032514	tetratricopeptide repeat domain 21A(Ttc21a)
**Gene Group 58**		**Enrichment Score: 1.03**
1	ENSMUSG00000041773	ectodermal-neural cortex 1(Enc1)
2	ENSMUSG00000043008	kelch-like 6(Klhl6)
3	ENSMUSG00000022750	kelch-like 22(Klhl22)
4	ENSMUSG00000020948	kelch-like 28(Klhl28)
5	ENSMUSG00000028696	IAP promoted placental gene(Ipp)
6	ENSMUSG00000070923	kelch-like 9(Klhl9)
7	ENSMUSG00000036782	kelch-like 13(Klhl13)
8	ENSMUSG00000043881	kelch repeat and BTB (POZ) domain containing 7(Kbtbd7)
**Gene Group 59**		**Enrichment Score: 0.97**
1	ENSMUSG00000058638	zinc finger protein 110(Zfp110)
2	ENSMUSG00000058331	zinc finger protein 85(Zfp85)
3	ENSMUSG00000038151	PR domain containing 1, with ZNF domain(Prdm1)

4	ENSMUSG00000068966	zinc finger and BTB domain containing 34(Zbtb34)
5	ENSMUSG00000039158	AT-hook transcription factor(Akna)
6	ENSMUSG00000051413	pleiomorphic adenoma gene-like 2(Plagl2)
7	ENSMUSG00000038872	zinc finger homeobox 3(Zfhx3)
8	ENSMUSG00000050240	hypermethylated in cancer 2(Hic2)
9	ENSMUSG00000074221	zinc finger protein 568(Zfp568)
10	ENSMUSG00000046658	zinc finger protein 316(Zfp316)
11	ENSMUSG00000025821	zinc finger protein 282(Zfp282)
12	ENSMUSG00000052040	Kruppel-like factor 13(Klf13)
13	ENSMUSG00000018654	IKAROS family zinc finger 1(Ikzf1)
14	ENSMUSG00000090641	zinc finger protein 712(Zfp712)
15	ENSMUSG00000036972	zinc finger protein of the cerebellum 4(Zic4)
16	ENSMUSG00000040829	zinc finger, MYND-type containing 15(Zmynd15)
17	ENSMUSG00000074220	zinc finger protein 382(Zfp382)

Functional Annotation Chart
Tool: DAVID
Input: 1448 macrophage translational signature genes
Criteria: DAVID: ≥ 1.5 fold enrichment, P-value < 0.05
Identified terms: 994
Note: sorted by P-value

Term	Category	Count	% of signature genes ass. with term	Fold Enrichment	P-Value
Phosphoprotein	UP_KEYWORDS	684	53,4	1,6	6,1E-54
Immunity	UP_KEYWORDS	115	9,0	5,1	7,4E-50
immune system process	GOTERM_BP_DIRECT	109	8,5	4,6	1,7E-42
Actin-binding	UP_KEYWORDS	83	6,5	5,9	6,6E-41
actin binding	GOTERM_MF_DIRECT	96	7,5	4,6	2,5E-37
Innate immunity	UP_KEYWORDS	72	5,6	5,4	2,0E-32
Cytoplasm	UP_KEYWORDS	408	31,9	1,7	2,6E-29
ATP-binding	UP_KEYWORDS	171	13,3	2,3	3,5E-24
P-loop containing nucleoside triphosphate hydrolase	INTERPRO	135	10,5	2,5	1,0E-23
Nucleotide-binding	UP_KEYWORDS	199	15,5	2,0	7,8E-23
ATP binding	GOTERM_MF_DIRECT	191	14,9	2,0	3,0E-22
innate immune response	GOTERM_BP_DIRECT	82	6,4	3,3	1,0E-21
actin filament binding	GOTERM_MF_DIRECT	44	3,4	5,3	3,1E-20
Calponin homology domain	INTERPRO	32	2,5	7,2	6,3E-19
Cytoskeleton	UP_KEYWORDS	133	10,4	2,2	2,2E-18
CH	SMART	31	2,4	6,2	5,8E-17

cytoskeleton	GOTERM_CC_DIRECT	140	10,9	2,1	7,2E-17
Alternative splicing	UP_KEYWORDS	388	30,3	1,5	1,6E-16
protein binding	GOTERM_MF_DIRECT	371	29,0	1,5	2,1E-16
Myosin head, motor domain	INTERPRO	22	1,7	9,6	2,5E-16
inflammatory response	GOTERM_BP_DIRECT	65	5,1	3,0	2,1E-15
brush border	GOTERM_CC_DIRECT	29	2,3	6,2	2,7E-15
chemotaxis	GOTERM_BP_DIRECT	36	2,8	4,9	3,0E-15
Actinin-type, actin-binding, conserved site	INTERPRO	17	1,3	12,6	3,0E-15
myosin complex	GOTERM_CC_DIRECT	24	1,9	7,6	5,3E-15
Staphylococcus aureus infection	KEGG_PATHWAY	25	2,0	6,6	1,4E-14
MYSc	SMART	22	1,7	7,4	4,2E-14
nucleotide binding	GOTERM_MF_DIRECT	202	15,8	1,7	7,0E-14
Inflammatory response	UP_KEYWORDS	38	3,0	4,2	1,0E-13
Myosin	UP_KEYWORDS	21	1,6	7,9	2,6E-13
nucleotide phosphate-binding region:ATP	UP_SEQ_FEATURE	118	9,2	2,0	4,1E-13
extracellular exosome	GOTERM_CC_DIRECT	250	19,5	1,5	5,9E-13
Spectrin/alpha-actinin	INTERPRO	17	1,3	10,0	5,9E-13
domain:CH 2	UP_SEQ_FEATURE	15	1,2	11,7	7,3E-13
domain:CH 1	UP_SEQ_FEATURE	15	1,2	11,7	7,3E-13
motor activity	GOTERM_MF_DIRECT	27	2,1	5,5	7,4E-13
domain:Myosin head-like	UP_SEQ_FEATURE	17	1,3	9,6	1,1E-12

WD40-repeat-containing domain	INTERPRO	54	4,2	3,0	1,3E-12
actin filament	GOTERM_CC_DIRECT	24	1,9	5,8	4,7E-12
Helicase	UP_KEYWORDS	31	2,4	4,3	2,0E-11
WD40/YVTN repeat-like-containing domain	INTERPRO	55	4,3	2,8	2,2E-11
SPEC	SMART	17	1,3	7,7	2,9E-11
helicase activity	GOTERM_MF_DIRECT	32	2,5	4,0	5,0E-11
Phagosome	KEGG_PATHWAY	41	3,2	3,2	5,5E-11
Motor protein	UP_KEYWORDS	30	2,3	4,2	6,8E-11
Immunoglobulin domain	UP_KEYWORDS	65	5,1	2,4	8,5E-11
Coiled coil	UP_KEYWORDS	252	19,7	1,5	8,9E-11
Adaptive immunity	UP_KEYWORDS	26	2,0	4,8	9,2E-11
Osteoclast differentiation	KEGG_PATHWAY	34	2,7	3,6	1,2E-10
Spectrin repeat	INTERPRO	14	1,1	9,5	2,5E-10
Pleckstrin homology domain	INTERPRO	45	3,5	2,9	2,6E-10
repeat:Spectrin 3	UP_SEQ_FEATURE	12	0,9	11,5	3,7E-10
Pleckstrin homology-like domain	INTERPRO	59	4,6	2,5	4,0E-10
Leishmaniasis	KEGG_PATHWAY	23	1,8	4,7	5,6E-10
repeat:Spectrin 4	UP_SEQ_FEATURE	11	0,9	12,8	5,6E-10
domain:Actin-binding	UP_SEQ_FEATURE	11	0,9	12,8	5,6E-10
Hydrolase	UP_KEYWORDS	151	11,8	1,6	9,5E-10
WD repeat	UP_KEYWORDS	42	3,3	2,9	9,7E-10

repeat:Spectrin 1	UP_SEQ_FEATURE	12	0,9	10,3	2,0E-9
repeat:Spectrin 2	UP_SEQ_FEATURE	12	0,9	10,3	2,0E-9
defense response to virus	GOTERM_BP_DIRECT	34	2,7	3,3	2,8E-9
Immunoglobulin C1-set	INTERPRO	25	2,0	4,2	3,0E-9
neutrophil chemotaxis	GOTERM_BP_DIRECT	21	1,6	4,9	4,1E-9
IQ motif, EF-hand binding site	INTERPRO	23	1,8	4,4	4,9E-9
C2 calcium-dependent membrane targeting	INTERPRO	31	2,4	3,4	7,4E-9
MHC classes I/II-like antigen recognition protein	INTERPRO	20	1,6	4,9	9,7E-9
Calmodulin-binding	UP_KEYWORDS	28	2,2	3,6	1,1E-8
Immunoglobulin/major histocompatibility complex, conserved site	INTERPRO	22	1,7	4,4	1,4E-8
Allosteric enzyme	UP_KEYWORDS	16	1,2	6,2	1,4E-8
IQ	SMART	19	1,5	4,9	1,5E-8
immune response	GOTERM_BP_DIRECT	44	3,4	2,6	1,6E-8
antigen processing and presentation of exogenous peptide antigen via MHC class II	GOTERM_BP_DIRECT	10	0,8	11,5	2,1E-8

adaptive immune response	GOTERM_BP_DIRECT	29	2,3	3,3	2,8E-8
Chemotaxis	UP_KEYWORDS	22	1,7	4,2	3,7E-8
ruffle	GOTERM_CC_DIRECT	23	1,8	4,0	4,0E-8
domain:PH	UP_SEQ_FEATURE	36	2,8	2,8	4,3E-8
cell surface	GOTERM_CC_DIRECT	74	5,8	1,9	6,8E-8
Immunoglobulin-like fold	INTERPRO	109	8,5	1,7	7,8E-8
carbohydrate binding	GOTERM_MF_DIRECT	38	3,0	2,7	8,9E-8
hydrolase activity	GOTERM_MF_DIRECT	147	11,5	1,5	1,0E-7
antigen processing and presentation	GOTERM_BP_DIRECT	17	1,3	5,0	1,0E-7
regulation of cell shape	GOTERM_BP_DIRECT	28	2,2	3,2	1,1E-7
domain:IQ 2	UP_SEQ_FEATURE	12	0,9	7,5	1,4E-7
SH3 domain	UP_KEYWORDS	33	2,6	2,8	1,7E-7
lamellipodium	GOTERM_CC_DIRECT	30	2,3	3,0	1,8E-7
IGc1	SMART	25	2,0	3,3	2,0E-7
actin-dependent ATPase activity	GOTERM_MF_DIRECT	9	0,7	11,1	2,2E-7
domain:IQ 1	UP_SEQ_FEATURE	12	0,9	7,3	2,2E-7
WD40 repeat	INTERPRO	39	3,0	2,5	2,6E-7
domain:IQ 4	UP_SEQ_FEATURE	8	0,6	13,1	3,2E-7
Lectin	UP_KEYWORDS	29	2,3	3,0	3,4E-7
domain:IQ 3	UP_SEQ_FEATURE	9	0,7	10,5	4,1E-7
PH	SMART	44	3,4	2,3	4,2E-7
Cell projection	UP_KEYWORDS	71	5,5	1,9	4,6E-7

region of interest:Actin-binding	UP_SEQ_FEATURE	11	0,9	7,5	6,2E-7
Immunoglobulin-like domain	INTERPRO	92	7,2	1,7	6,5E-7
cellular response to interferon-beta	GOTERM_BP_DIRECT	14	1,1	5,5	6,8E-7
Fc gamma R-mediated phagocytosis	KEGG_PATHWAY	22	1,7	3,5	6,9E-7
actin filament bundle assembly	GOTERM_BP_DIRECT	13	1,0	6,0	7,1E-7
Graft-versus-host disease	KEGG_PATHWAY	17	1,3	4,3	7,3E-7
actin filament polymerization	GOTERM_BP_DIRECT	11	0,9	7,3	7,5E-7
Rho GTPase activation protein	INTERPRO	20	1,6	3,8	7,8E-7
Tuberculosis	KEGG_PATHWAY	34	2,7	2,6	7,8E-7
catalytic activity	GOTERM_MF_DIRECT	59	4,6	2,0	9,0E-7
filamentous actin	GOTERM_CC_DIRECT	13	1,0	5,8	1,0E-6
stimulatory C-type lectin receptor signaling pathway	GOTERM_BP_DIRECT	7	0,5	14,0	1,5E-6
cytosol	GOTERM_CC_DIRECT	157	12,3	1,5	1,5E-6
podosome	GOTERM_CC_DIRECT	12	0,9	6,2	1,5E-6
MHC class II protein complex	GOTERM_CC_DIRECT	8	0,6	11,0	1,8E-6
microfilament motor activity	GOTERM_MF_DIRECT	9	0,7	9,0	1,8E-6

regulation of immune response	GOTERM_BP_DIRECT	12	0,9	6,0	2,0E-6
microvillus	GOTERM_CC_DIRECT	18	1,4	3,9	2,3E-6
SH2 domain	INTERPRO	22	1,7	3,3	2,3E-6
Src homology-3 domain	INTERPRO	32	2,5	2,6	2,5E-6
actin filament organization	GOTERM_BP_DIRECT	19	1,5	3,7	2,7E-6
actin filament-based movement	GOTERM_BP_DIRECT	9	0,7	8,5	3,3E-6
AAA+ ATPase domain	INTERPRO	25	2,0	3,0	3,4E-6
Influenza A	KEGG_PATHWAY	32	2,5	2,5	3,6E-6
repeat:Spectrin 5	UP_SEQ_FEATURE	7	0,5	12,7	3,7E-6
Immunoglobulin subtype	INTERPRO	58	4,5	1,9	3,8E-6
DNA replication	UP_KEYWORDS	19	1,5	3,6	4,3E-6
actin cytoskeleton	GOTERM_CC_DIRECT	31	2,4	2,5	4,4E-6
beta-2-microglobulin binding	GOTERM_MF_DIRECT	8	0,6	9,9	4,4E-6
C-type lectin-like	INTERPRO	24	1,9	3,0	4,7E-6
SH2 domain	UP_KEYWORDS	20	1,6	3,4	4,8E-6
domain:Ig-like C1-type	UP_SEQ_FEATURE	12	0,9	5,4	6,2E-6
ATPase activity	GOTERM_MF_DIRECT	31	2,4	2,5	6,8E-6
Antigen processing and presentation	KEGG_PATHWAY	20	1,6	3,2	7,8E-6

nuclear pore outer ring	GOTERM_CC_DIRECT	7	0,5	11,5	8,3E-6
T cell receptor binding	GOTERM_MF_DIRECT	8	0,6	9,2	8,4E-6
cell-cell adherens junction	GOTERM_CC_DIRECT	41	3,2	2,1	8,4E-6
calmodulin binding	GOTERM_MF_DIRECT	29	2,3	2,6	8,4E-6
stress fiber	GOTERM_CC_DIRECT	16	1,2	3,9	8,8E-6
cortical cytoskeleton	GOTERM_CC_DIRECT	11	0,9	5,8	8,9E-6
external side of plasma membrane	GOTERM_CC_DIRECT	41	3,2	2,1	9,0E-6
Chemokine signaling pathway	KEGG_PATHWAY	34	2,7	2,3	9,1E-6
Type I diabetes mellitus	KEGG_PATHWAY	17	1,3	3,6	9,4E-6
C-type lectin	INTERPRO	22	1,7	3,0	1,1E-5
MHC II	UP_KEYWORDS	7	0,5	11,4	1,1E-5
Allograft rejection	KEGG_PATHWAY	16	1,2	3,8	1,1E-5
C-type lectin fold	INTERPRO	24	1,9	2,8	1,2E-5
focal adhesion	GOTERM_CC_DIRECT	47	3,7	2,0	1,3E-5
Herpes simplex infection	KEGG_PATHWAY	35	2,7	2,2	1,3E-5
Plectin repeat	INTERPRO	6	0,5	14,6	1,3E-5
positive regulation of peptidyl-tyrosine phosphorylation	GOTERM_BP_DIRECT	20	1,6	3,2	1,3E-5
peptide antigen binding	GOTERM_MF_DIRECT	13	1,0	4,6	1,4E-5

mutagenesis site	UP_SEQ_FEATURE	78	6,1	1,7	1,4E-5
MHC class II, alpha/beta chain, N-terminal	INTERPRO	7	0,5	10,8	1,4E-5
cellular response to interferon-gamma	GOTERM_BP_DIRECT	16	1,2	3,8	1,5E-5
domain:IQ 5	UP_SEQ_FEATURE	6	0,5	14,0	1,6E-5
Viral myocarditis	KEGG_PATHWAY	19	1,5	3,2	1,7E-5
actomyosin	GOTERM_CC_DIRECT	7	0,5	10,5	1,7E-5
cell cortex	GOTERM_CC_DIRECT	24	1,9	2,7	1,8E-5
MHC class I protein complex	GOTERM_CC_DIRECT	8	0,6	8,2	2,1E-5
Calcium	UP_KEYWORDS	76	5,9	1,7	2,1E-5
actin cytoskeleton organization	GOTERM_BP_DIRECT	24	1,9	2,7	2,3E-5
Helicase, superfamily 1/2, ATP-binding domain	INTERPRO	20	1,6	3,1	2,3E-5
WD40 repeat, conserved site	INTERPRO	25	2,0	2,6	2,4E-5
domain:CH	UP_SEQ_FEATURE	12	0,9	4,8	2,4E-5
cell projection	GOTERM_CC_DIRECT	72	5,6	1,7	2,4E-5
domain:Ig-like V-type	UP_SEQ_FEATURE	20	1,6	3,0	2,7E-5
positive regulation of cytokine secretion	GOTERM_BP_DIRECT	11	0,9	5,2	2,8E-5
repeat:WD 4	UP_SEQ_FEATURE	32	2,5	2,3	2,9E-5

cell-cell junction	GOTERM_CC_DIRECT	29	2,3	2,4	3,3E-5
Myosin tail 2	INTERPRO	6	0,5	12,8	3,3E-5
repeat:WD 3	UP_SEQ_FEATURE	33	2,6	2,2	3,4E-5
Antiviral defense	UP_KEYWORDS	18	1,4	3,2	3,5E-5
repeat:WD 5	UP_SEQ_FEATURE	30	2,3	2,3	3,6E-5
GTPase activation	UP_KEYWORDS	24	1,9	2,6	3,7E-5
response to bacterium	GOTERM_BP_DIRECT	11	0,9	5,0	3,7E-5
peptidyl-tyrosine phosphorylation	GOTERM_BP_DIRECT	15	1,2	3,7	3,8E-5
actin crosslink formation	GOTERM_BP_DIRECT	7	0,5	9,4	3,9E-5
extrinsic component of external side of plasma membrane	GOTERM_CC_DIRECT	6	0,5	12,4	3,9E-5
phosphatidylinositol phospholipase C activity	GOTERM_MF_DIRECT	8	0,6	7,5	4,0E-5
repeat:Spectrin 6	UP_SEQ_FEATURE	6	0,5	12,3	4,1E-5
MHC class I, alpha chain, alpha1/alpha2	INTERPRO	12	0,9	4,5	4,4E-5
TAP binding	GOTERM_MF_DIRECT	6	0,5	12,0	4,5E-5
PLEC	SMART	6	0,5	11,3	4,6E-5
B cell receptor signaling pathway	KEGG_PATHWAY	17	1,3	3,2	4,9E-5

antigen processing and presentation of peptide antigen via MHC class I	GOTERM_BP_DIRECT	11	0,9	4,9	4,9E-5
Viral carcinogenesis	KEGG_PATHWAY	36	2,8	2,1	5,1E-5
filopodium	GOTERM_CC_DIRECT	16	1,2	3,4	5,1E-5
repeat:WD 1	UP_SEQ_FEATURE	33	2,6	2,2	5,1E-5
repeat:WD 2	UP_SEQ_FEATURE	33	2,6	2,2	5,1E-5
Toll-like receptor signaling pathway	KEGG_PATHWAY	21	1,6	2,7	5,3E-5
metabolic process	GOTERM_BP_DIRECT	52	4,1	1,8	5,4E-5
defense response to Gram-positive bacterium	GOTERM_BP_DIRECT	18	1,4	3,1	5,5E-5
defense response to protozoan	GOTERM_BP_DIRECT	10	0,8	5,3	6,0E-5
Leukocyte transendothelial migration	KEGG_PATHWAY	23	1,8	2,6	6,0E-5
Helicase, C-terminal	INTERPRO	19	1,5	3,0	6,2E-5
Nuclear pore complex	UP_KEYWORDS	11	0,9	4,8	6,3E-5
phagocytosis	GOTERM_BP_DIRECT	13	1,0	4,0	6,5E-5
chain:PHD finger protein 11	UP_SEQ_FEATURE	5	0,4	16,3	6,6E-5
chain:PHD finger protein 11-like	UP_SEQ_FEATURE	5	0,4	16,3	6,6E-5

region of interest:Connecting peptide	UP_SEQ_FEATURE	11	0,9	4,7	6,8E-5
ATPase, AAA-4	INTERPRO	6	0,5	11,3	7,1E-5
Gelsolin domain	INTERPRO	7	0,5	8,5	8,1E-5
SH2	SMART	21	1,6	2,7	8,1E-5
WD40	SMART	39	3,0	2,0	8,1E-5
MCM complex	GOTERM_CC_DIRECT	6	0,5	11,0	8,3E-5
phagocytic vesicle membrane	GOTERM_CC_DIRECT	12	0,9	4,2	8,9E-5
perinuclear region of cytoplasm	GOTERM_CC_DIRECT	68	5,3	1,6	9,9E-5
domain:PI-PLC Y-box	UP_SEQ_FEATURE	7	0,5	8,2	1,0E-4
nuclear pore	GOTERM_CC_DIRECT	14	1,1	3,6	1,0E-4
positive regulation of inflammatory response	GOTERM_BP_DIRECT	14	1,1	3,6	1,1E-4
MHC class I-like antigen recognition	INTERPRO	13	1,0	3,8	1,1E-4
lysosome	GOTERM_CC_DIRECT	39	3,0	1,9	1,2E-4
region of interest:Alpha-1	UP_SEQ_FEATURE	8	0,6	6,5	1,2E-4
region of interest:Alpha-2	UP_SEQ_FEATURE	8	0,6	6,5	1,2E-4
Phosphoinositide phospholipase C	INTERPRO	7	0,5	7,9	1,3E-4
Phospholipase C, phosphatidylinositol-specific, Y domain	INTERPRO	7	0,5	7,9	1,3E-4
cytoplasmic vesicle	GOTERM_CC_DIRECT	64	5,0	1,6	1,3E-4

monocyte chemotaxis	GOTERM_BP_DIRECT	11	0,9	4,4	1,3E-4
positive regulation of T cell proliferation	GOTERM_BP_DIRECT	14	1,1	3,5	1,3E-4
domain:SH2	UP_SEQ_FEATURE	17	1,3	3,0	1,5E-4
repeat:WD 9	UP_SEQ_FEATURE	10	0,8	4,8	1,5E-4
positive regulation of cell migration	GOTERM_BP_DIRECT	28	2,2	2,2	1,6E-4
Mini-chromosome maintenance, conserved site	INTERPRO	5	0,4	14,2	1,6E-4
C2	SMART	23	1,8	2,4	1,7E-4
positive regulation of type I interferon production	GOTERM_BP_DIRECT	7	0,5	7,5	1,8E-4
intracellular signal transduction	GOTERM_BP_DIRECT	45	3,5	1,8	1,8E-4
Inositol phosphate metabolism	KEGG_PATHWAY	16	1,2	3,0	1,8E-4
Cell cycle	UP_KEYWORDS	58	4,5	1,7	1,8E-4
Quinonprotein alcohol dehydrogenase-like superfamily	INTERPRO	11	0,9	4,3	1,9E-4
zinc finger region:PHD-type; degenerate	UP_SEQ_FEATURE	5	0,4	13,6	1,9E-4
SH3	SMART	31	2,4	2,1	1,9E-4

Epstein-Barr virus infection	KEGG_PATHWAY	24	1,9	2,3	1,9E-4
Cytoplasmic vesicle	UP_KEYWORDS	48	3,7	1,8	1,9E-4
cortical actin cytoskeleton	GOTERM_CC_DIRECT	11	0,9	4,2	2,0E-4
AAA	SMART	25	2,0	2,3	2,1E-4
Autoimmune thyroid disease	KEGG_PATHWAY	16	1,2	3,0	2,1E-4
DNA replication	GOTERM_BP_DIRECT	20	1,6	2,6	2,1E-4
Cell junction	UP_KEYWORDS	60	4,7	1,6	2,4E-4
domain:PI-PLC X-box	UP_SEQ_FEATURE	7	0,5	7,2	2,4E-4
growth cone	GOTERM_CC_DIRECT	23	1,8	2,4	2,5E-4
regulation of cell migration	GOTERM_BP_DIRECT	15	1,2	3,1	2,6E-4
Nucleotidyltransferase	UP_KEYWORDS	14	1,1	3,3	2,7E-4
positive regulation of interleukin-1 beta secretion	GOTERM_BP_DIRECT	8	0,6	5,8	2,7E-4
sister chromatid cohesion	GOTERM_BP_DIRECT	7	0,5	7,0	2,7E-4
Rho GTPase binding	GOTERM_MF_DIRECT	10	0,8	4,5	2,8E-4
Natural killer cell mediated cytotoxicity	KEGG_PATHWAY	20	1,6	2,5	2,8E-4
domain:C2	UP_SEQ_FEATURE	14	1,1	3,3	2,8E-4
Phosphatidylinositol signaling system	KEGG_PATHWAY	19	1,5	2,6	2,9E-4
NF-kappa B signaling pathway	KEGG_PATHWAY	19	1,5	2,6	2,9E-4

cell chemotaxis	GOTERM_BP_DIRECT	15	1,2	3,1	3,0E-4
actin cytoskeleton reorganization	GOTERM_BP_DIRECT	12	0,9	3,7	3,0E-4
positive regulation of interferon-gamma production	GOTERM_BP_DIRECT	12	0,9	3,7	3,0E-4
intraciliary retrograde transport	GOTERM_BP_DIRECT	6	0,5	8,7	3,1E-4
phospholipid binding	GOTERM_MF_DIRECT	16	1,2	2,9	3,3E-4
double-stranded RNA binding	GOTERM_MF_DIRECT	14	1,1	3,2	3,4E-4
GTPase activator activity	GOTERM_MF_DIRECT	30	2,3	2,0	3,4E-4
Zinc finger, N-recognin	INTERPRO	5	0,4	12,2	3,6E-4
Carbamoyl-phosphate synthetase large subunit-like, ATP-binding domain	INTERPRO	5	0,4	12,2	3,6E-4
phagocytosis, engulfment	GOTERM_BP_DIRECT	11	0,9	3,9	3,7E-4
cytokine receptor activity	GOTERM_MF_DIRECT	11	0,9	3,9	3,7E-4
toll-like receptor signaling pathway	GOTERM_BP_DIRECT	7	0,5	6,6	3,9E-4
CLECT	SMART	22	1,7	2,3	4,0E-4
HTLV-I infection	KEGG_PATHWAY	38	3,0	1,8	4,1E-4

CC chemokine, conserved site	INTERPRO	7	0,5	6,6	4,1E-4
Rho GTPase-activating protein domain	INTERPRO	13	1,0	3,4	4,1E-4
repeat:Spectrin 8	UP_SEQ_FEATURE	5	0,4	11,7	4,2E-4
repeat:Gelsolin-like 5	UP_SEQ_FEATURE	5	0,4	11,7	4,2E-4
repeat:Spectrin 14	UP_SEQ_FEATURE	5	0,4	11,7	4,2E-4
repeat:Spectrin 11	UP_SEQ_FEATURE	5	0,4	11,7	4,2E-4
domain:HIN-200	UP_SEQ_FEATURE	5	0,4	11,7	4,2E-4
repeat:Spectrin 10	UP_SEQ_FEATURE	5	0,4	11,7	4,2E-4
zinc finger region:UBR-type	UP_SEQ_FEATURE	5	0,4	11,7	4,2E-4
repeat:Spectrin 16	UP_SEQ_FEATURE	5	0,4	11,7	4,2E-4
repeat:Spectrin 7	UP_SEQ_FEATURE	5	0,4	11,7	4,2E-4
repeat:Spectrin 12	UP_SEQ_FEATURE	5	0,4	11,7	4,2E-4
repeat:Spectrin 17	UP_SEQ_FEATURE	5	0,4	11,7	4,2E-4
repeat:Gelsolin-like 4	UP_SEQ_FEATURE	5	0,4	11,7	4,2E-4
repeat:Spectrin 15	UP_SEQ_FEATURE	5	0,4	11,7	4,2E-4
repeat:Spectrin 9	UP_SEQ_FEATURE	5	0,4	11,7	4,2E-4
repeat:Spectrin 13	UP_SEQ_FEATURE	5	0,4	11,7	4,2E-4
Guanine-nucleotide releasing factor	UP_KEYWORDS	19	1,5	2,6	4,2E-4
domain:Ig-like C2-type 1	UP_SEQ_FEATURE	20	1,6	2,5	4,2E-4
Cell adhesion molecules (CAMs)	KEGG_PATHWAY	26	2,0	2,1	4,5E-4

positive regulation of B cell receptor signaling pathway	GOTERM_BP_DIRECT	5	0,4	11,5	4,5E-4
repeat:WD 8	UP_SEQ_FEATURE	11	0,9	3,8	4,5E-4
Fc epsilon RI signaling pathway	KEGG_PATHWAY	15	1,2	2,9	4,6E-4
domain:Ig-like C2-type 2	UP_SEQ_FEATURE	20	1,6	2,5	4,6E-4
MHC I	UP_KEYWORDS	6	0,5	8,3	4,7E-4
chemokine-mediated signaling pathway	GOTERM_BP_DIRECT	12	0,9	3,5	5,0E-4
B cell receptor signaling pathway	GOTERM_BP_DIRECT	12	0,9	3,5	5,0E-4
domain:SH3	UP_SEQ_FEATURE	23	1,8	2,3	5,0E-4
glucose binding	GOTERM_MF_DIRECT	6	0,5	8,0	5,1E-4
PLCYc	SMART	7	0,5	6,1	5,2E-4
actin filament capping	GOTERM_BP_DIRECT	7	0,5	6,2	5,6E-4
microglial cell activation	GOTERM_BP_DIRECT	7	0,5	6,2	5,6E-4
DNA helicase activity	GOTERM_MF_DIRECT	7	0,5	6,2	5,6E-4
Phospholipase C, phosphatidylinositol-specific , X domain	INTERPRO	7	0,5	6,3	5,7E-4
Myosin tail	INTERPRO	7	0,5	6,3	5,7E-4
DEXDc	SMART	20	1,6	2,4	5,8E-4
cellular response to lipopolysaccharide	GOTERM_BP_DIRECT	27	2,1	2,1	6,0E-4

Ras GTPase-activating protein, conserved site	INTERPRO	6	0,5	7,9	6,0E-4
membrane raft	GOTERM_CC_DIRECT	31	2,4	2,0	6,1E-4
chemokine activity	GOTERM_MF_DIRECT	11	0,9	3,7	6,3E-4
cadherin binding involved in cell-cell adhesion	GOTERM_MF_DIRECT	33	2,6	1,9	6,4E-4
negative regulation of T cell proliferation	GOTERM_BP_DIRECT	10	0,8	4,0	6,5E-4
positive regulation of ERK1 and ERK2 cascade	GOTERM_BP_DIRECT	25	2,0	2,1	6,6E-4
C-type lectin, conserved site	INTERPRO	11	0,9	3,7	6,6E-4
endoplasmic reticulum exit site	GOTERM_CC_DIRECT	7	0,5	6,1	6,7E-4
cellular response to organic cyclic compound	GOTERM_BP_DIRECT	14	1,1	3,0	6,8E-4
2-5-oligoadenylate synthetase, conserved site	INTERPRO	5	0,4	10,6	6,8E-4
Villin/Gelsolin	INTERPRO	5	0,4	10,6	6,8E-4
Phosphatidylinositol 3-kinase C2 (PI3K C2) domain	INTERPRO	5	0,4	10,6	6,8E-4

repeat:WD 7	UP_SEQ_FEATURE	19	1,5	2,4	7,1E-4
lymphocyte chemotaxis	GOTERM_BP_DIRECT	9	0,7	4,4	7,6E-4
Biotin-binding site	INTERPRO	4	0,3	17,0	7,7E-4
Bcl-2-related protein A1	INTERPRO	4	0,3	17,0	7,7E-4
intraciliary transport particle A	GOTERM_CC_DIRECT	5	0,4	10,3	7,7E-4
basal plasma membrane	GOTERM_CC_DIRECT	10	0,8	3,9	7,8E-4
immunological synapse	GOTERM_CC_DIRECT	9	0,7	4,4	7,9E-4
positive regulation of tumor necrosis factor biosynthetic process	GOTERM_BP_DIRECT	6	0,5	7,4	7,9E-4
repeat:Gelsolin-like 3	UP_SEQ_FEATURE	5	0,4	10,2	8,0E-4
repeat:Gelsolin-like 2	UP_SEQ_FEATURE	5	0,4	10,2	8,0E-4
repeat:Gelsolin-like 1	UP_SEQ_FEATURE	5	0,4	10,2	8,0E-4
domain:MCM	UP_SEQ_FEATURE	5	0,4	10,2	8,0E-4
B cell homeostasis	GOTERM_BP_DIRECT	8	0,6	4,9	8,3E-4
response to interferon-gamma	GOTERM_BP_DIRECT	8	0,6	4,9	8,3E-4
regulation of mast cell degranulation	GOTERM_BP_DIRECT	5	0,4	10,0	8,6E-4
ruffle membrane	GOTERM_CC_DIRECT	14	1,1	2,9	8,8E-4
Intestinal immune network for IgA production	KEGG_PATHWAY	11	0,9	3,5	9,0E-4

DNA repair	UP_KEYWORDS	30	2,3	1,9	9,2E-4
toll-like receptor 7 signaling pathway	GOTERM_BP_DIRECT	4	0,3	16,0	9,2E-4
positive regulation of type III hypersensitivity	GOTERM_BP_DIRECT	4	0,3	16,0	9,2E-4
antigen processing and presentation of peptide antigen	GOTERM_BP_DIRECT	4	0,3	16,0	9,2E-4
late endosome	GOTERM_CC_DIRECT	19	1,5	2,4	9,4E-4
ZnF_UBR1	SMART	5	0,4	9,4	9,7E-4
Immunoglobulin	INTERPRO	12	0,9	3,2	1,0E-3
cell cycle	GOTERM_BP_DIRECT	59	4,6	1,5	1,0E-3
receptor-mediated endocytosis	GOTERM_BP_DIRECT	12	0,9	3,2	1,1E-3
domain:C-type lectin	UP_SEQ_FEATURE	16	1,2	2,6	1,1E-3
Rheumatoid arthritis	KEGG_PATHWAY	16	1,2	2,6	1,1E-3
ligase activity	GOTERM_MF_DIRECT	39	3,0	1,7	1,2E-3
Mini-chromosome maintenance, DNA-dependent ATPase	INTERPRO	5	0,4	9,5	1,2E-3
Transcription factor jumonji, JmjN	INTERPRO	5	0,4	9,5	1,2E-3
HELICc	SMART	19	1,5	2,3	1,2E-3
Kinase	UP_KEYWORDS	60	4,7	1,5	1,2E-3

Measles	KEGG_PATHWAY	22	1,7	2,1	1,2E-3
Phosphatidylinositol 3/4-kinase, conserved site	INTERPRO	6	0,5	6,8	1,3E-3
Mitosis	UP_KEYWORDS	28	2,2	1,9	1,3E-3
cell junction	GOTERM_CC_DIRECT	65	5,1	1,5	1,3E-3
repeat:WD 6	UP_SEQ_FEATURE	22	1,7	2,2	1,3E-3
actin filament bundle	GOTERM_CC_DIRECT	5	0,4	9,2	1,3E-3
positive regulation of neutrophil chemotaxis	GOTERM_BP_DIRECT	8	0,6	4,6	1,3E-3
DNA-dependent ATPase activity	GOTERM_MF_DIRECT	8	0,6	4,6	1,3E-3
ATP-dependent helicase activity	GOTERM_MF_DIRECT	8	0,6	4,6	1,3E-3
domain:JmjN	UP_SEQ_FEATURE	5	0,4	9,1	1,4E-3
positive regulation of natural killer cell mediated cytotoxicity	GOTERM_BP_DIRECT	7	0,5	5,3	1,4E-3
phosphatidylinositol-mediated signaling	GOTERM_BP_DIRECT	9	0,7	4,0	1,4E-3
Polymorphism	UP_KEYWORDS	24	1,9	2,1	1,4E-3
toll-like receptor 3 signaling pathway	GOTERM_BP_DIRECT	5	0,4	8,9	1,5E-3

antigen processing and presentation of peptide or polysaccharide antigen via MHC class II	GOTERM_BP_DIRECT	5	0,4	8,9	1,5E-3
Asthma	KEGG_PATHWAY	8	0,6	4,4	1,5E-3
protein complex	GOTERM_CC_DIRECT	58	4,5	1,5	1,5E-3
mast cell granule	GOTERM_CC_DIRECT	7	0,5	5,2	1,6E-3
PLCXc	SMART	7	0,5	5,1	1,6E-3
guanyl-nucleotide exchange factor activity	GOTERM_MF_DIRECT	21	1,6	2,2	1,7E-3
Ras GTPase-activating protein	INTERPRO	6	0,5	6,4	1,8E-3
GEL	SMART	5	0,4	8,2	1,8E-3
PI3K_C2	SMART	5	0,4	8,2	1,8E-3
myeloid dendritic cell differentiation	GOTERM_BP_DIRECT	7	0,5	5,1	1,8E-3
leukocyte chemotaxis	GOTERM_BP_DIRECT	7	0,5	5,1	1,8E-3
ATP-dependent DNA helicase activity	GOTERM_MF_DIRECT	7	0,5	5,1	1,8E-3
Carbamoyl-phosphate synthase, large subunit, N-terminal	INTERPRO	4	0,3	13,6	1,8E-3
Biotin carboxylation domain	INTERPRO	4	0,3	13,6	1,8E-3

Biotin carboxylase, C-terminal	INTERPRO	4	0,3	13,6	1,8E-3
Biotin/lipoyl attachment	INTERPRO	5	0,4	8,5	1,9E-3
Phosphatidylinositol Kinase	INTERPRO	5	0,4	8,5	1,9E-3
Phosphoinositide 3-kinase, accessory (PIK) domain	INTERPRO	5	0,4	8,5	1,9E-3
Formyl peptide receptor family	INTERPRO	5	0,4	8,5	1,9E-3
HIN-200/IF120x	INTERPRO	5	0,4	8,5	1,9E-3
Actin capping	UP_KEYWORDS	6	0,5	6,3	1,9E-3
RasGAP	SMART	6	0,5	6,1	1,9E-3
cytoskeleton organization	GOTERM_BP_DIRECT	16	1,2	2,5	1,9E-3
FERM central domain	INTERPRO	10	0,8	3,5	2,0E-3
Band 4.1 domain	INTERPRO	10	0,8	3,5	2,0E-3
Protein-tyrosine phosphatase, receptor/non-receptor type	INTERPRO	9	0,7	3,8	2,0E-3
Cell adhesion	UP_KEYWORDS	42	3,3	1,6	2,0E-3
NLRP3 inflammasome complex	GOTERM_CC_DIRECT	4	0,3	13,2	2,0E-3
platelet aggregation	GOTERM_BP_DIRECT	9	0,7	3,8	2,0E-3

mitotic cytokinesis	GOTERM_BP_DIRECT	8	0,6	4,3	2,1E-3
domain:Biotinyl-binding	UP_SEQ_FEATURE	4	0,3	13,1	2,1E-3
repeat:Plectin 2	UP_SEQ_FEATURE	4	0,3	13,1	2,1E-3
repeat:Plectin 1	UP_SEQ_FEATURE	4	0,3	13,1	2,1E-3
repeat:TPR 14	UP_SEQ_FEATURE	4	0,3	13,1	2,1E-3
repeat:TPR 13	UP_SEQ_FEATURE	4	0,3	13,1	2,1E-3
domain:Biotin carboxylation	UP_SEQ_FEATURE	4	0,3	13,1	2,1E-3
Starch and sucrose metabolism	KEGG_PATHWAY	9	0,7	3,7	2,1E-3
domain:Ras-GAP	UP_SEQ_FEATURE	5	0,4	8,2	2,2E-3
repeat:TPR 11	UP_SEQ_FEATURE	5	0,4	8,2	2,2E-3
cellular response to diacyl bacterial lipopeptide	GOTERM_BP_DIRECT	4	0,3	12,8	2,2E-3
negative regulation of B cell receptor signaling pathway	GOTERM_BP_DIRECT	4	0,3	12,8	2,2E-3
antigen processing and presentation of exogenous peptide antigen via MHC class I	GOTERM_BP_DIRECT	4	0,3	12,8	2,2E-3
lipopeptide binding	GOTERM_MF_DIRECT	4	0,3	12,8	2,2E-3
biotin carboxylase activity	GOTERM_MF_DIRECT	4	0,3	12,8	2,2E-3

Toll-like receptor binding	GOTERM_MF_DIRECT	4	0,3	12,8	2,2E-3
Nucleic acid-binding, OB-fold	INTERPRO	14	1,1	2,6	2,2E-3
Chemokine interleukin-8-like domain	INTERPRO	10	0,8	3,4	2,3E-3
ruffle organization	GOTERM_BP_DIRECT	6	0,5	6,0	2,3E-3
cellular response to ethanol	GOTERM_BP_DIRECT	6	0,5	6,0	2,3E-3
MHC class I protein binding	GOTERM_MF_DIRECT	6	0,5	6,0	2,3E-3
Thiamine pyrophosphate	UP_KEYWORDS	5	0,4	8,2	2,3E-3
Endocytosis	UP_KEYWORDS	16	1,2	2,4	2,3E-3
4Fe-4S	UP_KEYWORDS	8	0,6	4,2	2,3E-3
glycogen catabolic process	GOTERM_BP_DIRECT	5	0,4	8,0	2,3E-3
DNA unwinding involved in DNA replication	GOTERM_BP_DIRECT	5	0,4	8,0	2,3E-3
Valine, leucine and isoleucine degradation	KEGG_PATHWAY	12	0,9	2,9	2,3E-3
Domain of unknown function DUF1605	INTERPRO	6	0,5	6,0	2,4E-3
inner ear development	GOTERM_BP_DIRECT	12	0,9	2,9	2,4E-3
kinetochore	GOTERM_CC_DIRECT	17	1,3	2,3	2,4E-3

phosphatidylinositol-3,4,5-trisphosphate binding	GOTERM_MF_DIRECT	9	0,7	3,7	2,4E-3
domain:Helicase ATP-binding	UP_SEQ_FEATURE	15	1,2	2,5	2,5E-3
lipopolysaccharide-mediated signaling pathway	GOTERM_BP_DIRECT	8	0,6	4,1	2,5E-3
Chromosome partition	UP_KEYWORDS	9	0,7	3,7	2,7E-3
Cytosolic DNA-sensing pathway	KEGG_PATHWAY	13	1,0	2,7	2,7E-3
Regulation of actin cytoskeleton	KEGG_PATHWAY	29	2,3	1,8	2,7E-3
protein tyrosine phosphatase activity	GOTERM_MF_DIRECT	15	1,2	2,5	2,7E-3
Golgi medial cisterna	GOTERM_CC_DIRECT	6	0,5	5,8	2,7E-3
PLC-like phosphodiesterase, TIM beta/alpha-barrel domain	INTERPRO	7	0,5	4,8	2,7E-3
response to lipopolysaccharide	GOTERM_BP_DIRECT	24	1,9	2,0	2,8E-3
IG	SMART	58	4,5	1,5	2,8E-3
leukocyte cell-cell adhesion	GOTERM_BP_DIRECT	7	0,5	4,7	2,9E-3
Transcriptional misregulation in cancer	KEGG_PATHWAY	24	1,9	1,9	3,0E-3

cell adhesion	GOTERM_BP_DIRECT	47	3,7	1,6	3,0E-3
EF-hand-like domain	INTERPRO	29	2,3	1,8	3,0E-3
Biotin	UP_KEYWORDS	4	0,3	12,0	3,0E-3
negative regulation of viral genome replication	GOTERM_BP_DIRECT	8	0,6	4,0	3,0E-3
Ubl conjugation pathway	UP_KEYWORDS	53	4,1	1,5	3,1E-3
JmjN	SMART	5	0,4	7,3	3,1E-3
MCM	SMART	5	0,4	7,3	3,1E-3
PI3Ka	SMART	5	0,4	7,3	3,1E-3
sequence variant	UP_SEQ_FEATURE	42	3,3	1,6	3,1E-3
Phosphatidylinositol 3-/4-kinase, catalytic domain	INTERPRO	6	0,5	5,7	3,1E-3
Helicase-associated domain	INTERPRO	6	0,5	5,7	3,1E-3
Myosin-like IQ motif-containing domain	INTERPRO	6	0,5	5,7	3,1E-3
ATP-grasp fold, subdomain 2	INTERPRO	6	0,5	5,7	3,1E-3
COP9 signalosome	GOTERM_CC_DIRECT	8	0,6	4,0	3,1E-3
phosphatidylinositol 3-kinase complex	GOTERM_CC_DIRECT	5	0,4	7,5	3,1E-3
Lysosome	UP_KEYWORDS	26	2,0	1,9	3,2E-3
zinc finger region:PHD-type; atypical	UP_SEQ_FEATURE	5	0,4	7,4	3,2E-3

positive regulation of tumor necrosis factor production	GOTERM_BP_DIRECT	11	0,9	3,0	3,3E-3
Pertussis	KEGG_PATHWAY	14	1,1	2,5	3,3E-3
Toll/interleukin-1 receptor homology (TIR) domain	INTERPRO	7	0,5	4,6	3,4E-3
cellular response to tumor necrosis factor	GOTERM_BP_DIRECT	16	1,2	2,3	3,4E-3
phosphatidylinositol binding	GOTERM_MF_DIRECT	14	1,1	2,5	3,4E-3
actin filament network formation	GOTERM_BP_DIRECT	5	0,4	7,3	3,5E-3
telomere maintenance via recombination	GOTERM_BP_DIRECT	5	0,4	7,3	3,5E-3
1-phosphatidylinositol-3-kinase activity	GOTERM_MF_DIRECT	5	0,4	7,3	3,5E-3
2'-5'-oligoadenylate synthetase activity	GOTERM_MF_DIRECT	5	0,4	7,3	3,5E-3
cell-cell adhesion	GOTERM_BP_DIRECT	23	1,8	2,0	3,5E-3
positive regulation of apoptotic process	GOTERM_BP_DIRECT	35	2,7	1,7	3,5E-3
EF-hand, Ca insensitive	INTERPRO	4	0,3	11,3	3,5E-3

Elongation factor G, III-V domain	INTERPRO	4	0,3	11,3	3,5E-3
Translation elongation factor EFG, V domain	INTERPRO	4	0,3	11,3	3,5E-3
Rudiment single hybrid motif	INTERPRO	4	0,3	11,3	3,5E-3
transmembrane receptor protein tyrosine kinase signaling pathway	GOTERM_BP_DIRECT	15	1,2	2,4	3,6E-3
cellular response to cytokine stimulus	GOTERM_BP_DIRECT	8	0,6	3,9	3,7E-3
region of interest:Alpha-3	UP_SEQ_FEATURE	6	0,5	5,4	3,7E-3
dendritic spine	GOTERM_CC_DIRECT	19	1,5	2,1	3,7E-3
Translocation	UP_KEYWORDS	12	0,9	2,8	3,8E-3
DNA replication	KEGG_PATHWAY	9	0,7	3,4	3,8E-3
cohesin complex	GOTERM_CC_DIRECT	4	0,3	11,0	3,9E-3
SM00878	SMART	4	0,3	10,5	3,9E-3
SM00838	SMART	4	0,3	10,5	3,9E-3
short sequence motif:DEAH box	UP_SEQ_FEATURE	8	0,6	3,8	3,9E-3
repeat:TPR 12	UP_SEQ_FEATURE	4	0,3	10,9	4,0E-3
glycosylation site:O-linked (Xyl...) (chondroitin sulfate)	UP_SEQ_FEATURE	4	0,3	10,9	4,0E-3

2'-5'-oligoadenylate synthetase 1, domain 2/C-terminal	INTERPRO	5	0,4	7,1	4,0E-3
Single hybrid motif	INTERPRO	5	0,4	7,1	4,0E-3
2'-5'-oligoadenylate synthase	INTERPRO	5	0,4	7,1	4,0E-3
positive regulation of B cell differentiation	GOTERM_BP_DIRECT	6	0,5	5,3	4,0E-3
Nuclease	UP_KEYWORDS	16	1,2	2,3	4,0E-3
HECT	INTERPRO	7	0,5	4,4	4,1E-3
DAPIN domain	INTERPRO	7	0,5	4,4	4,1E-3
Inflammatory bowel disease (IBD)	KEGG_PATHWAY	12	0,9	2,7	4,1E-3
negative regulation of natural killer cell mediated cytotoxicity	GOTERM_BP_DIRECT	4	0,3	10,7	4,2E-3
thiamine pyrophosphate binding	GOTERM_MF_DIRECT	4	0,3	10,7	4,2E-3
positive regulation of GTPase activity	GOTERM_BP_DIRECT	19	1,5	2,1	4,3E-3
cell adhesion molecule binding	GOTERM_MF_DIRECT	12	0,9	2,7	4,3E-3
mitotic nuclear division	GOTERM_BP_DIRECT	30	2,3	1,7	4,3E-3

positive regulation of interleukin-6 production	GOTERM_BP_DIRECT	10	0,8	3,1	4,5E-3
Alternative promoter usage	UP_KEYWORDS	10	0,8	3,1	4,5E-3
Multifunctional enzyme	UP_KEYWORDS	11	0,9	2,9	4,7E-3
dendritic cell differentiation	GOTERM_BP_DIRECT	5	0,4	6,7	5,0E-3
complement receptor mediated signaling pathway	GOTERM_BP_DIRECT	5	0,4	6,7	5,0E-3
regulation of innate immune response	GOTERM_BP_DIRECT	5	0,4	6,7	5,0E-3
Ribosomal protein S5 domain 2-type fold, subgroup	INTERPRO	7	0,5	4,3	5,0E-3
lipid metabolic process	GOTERM_BP_DIRECT	44	3,4	1,5	5,0E-3
repeat:WD 10	UP_SEQ_FEATURE	7	0,5	4,2	5,0E-3
domain:DAPIN	UP_SEQ_FEATURE	7	0,5	4,2	5,0E-3
repeat:WD 11	UP_SEQ_FEATURE	7	0,5	4,2	5,0E-3
domain:Helicase C-terminal	UP_SEQ_FEATURE	14	1,1	2,4	5,1E-3
DNA/RNA helicase, ATP-dependent, DEAH-box type, conserved site	INTERPRO	6	0,5	5,1	5,1E-3

chromosome, centromeric region	GOTERM_CC_DIRECT	18	1,4	2,1	5,2E-3
positive regulation of transcription elongation from RNA polymerase II promoter	GOTERM_BP_DIRECT	6	0,5	5,1	5,2E-3
positive regulation of T cell mediated cytotoxicity	GOTERM_BP_DIRECT	6	0,5	5,1	5,2E-3
hydrolase activity, acting on carbon-nitrogen (but not peptide) bonds	GOTERM_MF_DIRECT	6	0,5	5,1	5,2E-3
Cell cycle	KEGG_PATHWAY	19	1,5	2,0	5,3E-3
PI3Kc	SMART	6	0,5	4,9	5,4E-3
actomyosin structure organization	GOTERM_BP_DIRECT	7	0,5	4,2	5,5E-3
Biosynthesis of antibiotics	KEGG_PATHWAY	28	2,2	1,7	5,6E-3
Pyridoxal phosphate	UP_KEYWORDS	10	0,8	3,0	5,7E-3
Cell division	UP_KEYWORDS	34	2,7	1,6	5,7E-3
response to virus	GOTERM_BP_DIRECT	13	1,0	2,5	5,7E-3
Ligase	UP_KEYWORDS	31	2,4	1,7	5,8E-3
phagocytic cup	GOTERM_CC_DIRECT	6	0,5	4,9	5,9E-3

Growth-arrest-specific protein 2 domain	INTERPRO	4	0,3	9,7	5,9E-3
Anaphase-promoting complex, subunit 10/DOC domain	INTERPRO	4	0,3	9,7	5,9E-3
Tropomodulin	INTERPRO	4	0,3	9,7	5,9E-3
MHC class II, beta chain, N-terminal	INTERPRO	4	0,3	9,7	5,9E-3
Phosphatidylinositol 3-kinase Ras-binding (PI3K RBD) domain	INTERPRO	4	0,3	9,7	5,9E-3
Rho guanyl-nucleotide exchange factor activity	GOTERM_MF_DIRECT	12	0,9	2,6	5,9E-3
ADP binding	GOTERM_MF_DIRECT	8	0,6	3,6	6,1E-3
axon	GOTERM_CC_DIRECT	36	2,8	1,6	6,2E-3
nucleotidyltransferase activity	GOTERM_MF_DIRECT	13	1,0	2,5	6,3E-3
Cytoskeleton	COG_ONTOLOGY	9	0,7	3,1	6,3E-3
Amoebiasis	KEGG_PATHWAY	18	1,4	2,0	6,6E-3
domain:DOC	UP_SEQ_FEATURE	4	0,3	9,3	6,6E-3
chain:H-2 class I histocompatibility antigen, Q8 alpha chain	UP_SEQ_FEATURE	4	0,3	9,3	6,6E-3

chain:class Ib MHC antigen Qa-2	UP_SEQ_FEATURE	4	0,3	9,3	6,6E-3
chain:H-2 class I histocompatibility antigen, Q7 alpha chain	UP_SEQ_FEATURE	4	0,3	9,3	6,6E-3
chain:H-2 class I histocompatibility antigen, Q9 alpha chain	UP_SEQ_FEATURE	4	0,3	9,3	6,6E-3
DNA replication, recombination, and repair	COG_ONTOLOGY	13	1,0	2,4	6,7E-3
mast cell activation	GOTERM_BP_DIRECT	5	0,4	6,2	6,8E-3
PTPc	SMART	9	0,7	3,1	6,8E-3
channel activity	GOTERM_MF_DIRECT	5	0,4	6,2	6,8E-3
G-protein coupled purinergic nucleotide receptor activity	GOTERM_MF_DIRECT	5	0,4	6,2	6,8E-3
pointed-end actin filament capping	GOTERM_BP_DIRECT	4	0,3	9,2	7,0E-3
1-phosphatidylinositol-4-phosphate 3-kinase activity	GOTERM_MF_DIRECT	4	0,3	9,2	7,0E-3
CCR5 chemokine receptor binding	GOTERM_MF_DIRECT	4	0,3	9,2	7,0E-3
BTB/Kelch-associated	INTERPRO	10	0,8	2,9	7,1E-3

domain:Ig-like C2-type 3	UP_SEQ_FEATURE	13	1,0	2,4	7,1E-3
2-5-oligoadenylate synthetase, N-terminal	INTERPRO	5	0,4	6,1	7,4E-3
ATP-grasp fold	INTERPRO	5	0,4	6,1	7,4E-3
ATPase, dynein-related, AAA domain	INTERPRO	5	0,4	6,1	7,4E-3
Pre-ATP-grasp domain	INTERPRO	5	0,4	6,1	7,4E-3
PI3K_rbd	SMART	4	0,3	8,8	7,4E-3
DNA damage	UP_KEYWORDS	31	2,4	1,7	7,4E-3
negative regulation of inflammatory response	GOTERM_BP_DIRECT	13	1,0	2,4	7,6E-3
Centromere	UP_KEYWORDS	16	1,2	2,1	7,7E-3
Winged helix-turn-helix DNA-binding domain	INTERPRO	24	1,9	1,8	7,7E-3
Pyrimidine metabolism	KEGG_PATHWAY	16	1,2	2,1	7,7E-3
positive regulation of NF-kappaB transcription factor activity	GOTERM_BP_DIRECT	15	1,2	2,2	7,7E-3
regulation of cell proliferation	GOTERM_BP_DIRECT	25	2,0	1,8	7,9E-3

Ribosomal protein S5 domain 2-type fold	INTERPRO	8	0,6	3,4	8,0E-3
mRNA transport	UP_KEYWORDS	12	0,9	2,5	8,0E-3
determination of adult lifespan	GOTERM_BP_DIRECT	6	0,5	4,6	8,2E-3
striated muscle thin filament	GOTERM_CC_DIRECT	5	0,4	5,9	8,2E-3
RhoGAP	SMART	12	0,9	2,5	8,3E-3
Protein-tyrosine/Dual specificity phosphatase	INTERPRO	12	0,9	2,5	8,3E-3
Immunoglobulin subtype 2	INTERPRO	25	2,0	1,8	8,5E-3
cleavage furrow	GOTERM_CC_DIRECT	9	0,7	3,0	8,7E-3
structural constituent of cytoskeleton	GOTERM_MF_DIRECT	12	0,9	2,5	8,8E-3
Chromatin regulator	UP_KEYWORDS	26	2,0	1,7	8,9E-3
positive regulation of phagocytosis	GOTERM_BP_DIRECT	9	0,7	3,0	9,0E-3
Sp100	INTERPRO	4	0,3	8,5	9,0E-3
positive regulation of interleukin-2 production	GOTERM_BP_DIRECT	5	0,4	5,7	9,1E-3
Fatty acid metabolism	UP_KEYWORDS	15	1,2	2,2	9,1E-3
endosome	GOTERM_CC_DIRECT	48	3,7	1,5	9,2E-3

Kelch-like protein, gigaxonin	INTERPRO	8	0,6	3,3	9,2E-3
Ras-association	INTERPRO	8	0,6	3,3	9,2E-3
SCY	SMART	10	0,8	2,7	9,3E-3
SM00847	SMART	6	0,5	4,4	9,3E-3
cytokine-mediated signaling pathway	GOTERM_BP_DIRECT	18	1,4	2,0	9,5E-3
Lipid degradation	UP_KEYWORDS	13	1,0	2,3	9,5E-3
ATP-grasp fold, subdomain 1	INTERPRO	5	0,4	5,7	9,6E-3
Myosin, N-terminal, SH3-like	INTERPRO	5	0,4	5,7	9,6E-3
histone deacetylase binding	GOTERM_MF_DIRECT	15	1,2	2,1	9,8E-3
myosin II complex	GOTERM_CC_DIRECT	4	0,3	8,2	9,9E-3
RNA polymerase, beta subunit, protrusion	INTERPRO	3	0,2	17,0	9,9E-3
RNA polymerase Rpb2, domain 3	INTERPRO	3	0,2	17,0	9,9E-3
DNA-directed RNA polymerase, subunit 2, domain 6	INTERPRO	3	0,2	17,0	9,9E-3
RNA polymerase Rpb2, domain 7	INTERPRO	3	0,2	17,0	9,9E-3
RNA polymerase Rpb2, domain 2	INTERPRO	3	0,2	17,0	9,9E-3

Domain of unknown function DUF2075	INTERPRO	3	0,2	17,0	9,9E-3
DNA-directed RNA polymerase, subunit 2	INTERPRO	3	0,2	17,0	9,9E-3
Cleavage/polyadenylation specificity factor, A subunit, C-terminal	INTERPRO	3	0,2	17,0	9,9E-3
Glycosyl transferase, family 35	INTERPRO	3	0,2	17,0	9,9E-3
Glycogen/starch/alpha-glucan phosphorylase	INTERPRO	3	0,2	17,0	9,9E-3
RNA polymerase Rpb2, OB-fold	INTERPRO	3	0,2	17,0	9,9E-3
Toll-like receptor	INTERPRO	3	0,2	17,0	9,9E-3
RNA polymerase, beta subunit, conserved site	INTERPRO	3	0,2	17,0	9,9E-3
RasGAP protein, C-terminal	INTERPRO	3	0,2	17,0	9,9E-3
domain:Ig-like C2-type	UP_SEQ_FEATURE	14	1,1	2,2	9,9E-3
Protein-tyrosine phosphatase, active site	INTERPRO	11	0,9	2,6	1,0E-2

phosphatidylinositol-3,4-bisphosphate binding	GOTERM_MF_DIRECT	6	0,5	4,4	1,0E-2
compositionally biased region:Ala/Asp-rich (DA-box)	UP_SEQ_FEATURE	4	0,3	8,2	1,0E-2
neuronal cell body	GOTERM_CC_DIRECT	47	3,7	1,5	1,0E-2
FERM/acyl-CoA-binding protein, 3-helical bundle	INTERPRO	9	0,7	2,9	1,0E-2
chromatin binding	GOTERM_MF_DIRECT	43	3,4	1,5	1,0E-2
early endosome	GOTERM_CC_DIRECT	24	1,9	1,8	1,0E-2
response to organic cyclic compound	GOTERM_BP_DIRECT	12	0,9	2,4	1,1E-2
Toll-like receptor 2-Toll-like receptor 6 protein complex	GOTERM_CC_DIRECT	3	0,2	16,5	1,1E-2
myosin II filament	GOTERM_CC_DIRECT	3	0,2	16,5	1,1E-2
B41	SMART	10	0,8	2,7	1,1E-2
maintenance of DNA methylation	GOTERM_BP_DIRECT	4	0,3	8,0	1,1E-2
regulation of defense response to virus	GOTERM_BP_DIRECT	4	0,3	8,0	1,1E-2
actin filament severing	GOTERM_BP_DIRECT	4	0,3	8,0	1,1E-2

microtubule minus-end binding	GOTERM_MF_DIRECT	4	0,3	8,0	1,1E-2
repeat:Spectrin 23	UP_SEQ_FEATURE	3	0,2	16,3	1,1E-2
domain:CH 3	UP_SEQ_FEATURE	3	0,2	16,3	1,1E-2
domain:Actin-binding 2	UP_SEQ_FEATURE	3	0,2	16,3	1,1E-2
domain:CH 4	UP_SEQ_FEATURE	3	0,2	16,3	1,1E-2
site:Involved in the association of subunits	UP_SEQ_FEATURE	3	0,2	16,3	1,1E-2
repeat:TPR 16	UP_SEQ_FEATURE	3	0,2	16,3	1,1E-2
domain:Actin-binding 1	UP_SEQ_FEATURE	3	0,2	16,3	1,1E-2
site:May be involved in allosteric control	UP_SEQ_FEATURE	3	0,2	16,3	1,1E-2
Hematopoietic cell lineage	KEGG_PATHWAY	14	1,1	2,2	1,1E-2
Pyridine nucleotide-disulphide oxidoreductase, FAD/NAD(P)-binding domain	INTERPRO	10	0,8	2,7	1,1E-2
Protein-tyrosine phosphatase, catalytic	INTERPRO	10	0,8	2,7	1,1E-2
Cell division and chromosome partitioning	COG_ONTOLOGY	16	1,2	2,0	1,1E-2

4 iron, 4 sulfur cluster binding	GOTERM_MF_DIRECT	8	0,6	3,2	1,1E-2
receptor activity	GOTERM_MF_DIRECT	20	1,6	1,9	1,1E-2
response to bacterial lipoprotein	GOTERM_BP_DIRECT	3	0,2	16,0	1,1E-2
cellular response to molecule of fungal origin	GOTERM_BP_DIRECT	3	0,2	16,0	1,1E-2
inositol trisphosphate biosynthetic process	GOTERM_BP_DIRECT	3	0,2	16,0	1,1E-2
cellular response to triacyl bacterial lipopeptide	GOTERM_BP_DIRECT	3	0,2	16,0	1,1E-2
ubiquitin-dependent protein catabolic process via the N-end rule pathway	GOTERM_BP_DIRECT	3	0,2	16,0	1,1E-2
leukocyte activation involved in immune response	GOTERM_BP_DIRECT	3	0,2	16,0	1,1E-2
long-chain-enoyl-CoA hydratase activity	GOTERM_MF_DIRECT	3	0,2	16,0	1,1E-2
ribonucleoside binding	GOTERM_MF_DIRECT	3	0,2	16,0	1,1E-2
glycogen phosphorylase activity	GOTERM_MF_DIRECT	3	0,2	16,0	1,1E-2
SNF2-related	INTERPRO	7	0,5	3,6	1,1E-2
muscle contraction	GOTERM_BP_DIRECT	9	0,7	2,9	1,2E-2

compositionally biased region:Poly-Glu	UP_SEQ_FEATURE	37	2,9	1,5	1,2E-2
negative regulation of innate immune response	GOTERM_BP_DIRECT	5	0,4	5,3	1,2E-2
myofibril assembly	GOTERM_BP_DIRECT	5	0,4	5,3	1,2E-2
regulation of actin cytoskeleton reorganization	GOTERM_BP_DIRECT	5	0,4	5,3	1,2E-2
MHC class II protein complex binding	GOTERM_MF_DIRECT	5	0,4	5,3	1,2E-2
Glycolysis	UP_KEYWORDS	7	0,5	3,6	1,2E-2
Tumor suppressor	UP_KEYWORDS	15	1,2	2,1	1,2E-2
Proto-oncogene	UP_KEYWORDS	13	1,0	2,3	1,2E-2
Toxoplasmosis	KEGG_PATHWAY	16	1,2	2,0	1,2E-2
SM01337	SMART	4	0,3	7,5	1,2E-2
GAS2	SMART	4	0,3	7,5	1,2E-2
SM00921	SMART	4	0,3	7,5	1,2E-2
regulation of GTPase activity	GOTERM_BP_DIRECT	11	0,9	2,5	1,2E-2
condensed chromosome kinetochore	GOTERM_CC_DIRECT	12	0,9	2,4	1,2E-2
lipid homeostasis	GOTERM_BP_DIRECT	8	0,6	3,1	1,3E-2
integrin-mediated signaling pathway	GOTERM_BP_DIRECT	13	1,0	2,2	1,3E-2
Fibronectin, type III	INTERPRO	22	1,7	1,8	1,3E-2

Short hematopoietin receptor, family 1, conserved site	INTERPRO	4	0,3	7,6	1,3E-2
Interferon regulatory factor, conserved site	INTERPRO	4	0,3	7,6	1,3E-2
MyTH4 domain	INTERPRO	4	0,3	7,6	1,3E-2
Interferon regulatory factor DNA-binding domain	INTERPRO	4	0,3	7,6	1,3E-2
Fos transforming protein	INTERPRO	4	0,3	7,6	1,3E-2
Ribosomal protein S1, RNA-binding domain	INTERPRO	4	0,3	7,6	1,3E-2
RNA-binding domain, S1	INTERPRO	4	0,3	7,6	1,3E-2
Cell shape	UP_KEYWORDS	6	0,5	4,1	1,3E-2
Carbon metabolism	KEGG_PATHWAY	17	1,3	1,9	1,3E-2
Salmonella infection	KEGG_PATHWAY	13	1,0	2,2	1,3E-2
negative regulation of angiogenesis	GOTERM_BP_DIRECT	11	0,9	2,4	1,3E-2
Flavoprotein	UP_KEYWORDS	15	1,2	2,1	1,4E-2
wound healing	GOTERM_BP_DIRECT	13	1,0	2,2	1,4E-2
Zinc finger, FYVE/PHD-type	INTERPRO	16	1,2	2,0	1,4E-2

domain:Rho-GAP	UP_SEQ_FEATURE	10	0,8	2,6	1,4E-2
domain:PI3K/PI4K	UP_SEQ_FEATURE	5	0,4	5,1	1,4E-2
repeat:TPR 10	UP_SEQ_FEATURE	5	0,4	5,1	1,4E-2
SM01289	SMART	7	0,5	3,4	1,4E-2
HECTc	SMART	7	0,5	3,4	1,4E-2
FAD	UP_KEYWORDS	14	1,1	2,1	1,4E-2
DNA-binding region:Tryptophan pentad repeat	UP_SEQ_FEATURE	4	0,3	7,3	1,4E-2
domain:SH2 2	UP_SEQ_FEATURE	4	0,3	7,3	1,4E-2
domain:SH2 1	UP_SEQ_FEATURE	4	0,3	7,3	1,4E-2
phosphoric diester hydrolase activity	GOTERM_MF_DIRECT	9	0,7	2,8	1,5E-2
cellular amino acid metabolic process	GOTERM_BP_DIRECT	6	0,5	4,0	1,5E-2
meiotic nuclear division	GOTERM_BP_DIRECT	6	0,5	4,0	1,5E-2
macroautophagy	GOTERM_BP_DIRECT	6	0,5	4,0	1,5E-2
DNA replication initiation	GOTERM_BP_DIRECT	6	0,5	4,0	1,5E-2
MyD88-dependent toll-like receptor signaling pathway	GOTERM_BP_DIRECT	5	0,4	5,0	1,5E-2
very long-chain fatty acid metabolic process	GOTERM_BP_DIRECT	5	0,4	5,0	1,5E-2

auditory receptor cell differentiation	GOTERM_BP_DIRECT	5	0,4	5,0	1,5E-2
regulatory region DNA binding	GOTERM_MF_DIRECT	5	0,4	5,0	1,5E-2
Cytokine-cytokine receptor interaction	KEGG_PATHWAY	29	2,3	1,6	1,5E-2
activation of innate immune response	GOTERM_BP_DIRECT	4	0,3	7,1	1,5E-2
antigen processing and presentation of exogenous protein antigen via MHC class Ib, TAP-dependent	GOTERM_BP_DIRECT	4	0,3	7,1	1,5E-2
positive regulation of protein localization to plasma membrane	GOTERM_BP_DIRECT	4	0,3	7,1	1,5E-2
actin filament depolymerization	GOTERM_BP_DIRECT	4	0,3	7,1	1,5E-2
positive regulation of interleukin-6 biosynthetic process	GOTERM_BP_DIRECT	4	0,3	7,1	1,5E-2
adhesion of symbiont to host	GOTERM_BP_DIRECT	4	0,3	7,1	1,5E-2
Arp2/3 complex binding	GOTERM_MF_DIRECT	4	0,3	7,1	1,5E-2

Myristate	UP_KEYWORDS	18	1,4	1,9	1,6E-2
double-strand break repair	GOTERM_BP_DIRECT	10	0,8	2,5	1,6E-2
protein complex binding	GOTERM_MF_DIRECT	34	2,7	1,5	1,6E-2
brain development	GOTERM_BP_DIRECT	23	1,8	1,7	1,6E-2
blood vessel remodeling	GOTERM_BP_DIRECT	8	0,6	3,0	1,6E-2
domain:TIR	UP_SEQ_FEATURE	6	0,5	3,9	1,6E-2
cellular response to calcium ion	GOTERM_BP_DIRECT	9	0,7	2,7	1,6E-2
Homologous recombination	KEGG_PATHWAY	7	0,5	3,3	1,6E-2
Kinetochore	UP_KEYWORDS	12	0,9	2,3	1,6E-2
TNF signaling pathway	KEGG_PATHWAY	16	1,2	1,9	1,7E-2
toll-like receptor, 1/2/4/6/10 types [Parent=PIRSF800008]	PIR_SUPERFAMILY	3	0,2	12,9	1,7E-2
glucan phosphorylase	PIR_SUPERFAMILY	3	0,2	12,9	1,7E-2
single organismal cell-cell adhesion	GOTERM_BP_DIRECT	14	1,1	2,1	1,7E-2
leukocyte migration	GOTERM_BP_DIRECT	7	0,5	3,3	1,7E-2
positive regulation of osteoclast differentiation	GOTERM_BP_DIRECT	6	0,5	3,8	1,7E-2

G-protein beta WD-40 repeat	INTERPRO	12	0,9	2,2	1,7E-2
cell division	GOTERM_BP_DIRECT	35	2,7	1,5	1,8E-2
cellular response to drug	GOTERM_BP_DIRECT	11	0,9	2,4	1,8E-2
core promoter binding	GOTERM_MF_DIRECT	11	0,9	2,3	1,8E-2
Interleukin-6 receptor alpha chain, binding	INTERPRO	4	0,3	6,8	1,8E-2
Metal-dependent hydrolase, composite domain	INTERPRO	4	0,3	6,8	1,8E-2
chromosome	GOTERM_CC_DIRECT	32	2,5	1,5	1,8E-2
intrinsic apoptotic signaling pathway in response to DNA damage	GOTERM_BP_DIRECT	9	0,7	2,7	1,8E-2
endocytosis	GOTERM_BP_DIRECT	20	1,6	1,8	1,8E-2
fatty acid beta-oxidation	GOTERM_BP_DIRECT	8	0,6	2,9	1,8E-2
domain:SH3 1	UP_SEQ_FEATURE	7	0,5	3,3	1,8E-2
CD20-like	INTERPRO	6	0,5	3,8	1,9E-2
MHC class II, alpha chain, N-terminal	INTERPRO	3	0,2	12,8	1,9E-2
Hepatic lectin, N-terminal	INTERPRO	3	0,2	12,8	1,9E-2
Spectrin, beta subunit	INTERPRO	3	0,2	12,8	1,9E-2

Lysine-specific demethylase-like domain	INTERPRO	3	0,2	12,8	1,9E-2
Translation elongation factor EFG/EF2, domain IV	INTERPRO	3	0,2	12,8	1,9E-2
Protein of unknown function DUF1394	INTERPRO	3	0,2	12,8	1,9E-2
DNA/RNA helicase, DEAD/DEAH box type, N-terminal	INTERPRO	10	0,8	2,5	1,9E-2
Iron-sulfur	UP_KEYWORDS	9	0,7	2,6	1,9E-2
domain:R3H	UP_SEQ_FEATURE	4	0,3	6,5	2,0E-2
protein ubiquitination involved in ubiquitin-dependent protein catabolic process	GOTERM_BP_DIRECT	16	1,2	1,9	2,0E-2
pyridoxal phosphate binding	GOTERM_MF_DIRECT	9	0,7	2,6	2,0E-2
AIM2 inflammasome complex	GOTERM_CC_DIRECT	3	0,2	12,4	2,0E-2
positive regulation of nitric oxide biosynthetic process	GOTERM_BP_DIRECT	8	0,6	2,8	2,0E-2
mRNA transport	GOTERM_BP_DIRECT	12	0,9	2,2	2,1E-2

region of interest:Poly phosphoinositide binding	UP_SEQ_FEATURE	3	0,2	12,3	2,1E-2
repeat:Plectin 3	UP_SEQ_FEATURE	3	0,2	12,3	2,1E-2
repeat:TPR 15	UP_SEQ_FEATURE	3	0,2	12,3	2,1E-2
repeat:Spectrin 22	UP_SEQ_FEATURE	3	0,2	12,3	2,1E-2
repeat:Plectin 5	UP_SEQ_FEATURE	3	0,2	12,3	2,1E-2
domain:IQ 6	UP_SEQ_FEATURE	3	0,2	12,3	2,1E-2
region of interest:Beta-1	UP_SEQ_FEATURE	3	0,2	12,3	2,1E-2
region of interest:Beta-2	UP_SEQ_FEATURE	3	0,2	12,3	2,1E-2
region of interest:Regulatory	UP_SEQ_FEATURE	3	0,2	12,3	2,1E-2
repeat:Plectin 4	UP_SEQ_FEATURE	3	0,2	12,3	2,1E-2
repeat:Plectin 7	UP_SEQ_FEATURE	3	0,2	12,3	2,1E-2
domain:C2 5	UP_SEQ_FEATURE	3	0,2	12,3	2,1E-2
repeat:Plectin 6	UP_SEQ_FEATURE	3	0,2	12,3	2,1E-2
domain:SH3 2	UP_SEQ_FEATURE	7	0,5	3,2	2,1E-2
nuclear pore complex assembly	GOTERM_BP_DIRECT	4	0,3	6,4	2,1E-2
pyroptosis	GOTERM_BP_DIRECT	4	0,3	6,4	2,1E-2
regulation of Rho protein signal transduction	GOTERM_BP_DIRECT	11	0,9	2,3	2,1E-2
ubiquitin-protein transferase activity	GOTERM_MF_DIRECT	31	2,4	1,5	2,1E-2

cellular response to DNA damage stimulus	GOTERM_BP_DIRECT	38	3,0	1,5	2,1E-2
Zinc finger, RING/FYVE/PHD-type	INTERPRO	38	3,0	1,5	2,1E-2
domain:LIM zinc-binding	UP_SEQ_FEATURE	5	0,4	4,5	2,1E-2
phosphatidylinositol-3-phosphate biosynthetic process	GOTERM_BP_DIRECT	3	0,2	12,0	2,1E-2
regulation of podosome assembly	GOTERM_BP_DIRECT	3	0,2	12,0	2,1E-2
negative regulation of CD4-positive, alpha-beta T cell proliferation	GOTERM_BP_DIRECT	3	0,2	12,0	2,1E-2
positive regulation of CD4-positive, alpha-beta T cell differentiation	GOTERM_BP_DIRECT	3	0,2	12,0	2,1E-2
phagolysosome assembly	GOTERM_BP_DIRECT	3	0,2	12,0	2,1E-2
positive regulation of pinocytosis	GOTERM_BP_DIRECT	3	0,2	12,0	2,1E-2
T cell antigen processing and presentation	GOTERM_BP_DIRECT	3	0,2	12,0	2,1E-2

neutrophil activation involved in immune response	GOTERM_BP_DIRECT	3	0,2	12,0	2,1E-2
negative regulation of myeloid dendritic cell activation	GOTERM_BP_DIRECT	3	0,2	12,0	2,1E-2
phosphorylase activity	GOTERM_MF_DIRECT	3	0,2	12,0	2,1E-2
mannokinase activity	GOTERM_MF_DIRECT	3	0,2	12,0	2,1E-2
fructokinase activity	GOTERM_MF_DIRECT	3	0,2	12,0	2,1E-2
RNA-dependent ATPase activity	GOTERM_MF_DIRECT	3	0,2	12,0	2,1E-2
microtubule associated complex	GOTERM_CC_DIRECT	6	0,5	3,7	2,1E-2
nuclear chromosome, telomeric region	GOTERM_CC_DIRECT	15	1,2	1,9	2,2E-2
calcium-binding region:2	UP_SEQ_FEATURE	14	1,1	2,0	2,2E-2
domain:C2 1	UP_SEQ_FEATURE	9	0,7	2,6	2,2E-2
domain:C2 2	UP_SEQ_FEATURE	9	0,7	2,6	2,2E-2
protein kinase C binding	GOTERM_MF_DIRECT	9	0,7	2,6	2,2E-2
drug binding	GOTERM_MF_DIRECT	14	1,1	2,0	2,2E-2
chromosome segregation	GOTERM_BP_DIRECT	12	0,9	2,2	2,2E-2
Chagas disease (American trypanosomiasis)	KEGG_PATHWAY	15	1,2	1,9	2,2E-2

EF-hand domain	INTERPRO	22	1,7	1,7	2,2E-2
double-stranded DNA binding	GOTERM_MF_DIRECT	16	1,2	1,9	2,2E-2
Leucine rich repeat 4	INTERPRO	5	0,4	4,5	2,3E-2
cellular response to epidermal growth factor stimulus	GOTERM_BP_DIRECT	7	0,5	3,1	2,3E-2
cytoplasmic microtubule organization	GOTERM_BP_DIRECT	7	0,5	3,1	2,3E-2
glycolytic process	GOTERM_BP_DIRECT	7	0,5	3,1	2,3E-2
sarcomere	GOTERM_CC_DIRECT	7	0,5	3,1	2,3E-2
negative regulation of B cell proliferation	GOTERM_BP_DIRECT	5	0,4	4,5	2,3E-2
DNA duplex unwinding	GOTERM_BP_DIRECT	5	0,4	4,5	2,3E-2
phosphotyrosine binding	GOTERM_MF_DIRECT	5	0,4	4,5	2,3E-2
neuron projection	GOTERM_CC_DIRECT	37	2,9	1,5	2,3E-2
Fc Epsilon Receptor I Signaling in Mast Cells	BIOCARTA	10	0,8	2,3	2,3E-2
Endosome	UP_KEYWORDS	36	2,8	1,5	2,3E-2
DHR-1 domain	INTERPRO	4	0,3	6,2	2,3E-2
Single-stranded nucleic acid binding R3H	INTERPRO	4	0,3	6,2	2,3E-2

DHR-2 domain	INTERPRO	4	0,3	6,2	2,3E-2
Dedicator of cytokinesis	INTERPRO	4	0,3	6,2	2,3E-2
Phospholipase C, phosphoinositol-specific, EF-hand-like	INTERPRO	4	0,3	6,2	2,3E-2
Amidohydrolase 1	INTERPRO	4	0,3	6,2	2,3E-2
Dedicator of cytokinesis C-terminal	INTERPRO	4	0,3	6,2	2,3E-2
FERM domain	INTERPRO	8	0,6	2,8	2,3E-2
response to ionizing radiation	GOTERM_BP_DIRECT	9	0,7	2,5	2,4E-2
cytoskeletal protein binding	GOTERM_MF_DIRECT	10	0,8	2,4	2,5E-2
microtubule binding	GOTERM_MF_DIRECT	21	1,6	1,7	2,5E-2
protein dephosphorylation	GOTERM_BP_DIRECT	16	1,2	1,9	2,5E-2
uropod	GOTERM_CC_DIRECT	4	0,3	6,0	2,5E-2
cytokinesis	GOTERM_BP_DIRECT	7	0,5	3,0	2,6E-2
repeat:Kelch 6	UP_SEQ_FEATURE	8	0,6	2,7	2,6E-2
repeat:WD 13	UP_SEQ_FEATURE	5	0,4	4,3	2,6E-2
domain:DHR-2	UP_SEQ_FEATURE	4	0,3	5,9	2,6E-2
short sequence motif:ITIM motif	UP_SEQ_FEATURE	4	0,3	5,9	2,6E-2
domain:DHR-1	UP_SEQ_FEATURE	4	0,3	5,9	2,6E-2
microtubule	GOTERM_CC_DIRECT	30	2,3	1,5	2,6E-2
MyTH4	SMART	4	0,3	5,8	2,6E-2
SM00316	SMART	4	0,3	5,8	2,6E-2

IRF	SMART	4	0,3	5,8	2,6E-2
nucleotide phosphate-binding region:FAD	UP_SEQ_FEATURE	9	0,7	2,5	2,6E-2
covalent chromatin modification	GOTERM_BP_DIRECT	26	2,0	1,6	2,7E-2
Complement and coagulation cascades	KEGG_PATHWAY	12	0,9	2,1	2,7E-2
TIR	SMART	6	0,5	3,4	2,7E-2
spindle pole	GOTERM_CC_DIRECT	13	1,0	2,0	2,7E-2
Lipid metabolism	UP_KEYWORDS	34	2,7	1,5	2,7E-2
nuclease activity	GOTERM_MF_DIRECT	14	1,1	2,0	2,7E-2
regulation of cytokine secretion	GOTERM_BP_DIRECT	4	0,3	5,8	2,7E-2
positive regulation of phagocytosis, engulfment	GOTERM_BP_DIRECT	4	0,3	5,8	2,7E-2
toll-like receptor 4 signaling pathway	GOTERM_BP_DIRECT	4	0,3	5,8	2,7E-2
histone monoubiquitination	GOTERM_BP_DIRECT	4	0,3	5,8	2,7E-2
nuclear migration	GOTERM_BP_DIRECT	4	0,3	5,8	2,7E-2
regulation of B cell differentiation	GOTERM_BP_DIRECT	4	0,3	5,8	2,7E-2
extracellular vesicle	GOTERM_CC_DIRECT	8	0,6	2,7	2,7E-2
profilin binding	GOTERM_MF_DIRECT	4	0,3	5,8	2,7E-2
complement receptor activity	GOTERM_MF_DIRECT	4	0,3	5,8	2,7E-2

phospholipid catabolic process	GOTERM_BP_DIRECT	5	0,4	4,2	2,7E-2
promoter-specific chromatin binding	GOTERM_MF_DIRECT	5	0,4	4,2	2,7E-2
peroxisome	GOTERM_CC_DIRECT	15	1,2	1,9	2,8E-2
Disease mutation	UP_KEYWORDS	11	0,9	2,2	2,8E-2
Kelch repeat type 1	INTERPRO	9	0,7	2,5	2,8E-2
protein kinase B signaling	GOTERM_BP_DIRECT	7	0,5	3,0	2,9E-2
Protein phosphatase	UP_KEYWORDS	14	1,1	1,9	2,9E-2
Immunoglobulin E-set	INTERPRO	13	1,0	2,0	3,0E-2
zinc finger region:Phorbol-ester/DAG-type	UP_SEQ_FEATURE	7	0,5	2,9	3,0E-2
calcium ion transport	GOTERM_BP_DIRECT	16	1,2	1,8	3,0E-2
SAND domain-like	INTERPRO	4	0,3	5,7	3,0E-2
Interferon alpha/beta receptor, beta chain	INTERPRO	4	0,3	5,7	3,0E-2
Spc97/Spc98	INTERPRO	3	0,2	10,2	3,1E-2
MHC class I, alpha chain, C-terminal	INTERPRO	3	0,2	10,2	3,1E-2
Zinc finger, C5HC2-type	INTERPRO	3	0,2	10,2	3,1E-2
Hexokinase, N-terminal	INTERPRO	3	0,2	10,2	3,1E-2
Hexokinase, conserved site	INTERPRO	3	0,2	10,2	3,1E-2

Hexokinase	INTERPRO	3	0,2	10,2	3,1E-2
Hexokinase, C-terminal	INTERPRO	3	0,2	10,2	3,1E-2
ubiquitin-dependent protein catabolic process	GOTERM_BP_DIRECT	17	1,3	1,8	3,1E-2
SM00889	SMART	3	0,2	9,8	3,1E-2
SM00920	SMART	3	0,2	9,8	3,1E-2
SMAD/FHA domain	INTERPRO	8	0,6	2,6	3,1E-2
positive regulation of cytokine production	GOTERM_BP_DIRECT	6	0,5	3,3	3,2E-2
DEP domain	INTERPRO	5	0,4	4,1	3,2E-2
Anaphase-promoting complex subunit 4, WD40 domain	INTERPRO	5	0,4	4,1	3,2E-2
spectrin, beta subunit	PIR_SUPERFAMILY	3	0,2	9,7	3,2E-2
phospholipase C-activating G-protein coupled receptor signaling pathway	GOTERM_BP_DIRECT	9	0,7	2,4	3,2E-2
platelet activation	GOTERM_BP_DIRECT	7	0,5	2,9	3,2E-2
spectrin	GOTERM_CC_DIRECT	3	0,2	9,9	3,2E-2
cuticular plate	GOTERM_CC_DIRECT	3	0,2	9,9	3,2E-2
dendritic spine neck	GOTERM_CC_DIRECT	3	0,2	9,9	3,2E-2
positive regulation of chemokine production	GOTERM_BP_DIRECT	5	0,4	4,0	3,3E-2
repeat:Spectrin 20	UP_SEQ_FEATURE	3	0,2	9,8	3,3E-2

repeat:Gelsolin-like 6	UP_SEQ_FEATURE	3	0,2	9,8	3,3E-2
repeat:Spectrin 18	UP_SEQ_FEATURE	3	0,2	9,8	3,3E-2
domain:C2 4	UP_SEQ_FEATURE	3	0,2	9,8	3,3E-2
repeat:Spectrin 19	UP_SEQ_FEATURE	3	0,2	9,8	3,3E-2
repeat:Spectrin 21	UP_SEQ_FEATURE	3	0,2	9,8	3,3E-2
domain:Carboxyltransferase	UP_SEQ_FEATURE	3	0,2	9,8	3,3E-2
SM00875	SMART	10	0,8	2,2	3,3E-2
domain:ATP-grasp	UP_SEQ_FEATURE	4	0,3	5,4	3,3E-2
domain:ARID	UP_SEQ_FEATURE	4	0,3	5,4	3,3E-2
memory	GOTERM_BP_DIRECT	11	0,9	2,1	3,3E-2
Endonuclease	UP_KEYWORDS	10	0,8	2,2	3,3E-2
kelch-like protein, gigaxonin type	PIR_SUPERFAMILY	8	0,6	2,5	3,4E-2
membrane to membrane docking	GOTERM_BP_DIRECT	3	0,2	9,6	3,4E-2
negative regulation of Arp2/3 complex-mediated actin nucleation	GOTERM_BP_DIRECT	3	0,2	9,6	3,4E-2
natural killer cell degranulation	GOTERM_BP_DIRECT	3	0,2	9,6	3,4E-2
negative regulation of mast cell activation	GOTERM_BP_DIRECT	3	0,2	9,6	3,4E-2

positive regulation of lymphocyte differentiation	GOTERM_BP_DIRECT	3	0,2	9,6	3,4E-2
hypermethylation of CpG island	GOTERM_BP_DIRECT	3	0,2	9,6	3,4E-2
positive regulation of receptor binding	GOTERM_BP_DIRECT	3	0,2	9,6	3,4E-2
B cell proliferation involved in immune response	GOTERM_BP_DIRECT	3	0,2	9,6	3,4E-2
positive regulation of cysteine-type endopeptidase activity	GOTERM_BP_DIRECT	3	0,2	9,6	3,4E-2
hexokinase activity	GOTERM_MF_DIRECT	3	0,2	9,6	3,4E-2
leucine binding	GOTERM_MF_DIRECT	3	0,2	9,6	3,4E-2
N-acylphosphatidylethanolamine-specific phospholipase D activity	GOTERM_MF_DIRECT	3	0,2	9,6	3,4E-2
oxidoreductase activity, acting on the aldehyde or oxo group of donors, disulfide as acceptor	GOTERM_MF_DIRECT	3	0,2	9,6	3,4E-2
glucokinase activity	GOTERM_MF_DIRECT	3	0,2	9,6	3,4E-2

nuclear membrane	GOTERM_CC_DIRECT	22	1,7	1,6	3,4E-2
flavin adenine dinucleotide binding	GOTERM_MF_DIRECT	10	0,8	2,2	3,4E-2
repeat:TPR 7	UP_SEQ_FEATURE	8	0,6	2,6	3,5E-2
mRNA cleavage	GOTERM_BP_DIRECT	4	0,3	5,3	3,5E-2
positive thymic T cell selection	GOTERM_BP_DIRECT	4	0,3	5,3	3,5E-2
blood circulation	GOTERM_BP_DIRECT	4	0,3	5,3	3,5E-2
positive regulation of cation channel activity	GOTERM_BP_DIRECT	4	0,3	5,3	3,5E-2
glomerular visceral epithelial cell development	GOTERM_BP_DIRECT	4	0,3	5,3	3,5E-2
negative regulation of cytokine production	GOTERM_BP_DIRECT	4	0,3	5,3	3,5E-2
tropomyosin binding	GOTERM_MF_DIRECT	4	0,3	5,3	3,5E-2
calcium-release channel activity	GOTERM_MF_DIRECT	4	0,3	5,3	3,5E-2
protein binding, bridging	GOTERM_MF_DIRECT	9	0,7	2,4	3,5E-2
phosphatidylinositol-4,5-bisphosphate binding	GOTERM_MF_DIRECT	9	0,7	2,4	3,5E-2
Death-like domain	INTERPRO	11	0,9	2,1	3,6E-2
phagocytic vesicle	GOTERM_CC_DIRECT	7	0,5	2,8	3,6E-2

Fatty acid metabolism	KEGG_PATHWAY	9	0,7	2,3	3,6E-2
Carbohydrate digestion and absorption	KEGG_PATHWAY	8	0,6	2,5	3,6E-2
Thiol protease	UP_KEYWORDS	14	1,1	1,9	3,6E-2
repeat:WD 12	UP_SEQ_FEATURE	5	0,4	3,9	3,6E-2
cellular response to fibroblast growth factor stimulus	GOTERM_BP_DIRECT	6	0,5	3,2	3,6E-2
positive regulation of calcium ion transport	GOTERM_BP_DIRECT	6	0,5	3,2	3,6E-2
Thiolase-like	INTERPRO	4	0,3	5,2	3,7E-2
Blc2 family	INTERPRO	4	0,3	5,2	3,7E-2
Proteoglycan	UP_KEYWORDS	7	0,5	2,8	3,7E-2
Platelet activation	KEGG_PATHWAY	17	1,3	1,7	3,7E-2
domain:EF-hand 1	UP_SEQ_FEATURE	18	1,4	1,7	3,8E-2
regulation of protein phosphorylation	GOTERM_BP_DIRECT	9	0,7	2,3	3,8E-2
protein localization to cilium	GOTERM_BP_DIRECT	5	0,4	3,8	3,8E-2
positive regulation of actin cytoskeleton reorganization	GOTERM_BP_DIRECT	5	0,4	3,8	3,8E-2

positive regulation of angiogenesis	GOTERM_BP_DIRECT	14	1,1	1,9	3,8E-2
lipopolysaccharide binding	GOTERM_MF_DIRECT	5	0,4	3,8	3,8E-2
cytokine binding	GOTERM_MF_DIRECT	5	0,4	3,8	3,8E-2
phosphotransferase activity, alcohol group as acceptor	GOTERM_MF_DIRECT	5	0,4	3,8	3,8E-2
DNA-directed RNA polymerase	UP_KEYWORDS	6	0,5	3,2	3,8E-2
Microtubule	UP_KEYWORDS	23	1,8	1,6	3,9E-2
Protein kinase C-like, phorbol ester/diacylglycerol binding	INTERPRO	9	0,7	2,3	3,9E-2
lipid catabolic process	GOTERM_BP_DIRECT	13	1,0	1,9	3,9E-2
neuromuscular junction	GOTERM_CC_DIRECT	9	0,7	2,3	3,9E-2
Inflammasome	UP_KEYWORDS	3	0,2	9,0	4,0E-2
positive regulation of multicellular organism growth	GOTERM_BP_DIRECT	7	0,5	2,7	4,0E-2
peptidyl-tyrosine autophosphorylation	GOTERM_BP_DIRECT	7	0,5	2,7	4,0E-2
Rac GTPase binding	GOTERM_MF_DIRECT	7	0,5	2,7	4,0E-2

low-density lipoprotein particle	GOTERM_CC_DIRECT	4	0,3	5,1	4,0E-2
fascia adherens	GOTERM_CC_DIRECT	4	0,3	5,1	4,0E-2
DNA repair	GOTERM_BP_DIRECT	29	2,3	1,5	4,0E-2
Zinc finger, PHD-type	INTERPRO	11	0,9	2,1	4,1E-2
Dioxygenase	UP_KEYWORDS	10	0,8	2,2	4,1E-2
response to toxic substance	GOTERM_BP_DIRECT	11	0,9	2,1	4,1E-2
protein processing	GOTERM_BP_DIRECT	11	0,9	2,1	4,1E-2
hemopoiesis	GOTERM_BP_DIRECT	11	0,9	2,1	4,1E-2
membrane organization	GOTERM_BP_DIRECT	9	0,7	2,3	4,1E-2
spectrin binding	GOTERM_MF_DIRECT	6	0,5	3,1	4,1E-2
ATP-dependent RNA helicase activity	GOTERM_MF_DIRECT	9	0,7	2,3	4,1E-2
Host-virus interaction	UP_KEYWORDS	5	0,4	3,7	4,2E-2
JmjC domain	INTERPRO	6	0,5	3,1	4,2E-2
domain:HECT	UP_SEQ_FEATURE	5	0,4	3,7	4,2E-2
Kelch repeat	UP_KEYWORDS	9	0,7	2,3	4,3E-2
chaperone mediated protein folding requiring cofactor	GOTERM_BP_DIRECT	4	0,3	4,9	4,3E-2
response to molecule of bacterial origin	GOTERM_BP_DIRECT	4	0,3	4,9	4,3E-2

JAK-STAT cascade involved in growth hormone signaling pathway	GOTERM_BP_DIRECT	4	0,3	4,9	4,3E-2
carbohydrate phosphorylation	GOTERM_BP_DIRECT	4	0,3	4,9	4,3E-2
apical plasma membrane	GOTERM_CC_DIRECT	29	2,3	1,5	4,3E-2
myelin sheath	GOTERM_CC_DIRECT	19	1,5	1,6	4,4E-2
Caspase, interleukin-1 beta convertase	INTERPRO	3	0,2	8,5	4,4E-2
Dedicator of cytokinesis C/D, N-terminal	INTERPRO	3	0,2	8,5	4,4E-2
Dilute	INTERPRO	3	0,2	8,5	4,4E-2
Dehydrogenase, E1 component	INTERPRO	3	0,2	8,5	4,4E-2
Hydantoinase/dihydropyrimidinase	INTERPRO	3	0,2	8,5	4,4E-2
Glutamine amidotransferase	INTERPRO	3	0,2	8,5	4,4E-2
calcium-binding region:1	UP_SEQ_FEATURE	14	1,1	1,8	4,4E-2
regulation of synaptic plasticity	GOTERM_BP_DIRECT	7	0,5	2,7	4,4E-2
iron-sulfur cluster binding	GOTERM_MF_DIRECT	9	0,7	2,3	4,5E-2
extracellular matrix disassembly	GOTERM_BP_DIRECT	5	0,4	3,6	4,5E-2

centrosome duplication	GOTERM_BP_DIRECT	5	0,4	3,6	4,5E-2
cellular response to extracellular stimulus	GOTERM_BP_DIRECT	5	0,4	3,6	4,5E-2
positive regulation of innate immune response	GOTERM_BP_DIRECT	5	0,4	3,6	4,5E-2
mitochondrial outer membrane	GOTERM_CC_DIRECT	16	1,2	1,7	4,5E-2
Dbl homology (DH) domain	INTERPRO	9	0,7	2,3	4,5E-2
Nucleotidyl transferase domain	INTERPRO	4	0,3	4,9	4,5E-2
Bcl2-like	INTERPRO	4	0,3	4,9	4,5E-2
Jak-STAT signaling pathway	KEGG_PATHWAY	18	1,4	1,6	4,6E-2
apical part of cell	GOTERM_CC_DIRECT	13	1,0	1,9	4,6E-2
response to axon injury	GOTERM_BP_DIRECT	6	0,5	3,0	4,6E-2
Neutrophil and Its Surface Molecules	BIOCARTA	4	0,3	4,6	4,7E-2
equatorial microtubule organizing center	GOTERM_CC_DIRECT	3	0,2	8,2	4,7E-2
inflammasome complex	GOTERM_CC_DIRECT	3	0,2	8,2	4,7E-2
meiotic cohesin complex	GOTERM_CC_DIRECT	3	0,2	8,2	4,7E-2
nuclear meiotic cohesin complex	GOTERM_CC_DIRECT	3	0,2	8,2	4,7E-2

endocytic vesicle	GOTERM_CC_DIRECT	8	0,6	2,4	4,7E-2
binding site:Sialic acid	UP_SEQ_FEATURE	3	0,2	8,2	4,7E-2
domain:PLD phosphodiesterase 2	UP_SEQ_FEATURE	3	0,2	8,2	4,7E-2
domain:PLD phosphodiesterase 1	UP_SEQ_FEATURE	3	0,2	8,2	4,7E-2
Tyrosine-protein kinase, catalytic domain	INTERPRO	10	0,8	2,1	4,8E-2
Sulfation	UP_KEYWORDS	6	0,5	3,0	4,8E-2
PTPc_motif	SMART	10	0,8	2,1	4,8E-2
domain:JmjC	UP_SEQ_FEATURE	6	0,5	3,0	4,8E-2
domain:Collagen-like	UP_SEQ_FEATURE	6	0,5	3,0	4,8E-2
repeat:TPR 9	UP_SEQ_FEATURE	5	0,4	3,6	4,9E-2
Butirosin and neomycin biosynthesis	KEGG_PATHWAY	3	0,2	7,9	4,9E-2
positive regulation of B cell proliferation	GOTERM_BP_DIRECT	7	0,5	2,6	4,9E-2
negative regulation of tumor necrosis factor production	GOTERM_BP_DIRECT	7	0,5	2,6	4,9E-2
Rhodanese-like domain	INTERPRO	5	0,4	3,5	4,9E-2
repeat:LRR 14	UP_SEQ_FEATURE	8	0,6	2,4	4,9E-2

toll-like receptor 9 signaling pathway	GOTERM_BP_DIRECT	3	0,2	8,0	4,9E-2
interphase microtubule nucleation by interphase microtubule organizing center	GOTERM_BP_DIRECT	3	0,2	8,0	4,9E-2
neutrophil clearance	GOTERM_BP_DIRECT	3	0,2	8,0	4,9E-2
positive regulation of natural killer cell chemotaxis	GOTERM_BP_DIRECT	3	0,2	8,0	4,9E-2
positive regulation of cellular extravasation	GOTERM_BP_DIRECT	3	0,2	8,0	4,9E-2
pore complex assembly	GOTERM_BP_DIRECT	3	0,2	8,0	4,9E-2
T cell chemotaxis	GOTERM_BP_DIRECT	3	0,2	8,0	4,9E-2
negative regulation of cellular component movement	GOTERM_BP_DIRECT	3	0,2	8,0	4,9E-2
regulation of adaptive immune response	GOTERM_BP_DIRECT	3	0,2	8,0	4,9E-2
lipoteichoic acid binding	GOTERM_MF_DIRECT	3	0,2	8,0	4,9E-2
xenobiotic-transporting ATPase activity	GOTERM_MF_DIRECT	3	0,2	8,0	4,9E-2

34 Genes from your list are not in the output

Sup. Table 6

Term	Gene count	%	Fold enrichment	p-value
Macrophage	122	9,5	3,2	3.3E-31
Spleen	160	12,5	2,4	7.1E-26
Thymus	294	23	1,7	5.1E-23
Bone marrow	179	14	1,7	9.5E-12
Activated spleen	81	6,3	2,2	1.3E-10

Sup. Table 6: Top 5 DAVID UP-TISSUE showing overrepresented genes in corresponding tissues/ cell types. Input: Mac^{TRAP} kidney messages with an enrichment > 2 and $P < 0.05$.

Functional annotation chart UP_TISSUE

Tool: DAVID

Input: 1448 macrophage translational signature genes

Category: tissue expression

Note: sorted by P-value

Category	Term	Count	%	P-Value	Fold Enrichment
UP_TISSUE	Macrophage	122	9,5	3,3E-31	3,2
UP_TISSUE	Spleen	160	12,5	7,1E-26	2,4
UP_TISSUE	Thymus	294	23,0	5,1E-23	1,7
UP_TISSUE	Bone marrow	179	14,0	9,5E-12	1,7
UP_TISSUE	Activated spleen	81	6,3	1,3E-10	2,2
UP_TISSUE	Bone	44	3,4	9,5E-9	2,7
UP_TISSUE	Mast cell	43	3,4	2,1E-8	2,6
UP_TISSUE	Embryonic tail	53	4,1	2,2E-8	2,3
UP_TISSUE	Liver	299	23,3	3,3E-7	1,3
UP_TISSUE	Hematopoietic stem cell	21	1,6	3,7E-6	3,4
UP_TISSUE	Dendritic cell	28	2,2	5,8E-6	2,7
UP_TISSUE	Mammary tumor metastatized to lung. Tumor arose spontaneously	60	4,7	1,3E-4	1,7
UP_TISSUE	Adult spleen	9	0,7	3,8E-4	4,9
UP_TISSUE	Lung	121	9,4	6,1E-4	1,3
UP_TISSUE	Aorta	20	1,6	7,3E-4	2,4
UP_TISSUE	Pancreatic islet	15	1,2	2,4E-3	2,5
UP_TISSUE	Mammary gland	185	14,4	3,0E-3	1,2
UP_TISSUE	Embryonic intestinal tract	8	0,6	3,4E-3	4,0
UP_TISSUE	Bone marrow macrophage	10	0,8	4,4E-3	3,1

UP_TISSUE	Aorta and vein	22	1,7	4,8E-3	1,9
UP_TISSUE	Mammary tumor. C3	51	4,0	5,1E-3	1,5
UP_TISSUE	Osteoblast	13	1,0	6,4E-3	2,5
UP_TISSUE	Eyeball	29	2,3	6,5E-3	1,7
UP_TISSUE	Vein	14	1,1	6,8E-3	2,3
UP_TISSUE	B-cell	18	1,4	7,7E-3	2,0
UP_TISSUE	Mammary tumor. Metallothionien-TGF alpha model. 10 month old virgin mouse. Taken by biopsy.	35	2,7	8,2E-3	1,6
UP_TISSUE	Plasma	13	1,0	1,0E-2	2,3
UP_TISSUE	Peritoneum	4	0,3	1,9E-2	6,7
UP_TISSUE	Peritoneal exudate cells	4	0,3	1,9E-2	6,7
UP_TISSUE	Skin	57	4,4	1,9E-2	1,3
UP_TISSUE	Neural Stem Cell	12	0,9	2,0E-2	2,2
UP_TISSUE	Lymph node	5	0,4	2,9E-2	4,2
UP_TISSUE	Fetal brain	29	2,3	3,0E-2	1,5
UP_TISSUE	Pre-B cell	4	0,3	3,1E-2	5,6
UP_TISSUE	Salivary gland	45	3,5	3,1E-2	1,4
UP_TISSUE	Adult thymus	6	0,5	3,2E-2	3,3
UP_TISSUE	Trophoblast stem cells	3	0,2	3,8E-2	9,2
UP_TISSUE	Neuron	3	0,2	3,8E-2	9,2
UP_TISSUE	Spinal ganglion	38	3,0	4,3E-2	1,4
UP_TISSUE	Natural killer cell	4	0,3	4,5E-2	4,9
UP_TISSUE	Leukemia	3	0,2	5,2E-2	7,9
UP_TISSUE	Mammary tumor	195	15,2	6,5E-2	1,1
UP_TISSUE	Diencephalon	28	2,2	6,5E-2	1,4
UP_TISSUE	Brain cortex	46	3,6	6,7E-2	1,3

UP_TISSUE	Retina	48	3,7	8,0E-2	1,3
UP_TISSUE	Endothelial cell	3	0,2	8,3E-2	6,1
UP_TISSUE	Cerebellum	110	8,6	8,3E-2	1,1
UP_TISSUE	Thymocyte	4	0,3	9,2E-2	3,7
UP_TISSUE	Inner ear	22	1,7	9,2E-2	1,4
UP_TISSUE	Adipose tissue	10	0,8	9,5E-2	1,8
UP_TISSUE	Vagina	17	1,3	9,7E-2	1,5

Functional Annotation Clustering
Tool: DAVID
Input: 1448 macrophage translational signature genes
Classification stringency: Medium
106 clusters with group enrichment score (GES) ≥ 1

**Annotation Cluster 1**		**Enrichment Score: 35.9**	**Count**	**P_Value**
	UP_KEYWORDS	Immunity	115	7.4E-50
	GOTERM_BP_DIRECT	immune system process	109	1.7E-42
	UP_KEYWORDS	Innate immunity	72	2.0E-32
	GOTERM_BP_DIRECT	innate immune response	82	1.0E-21
**Annotation Cluster 2**		**Enrichment Score: 32.1**	**Count**	**P_Value**
	UP_KEYWORDS	Actin-binding	83	6.6E-41
	GOTERM_MF_DIRECT	actin binding	96	2.5E-37
	GOTERM_MF_DIRECT	actin filament binding	44	3.1E-20
**Annotation Cluster 3**		**Enrichment Score: 19.27**	**Count**	**P_Value**
	UP_KEYWORDS	ATP-binding	171	3.5E-24
	INTERPRO	P-loop containing nucleoside triphosphate hydrolase	135	1.0E-23
	UP_KEYWORDS	Nucleotide-binding	199	7.8E-23
	GOTERM_MF_DIRECT	ATP binding	191	3.0E-22
	GOTERM_MF_DIRECT	nucleotide binding	202	7.0E-14
	UP_SEQ_FEATURE	nucleotide phosphate-binding region:ATP	118	4.1E-13
**Annotation Cluster 4**		**Enrichment Score: 8.18**	**Count**	**P_Value**

	INTERPRO	Pleckstrin homology domain	45	2.6E-10
	INTERPRO	Pleckstrin homology-like domain	59	4.0E-10
	UP_SEQ_FEATURE	domain:PH	36	4.3E-8
	SMART	PH	44	4.2E-7
**Annotation Cluster 5**		**Enrichment Score: 7.71**	**Count**	**P_Value**
	INTERPRO	Myosin head, motor domain	22	2.5E-16
	GOTERM_CC_DIRECT	brush border	29	2.7E-15
	GOTERM_CC_DIRECT	myosin complex	24	5.3E-15
	SMART	MYSc	22	4.2E-14
	UP_KEYWORDS	Myosin	21	2.6E-13
	GOTERM_MF_DIRECT	motor activity	27	7.4E-13
	UP_SEQ_FEATURE	domain:Myosin head-like	17	1.1E-12
	UP_KEYWORDS	Motor protein	30	6.8E-11
	INTERPRO	IQ motif, EF-hand binding site	23	4.9E-9
	UP_KEYWORDS	Calmodulin-binding	28	1.1E-8
	SMART	IQ	19	1.5E-8
	UP_SEQ_FEATURE	domain:IQ 2	12	1.4E-7
	GOTERM_MF_DIRECT	actin-dependent ATPase activity	9	2.2E-7
	UP_SEQ_FEATURE	domain:IQ 1	12	2.2E-7
	UP_SEQ_FEATURE	domain:IQ 4	8	3.2E-7
	UP_SEQ_FEATURE	domain:IQ 3	9	4.1E-7
	UP_SEQ_FEATURE	region of interest:Actin-binding	11	6.2E-7

GOTERM_MF_DIRECT	microfilament motor activity	9	1.8E-6
GOTERM_BP_DIRECT	actin filament-based movement	9	3.3E-6
GOTERM_MF_DIRECT	calmodulin binding	29	8.4E-6
UP_SEQ_FEATURE	domain:IQ 5	6	1.6E-5
GOTERM_CC_DIRECT	actomyosin	7	1.7E-5
INTERPRO	Myosin, N-terminal, SH3-like	5	9.6E-3
GOTERM_CC_DIRECT	myosin II filament	3	1.1E-2
UP_SEQ_FEATURE	domain:IQ 6	3	2.1E-2
INTERPRO	Myosin S1 fragment, N-terminal	3	1.1E-1

Annotation Cluster 6		Enrichment Score: 6.13	Count	P_Value
	INTERPRO	Calponin homology domain	32	6.3E-19
	SMART	CH	31	5.8E-17
	INTERPRO	Actinin-type, actin-binding, conserved site	17	3.0E-15
	INTERPRO	Spectrin/alpha-actinin	17	5.9E-13
	UP_SEQ_FEATURE	domain:CH 1	15	7.3E-13
	UP_SEQ_FEATURE	domain:CH 2	15	7.3E-13
	SMART	SPEC	17	2.9E-11
	INTERPRO	Spectrin repeat	14	2.5E-10
	UP_SEQ_FEATURE	repeat:Spectrin 3	12	3.7E-10
	UP_SEQ_FEATURE	repeat:Spectrin 4	11	5.6E-10
	UP_SEQ_FEATURE	domain:Actin-binding	11	5.6E-10

UP_SEQ_FEATURE	repeat:Spectrin 1	12	2.0E-9
UP_SEQ_FEATURE	repeat:Spectrin 2	12	2.0E-9
UP_SEQ_FEATURE	repeat:Spectrin 5	7	3.7E-6
UP_SEQ_FEATURE	domain:CH	12	2.4E-5
UP_SEQ_FEATURE	repeat:Spectrin 6	6	4.1E-5
UP_SEQ_FEATURE	repeat:Spectrin 9	5	4.2E-4
UP_SEQ_FEATURE	repeat:Spectrin 10	5	4.2E-4
UP_SEQ_FEATURE	repeat:Spectrin 11	5	4.2E-4
UP_SEQ_FEATURE	repeat:Spectrin 12	5	4.2E-4
UP_SEQ_FEATURE	repeat:Spectrin 13	5	4.2E-4
UP_SEQ_FEATURE	repeat:Spectrin 7	5	4.2E-4
UP_SEQ_FEATURE	repeat:Spectrin 8	5	4.2E-4
UP_SEQ_FEATURE	repeat:Spectrin 14	5	4.2E-4
UP_SEQ_FEATURE	repeat:Spectrin 15	5	4.2E-4
UP_SEQ_FEATURE	repeat:Spectrin 16	5	4.2E-4
UP_SEQ_FEATURE	repeat:Spectrin 17	5	4.2E-4
GOTERM_BP_DIRECT	actin filament capping	7	5.6E-4
UP_KEYWORDS	Actin capping	6	1.9E-3
INTERPRO	EF-hand, Ca insensitive	4	3.5E-3
UP_SEQ_FEATURE	repeat:Spectrin 23	3	1.1E-2
UP_SEQ_FEATURE	repeat:Spectrin 22	3	2.1E-2
UP_SEQ_FEATURE	repeat:Spectrin 18	3	3.3E-2
UP_SEQ_FEATURE	repeat:Spectrin 19	3	3.3E-2
UP_SEQ_FEATURE	repeat:Spectrin 20	3	3.3E-2

	UP_SEQ_FEATURE	repeat:Spectrin 21	3	3.3E-2
**Annotation Cluster 7**		**Enrichment Score: 5.48**	**Count**	**P_Value**
	INTERPRO	WD40-repeat-containing domain	54	1.3E-12
	INTERPRO	WD40/YVTN repeat-like-containing domain	55	2.2E-11
	UP_KEYWORDS	WD repeat	42	9.7E-10
	INTERPRO	WD40 repeat	39	2.6E-7
	INTERPRO	WD40 repeat, conserved site	25	2.4E-5
	UP_SEQ_FEATURE	repeat:WD 4	32	2.9E-5
	UP_SEQ_FEATURE	repeat:WD 3	33	3.4E-5
	UP_SEQ_FEATURE	repeat:WD 5	30	3.6E-5
	UP_SEQ_FEATURE	repeat:WD 1	33	5.1E-5
	UP_SEQ_FEATURE	repeat:WD 2	33	5.1E-5
	SMART	WD40	39	8.1E-5
	UP_SEQ_FEATURE	repeat:WD 7	19	7.1E-4
	UP_SEQ_FEATURE	repeat:WD 6	22	1.3E-3
	INTERPRO	G-protein beta WD-40 repeat	12	1.7E-2
**Annotation Cluster 8**		**Enrichment Score: 5.15**	**Count**	**P_Value**
	INTERPRO	C2 calcium-dependent membrane targeting	31	7.4E-9
	SMART	C2	23	1.7E-4
	UP_SEQ_FEATURE	domain:C2	14	2.8E-4
**Annotation Cluster 9**		**Enrichment Score: 4.85**	**Count**	**P_Value**

	UP_KEYWORDS	SH3 domain	33	1.7E-7
	INTERPRO	Src homology-3 domain	32	2.5E-6
	SMART	SH3	31	1.9E-4
	UP_SEQ_FEATURE	domain:SH3	23	5.0E-4
**Annotation Cluster 10**		**Enrichment Score: 4.78**	**Count**	**P_Value**
	INTERPRO	AAA+ ATPase domain	25	3.4E-6
	GOTERM_MF_DIRECT	ATPase activity	31	6.8E-6
	SMART	AAA	25	2.1E-4
**Annotation Cluster 11**		**Enrichment Score: 4.32**	**Count**	**P_Value**
	GOTERM_MF_DIRECT	carbohydrate binding	38	8.9E-8
	UP_KEYWORDS	Lectin	29	3.4E-7
	INTERPRO	C-type lectin-like	24	4.7E-6
	INTERPRO	C-type lectin	22	1.1E-5
	INTERPRO	C-type lectin fold	24	1.2E-5
	SMART	CLECT	22	4.0E-4
	INTERPRO	C-type lectin, conserved site	11	6.6E-4
	UP_SEQ_FEATURE	domain:C-type lectin	16	1.1E-3
	UP_KEYWORDS	Signal-anchor	29	2.5E-1
**Annotation Cluster 12**		**Enrichment Score: 3.88**	**Count**	**P_Value**
	KEGG_PATHWAY	Phagosome	41	5.5E-11
	KEGG_PATHWAY	Leishmaniasis	23	5.6E-10
	INTERPRO	Immunoglobulin C1-set	25	3.0E-9

INTERPRO	MHC classes I/II-like antigen recognition protein	20	9.7E-9
INTERPRO	Immunoglobulin/major histocompatibility complex, conserved site	22	1.4E-8
GOTERM_BP_DIRECT	antigen processing and presentation of exogenous peptide antigen via MHC class II	10	2.1E-8
GOTERM_BP_DIRECT	antigen processing and presentation	17	1.0E-7
SMART	IGc1	25	2.0E-7
KEGG_PATHWAY	Graft-versus-host disease	17	7.3E-7
KEGG_PATHWAY	Tuberculosis	34	7.8E-7
GOTERM_CC_DIRECT	MHC class II protein complex	8	1.8E-6
GOTERM_MF_DIRECT	beta-2-microglobulin binding	8	4.4E-6
UP_SEQ_FEATURE	domain:Ig-like C1-type	12	6.2E-6
KEGG_PATHWAY	Antigen processing and presentation	20	7.8E-6
GOTERM_MF_DIRECT	T cell receptor binding	8	8.4E-6

KEGG_PATHWAY	Type I diabetes mellitus	17	9.4E-6
UP_KEYWORDS	MHC II	7	1.1E-5
KEGG_PATHWAY	Allograft rejection	16	1.1E-5
KEGG_PATHWAY	Herpes simplex infection	35	1.3E-5
GOTERM_MF_DIRECT	peptide antigen binding	13	1.4E-5
INTERPRO	MHC class II, alpha/beta chain, N-terminal	7	1.4E-5
KEGG_PATHWAY	Viral myocarditis	19	1.7E-5
GOTERM_CC_DIRECT	MHC class I protein complex	8	2.1E-5
INTERPRO	MHC class I, alpha chain, alpha1/alpha2	12	4.4E-5
GOTERM_MF_DIRECT	TAP binding	6	4.5E-5
GOTERM_BP_DIRECT	antigen processing and presentation of peptide antigen via MHC class I	11	4.9E-5
UP_SEQ_FEATURE	region of interest:Connecting peptide	11	6.8E-5
INTERPRO	MHC class I-like antigen recognition	13	1.1E-4
UP_SEQ_FEATURE	region of interest:Alpha-2	8	1.2E-4

UP_SEQ_FEATURE	region of interest:Alpha-1	8	1.2E-4
KEGG_PATHWAY	Autoimmune thyroid disease	16	2.1E-4
KEGG_PATHWAY	HTLV-I infection	38	4.1E-4
KEGG_PATHWAY	Cell adhesion molecules (CAMs)	26	4.5E-4
UP_KEYWORDS	MHC I	6	4.7E-4
GOTERM_CC_DIRECT	endoplasmic reticulum exit site	7	6.7E-4
KEGG_PATHWAY	Intestinal immune network for IgA production	11	9.0E-4
KEGG_PATHWAY	Rheumatoid arthritis	16	1.1E-3
GOTERM_BP_DIRECT	antigen processing and presentation of peptide or polysaccharide antigen via MHC class II	5	1.5E-3
KEGG_PATHWAY	Asthma	8	1.5E-3
GOTERM_CC_DIRECT	Golgi medial cisterna	6	2.7E-3
UP_SEQ_FEATURE	region of interest:Alpha-3	6	3.7E-3
KEGG_PATHWAY	Inflammatory bowel disease (IBD)	12	4.1E-3

GOTERM_BP_DIRECT	positive regulation of T cell mediated cytotoxicity	6	5.2E-3
INTERPRO	MHC class II, beta chain, N-terminal	4	5.9E-3
UP_SEQ_FEATURE	chain:H-2 class I histocompatibility antigen, Q9 alpha chain	4	6.6E-3
UP_SEQ_FEATURE	chain:H-2 class I histocompatibility antigen, Q7 alpha chain	4	6.6E-3
UP_SEQ_FEATURE	chain:H-2 class I histocompatibility antigen, Q8 alpha chain	4	6.6E-3
UP_SEQ_FEATURE	chain:class Ib MHC antigen Qa-2	4	6.6E-3
GOTERM_MF_DIRECT	MHC class II protein complex binding	5	1.2E-2
KEGG_PATHWAY	Toxoplasmosis	16	1.2E-2
SMART	SM00921	4	1.2E-2
INTERPRO	MHC class II, alpha chain, N-terminal	3	1.9E-2
UP_SEQ_FEATURE	region of interest:Beta-2	3	2.1E-2
UP_SEQ_FEATURE	region of interest:Beta-1	3	2.1E-2

	INTERPRO	MHC class I, alpha chain, C-terminal	3	3.1E-2
	SMART	SM00920	3	3.1E-2
	GOTERM_BP_DIRECT	chaperone mediated protein folding requiring cofactor	4	4.3E-2
	KEGG_PATHWAY	Systemic lupus erythematosus	17	8.8E-2
	GOTERM_CC_DIRECT	multivesicular body	5	9.6E-2
	GOTERM_BP_DIRECT	antigen processing and presentation of exogenous peptide antigen via MHC class I, TAP-dependent	5	1.1E-1
	GOTERM_CC_DIRECT	integral component of lumenal side of endoplasmic reticulum membrane	3	2.3E-1
	GOTERM_MF_DIRECT	peptide binding	7	5.3E-1
**Annotation Cluster 13**		**Enrichment Score: 3.84**	**Count**	**P_Value**
	UP_KEYWORDS	Allosteric enzyme	16	1.4E-8
	KEGG_PATHWAY	Starch and sucrose metabolism	9	2.1E-3
	KEGG_PATHWAY	Insulin signaling pathway	16	1.1E-1
**Annotation Cluster 14**		**Enrichment Score: 3.82**	**Count**	**P_Value**

	UP_KEYWORDS	DNA replication	19	4.3E-6
	GOTERM_BP_DIRECT	DNA replication	20	2.1E-4
	KEGG_PATHWAY	DNA replication	9	3.8E-3
**Annotation Cluster 15**		**Enrichment**	**Count**	**P_Value**
	INTERPRO	SH2 domain	22	2.3E-6
	UP_KEYWORDS	SH2 domain	20	4.8E-6
	GOTERM_BP_DIRECT	peptidyl-tyrosine phosphorylation	15	3.8E-5
	SMART	SH2	21	8.1E-5
	UP_SEQ_FEATURE	domain:SH2	17	1.5E-4
	GOTERM_BP_DIRECT	peptidyl-tyrosine autophosphorylation	7	4.0E-2
	GOTERM_MF_DIRECT	non-membrane spanning protein tyrosine kinase activity	7	5.9E-2
**Annotation Cluster 16**		**Enrichment Score: 3.64**	**Count**	**P_Value**
	UP_SEQ_FEATURE	chain:PHD finger protein 11-like	5	6.6E-5
	UP_SEQ_FEATURE	chain:PHD finger protein 11	5	6.6E-5
	UP_SEQ_FEATURE	zinc finger region:PHD-type; degenerate	5	1.9E-4
	UP_SEQ_FEATURE	zinc finger region:PHD-type; atypical	5	3.2E-3
**Annotation Cluster 17**		**Enrichment Score: 3.58**	**Count**	**P_Value**
	GOTERM_CC_DIRECT	cell-cell adherens junction	41	8.4E-6

	GOTERM_MF_DIRECT	cadherin binding involved in cell-cell adhesion	33	6.4E-4
	GOTERM_BP_DIRECT	cell-cell adhesion	23	3.5E-3
**Annotation Cluster 18**		**Enrichment Score: 3.57**	**Count**	**P_Value**
	UP_KEYWORDS	Helicase	31	2.0E-11
	GOTERM_MF_DIRECT	helicase activity	32	5.0E-11
	INTERPRO	Helicase, superfamily 1/2, ATP-binding domain	20	2.3E-5
	INTERPRO	Helicase, C-terminal	19	6.2E-5
	SMART	DEXDc	20	5.8E-4
	SMART	HELICc	19	1.2E-3
	GOTERM_MF_DIRECT	ATP-dependent helicase activity	8	1.3E-3
	INTERPRO	Domain of unknown function DUF1605	6	2.4E-3
	UP_SEQ_FEATURE	domain:Helicase ATP-binding	15	2.5E-3
	INTERPRO	Helicase-associated domain	6	3.1E-3
	UP_SEQ_FEATURE	short sequence motif:DEAH box	8	3.9E-3
	UP_SEQ_FEATURE	domain:Helicase C-terminal	14	5.1E-3

	INTERPRO	DNA/RNA helicase, ATP-dependent, DEAH-box type, conserved site	6	5.1E-3
	SMART	SM00847	6	9.3E-3
	INTERPRO	SNF2-related	7	1.1E-2
	INTERPRO	DNA/RNA helicase, DEAD/DEAH box type, N-terminal	10	1.9E-2
	GOTERM_MF_DIRECT	ATP-dependent RNA helicase activity	9	4.1E-2
**Annotation Cluster 19**		**Enrichment Score: 3.56**	**Count**	**P_Value**
	INTERPRO	Rho GTPase activation protein	20	7.8E-7
	UP_KEYWORDS	GTPase activation	24	3.7E-5
	GOTERM_MF_DIRECT	GTPase activator activity	30	3.4E-4
	INTERPRO	Rho GTPase-activating protein domain	13	4.1E-4
	SMART	RhoGAP	12	8.3E-3
	UP_SEQ_FEATURE	domain:Rho-GAP	10	1.4E-2
**Annotation Cluster 20**		**Enrichment Score: 3.17**	**Count**	**P_Value**
	GOTERM_BP_DIRECT	stimulatory C-type lectin receptor signaling pathway	7	1.5E-6
	UP_SEQ_FEATURE	domain:SH2 1	4	1.4E-2
	UP_SEQ_FEATURE	domain:SH2 2	4	1.4E-2

Annotation Cluster 21		Enrichment Score: 3.14	Count	P_Value
	KEGG_PATHWAY	Influenza A	32	3.6E-6
	KEGG_PATHWAY	Measles	22	1.2E-3
	KEGG_PATHWAY	Hepatitis C	16	8.8E-2
**Annotation Cluster 22**		**Enrichment Score: 3.1**	**Count**	**P_Value**
	GOTERM_BP_DIRECT	neutrophil chemotaxis	21	4.1E-9
	UP_KEYWORDS	Chemotaxis	22	3.7E-8
	GOTERM_BP_DIRECT	cellular response to interferon-gamma	16	1.5E-5
	GOTERM_BP_DIRECT	positive regulation of inflammatory response	14	1.1E-4
	GOTERM_BP_DIRECT	monocyte chemotaxis	11	1.3E-4
	GOTERM_BP_DIRECT	cell chemotaxis	15	3.0E-4
	INTERPRO	CC chemokine, conserved site	7	4.1E-4
	GOTERM_BP_DIRECT	chemokine-mediated signaling pathway	12	5.0E-4
	GOTERM_MF_DIRECT	chemokine activity	11	6.3E-4
	GOTERM_BP_DIRECT	lymphocyte chemotaxis	9	7.6E-4
	INTERPRO	Chemokine interleukin-8-like domain	10	2.3E-3
	GOTERM_BP_DIRECT	cellular response to tumor necrosis factor	16	3.4E-3

	GOTERM_BP_DIRECT	positive regulation of GTPase activity	19	4.3E-3
	SMART	SCY	10	9.3E-3
	KEGG_PATHWAY	Cytokine-cytokine receptor interaction	29	1.5E-2
	GOTERM_BP_DIRECT	positive regulation of natural killer cell chemotaxis	3	4.9E-2
	UP_KEYWORDS	Cytokine	18	5.5E-2
	GOTERM_BP_DIRECT	cellular response to interleukin-1	10	6.1E-2
	GOTERM_MF_DIRECT	CCR chemokine receptor binding	5	9.4E-2
	GOTERM_MF_DIRECT	cytokine activity	18	1.8E-1
**Annotation Cluster 23**		**Enrichment Score: 3.02**	**Count**	**P_Value**
	UP_KEYWORDS	Immunoglobulin domain	65	8.5E-11
	INTERPRO	Immunoglobulin-like fold	109	7.8E-8
	INTERPRO	Immunoglobulin-like domain	92	6.5E-7
	INTERPRO	Immunoglobulin subtype	58	3.8E-6
	UP_SEQ_FEATURE	domain:Ig-like C2-type 1	20	4.2E-4
	UP_SEQ_FEATURE	domain:Ig-like C2-type 2	20	4.6E-4
	SMART	IG	58	2.8E-3
	UP_SEQ_FEATURE	domain:Ig-like C2-type 3	13	7.1E-3

	INTERPRO	Immunoglobulin subtype 2	25	8.5E-3
	UP_SEQ_FEATURE	domain:Ig-like C2-type 4	8	5.3E-2
	UP_SEQ_FEATURE	domain:Ig-like C2-type 7	4	7.0E-2
	UP_SEQ_FEATURE	domain:Ig-like C2-type 5	7	7.1E-2
	SMART	IGc2	25	1.1E-1
	INTERPRO	Immunoglobulin I-set	13	1.6E-1
	UP_SEQ_FEATURE	domain:Ig-like C2-type 6	4	2.9E-1
	INTERPRO	Immunoglobulin V-set	32	6.4E-1
**Annotation Cluster 24**		**Enrichment Score: 2.86**	**Count**	**P_Value**
	INTERPRO	Gelsolin domain	7	8.1E-5
	UP_SEQ_FEATURE	repeat:Gelsolin-like 4	5	4.2E-4
	UP_SEQ_FEATURE	repeat:Gelsolin-like 5	5	4.2E-4
	GOTERM_BP_DIRECT	actin filament capping	7	5.6E-4
	INTERPRO	Villin/Gelsolin	5	6.8E-4
	UP_SEQ_FEATURE	repeat:Gelsolin-like 1	5	8.0E-4
	UP_SEQ_FEATURE	repeat:Gelsolin-like 2	5	8.0E-4
	UP_SEQ_FEATURE	repeat:Gelsolin-like 3	5	8.0E-4
	SMART	GEL	5	1.8E-3
	UP_KEYWORDS	Actin capping	6	1.9E-3
	GOTERM_BP_DIRECT	actin filament severing	4	1.1E-2
	UP_SEQ_FEATURE	region of interest:Polyphosphoinositide binding	3	2.1E-2

	UP_SEQ_FEATURE	repeat:Gelsolin-like 6	3	3.3E-2
**Annotation Cluster 25**		**Enrichment Score: 2.81**	**Count**	**P_Value**
	GOTERM_CC_DIRECT	MCM complex	6	8.3E-5
	INTERPRO	Mini-chromosome maintenance, conserved site	5	1.6E-4
	GOTERM_MF_DIRECT	DNA helicase activity	7	5.6E-4
	UP_SEQ_FEATURE	domain:MCM	5	8.0E-4
	INTERPRO	Mini-chromosome maintenance, DNA-dependent ATPase	5	1.2E-3
	INTERPRO	Nucleic acid-binding, OB-fold	14	2.2E-3
	GOTERM_BP_DIRECT	DNA unwinding involved in DNA replication	5	2.3E-3
	SMART	MCM	5	3.1E-3
	KEGG_PATHWAY	DNA replication	9	3.8E-3
	GOTERM_BP_DIRECT	DNA replication initiation	6	1.5E-2
	GOTERM_CC_DIRECT	nuclear chromosome, telomeric region	15	2.2E-2
**Annotation Cluster 26**		**Enrichment Score: 2.68**	**Count**	**P_Value**
	GOTERM_BP_DIRECT	intraciliary retrograde transport	6	3.1E-4

	GOTERM_CC_DIRECT	intraciliary transport particle A	5	7.7E-4
	GOTERM_BP_DIRECT	protein localization to cilium	5	3.8E-2
**Annotation Cluster 27**		**Enrichment Score: 2.67**	**Count**	**P_Value**
	GOTERM_MF_DIRECT	phosphatidylinositol phospholipase C activity	8	4.0E-5
	UP_SEQ_FEATURE	domain:PI-PLC Y-box	7	1.0E-4
	INTERPRO	Phospholipase C, phosphatidylinositol-specific, Y domain	7	1.3E-4
	INTERPRO	Phosphoinositide phospholipase C	7	1.3E-4
	UP_SEQ_FEATURE	domain:PI-PLC X-box	7	2.4E-4
	UP_SEQ_FEATURE	domain:C2	14	2.8E-4
	SMART	PLCYc	7	5.2E-4
	INTERPRO	Phospholipase C, phosphatidylinositol-specific , X domain	7	5.7E-4
	SMART	PLCXc	7	1.6E-3
	INTERPRO	PLC-like phosphodiesterase, TIM beta/alpha-barrel domain	7	2.7E-3
	UP_KEYWORDS	Lipid degradation	13	9.5E-3

	GOTERM_MF_DIRECT	phosphoric diester hydrolase activity	9	1.5E-2
	INTERPRO	Phospholipase C, phosphoinositol-specific, EF-hand-like	4	2.3E-2
	GOTERM_BP_DIRECT	phospholipid catabolic process	5	2.7E-2
	GOTERM_BP_DIRECT	lipid catabolic process	13	3.9E-2
	GOTERM_MF_DIRECT	phospholipase C activity	3	1.0E-1
	KEGG_PATHWAY	Ether lipid metabolism	6	2.4E-1
**Annotation Cluster 28**		**Enrichment Score: 2.66**	**Count**	**P_Value**
	UP_KEYWORDS	Cell cycle	58	1.8E-4
	GOTERM_BP_DIRECT	cell cycle	59	1.0E-3
	UP_KEYWORDS	Mitosis	28	1.3E-3
	GOTERM_BP_DIRECT	mitotic nuclear division	30	4.3E-3
	UP_KEYWORDS	Cell division	34	5.7E-3
	GOTERM_BP_DIRECT	cell division	35	1.8E-2
**Annotation Cluster 29**		**Enrichment Score: 2.65**	**Count**	**P_Value**
	KEGG_PATHWAY	B cell receptor signaling pathway	17	4.9E-5
	KEGG_PATHWAY	Fc epsilon RI signaling pathway	15	4.6E-4
	KEGG_PATHWAY	T cell receptor signaling pathway	9	5.0E-1

Annotation Cluster 30		Enrichment Score: 2.52	Count	P_Value
	GOTERM_BP_DIRECT	actin crosslink formation	7	3.9E-5
	GOTERM_CC_DIRECT	actin filament bundle	5	1.3E-3
	GOTERM_BP_DIRECT	actin filament network formation	5	3.5E-3
	UP_SEQ_FEATURE	domain:CH 4	3	1.1E-2
	UP_SEQ_FEATURE	domain:CH 3	3	1.1E-2
	UP_SEQ_FEATURE	domain:Actin-binding 2	3	1.1E-2
	UP_SEQ_FEATURE	domain:Actin-binding 1	3	1.1E-2
**Annotation Cluster 31**		**Enrichment Score: 2.5**	**Count**	**P_Value**
	UP_SEQ_FEATURE	repeat:WD 9	10	1.5E-4
	INTERPRO	Quinonprotein alcohol dehydrogenase-like superfamily	11	1.9E-4
	UP_SEQ_FEATURE	repeat:WD 8	11	4.5E-4
	UP_SEQ_FEATURE	repeat:WD 7	19	7.1E-4
	UP_SEQ_FEATURE	repeat:WD 10	7	5.0E-3
	UP_SEQ_FEATURE	repeat:WD 11	7	5.0E-3
	UP_SEQ_FEATURE	repeat:WD 13	5	2.6E-2
	UP_SEQ_FEATURE	repeat:WD 12	5	3.6E-2
	UP_SEQ_FEATURE	repeat:WD 14	3	1.6E-1
**Annotation Cluster 32**		**Enrichment Score: 2.44**	**Count**	**P_Value**
	GOTERM_CC_DIRECT	actomyosin	7	1.7E-5

INTERPRO	Myosin tail	7	5.7E-4
INTERPRO	Myosin-like IQ motif-containing domain	6	3.1E-3
GOTERM_BP_DIRECT	actomyosin structure organization	7	5.5E-3
INTERPRO	Myosin, N-terminal, SH3-like	5	9.6E-3
GOTERM_CC_DIRECT	myosin II complex	4	9.9E-3
GOTERM_CC_DIRECT	myosin II filament	3	1.1E-2
UP_KEYWORDS	Cell shape	6	1.3E-2
UP_SEQ_FEATURE	domain:IQ	8	5.3E-2
**Annotation Cluster 33**	**Enrichment Score: 2.41**	**Count**	**P_Value**
INTERPRO	Carbamoyl-phosphate synthetase large subunit-like, ATP-binding domain	5	3.6E-4
INTERPRO	Biotin-binding site	4	7.7E-4
INTERPRO	Carbamoyl-phosphate synthase, large subunit, N-terminal	4	1.8E-3
INTERPRO	Biotin carboxylase, C-terminal	4	1.8E-3
INTERPRO	Biotin carboxylation domain	4	1.8E-3
INTERPRO	Biotin/lipoyl attachment	5	1.9E-3
UP_SEQ_FEATURE	domain:Biotinyl-binding	4	2.1E-3

	UP_SEQ_FEATURE	domain:Biotin carboxylation	4	2.1E-3
	GOTERM_MF_DIRECT	biotin carboxylase activity	4	2.2E-3
	UP_KEYWORDS	Biotin	4	3.0E-3
	INTERPRO	ATP-grasp fold, subdomain 2	6	3.1E-3
	INTERPRO	Rudiment single hybrid motif	4	3.5E-3
	SMART	SM00878	4	3.9E-3
	INTERPRO	Single hybrid motif	5	4.0E-3
	INTERPRO	Pre-ATP-grasp domain	5	7.4E-3
	INTERPRO	ATP-grasp fold	5	7.4E-3
	INTERPRO	ATP-grasp fold, subdomain 1	5	9.6E-3
	UP_SEQ_FEATURE	domain:ATP-grasp	4	3.3E-2
	COG_ONTOLOGY	Lipid metabolism	8	5.4E-1
**Annotation Cluster 34**		**Enrichment Score: 2.4**	**Count**	**P_Value**
	UP_SEQ_FEATURE	domain:HIN-200	5	4.2E-4
	INTERPRO	HIN-200/IF120x	5	1.9E-3
	INTERPRO	DAPIN domain	7	4.1E-3
	UP_SEQ_FEATURE	domain:DAPIN	7	5.0E-3
	SMART	SM01289	7	1.4E-2
	GOTERM_MF_DIRECT	core promoter binding	11	1.8E-2

Annotation Cluster 35		Enrichment Score: 2.4	Count	P_Value
	INTERPRO	Zinc finger, N-recognin	5	3.6E-4
	UP_SEQ_FEATURE	zinc finger region:UBR-type	5	4.2E-4
	SMART	ZnF_UBR1	5	9.7E-4
	GOTERM_BP_DIRECT	ubiquitin-dependent protein catabolic process via the N-end rule pathway	3	1.1E-2
	GOTERM_CC_DIRECT	ubiquitin ligase complex	6	6.4E-1
**Annotation Cluster 36**		**Enrichment Score: 2.37**	**Count**	**P_Value**
	INTERPRO	Plectin repeat	6	1.3E-5
	SMART	PLEC	6	4.6E-5
	UP_SEQ_FEATURE	repeat:Plectin 1	4	2.1E-3
	UP_SEQ_FEATURE	repeat:Plectin 2	4	2.1E-3
	UP_SEQ_FEATURE	repeat:Plectin 6	3	2.1E-2
	UP_SEQ_FEATURE	repeat:Plectin 7	3	2.1E-2
	UP_SEQ_FEATURE	repeat:Plectin 3	3	2.1E-2
	UP_SEQ_FEATURE	repeat:Plectin 4	3	2.1E-2
	UP_SEQ_FEATURE	repeat:Plectin 5	3	2.1E-2
	GOTERM_CC_DIRECT	desmosome	4	2.1E-1
**Annotation Cluster 37**		**Enrichment Score: 2.33**	**Count**	**P_Value**
	UP_KEYWORDS	Disulfide bond	249	1.7E-9
	UP_SEQ_FEATURE	topological domain:Extracellular	197	1.2E-7
	UP_SEQ_FEATURE	disulfide bond	202	2.2E-5

	UP_KEYWORDS	Glycoprotein	265	5.6E-5
	UP_SEQ_FEATURE	topological domain:Cytoplasmic	214	1.2E-3
	UP_SEQ_FEATURE	signal peptide	218	1.9E-2
	UP_SEQ_FEATURE	glycosylation site:N-linked (GlcNAc...)	245	2.3E-2
	UP_KEYWORDS	Membrane	503	1.4E-1
	UP_KEYWORDS	Signal	262	2.9E-1
	UP_SEQ_FEATURE	transmembrane region	267	4.4E-1
	UP_KEYWORDS	Receptor	126	9.7E-1
	UP_KEYWORDS	Transmembrane	335	1.0E0
	UP_KEYWORDS	Transmembrane helix	333	1.0E0
	GOTERM_CC_DIRECT	integral component of membrane	338	1.0E0
**Annotation Cluster 38**		**Enrichment Score: 2.28**	**Count**	**P_Value**
	GOTERM_MF_DIRECT	double-stranded RNA binding	14	3.4E-4
	INTERPRO	2-5-oligoadenylate synthetase, conserved site	5	6.8E-4
	GOTERM_MF_DIRECT	2'-5'-oligoadenylate synthetase activity	5	3.5E-3
	INTERPRO	2'-5'-oligoadenylate synthase	5	4.0E-3

	INTERPRO	2'-5'-oligoadenylate synthetase 1, domain 2/C-terminal	5	4.0E-3
	INTERPRO	2-5-oligoadenylate synthetase, N-terminal	5	7.4E-3
	INTERPRO	Nucleotidyl transferase domain	4	4.5E-2
	GOTERM_BP_DIRECT	purine nucleotide biosynthetic process	4	1.3E-1
**Annotation Cluster 39**		**Enrichment Score: 2.27**	**Count**	**P_Value**
	INTERPRO	Phosphatidylinositol 3-kinase C2 (PI3K C2) domain	5	6.8E-4
	INTERPRO	Phosphatidylinositol 3/4-kinase, conserved site	6	1.3E-3
	GOTERM_BP_DIRECT	phosphatidylinositol-mediated signaling	9	1.4E-3
	SMART	PI3K_C2	5	1.8E-3
	INTERPRO	Phosphatidylinositol Kinase	5	1.9E-3
	INTERPRO	Phosphoinositide 3-kinase, accessory (PIK) domain	5	1.9E-3
	SMART	PI3Ka	5	3.1E-3

	INTERPRO	Phosphatidylinositol 3-/4-kinase, catalytic domain	6	3.1E-3
	GOTERM_CC_DIRECT	phosphatidylinositol 3-kinase complex	5	3.1E-3
	GOTERM_MF_DIRECT	1-phosphatidylinositol-3-kinase activity	5	3.5E-3
	SMART	PI3Kc	6	5.4E-3
	INTERPRO	Phosphatidylinositol 3-kinase Ras-binding (PI3K RBD) domain	4	5.9E-3
	GOTERM_MF_DIRECT	1-phosphatidylinositol-4-phosphate 3-kinase activity	4	7.0E-3
	SMART	PI3K_rbd	4	7.4E-3
	UP_SEQ_FEATURE	domain:PI3K/PI4K	5	1.4E-2
	GOTERM_BP_DIRECT	macroautophagy	6	1.5E-2
	GOTERM_BP_DIRECT	phosphatidylinositol-3-phosphate biosynthetic process	3	2.1E-2
	GOTERM_BP_DIRECT	phosphatidylinositol phosphorylation	5	6.7E-2
	GOTERM_BP_DIRECT	cellular response to starvation	7	1.7E-1
**Annotation Cluster 40**		**Enrichment Score: 2.17**	**Count**	**P_Value**
	GOTERM_BP_DIRECT	sister chromatid cohesion	7	2.7E-4

GOTERM_CC_DIRECT	cohesin complex	4	3.9E-3
GOTERM_BP_DIRECT	mitotic sister chromatid cohesion	3	2.9E-1
**Annotation Cluster 41**	**Enrichment Score: 2.06**	**Count**	**P_Value**
GOTERM_BP_DIRECT	positive regulation of interleukin-1 beta secretion	8	2.7E-4
GOTERM_CC_DIRECT	NLRP3 inflammasome complex	4	2.0E-3
GOTERM_CC_DIRECT	AIM2 inflammasome complex	3	2.0E-2
GOTERM_BP_DIRECT	pyroptosis	4	2.1E-2
UP_KEYWORDS	Inflammasome	3	4.0E-2
GOTERM_CC_DIRECT	inflammasome complex	3	4.7E-2
**Annotation Cluster 42**	**Enrichment Score: 2.06**	**Count**	**P_Value**
UP_KEYWORDS	DNA repair	30	9.2E-4
UP_KEYWORDS	DNA damage	31	7.4E-3
GOTERM_BP_DIRECT	cellular response to DNA damage stimulus	38	2.1E-2
GOTERM_BP_DIRECT	DNA repair	29	4.0E-2
**Annotation Cluster 43**	**Enrichment Score: 2**	**Count**	**P_Value**
GOTERM_BP_DIRECT	positive regulation of type III hypersensitivity	4	9.2E-4

	GOTERM_BP_DIRECT	antigen processing and presentation of exogenous peptide antigen via MHC class I	4	2.2E-3
	GOTERM_BP_DIRECT	positive regulation of phagocytosis	9	9.0E-3
	GOTERM_MF_DIRECT	IgG binding	3	6.6E-2
	GOTERM_BP_DIRECT	positive regulation of type IIa hypersensitivity	3	8.5E-2
**Annotation Cluster 44**		**Enrichment Score: 2**	**Count**	**P_Value**
	GOTERM_BP_DIRECT	sister chromatid cohesion	7	2.7E-4
	UP_SEQ_FEATURE	compositionally biased region:Ala/Asp-rich (DA-box)	4	1.0E-2
	INTERPRO	RecF/RecN/SMC	3	5.9E-2
	UP_SEQ_FEATURE	region of interest:Flexible hinge	3	6.4E-2
**Annotation Cluster 45**		**Enrichment Score: 1.96**	**Count**	**P_Value**
	GOTERM_CC_DIRECT	nuclear pore outer ring	7	8.3E-6
	UP_KEYWORDS	Nuclear pore complex	11	6.3E-5
	GOTERM_CC_DIRECT	nuclear pore	14	1.0E-4
	UP_KEYWORDS	Translocation	12	3.8E-3

UP_KEYWORDS	mRNA transport	12	8.0E-3
GOTERM_BP_DIRECT	mRNA transport	12	2.1E-2
GOTERM_BP_DIRECT	nuclear pore complex assembly	4	2.1E-2
GOTERM_MF_DIRECT	nucleocytoplasmic transporter activity	4	8.6E-2
GOTERM_MF_DIRECT	structural constituent of nuclear pore	4	8.6E-2
GOTERM_CC_DIRECT	nuclear periphery	4	1.2E-1
GOTERM_BP_DIRECT	mRNA export from nucleus	5	2.1E-1
KEGG_PATHWAY	RNA transport	14	5.2E-1
GOTERM_BP_DIRECT	protein import into nucleus	5	5.6E-1

**Annotation Cluster 46**	**Enrichment Score: 1.95**	**Count**	**P_Value**
INTERPRO	Ras GTPase-activating protein, conserved site	6	6.0E-4
INTERPRO	Ras GTPase-activating protein	6	1.8E-3
SMART	RasGAP	6	1.9E-3
UP_SEQ_FEATURE	domain:Ras-GAP	5	2.2E-3
INTERPRO	RasGAP protein, C-terminal	3	9.9E-3
GOTERM_BP_DIRECT	regulation of GTPase activity	11	1.2E-2

	GOTERM_CC_DIRECT	intrinsic component of the cytoplasmic side of the plasma membrane	3	1.6E-1
	INTERPRO	WW domain	6	1.8E-1
	GOTERM_BP_DIRECT	negative regulation of Ras protein signal transduction	4	2.0E-1
**Annotation Cluster 47**		**Enrichment Score: 1.94**	**Count**	**P_Value**
	GOTERM_CC_DIRECT	kinetochore	17	2.4E-3
	GOTERM_CC_DIRECT	chromosome, centromeric region	18	5.2E-3
	UP_KEYWORDS	Centromere	16	7.7E-3
	GOTERM_CC_DIRECT	condensed chromosome kinetochore	12	1.2E-2
	UP_KEYWORDS	Kinetochore	12	1.6E-2
	GOTERM_CC_DIRECT	chromosome	32	1.8E-2
	UP_KEYWORDS	Chromosome	31	7.6E-2
**Annotation Cluster 48**		**Enrichment Score: 1.92**	**Count**	**P_Value**
	INTERPRO	Tropomodulin	4	5.9E-3
	GOTERM_BP_DIRECT	pointed-end actin filament capping	4	7.0E-3
	GOTERM_CC_DIRECT	striated muscle thin filament	5	8.2E-3
	GOTERM_BP_DIRECT	muscle contraction	9	1.2E-2

	GOTERM_BP_DIRECT	myofibril assembly	5	1.2E-2
	GOTERM_CC_DIRECT	sarcomere	7	2.3E-2
	GOTERM_MF_DIRECT	tropomyosin binding	4	3.5E-2
**Annotation Cluster 49**		**Enrichment Score: 1.9**	**Count**	**P_Value**
	GOTERM_MF_DIRECT	cytokine receptor activity	11	3.7E-4
	INTERPRO	Short hematopoietin receptor, family 1, conserved site	4	1.3E-2
	INTERPRO	Interleukin-6 receptor alpha chain, binding	4	1.8E-2
	UP_SEQ_FEATURE	short sequence motif:WSXWS motif	6	5.4E-2
	UP_SEQ_FEATURE	short sequence motif:Box 1 motif	5	7.1E-2
**Annotation Cluster 50**		**Enrichment Score: 1.82**	**Count**	**P_Value**
	UP_KEYWORDS	Thiamine pyrophosphate	5	2.3E-3
	GOTERM_MF_DIRECT	oxidoreductase activity, acting on the aldehyde or oxo group of donors, disulfide as acceptor	3	3.4E-2
	INTERPRO	Dehydrogenase, E1 component	3	4.4E-2

Annotation Cluster 51		Enrichment Score: 1.79	Count	P_Value
	INTERPRO	Formyl peptide receptor family	5	1.9E-3
	GOTERM_BP_DIRECT	complement receptor mediated signaling pathway	5	5.0E-3
	GOTERM_BP_DIRECT	leukocyte migration	7	1.7E-2
	GOTERM_MF_DIRECT	complement receptor activity	4	2.7E-2
	GOTERM_BP_DIRECT	phospholipase C-activating G-protein coupled receptor signaling pathway	9	3.2E-2
	GOTERM_BP_DIRECT	positive regulation of cytosolic calcium ion concentration	14	1.3E-1
**Annotation Cluster 52**		**Enrichment Score: 1.76**	**Count**	**P_Value**
	UP_KEYWORDS	Thiamine pyrophosphate	5	2.3E-3
	GOTERM_MF_DIRECT	thiamine pyrophosphate binding	4	4.2E-3
	INTERPRO	Transketolase-like, pyrimidine-binding domain	3	7.6E-2
	SMART	SM00861	3	1.2E-1
**Annotation Cluster 53**		**Enrichment Score: 1.76**	**Count**	**P_Value**

	GOTERM_MF_DIRECT	hydrolase activity, acting on carbon-nitrogen (but not peptide) bonds	6	5.2E-3
	INTERPRO	Metal-dependent hydrolase, composite domain	4	1.8E-2
	INTERPRO	Amidohydrolase 1	4	2.3E-2
	INTERPRO	Hydantoinase/dihydropyrimidinase	3	4.4E-2
**Annotation Cluster 54**		**Enrichment Score: 1.74**	**Count**	**P_Value**
	INTERPRO	Anaphase-promoting complex, subunit 10/DOC domain	4	5.9E-3
	UP_SEQ_FEATURE	domain:DOC	4	6.6E-3
	SMART	SM01337	4	1.2E-2
	INTERPRO	Galactose-binding domain-like	8	2.4E-1
**Annotation Cluster 55**		**Enrichment Score: 1.69**	**Count**	**P_Value**
	UP_KEYWORDS	Guanine-nucleotide releasing factor	19	4.2E-4
	GOTERM_MF_DIRECT	guanyl-nucleotide exchange factor activity	21	1.7E-3
	GOTERM_MF_DIRECT	Rho guanyl-nucleotide exchange factor activity	12	5.9E-3

	GOTERM_BP_DIRECT	regulation of Rho protein signal transduction	11	2.1E-2
	INTERPRO	Dbl homology (DH) domain	9	4.5E-2
	SMART	RhoGEF	9	1.2E-1
	UP_SEQ_FEATURE	domain:DH	7	1.5E-1
	INTERPRO	Guanine-nucleotide dissociation stimulator, CDC24, conserved site	3	4.2E-1
**Annotation Cluster 56**		**Enrichment Score: 1.66**	**Count**	**P_Value**
	UP_KEYWORDS	4Fe-4S	8	2.3E-3
	GOTERM_MF_DIRECT	4 iron, 4 sulfur cluster binding	8	1.1E-2
	UP_KEYWORDS	Iron-sulfur	9	1.9E-2
	GOTERM_MF_DIRECT	iron-sulfur cluster binding	9	4.5E-2
	UP_SEQ_FEATURE	metal ion-binding site:Iron-sulfur (4Fe-4S)	3	2.3E-1
**Annotation Cluster 57**		**Enrichment Score: 1.61**	**Count**	**P_Value**
	INTERPRO	Pyridine nucleotide-disulphide oxidoreductase, FAD/NAD(P)-binding domain	10	1.1E-2

	UP_KEYWORDS	Flavoprotein	15	1.4E-2
	UP_KEYWORDS	FAD	14	1.4E-2
	UP_SEQ_FEATURE	nucleotide phosphate-binding region:FAD	9	2.6E-2
	GOTERM_MF_DIRECT	flavin adenine dinucleotide binding	10	3.4E-2
	GOTERM_MF_DIRECT	electron carrier activity	7	1.1E-1
**Annotation Cluster 58**		**Enrichment Score: 1.6**	**Count**	**P_Value**
	UP_KEYWORDS	Nucleotidyltransferase	14	2.7E-4
	GOTERM_MF_DIRECT	nucleotidyltransferase activity	13	6.3E-3
	KEGG_PATHWAY	Pyrimidine metabolism	16	7.7E-3
	INTERPRO	RNA polymerase Rpb2, domain 2	3	9.9E-3
	INTERPRO	RNA polymerase Rpb2, domain 7	3	9.9E-3
	INTERPRO	RNA polymerase Rpb2, domain 3	3	9.9E-3
	INTERPRO	RNA polymerase, beta subunit, protrusion	3	9.9E-3
	INTERPRO	RNA polymerase, beta subunit, conserved site	3	9.9E-3

	INTERPRO	RNA polymerase Rpb2, OB-fold	3	9.9E-3
	INTERPRO	DNA-directed RNA polymerase, subunit 2, domain 6	3	9.9E-3
	INTERPRO	DNA-directed RNA polymerase, subunit 2	3	9.9E-3
	GOTERM_MF_DIRECT	ribonucleoside binding	3	1.1E-2
	UP_KEYWORDS	DNA-directed RNA polymerase	6	3.8E-2
	GOTERM_MF_DIRECT	DNA-directed RNA polymerase activity	6	6.4E-2
	KEGG_PATHWAY	RNA polymerase	6	7.2E-2
	KEGG_PATHWAY	Purine metabolism	17	2.8E-1
	GOTERM_MF_DIRECT	RNA polymerase III activity	3	3.1E-1
	GOTERM_CC_DIRECT	DNA-directed RNA polymerase III complex	3	3.2E-1
	GOTERM_BP_DIRECT	transcription from RNA polymerase III promoter	3	3.3E-1
	UP_SEQ_FEATURE	zinc finger region:C4-type	7	4.3E-1
**Annotation Cluster 59**		**Enrichment**	**Count**	**P_Value**
	INTERPRO	HECT	7	4.1E-3
	SMART	HECTc	7	1.4E-2

	GOTERM_BP_DIRECT	protein ubiquitination involved in ubiquitin-dependent protein catabolic process	16	2.0E-2
	UP_SEQ_FEATURE	domain:HECT	5	4.2E-2
	UP_SEQ_FEATURE	active site:Glycyl thioester intermediate	8	2.3E-1
**Annotation Cluster 60**		**Enrichment Score: 1.59**	**Count**	**P_Value**
	GOTERM_MF_DIRECT	long-chain-enoyl-CoA hydratase activity	3	1.1E-2
	GOTERM_BP_DIRECT	fatty acid beta-oxidation	8	1.8E-2
	GOTERM_MF_DIRECT	3-hydroxyacyl-CoA dehydrogenase activity	3	8.5E-2
**Annotation Cluster 61**		**Enrichment Score: 1.58**	**Count**	**P_Value**
	GOTERM_BP_DIRECT	lipid metabolic process	44	5.0E-3
	UP_KEYWORDS	Fatty acid metabolism	15	9.1E-3
	GOTERM_BP_DIRECT	fatty acid beta-oxidation	8	1.8E-2
	UP_KEYWORDS	Lipid metabolism	34	2.7E-2
	KEGG_PATHWAY	Fatty acid metabolism	9	3.6E-2
	GOTERM_BP_DIRECT	fatty acid metabolic process	16	6.3E-2

	KEGG_PATHWAY	Fatty acid degradation	7	1.6E-1
**Annotation Cluster 62**		**Enrichment Score: 1.58**	**Count**	**P_Value**
	INTERPRO	Dedicator of cytokinesis	4	2.3E-2
	INTERPRO	Dedicator of cytokinesis C-terminal	4	2.3E-2
	INTERPRO	DHR-1 domain	4	2.3E-2
	INTERPRO	DHR-2 domain	4	2.3E-2
	UP_SEQ_FEATURE	domain:DHR-2	4	2.6E-2
	UP_SEQ_FEATURE	domain:DHR-1	4	2.6E-2
	INTERPRO	Dedicator of cytokinesis C/D, N-terminal	3	4.4E-2
**Annotation Cluster 63**		**Enrichment Score: 1.57**	**Count**	**P_Value**
	INTERPRO	Ribosomal protein S1, RNA-binding domain	4	1.3E-2
	INTERPRO	RNA-binding domain, S1	4	1.3E-2
	SMART	SM00316	4	2.6E-2
	UP_SEQ_FEATURE	domain:S1 motif	3	1.2E-1
**Annotation Cluster 64**		**Enrichment Score: 1.57**	**Count**	**P_Value**
	COG_ONTOLOGY	Cytoskeleton	9	6.3E-3
	INTERPRO	Dilute	3	4.4E-2
	SMART	SM01132	3	7.1E-2
**Annotation Cluster 65**		**Enrichment Score: 1.53**	**Count**	**P_Value**
	GOTERM_MF_DIRECT	microtubule binding	21	2.5E-2
	GOTERM_CC_DIRECT	microtubule	30	2.6E-2

	UP_KEYWORDS	Microtubule	23	3.9E-2
**Annotation Cluster 66**	**Enrichment Score: 1.5**	**Count**	**P_Value**
	UP_KEYWORDS	Metal-binding	231	6.6E-4
	GOTERM_MF_DIRECT	zinc ion binding	84	2.4E-2
	GOTERM_MF_DIRECT	metal ion binding	230	6.4E-2
	UP_KEYWORDS	Zinc	129	1.4E-1
	UP_KEYWORDS	Zinc-finger	95	2.3E-1
**Annotation Cluster 67**	**Enrichment Score: 1.49**	**Count**	**P_Value**
	INTERPRO	Elongation factor G, III-V domain	4	3.5E-3
	INTERPRO	Translation elongation factor EFG, V domain	4	3.5E-3
	SMART	SM00838	4	3.9E-3
	INTERPRO	Ribosomal protein S5 domain 2-type fold, subgroup	7	5.0E-3
	INTERPRO	Ribosomal protein S5 domain 2-type fold	8	8.0E-3
	INTERPRO	Translation elongation factor EFG/EF2, domain IV	3	1.9E-2
	SMART	SM00889	3	3.1E-2
	INTERPRO	Elongation factor, GTP-binding domain	4	1.1E-1
	GOTERM_MF_DIRECT	translation elongation factor activity	4	2.4E-1

	INTERPRO	Translation elongation/initiation factor/Ribosomal, beta-barrel	4	2.4E-1
	UP_KEYWORDS	Elongation factor	3	3.7E-1
	UP_KEYWORDS	Protein biosynthesis	10	4.3E-1
**Annotation Cluster 68**		**Enrichment Score: 1.49**	**Count**	**P_Value**
	INTERPRO	Interferon regulatory factor, conserved site	4	1.3E-2
	INTERPRO	Interferon regulatory factor DNA-binding domain	4	1.3E-2
	UP_SEQ_FEATURE	DNA-binding region:Tryptophan pentad repeat	4	1.4E-2
	GOTERM_MF_DIRECT	regulatory region DNA binding	5	1.5E-2
	SMART	IRF	4	2.6E-2
	INTERPRO	SMAD/FHA domain	8	3.1E-2
	INTERPRO	Interferon regulatory factor-3	3	5.9E-2
	SMART	SM01243	3	9.4E-2
	INTERPRO	SMAD domain-like	3	2.2E-1
**Annotation Cluster 69**		**Enrichment Score: 1.48**	**Count**	**P_Value**
	INTERPRO	Spectrin, beta subunit	3	1.9E-2
	PIR_SUPERFAMILY	spectrin, beta subunit	3	3.2E-2

	GOTERM_CC_DIRECT	spectrin	3	3.2E-2
	INTERPRO	Pleckstrin homology domain, spectrin-type	3	5.9E-2
**Annotation Cluster 70**	**Enrichment Score: 1.48**	**Count**	**P_Value**
	INTERPRO	Protein-tyrosine phosphatase, receptor/non-receptor type	9	2.0E-3
	GOTERM_MF_DIRECT	protein tyrosine phosphatase activity	15	2.7E-3
	SMART	PTPc	9	6.8E-3
	INTERPRO	Protein-tyrosine/Dual specificity phosphatase	12	8.3E-3
	INTERPRO	Protein-tyrosine phosphatase, active site	11	1.0E-2
	INTERPRO	Protein-tyrosine phosphatase, catalytic	10	1.1E-2
	GOTERM_BP_DIRECT	protein dephosphorylation	16	2.5E-2
	UP_KEYWORDS	Protein phosphatase	14	2.9E-2
	SMART	PTPc_motif	10	4.8E-2
	GOTERM_BP_DIRECT	peptidyl-tyrosine dephosphorylation	5	5.9E-2
	GOTERM_MF_DIRECT	phosphatase activity	13	6.4E-2

GOTERM_BP_DIRECT	dephosphorylation	12	7.7E-2
UP_SEQ_FEATURE	active site:Phosphocysteine intermediate	9	1.4E-1
GOTERM_MF_DIRECT	phosphoprotein phosphatase activity	13	1.6E-1
UP_SEQ_FEATURE	domain:Tyrosine-protein phosphatase 2	3	1.6E-1
UP_SEQ_FEATURE	domain:Tyrosine-protein phosphatase 1	3	1.6E-1
UP_SEQ_FEATURE	domain:Tyrosine-protein phosphatase	6	3.3E-1

Annotation Cluster 71	Enrichment Score: 1.48	Count	P_Value
UP_SEQ_FEATURE	domain:C2 5	3	2.1E-2
UP_SEQ_FEATURE	domain:C2 1	9	2.2E-2
UP_SEQ_FEATURE	domain:C2 2	9	2.2E-2
UP_SEQ_FEATURE	domain:C2 4	3	3.3E-2
UP_SEQ_FEATURE	domain:C2 3	3	1.2E-1

Annotation Cluster 72	Enrichment Score: 1.46	Count	P_Value
INTERPRO	EF-hand-like domain	29	3.0E-3
UP_SEQ_FEATURE	calcium-binding region:2	14	2.2E-2
INTERPRO	EF-hand domain	22	2.2E-2
UP_SEQ_FEATURE	domain:EF-hand 1	18	3.8E-2

	UP_SEQ_FEATURE	calcium-binding region:1	14	4.4E-2
	UP_SEQ_FEATURE	domain:EF-hand 2	17	6.4E-2
	INTERPRO	EF-Hand 1, calcium-binding site	16	9.1E-2
	SMART	EFh	16	1.4E-1
**Annotation Cluster 73**		**Enrichment Score: 1.44**	**Count**	**P_Value**
	KEGG_PATHWAY	Starch and sucrose metabolism	9	2.1E-3
	GOTERM_BP_DIRECT	glycogen catabolic process	5	2.3E-3
	INTERPRO	Glycogen/starch/alpha-glucan phosphorylase	3	9.9E-3
	INTERPRO	Glycosyl transferase, family 35	3	9.9E-3
	UP_SEQ_FEATURE	site:May be involved in allosteric control	3	1.1E-2
	UP_SEQ_FEATURE	site:Involved in the association of subunits	3	1.1E-2
	GOTERM_MF_DIRECT	glycogen phosphorylase activity	3	1.1E-2
	PIR_SUPERFAMILY	glucan phosphorylase	3	1.7E-2
	GOTERM_MF_DIRECT	pyridoxal phosphate binding	9	2.0E-2
	GOTERM_MF_DIRECT	phosphorylase activity	3	2.1E-2
	GOTERM_BP_DIRECT	glycogen metabolic process	6	9.3E-2

	UP_KEYWORDS	Glycogen metabolism	4	1.6E-1
	UP_SEQ_FEATURE	binding site:AMP	3	2.1E-1
	UP_KEYWORDS	Glycosyltransferase	10	8.8E-1
	UP_KEYWORDS	Carbohydrate metabolism	4	8.9E-1
	GOTERM_MF_DIRECT	transferase activity, transferring glycosyl groups	10	9.1E-1
**Annotation Cluster 74**		**Enrichment Score: 1.42**	**Count**	**P_Value**
	GOTERM_MF_DIRECT	glucose binding	6	5.1E-4
	KEGG_PATHWAY	Starch and sucrose metabolism	9	2.1E-3
	UP_KEYWORDS	Glycolysis	7	1.2E-2
	UP_SEQ_FEATURE	region of interest:Regulatory	3	2.1E-2
	GOTERM_MF_DIRECT	mannokinase activity	3	2.1E-2
	GOTERM_MF_DIRECT	fructokinase activity	3	2.1E-2
	GOTERM_BP_DIRECT	glycolytic process	7	2.3E-2
	INTERPRO	Hexokinase, C-terminal	3	3.1E-2
	INTERPRO	Hexokinase, N-terminal	3	3.1E-2
	INTERPRO	Hexokinase, conserved site	3	3.1E-2
	INTERPRO	Hexokinase	3	3.1E-2
	GOTERM_MF_DIRECT	glucokinase activity	3	3.4E-2
	GOTERM_MF_DIRECT	hexokinase activity	3	3.4E-2

KEGG_PATHWAY	Carbohydrate digestion and absorption	8	3.6E-2
GOTERM_MF_DIRECT	phosphotransferase activity, alcohol group as acceptor	5	3.8E-2
GOTERM_BP_DIRECT	carbohydrate phosphorylation	4	4.3E-2
KEGG_PATHWAY	Butirosin and neomycin biosynthesis	3	4.9E-2
UP_SEQ_FEATURE	region of interest:Catalytic	7	6.5E-2
KEGG_PATHWAY	Galactose metabolism	6	9.0E-2
KEGG_PATHWAY	Fructose and mannose metabolism	6	1.1E-1
KEGG_PATHWAY	Amino sugar and nucleotide sugar metabolism	7	1.6E-1
KEGG_PATHWAY	Type II diabetes mellitus	7	1.7E-1
COG_ONTOLOGY	Carbohydrate transport and metabolism	5	2.0E-1
GOTERM_BP_DIRECT	cellular glucose homeostasis	3	3.1E-1

	KEGG_PATHWAY	Glycolysis / Gluconeogenesis	7	3.8E-1
**Annotation Cluster 75**		**Enrichment**	**Count**	**P_Value**
	INTERPRO	Sp100	4	9.0E-3
	INTERPRO	SAND domain-like	4	3.0E-2
	INTERPRO	SAND domain	3	7.6E-2
	SMART	SAND	3	1.2E-1
**Annotation Cluster 76**		**Enrichment Score: 1.39**	**Count**	**P_Value**
	INTERPRO	Bcl-2-related protein A1	4	7.7E-4
	GOTERM_MF_DIRECT	channel activity	5	6.8E-3
	GOTERM_BP_DIRECT	intrinsic apoptotic signaling pathway in response to DNA damage	9	1.8E-2
	INTERPRO	Blc2 family	4	3.7E-2
	INTERPRO	Bcl2-like	4	4.5E-2
	GOTERM_BP_DIRECT	release of cytochrome c from mitochondria	5	7.5E-2
	INTERPRO	Apoptosis regulator, Bcl-2, BH1 motif, conserved site	3	9.4E-2
	INTERPRO	Apoptosis regulator, Bcl-2, BH2 motif, conserved site	3	9.4E-2
	GOTERM_BP_DIRECT	mitochondrial fusion	4	1.1E-1
	GOTERM_MF_DIRECT	BH domain binding	3	1.5E-1

	GOTERM_BP_DIRECT	extrinsic apoptotic signaling pathway in absence of ligand	5	2.6E-1
**Annotation Cluster 77**		**Enrichment Score: 1.39**	**Count**	**P_Value**
	UP_SEQ_FEATURE	domain:R3H	4	2.0E-2
	INTERPRO	Single-stranded nucleic acid binding R3H	4	2.3E-2
	SMART	R3H	3	1.5E-1
**Annotation Cluster 78**		**Enrichment Score: 1.38**	**Count**	**P_Value**
	INTERPRO	Band 4.1 domain	10	2.0E-3
	INTERPRO	FERM central domain	10	2.0E-3
	INTERPRO	FERM/acyl-CoA-binding protein, 3-helical bundle	9	1.0E-2
	SMART	B41	10	1.1E-2
	INTERPRO	MyTH4 domain	4	1.3E-2
	INTERPRO	FERM domain	8	2.3E-2
	SMART	MyTH4	4	2.6E-2
	UP_SEQ_FEATURE	domain:FERM	6	1.5E-1
	INTERPRO	FERM conserved site	3	4.2E-1
	INTERPRO	FERM, C-terminal PH-like domain	3	4.6E-1
	INTERPRO	FERM, N-terminal	3	5.0E-1
	SMART	SM01196	3	6.0E-1
**Annotation Cluster 79**		**Enrichment Score: 1.38**	**Count**	**P_Value**

GOTERM_BP_DIRECT	peptidyl-tyrosine phosphorylation	15	3.8E-5
GOTERM_BP_DIRECT	transmembrane receptor protein tyrosine kinase signaling pathway	15	3.6E-3
GOTERM_BP_DIRECT	peptidyl-tyrosine autophosphorylation	7	4.0E-2
INTERPRO	Tyrosine-protein kinase, catalytic domain	10	4.8E-2
GOTERM_CC_DIRECT	extrinsic component of cytoplasmic side of plasma membrane	9	5.3E-2
GOTERM_MF_DIRECT	non-membrane spanning protein tyrosine kinase activity	7	5.9E-2
INTERPRO	Tyrosine-protein kinase, active site	11	6.5E-2
UP_KEYWORDS	Tyrosine-protein kinase	11	9.8E-2
GOTERM_BP_DIRECT	regulation of phagocytosis	3	1.0E-1
GOTERM_BP_DIRECT	protein autophosphorylation	17	1.1E-1

	INTERPRO	Serine-threonine/tyrosine-protein kinase catalytic domain	13	1.2E-1
	GOTERM_MF_DIRECT	protein tyrosine kinase activity	12	1.3E-1
	SMART	TyrKc	10	1.6E-1
	INTERPRO	Protein kinase, ATP binding site	26	3.8E-1
**Annotation Cluster 80**		**Enrichment Score: 1.36**	**Count**	**P_Value**
	GOTERM_BP_DIRECT	positive regulation of transcription elongation from RNA polymerase II promoter	6	5.2E-3
	GOTERM_CC_DIRECT	Cdc73/Paf1 complex	3	6.3E-2
	GOTERM_BP_DIRECT	negative regulation of myeloid cell differentiation	4	2.5E-1
**Annotation Cluster 81**		**Enrichment Score: 1.36**	**Count**	**P_Value**
	GOTERM_BP_DIRECT	leukocyte cell-cell adhesion	7	2.9E-3
	BIOCARTA	Neutrophil and Its Surface Molecules	4	4.7E-2
	BIOCARTA	Monocyte and its Surface Molecules	4	1.1E-1

	BIOCARTA	Adhesion Molecules on Lymphocyte	3	2.5E-1
**Annotation Cluster 82**		**Enrichment Score: 1.35**	**Count**	**P_Value**
	UP_SEQ_FEATURE	repeat:TPR 13	4	2.1E-3
	UP_SEQ_FEATURE	repeat:TPR 14	4	2.1E-3
	UP_SEQ_FEATURE	repeat:TPR 11	5	2.2E-3
	UP_SEQ_FEATURE	repeat:TPR 12	4	4.0E-3
	UP_SEQ_FEATURE	repeat:TPR 16	3	1.1E-2
	UP_SEQ_FEATURE	repeat:TPR 10	5	1.4E-2
	UP_SEQ_FEATURE	repeat:TPR 15	3	2.1E-2
	UP_SEQ_FEATURE	repeat:TPR 7	8	3.5E-2
	UP_SEQ_FEATURE	repeat:TPR 9	5	4.9E-2
	UP_SEQ_FEATURE	repeat:TPR 6	8	5.8E-2
	UP_SEQ_FEATURE	repeat:TPR 8	6	8.7E-2
	UP_SEQ_FEATURE	repeat:TPR 5	8	9.5E-2
	UP_KEYWORDS	TPR repeat	13	1.1E-1
	UP_SEQ_FEATURE	repeat:TPR 2	13	1.3E-1
	UP_SEQ_FEATURE	repeat:TPR 1	13	1.3E-1
	UP_SEQ_FEATURE	repeat:TPR 3	12	1.4E-1
	INTERPRO	Tetratricopeptide-like helical	17	1.5E-1
	UP_SEQ_FEATURE	repeat:TPR 4	9	1.5E-1
	INTERPRO	Tetratricopeptide repeat-containing domain	10	2.7E-1

	INTERPRO	Tetratricopeptide repeat	9	4.6E-1
	SMART	TPR	9	6.9E-1
**Annotation Cluster 83**		**Enrichment Score: 1.3**	**Count**	**P_Value**
	INTERPRO	Ras-association	8	9.2E-3
	UP_SEQ_FEATURE	domain:Ras-associating	6	5.4E-2
	SMART	RA	6	6.5E-2
	INTERPRO	SARAH domain	3	9.4E-2
	UP_SEQ_FEATURE	domain:SARAH	3	1.0E-1
**Annotation Cluster 84**		**Enrichment Score: 1.3**	**Count**	**P_Value**
	INTERPRO	Transcription factor jumonji, JmjN	5	1.2E-3
	UP_SEQ_FEATURE	domain:JmjN	5	1.4E-3
	SMART	JmjN	5	3.1E-3
	INTERPRO	Zinc finger, FYVE/PHD-type	16	1.4E-2
	INTERPRO	Lysine-specific demethylase-like domain	3	1.9E-2
	INTERPRO	Zinc finger, C5HC2-type	3	3.1E-2
	UP_SEQ_FEATURE	domain:ARID	4	3.3E-2
	INTERPRO	Zinc finger, PHD-type	11	4.1E-2
	UP_KEYWORDS	Dioxygenase	10	4.1E-2
	INTERPRO	JmjC domain	6	4.2E-2
	UP_SEQ_FEATURE	domain:JmjC	6	4.8E-2
	INTERPRO	ARID/BRIGHT DNA-binding domain	4	5.4E-2
	GOTERM_MF_DIRECT	dioxygenase activity	10	6.1E-2

UP_SEQ_FEATURE	zinc finger region:PHD-type 2	5	6.3E-2
GOTERM_BP_DIRECT	histone H3-K4 demethylation	3	6.6E-2
SMART	JmjC	6	8.2E-2
UP_SEQ_FEATURE	zinc finger region:PHD-type 1	5	8.9E-2
SMART	BRIGHT	4	1.0E-1
UP_KEYWORDS	Iron	27	1.5E-1
SMART	PHD	11	1.5E-1
INTERPRO	Zinc finger, PHD-finger	8	1.7E-1
GOTERM_MF_DIRECT	histone demethylase activity (H3-K9 specific)	3	1.7E-1
GOTERM_BP_DIRECT	histone H3-K9 demethylation	3	1.9E-1
UP_SEQ_FEATURE	metal ion-binding site:Iron; catalytic	4	2.1E-1
INTERPRO	Zinc finger, PHD-type, conserved site	6	3.2E-1

	GOTERM_MF_DIRECT	oxidoreductase activity, acting on paired donors, with incorporation or reduction of molecular oxygen, 2-oxoglutarate as one donor, and incorporation of one atom each of oxygen into both donors	3	4.7E-1
**Annotation Cluster 85**		**Enrichment Score: 1.27**	**Count**	**P_Value**
	GOTERM_MF_DIRECT	microtubule minus-end binding	4	1.1E-2
	GOTERM_BP_DIRECT	meiotic nuclear division	6	1.5E-2
	GOTERM_BP_DIRECT	cytoplasmic microtubule organization	7	2.3E-2
	INTERPRO	Spc97/Spc98	3	3.1E-2
	GOTERM_BP_DIRECT	centrosome duplication	5	4.5E-2
	GOTERM_CC_DIRECT	equatorial microtubule organizing center	3	4.7E-2

	GOTERM_BP_DIRECT	interphase microtubule nucleation by interphase microtubule organizing center	3	4.9E-2
	GOTERM_BP_DIRECT	microtubule nucleation	4	7.4E-2
	GOTERM_MF_DIRECT	gamma-tubulin binding	5	9.4E-2
	GOTERM_BP_DIRECT	mitotic spindle assembly	4	3.7E-1
	GOTERM_BP_DIRECT	microtubule cytoskeleton organization	8	3.9E-1
**Annotation Cluster 86**		**Enrichment Score: 1.26**	**Count**	**P_Value**
	GOTERM_BP_DIRECT	toll-like receptor signaling pathway	7	3.9E-4
	GOTERM_BP_DIRECT	microglial cell activation	7	5.6E-4
	GOTERM_BP_DIRECT	cellular response to diacyl bacterial lipopeptide	4	2.2E-3
	GOTERM_MF_DIRECT	lipopeptide binding	4	2.2E-3
	INTERPRO	Toll/interleukin-1 receptor homology (TIR) domain	7	3.4E-3
	INTERPRO	Toll-like receptor	3	9.9E-3

GOTERM_CC_DIRECT	Toll-like receptor 2-Toll-like receptor 6 protein complex	3	1.1E-2
GOTERM_BP_DIRECT	response to bacterial lipoprotein	3	1.1E-2
GOTERM_BP_DIRECT	MyD88-dependent toll-like receptor signaling pathway	5	1.5E-2
UP_SEQ_FEATURE	domain:TIR	6	1.6E-2
PIR_SUPERFAMILY	toll-like receptor, 1/2/4/6/10 types [Parent=PIRSF800008]	3	1.7E-2
INTERPRO	Leucine rich repeat 4	5	2.3E-2
SMART	TIR	6	2.7E-2
GOTERM_BP_DIRECT	regulation of cytokine secretion	4	2.7E-2
UP_SEQ_FEATURE	repeat:LRR 14	8	4.9E-2
UP_KEYWORDS	Leucine-rich repeat	23	5.6E-2
UP_SEQ_FEATURE	repeat:LRR 15	7	7.1E-2
UP_SEQ_FEATURE	repeat:LRR 13	8	1.0E-1
UP_SEQ_FEATURE	repeat:LRR 16	6	1.0E-1
UP_SEQ_FEATURE	repeat:LRR 7	15	1.0E-1
INTERPRO	Leucine-rich repeat	21	1.3E-1
UP_SEQ_FEATURE	repeat:LRR 9	12	1.3E-1
UP_SEQ_FEATURE	repeat:LRR 5	18	1.4E-1
UP_SEQ_FEATURE	repeat:LRR 19	4	1.5E-1

INTERPRO	Leucine-rich repeat, typical subtype	15	1.5E-1
UP_SEQ_FEATURE	repeat:LRR 4	19	1.6E-1
UP_SEQ_FEATURE	repeat:LRR 17	5	1.6E-1
UP_SEQ_FEATURE	repeat:LRR 2	22	1.7E-1
UP_SEQ_FEATURE	repeat:LRR 1	22	1.7E-1
UP_SEQ_FEATURE	repeat:LRR 11	9	1.7E-1
UP_SEQ_FEATURE	repeat:LRR 8	12	2.0E-1
UP_SEQ_FEATURE	repeat:LRR 3	20	2.1E-1
UP_SEQ_FEATURE	repeat:LRR 12	8	2.1E-1
UP_SEQ_FEATURE	repeat:LRR 18	4	2.1E-1
UP_SEQ_FEATURE	repeat:LRR 21	3	2.3E-1
UP_SEQ_FEATURE	repeat:LRR 6	15	2.8E-1
INTERPRO	Cysteine-rich flanking region, C-terminal	7	2.9E-1
UP_SEQ_FEATURE	repeat:LRR 10	9	2.9E-1
UP_SEQ_FEATURE	repeat:LRR 20	3	3.3E-1
SMART	LRR_TYP	15	4.6E-1
SMART	LRRCT	7	5.1E-1

**Annotation Cluster 87**	**Enrichment Score: 1.26**	**Count**	**P_Value**
UP_SEQ_FEATURE	domain:PLD phosphodiesterase 2	3	4.7E-2
UP_SEQ_FEATURE	domain:PLD phosphodiesterase 1	3	4.7E-2

	INTERPRO	Phospholipase D/Transphosphatidylase	3	5.9E-2
	SMART	PLDc	3	7.1E-2
**Annotation Cluster 88**		**Enrichment Score: 1.25**	**Count**	**P_Value**
	INTERPRO	DEP domain	5	3.2E-2
	SMART	DEP	5	7.0E-2
	UP_SEQ_FEATURE	domain:DEP	4	8.2E-2
**Annotation Cluster 89**		**Enrichment Score: 1.23**	**Count**	**P_Value**
	GOTERM_MF_DIRECT	lipopolysaccharide binding	5	3.8E-2
	GOTERM_MF_DIRECT	lipoteichoic acid binding	3	4.9E-2
	GOTERM_BP_DIRECT	cellular response to lipoteichoic acid	3	1.0E-1
**Annotation Cluster 90**		**Enrichment Score: 1.23**	**Count**	**P_Value**
	UP_KEYWORDS	Pyridoxal phosphate	10	5.7E-3
	GOTERM_BP_DIRECT	cellular amino acid metabolic process	6	1.5E-2
	GOTERM_MF_DIRECT	pyridoxal phosphate binding	9	2.0E-2
	INTERPRO	Pyridoxal phosphate-dependent transferase, major region, subdomain 1	6	1.1E-1
	INTERPRO	Pyridoxal phosphate-dependent transferase	6	1.1E-1

	KEGG_PATHWAY	Glycine, serine and threonine metabolism	5	3.6E-1
	INTERPRO	Pyridoxal phosphate-dependent transferase, major region, subdomain 2	4	3.9E-1
**Annotation Cluster 91**		**Enrichment Score: 1.21**	**Count**	**P_Value**
	GOTERM_BP_DIRECT	double-strand break repair	10	1.6E-2
	UP_KEYWORDS	DNA recombination	8	9.4E-2
	GOTERM_BP_DIRECT	DNA recombination	9	1.6E-1
**Annotation Cluster 92**		**Enrichment Score: 1.2**	**Count**	**P_Value**
	UP_SEQ_FEATURE	domain:LIM zinc-binding	5	2.1E-2
	UP_KEYWORDS	LIM domain	9	5.3E-2
	INTERPRO	Zinc finger, LIM-type	9	6.8E-2
	SMART	LIM	9	2.0E-1
**Annotation Cluster 93**		**Enrichment Score: 1.2**	**Count**	**P_Value**
	GOTERM_MF_DIRECT	phosphatidylinositol binding	14	3.4E-3
	GOTERM_MF_DIRECT	superoxide-generating NADPH oxidase activator activity	3	6.6E-2
	INTERPRO	Phox homologous domain	7	7.7E-2
	SMART	PX	7	9.6E-2

	UP_SEQ_FEATURE	domain:PX	6	1.2E-1
	GOTERM_MF_DIRECT	phosphatidylinositol-3-phosphate binding	4	3.2E-1
**Annotation Cluster 94**		**Enrichment Score: 1.19**	**Count**	**P_Value**
	INTERPRO	Rhodanese-like domain	5	4.9E-2
	UP_SEQ_FEATURE	domain:Rhodanese	4	6.0E-2
	SMART	RHOD	5	9.3E-2
**Annotation Cluster 95**		**Enrichment Score: 1.18**	**Count**	**P_Value**
	GOTERM_BP_DIRECT	telomere maintenance via recombination	5	3.5E-3
	UP_KEYWORDS	Telomere	5	1.8E-1
	GOTERM_CC_DIRECT	chromosome, telomeric region	5	4.7E-1
**Annotation Cluster 96**		**Enrichment**	**Count**	**P_Value**
	INTERPRO	Coronin	3	5.9E-2
	INTERPRO	Domain of unknown function DUF1899	3	5.9E-2
	SMART	SM01166	3	9.4E-2
**Annotation Cluster 97**		**Enrichment Score: 1.09**	**Count**	**P_Value**
	UP_SEQ_FEATURE	active site:For GATase activity	3	6.4E-2
	UP_KEYWORDS	Glutamine amidotransferase	3	6.9E-2
	GOTERM_BP_DIRECT	glutamine metabolic process	4	1.3E-1
**Annotation Cluster 98**		**Enrichment Score: 1.07**	**Count**	**P_Value**
	GOTERM_BP_DIRECT	adhesion of symbiont to host	4	1.5E-2

	GOTERM_CC_DIRECT	symbiont-containing vacuole membrane	3	8.1E-2
	INTERPRO	Guanylate-binding protein, C-terminal	3	2.0E-1
	INTERPRO	Guanylate-binding protein, N-terminal	3	2.2E-1
**Annotation Cluster 99**		**Enrichment Score: 1.07**	**Count**	**P_Value**
	UP_KEYWORDS	Transferase	124	5.4E-4
	UP_KEYWORDS	Kinase	60	1.2E-3
	GOTERM_MF_DIRECT	kinase activity	60	5.2E-3
	GOTERM_BP_DIRECT	phosphorylation	54	9.5E-3
	GOTERM_MF_DIRECT	transferase activity	112	2.0E-2
	GOTERM_MF_DIRECT	protein kinase activity	43	6.6E-2
	INTERPRO	Protein kinase-like domain	41	1.1E-1
	UP_SEQ_FEATURE	binding site:ATP	42	1.9E-1
	UP_SEQ_FEATURE	active site:Proton acceptor	49	2.5E-1
	GOTERM_BP_DIRECT	protein phosphorylation	41	2.6E-1
	UP_KEYWORDS	Serine/threonine-protein kinase	26	3.2E-1
	INTERPRO	Protein kinase, ATP binding site	26	3.8E-1
	INTERPRO	Protein kinase, catalytic domain	33	4.0E-1

	UP_SEQ_FEATURE	domain:Protein kinase	31	5.8E-1
	GOTERM_MF_DIRECT	protein serine/threonine kinase activity	27	5.8E-1
	INTERPRO	Serine/threonine-protein kinase, active site	18	7.6E-1
	SMART	S_TKc	19	9.9E-1
**Annotation Cluster 100**		**Enrichment Score: 1.06**	**Count**	**P_Value**
	UP_KEYWORDS	Nuclease	16	4.0E-3
	GOTERM_MF_DIRECT	nuclease activity	14	2.7E-2
	UP_KEYWORDS	Endonuclease	10	3.3E-2
	GOTERM_MF_DIRECT	endonuclease activity	10	7.3E-2
	UP_KEYWORDS	Exonuclease	6	1.3E-1
	INTERPRO	Ribonuclease A, active site	4	1.4E-1
	INTERPRO	Ribonuclease A	4	2.1E-1
	INTERPRO	Ribonuclease A-domain	4	2.1E-1
	SMART	RNAse_Pc	4	3.0E-1
	GOTERM_MF_DIRECT	ribonuclease activity	4	4.1E-1
**Annotation Cluster 101**		**Enrichment Score: 1.06**	**Count**	**P_Value**
	UP_SEQ_FEATURE	zinc finger region:Phorbol-ester/DAG-type	7	3.0E-2
	INTERPRO	Protein kinase C-like, phorbol ester/diacylglycerol binding	9	3.9E-2

	SMART	C1	9	1.1E-1
	INTERPRO	Diacylglycerol/phorbol-ester binding	3	4.6E-1
**Annotation Cluster 102**		**Enrichment**	**Count**	**P_Value**
	INTERPRO	Ets domain	5	7.1E-2
	UP_SEQ_FEATURE	DNA-binding region:ETS	5	7.1E-2
	SMART	ETS	5	1.5E-1
**Annotation Cluster 103**		**Enrichment Score: 1.04**	**Count**	**P_Value**
	INTERPRO	Zinc finger, Btk motif	3	7.6E-2
	UP_SEQ_FEATURE	zinc finger region:Btk-type	3	8.2E-2
	SMART	BTK	3	1.2E-1
**Annotation Cluster 104**		**Enrichment Score: 1.04**	**Count**	**P_Value**
	INTERPRO	BTB/Kelch-associated	10	7.1E-3
	INTERPRO	Kelch-like protein, gigaxonin	8	9.2E-3
	UP_SEQ_FEATURE	repeat:Kelch 6	8	2.6E-2
	INTERPRO	Kelch repeat type 1	9	2.8E-2
	SMART	SM00875	10	3.3E-2
	PIR_SUPERFAMILY	kelch-like protein, gigaxonin type	8	3.4E-2
	UP_KEYWORDS	Kelch repeat	9	4.3E-2
	UP_SEQ_FEATURE	repeat:Kelch 5	8	5.3E-2
	UP_SEQ_FEATURE	repeat:Kelch 4	8	6.7E-2
	UP_SEQ_FEATURE	repeat:Kelch 3	8	7.8E-2
	UP_SEQ_FEATURE	repeat:Kelch 1	8	7.8E-2
	UP_SEQ_FEATURE	repeat:Kelch 2	8	7.8E-2
	UP_SEQ_FEATURE	domain:BACK	5	8.0E-2

	SMART	Kelch	8	1.3E-1
	GOTERM_CC_DIRECT	Cul3-RING ubiquitin ligase complex	7	2.2E-1
	INTERPRO	Galactose oxidase, beta-propeller	4	2.4E-1
	INTERPRO	Kelch-type beta propeller	5	3.9E-1
	UP_SEQ_FEATURE	domain:BTB	11	4.3E-1
	INTERPRO	BTB/POZ-like	13	6.6E-1
	INTERPRO	BTB/POZ fold	13	7.2E-1
	SMART	BTB	13	9.1E-1
**Annotation Cluster 105**		**Enrichment Score: 1.03**	**Count**	**P_Value**
	GOTERM_BP_DIRECT	very long-chain fatty acid metabolic process	5	1.5E-2
	GOTERM_CC_DIRECT	peroxisome	15	2.8E-2
	UP_KEYWORDS	Peroxisome	11	7.4E-2
	GOTERM_CC_DIRECT	peroxisomal membrane	5	4.0E-1
	KEGG_PATHWAY	Peroxisome	7	6.1E-1
**Annotation Cluster 106**		**Enrichment Score: 1.02**	**Count**	**P_Value**
	UP_KEYWORDS	Oxidoreductase	45	8.3E-2
	GOTERM_MF_DIRECT	oxidoreductase activity	47	9.1E-2
	GOTERM_BP_DIRECT	oxidation-reduction process	51	1.2E-1

StringDB enriched biological processes
Input: top 300 enriched MacTRAP translational signatures

#term ID	term description	obs. Genes	FDR
GO:0002376	immune system process	111	2.18E-45
GO:0006955	immune response	80	2.48E-39
GO:0002682	regulation of immune system process	80	2.12E-32
GO:0006952	defense response	74	1.17E-29
GO:0002684	positive regulation of immune system process	62	8.14E-28
GO:0050776	regulation of immune response	54	5.77E-25
GO:0045087	innate immune response	49	8.14E-24
GO:0048584	positive regulation of response to stimulus	85	3.57E-22
GO:0030029	actin filament-based process	43	8.53E-21
GO:0001775	cell activation	46	9.63E-21
GO:0048583	regulation of response to stimulus	114	3.28E-20
GO:0006954	inflammatory response	41	1.56E-19
GO:0045321	leukocyte activation	41	3.05E-19
GO:0050778	positive regulation of immune response	40	3.07E-19
GO:0030036	actin cytoskeleton organization	38	2.35E-18
GO:0002252	immune effector process	37	4.59E-18
GO:0002250	adaptive immune response	31	1.15E-17
GO:0002443	leukocyte mediated immunity	25	1.20E-16
GO:0002274	myeloid leukocyte activation	22	3.74E-16
GO:0048518	positive regulation of biological process	135	4.36E-16
GO:0050896	response to stimulus	152	3.18E-15
GO:0006950	response to stress	91	1.12E-14
GO:0002253	activation of immune response	26	1.31E-14
GO:0002764	immune response-regulating signaling pathway	24	2.45E-14
GO:0007015	actin filament organization	24	5.63E-14
GO:0016192	vesicle-mediated transport	50	6.53E-14
GO:0006909	phagocytosis	20	8.91E-14
GO:0032879	regulation of localization	83	9.04E-14
GO:0007010	cytoskeleton organization	47	9.86E-14
GO:0002449	lymphocyte mediated immunity	19	1.31E-12
GO:0032101	regulation of response to external stimulus	39	1.31E-12
GO:0050789	regulation of biological process	185	1.45E-12
GO:0006897	endocytosis	31	1.90E-12
GO:0001817	regulation of cytokine production	36	2.48E-12
GO:0009605	response to external stimulus	64	4.68E-12
GO:0008064	regulation of actin polymerization or depolymerization	20	6.31E-12
GO:0002694	regulation of leukocyte activation	32	6.43E-12
GO:0050865	regulation of cell activation	33	9.34E-12
GO:0002460	adaptive immune response based on somatic recombination of immur	19	1.35E-11
GO:0032103	positive regulation of response to external stimulus	25	1.42E-11
GO:0050900	leukocyte migration	21	1.52E-11
GO:0065007	biological regulation	189	1.87E-11
GO:0031347	regulation of defense response	33	2.02E-11
GO:0002757	immune response-activating signal transduction	20	2.22E-11
GO:0048002	antigen processing and presentation of peptide antigen	12	2.32E-11

GO:0031349	positive regulation of defense response	24	2.56E-11
GO:0006928	movement of cell or subcellular component	50	2.97E-11
GO:0048522	positive regulation of cellular process	115	2.97E-11
GO:0060326	cell chemotaxis	20	3.99E-11
GO:0030595	leukocyte chemotaxis	17	4.79E-11
GO:0110053	regulation of actin filament organization	22	7.49E-11
GO:0034341	response to interferon-gamma	17	8.60E-11
GO:0002478	antigen processing and presentation of exogenous peptide antigen	10	1.24E-10
GO:0001819	positive regulation of cytokine production	27	1.37E-10
GO:0032956	regulation of actin cytoskeleton organization	24	2.12E-10
GO:0051049	regulation of transport	60	2.29E-10
GO:0016064	immunoglobulin mediated immune response	14	2.31E-10
GO:0030593	neutrophil chemotaxis	13	2.43E-10
GO:0032970	regulation of actin filament-based process	25	3.98E-10
GO:0071346	cellular response to interferon-gamma	15	5.68E-10
GO:0007166	cell surface receptor signaling pathway	57	6.53E-10
GO:0051716	cellular response to stimulus	116	6.58E-10
GO:0097435	supramolecular fiber organization	26	6.95E-10
GO:0050794	regulation of cellular process	170	7.91E-10
GO:0002683	negative regulation of immune system process	27	8.35E-10
GO:0007165	signal transduction	91	9.64E-10
GO:0070887	cellular response to chemical stimulus	68	1.14E-09
GO:0002366	leukocyte activation involved in immune response	17	1.26E-09
GO:0002768	immune response-regulating cell surface receptor signaling pathway	16	1.26E-09
GO:0007154	cell communication	97	1.26E-09
GO:0023052	signaling	95	1.26E-09
GO:0006935	chemotaxis	27	1.30E-09
GO:0051707	response to other organism	36	2.06E-09
GO:0030155	regulation of cell adhesion	32	2.20E-09
GO:0046649	lymphocyte activation	25	2.23E-09
GO:0032535	regulation of cellular component size	24	2.85E-09
GO:0034097	response to cytokine	36	2.98E-09
GO:0030833	regulation of actin filament polymerization	16	3.39E-09
GO:0040011	locomotion	42	4.67E-09
GO:0002703	regulation of leukocyte mediated immunity	18	5.21E-09
GO:0051249	regulation of lymphocyte activation	25	5.21E-09
GO:0030834	regulation of actin filament depolymerization	11	5.86E-09
GO:0030835	negative regulation of actin filament depolymerization	10	8.04E-09
GO:0045576	mast cell activation	9	9.60E-09
GO:0071345	cellular response to cytokine stimulus	32	1.28E-08
GO:0002886	regulation of myeloid leukocyte mediated immunity	11	1.41E-08
GO:0032680	regulation of tumor necrosis factor production	15	2.66E-08
GO:0051046	regulation of secretion	34	3.39E-08
GO:0051179	localization	98	3.39E-08
GO:0016477	cell migration	33	4.65E-08
GO:0042221	response to chemical	85	5.08E-08
GO:0002275	myeloid cell activation involved in immune response	10	5.59E-08
GO:0065008	regulation of biological quality	83	5.75E-08
GO:1902904	negative regulation of supramolecular fiber organization	14	5.75E-08
GO:0009617	response to bacterium	28	6.13E-08

GO:0051050	positive regulation of transport	38	6.13E-08
GO:0019886	antigen processing and presentation of exogenous peptide antigen via	7	6.22E-08
GO:0060627	regulation of vesicle-mediated transport	26	6.37E-08
GO:0050863	regulation of T cell activation	20	6.43E-08
GO:0048870	cell motility	35	7.41E-08
GO:0006911	phagocytosis, engulfment	9	7.68E-08
GO:0071216	cellular response to biotic stimulus	16	1.09E-07
GO:0051494	negative regulation of cytoskeleton organization	14	1.11E-07
GO:0040017	positive regulation of locomotion	27	1.30E-07
GO:0006810	transport	78	1.34E-07
GO:0002697	regulation of immune effector process	21	1.36E-07
GO:0051234	establishment of localization	80	1.49E-07
GO:0002819	regulation of adaptive immune response	15	1.51E-07
GO:2000147	positive regulation of cell motility	26	1.63E-07
GO:0030097	hemopoiesis	28	1.78E-07
GO:0002520	immune system development	30	2.09E-07
GO:0002685	regulation of leukocyte migration	16	2.16E-07
GO:0002688	regulation of leukocyte chemotaxis	13	2.34E-07
GO:0050867	positive regulation of cell activation	20	2.34E-07
GO:0045785	positive regulation of cell adhesion	22	2.41E-07
GO:0032940	secretion by cell	22	2.50E-07
GO:0071310	cellular response to organic substance	54	2.50E-07
GO:0009966	regulation of signal transduction	71	2.58E-07
GO:0050920	regulation of chemotaxis	16	2.68E-07
GO:0070663	regulation of leukocyte proliferation	17	2.70E-07
GO:0002699	positive regulation of immune effector process	16	2.82E-07
GO:0002687	positive regulation of leukocyte migration	14	2.85E-07
GO:0007018	microtubule-based movement	18	2.95E-07
GO:0030335	positive regulation of cell migration	25	2.97E-07
GO:0002444	myeloid leukocyte mediated immunity	9	3.06E-07
GO:0046903	secretion	25	3.06E-07
GO:0022409	positive regulation of cell-cell adhesion	17	3.09E-07
GO:1903708	positive regulation of hemopoiesis	16	3.28E-07
GO:0051270	regulation of cellular component movement	35	3.48E-07
GO:0002675	positive regulation of acute inflammatory response	8	3.54E-07
GO:0030837	negative regulation of actin filament polymerization	9	3.54E-07
GO:0043299	leukocyte degranulation	8	3.54E-07
GO:0022407	regulation of cell-cell adhesion	21	3.59E-07
GO:0090066	regulation of anatomical structure size	25	3.71E-07
GO:0050764	regulation of phagocytosis	11	4.17E-07
GO:1903037	regulation of leukocyte cell-cell adhesion	18	4.17E-07
GO:0051693	actin filament capping	8	4.24E-07
GO:0002696	positive regulation of leukocyte activation	19	5.00E-07
GO:0032760	positive regulation of tumor necrosis factor production	11	5.12E-07
GO:0071222	cellular response to lipopolysaccharide	14	5.12E-07
GO:0050727	regulation of inflammatory response	19	5.16E-07
GO:0045088	regulation of innate immune response	17	6.57E-07
GO:0050670	regulation of lymphocyte proliferation	16	7.95E-07
GO:0051128	regulation of cellular component organization	61	8.70E-07
GO:0040012	regulation of locomotion	34	8.76E-07

GO:0002429	immune response-activating cell surface receptor signaling pathway	12	8.82E-07
GO:0030100	regulation of endocytosis	17	8.82E-07
GO:0002237	response to molecule of bacterial origin	19	9.40E-07
GO:0080134	regulation of response to stress	39	9.52E-07
GO:1902107	positive regulation of leukocyte differentiation	14	9.67E-07
GO:1903039	positive regulation of leukocyte cell-cell adhesion	15	9.68E-07
GO:0030048	actin filament-based movement	10	1.17E-06
GO:0051240	positive regulation of multicellular organismal process	49	1.21E-06
GO:0009967	positive regulation of signal transduction	44	1.23E-06
GO:2000145	regulation of cell motility	32	1.25E-06
GO:0050729	positive regulation of inflammatory response	12	1.41E-06
GO:0051017	actin filament bundle assembly	9	1.41E-06
GO:0002690	positive regulation of leukocyte chemotaxis	11	1.42E-06
GO:0016043	cellular component organization	96	1.45E-06
GO:0090022	regulation of neutrophil chemotaxis	8	1.45E-06
GO:0050921	positive regulation of chemotaxis	13	1.48E-06
GO:0031032	actomyosin structure organization	11	1.54E-06
GO:0010647	positive regulation of cell communication	47	1.63E-06
GO:0023056	positive regulation of signaling	47	1.84E-06
GO:0006887	exocytosis	15	1.89E-06
GO:0032496	response to lipopolysaccharide	18	1.90E-06
GO:0002822	regulation of adaptive immune response based on somatic recombina	13	1.92E-06
GO:1903530	regulation of secretion by cell	29	1.92E-06
GO:0002673	regulation of acute inflammatory response	9	2.01E-06
GO:0070374	positive regulation of ERK1 and ERK2 cascade	15	2.06E-06
GO:0043254	regulation of protein complex assembly	21	2.14E-06
GO:0010646	regulation of cell communication	73	2.15E-06
GO:1903706	regulation of hemopoiesis	20	2.60E-06
GO:0023051	regulation of signaling	73	2.73E-06
GO:0050870	positive regulation of T cell activation	14	2.82E-06
GO:0010033	response to organic substance	63	2.98E-06
GO:0032675	regulation of interleukin-6 production	12	3.37E-06
GO:0030334	regulation of cell migration	30	3.69E-06
GO:0032880	regulation of protein localization	33	3.84E-06
GO:0038093	Fc receptor signaling pathway	6	3.84E-06
GO:0045807	positive regulation of endocytosis	13	3.89E-06
GO:0045089	positive regulation of innate immune response	13	4.14E-06
GO:1902105	regulation of leukocyte differentiation	17	4.43E-06
GO:0044087	regulation of cellular component biogenesis	31	4.81E-06
GO:0002821	positive regulation of adaptive immune response	11	4.92E-06
GO:0051251	positive regulation of lymphocyte activation	16	4.94E-06
GO:0002695	negative regulation of leukocyte activation	13	5.65E-06
GO:0090023	positive regulation of neutrophil chemotaxis	7	6.42E-06
GO:1902533	positive regulation of intracellular signal transduction	32	6.95E-06
GO:0002521	leukocyte differentiation	18	1.03E-05
GO:0038094	Fc-gamma receptor signaling pathway	5	1.26E-05
GO:0072676	lymphocyte migration	8	1.39E-05
GO:0051047	positive regulation of secretion	21	1.41E-05
GO:0045730	respiratory burst	5	1.78E-05
GO:1902531	regulation of intracellular signal transduction	44	1.84E-05

GO:0001906	cell killing	8	1.97E-05
GO:1903532	positive regulation of secretion by cell	20	2.00E-05
GO:0098542	defense response to other organism	19	2.04E-05
GO:0002824	positive regulation of adaptive immune response based on somatic re	10	2.17E-05
GO:0045621	positive regulation of lymphocyte differentiation	10	2.34E-05
GO:1905475	regulation of protein localization to membrane	12	2.38E-05
GO:0001805	positive regulation of type III hypersensitivity	4	2.39E-05
GO:0035589	G protein-coupled purinergic nucleotide receptor signaling pathway	5	2.39E-05
GO:0042116	macrophage activation	7	2.64E-05
GO:0048247	lymphocyte chemotaxis	7	2.64E-05
GO:0051239	regulation of multicellular organismal process	65	2.64E-05
GO:0051130	positive regulation of cellular component organization	36	2.68E-05
GO:0042129	regulation of T cell proliferation	12	2.94E-05
GO:0002218	activation of innate immune response	9	3.26E-05
GO:0030099	myeloid cell differentiation	14	3.50E-05
GO:0036230	granulocyte activation	6	3.54E-05
GO:0043410	positive regulation of MAPK cascade	21	3.85E-05
GO:0002706	regulation of lymphocyte mediated immunity	11	3.99E-05
GO:0002221	pattern recognition receptor signaling pathway	8	4.05E-05
GO:0051250	negative regulation of lymphocyte activation	11	4.23E-05
GO:0051247	positive regulation of protein metabolic process	42	4.88E-05
GO:0002224	toll-like receptor signaling pathway	7	5.04E-05
GO:0045055	regulated exocytosis	10	5.41E-05
GO:0045582	positive regulation of T cell differentiation	9	5.77E-05
GO:0032729	positive regulation of interferon-gamma production	8	6.62E-05
GO:0042110	T cell activation	14	6.70E-05
GO:0043300	regulation of leukocyte degranulation	7	7.27E-05
GO:0045937	positive regulation of phosphate metabolic process	32	7.73E-05
GO:0002705	positive regulation of leukocyte mediated immunity	10	8.02E-05
GO:0050707	regulation of cytokine secretion	12	8.02E-05
GO:0001909	leukocyte mediated cytotoxicity	6	8.08E-05
GO:0045639	positive regulation of myeloid cell differentiation	9	8.39E-05
GO:0002577	regulation of antigen processing and presentation	5	8.64E-05
GO:0032649	regulation of interferon-gamma production	9	9.01E-05
GO:0002888	positive regulation of myeloid leukocyte mediated immunity	6	9.36E-05
GO:0031663	lipopolysaccharide-mediated signaling pathway	6	9.36E-05
GO:0051694	pointed-end actin filament capping	4	9.65E-05
GO:0051222	positive regulation of protein transport	18	0.0001
GO:0007186	G protein-coupled receptor signaling pathway	27	0.00012
GO:0002455	humoral immune response mediated by circulating immunoglobulin	6	0.00014
GO:0032270	positive regulation of cellular protein metabolic process	39	0.00014
GO:0042590	antigen processing and presentation of exogenous peptide antigen via	4	0.00014
GO:0050715	positive regulation of cytokine secretion	10	0.00014
GO:0030239	myofibril assembly	7	0.00017
GO:0050766	positive regulation of phagocytosis	7	0.00017
GO:0050793	regulation of developmental process	56	0.00017
GO:0045637	regulation of myeloid cell differentiation	12	0.00018
GO:0042119	neutrophil activation	5	0.00019
GO:0050714	positive regulation of protein secretion	14	0.00019
GO:0050868	negative regulation of T cell activation	9	0.0002

GO:0030098	lymphocyte differentiation	13	0.00026
GO:0099515	actin filament-based transport	4	0.00026
GO:0001816	cytokine production	10	0.00027
GO:0051125	regulation of actin nucleation	5	0.00027
GO:0009987	cellular process	191	0.00028
GO:0043085	positive regulation of catalytic activity	34	0.00028
GO:0050830	defense response to Gram-positive bacterium	8	0.00028
GO:1901701	cellular response to oxygen-containing compound	27	0.00028
GO:0002283	neutrophil activation involved in immune response	4	0.00034
GO:0030888	regulation of B cell proliferation	7	0.00034
GO:0031401	positive regulation of protein modification process	32	0.00034
GO:0051639	actin filament network formation	4	0.00034
GO:0001934	positive regulation of protein phosphorylation	28	0.00036
GO:0044093	positive regulation of molecular function	40	0.00036
GO:0007017	microtubule-based process	21	0.00039
GO:0070098	chemokine-mediated signaling pathway	7	0.00045
GO:0048869	cellular developmental process	71	0.00048
GO:0022603	regulation of anatomical structure morphogenesis	29	0.00051
GO:0048519	negative regulation of biological process	89	0.00051
GO:0090087	regulation of peptide transport	23	0.00051
GO:0010639	negative regulation of organelle organization	15	0.00052
GO:0002708	positive regulation of lymphocyte mediated immunity	8	0.00054
GO:0002761	regulation of myeloid leukocyte differentiation	9	0.00054
GO:0031334	positive regulation of protein complex assembly	12	0.00054
GO:0002430	complement receptor mediated signaling pathway	4	0.00056
GO:0002579	positive regulation of antigen processing and presentation	4	0.00056
GO:0006959	humoral immune response	9	0.00056
GO:0043408	regulation of MAPK cascade	23	0.00056
GO:0051764	actin crosslink formation	4	0.00056
GO:0022610	biological adhesion	23	0.00058
GO:0001818	negative regulation of cytokine production	12	0.00059
GO:0046634	regulation of alpha-beta T cell activation	8	0.00061
GO:1903076	regulation of protein localization to plasma membrane	8	0.00061
GO:0002791	regulation of peptide secretion	18	0.00062
GO:0006996	organelle organization	59	0.00065
GO:0033003	regulation of mast cell activation	6	0.00067
GO:0030154	cell differentiation	69	0.00069
GO:0001798	positive regulation of type IIa hypersensitivity	3	0.0007
GO:0001812	positive regulation of type I hypersensitivity	3	0.0007
GO:0034154	toll-like receptor 7 signaling pathway	3	0.0007
GO:0043378	positive regulation of CD8-positive, alpha-beta T cell differentiation	3	0.0007
GO:0045597	positive regulation of cell differentiation	29	0.0007
GO:1903827	regulation of cellular protein localization	18	0.0007
GO:0051094	positive regulation of developmental process	36	0.00072
GO:1901700	response to oxygen-containing compound	36	0.00078
GO:0050878	regulation of body fluid levels	14	0.00079
GO:0002548	monocyte chemotaxis	5	0.00082
GO:0006958	complement activation, classical pathway	5	0.00082
GO:0032743	positive regulation of interleukin-2 production	5	0.00082
GO:0060099	regulation of phagocytosis, engulfment	4	0.00082

GO:0050671	positive regulation of lymphocyte proliferation	9	0.0009
GO:0030168	platelet activation	6	0.00093
GO:0034122	negative regulation of toll-like receptor signaling pathway	5	0.00093
GO:0035556	intracellular signal transduction	33	0.00093
GO:0030838	positive regulation of actin filament polymerization	7	0.00094
GO:0051656	establishment of organelle localization	14	0.00096
GO:0044089	positive regulation of cellular component biogenesis	18	0.00098
GO:0070664	negative regulation of leukocyte proliferation	7	0.00099
GO:0006898	receptor-mediated endocytosis	10	0.001
GO:0051495	positive regulation of cytoskeleton organization	11	0.001
GO:0008360	regulation of cell shape	9	0.0011
GO:0032501	multicellular organismal process	103	0.0011
GO:0032663	regulation of interleukin-2 production	6	0.0011
GO:0032989	cellular component morphogenesis	23	0.0011
GO:0043303	mast cell degranulation	4	0.0011
GO:0043304	regulation of mast cell degranulation	5	0.0011
GO:0072376	protein activation cascade	6	0.0011
GO:0007155	cell adhesion	22	0.0012
GO:0032755	positive regulation of interleukin-6 production	7	0.0012
GO:0033043	regulation of organelle organization	30	0.0012
GO:0065009	regulation of molecular function	62	0.0012
GO:0071396	cellular response to lipid	17	0.0012
GO:0001776	leukocyte homeostasis	7	0.0013
GO:0001932	regulation of protein phosphorylation	34	0.0013
GO:0002573	myeloid leukocyte differentiation	8	0.0013
GO:0007162	negative regulation of cell adhesion	12	0.0013
GO:0051223	regulation of protein transport	21	0.0013
GO:0060341	regulation of cellular localization	24	0.0013
GO:0032722	positive regulation of chemokine production	6	0.0014
GO:0042113	B cell activation	9	0.0014
GO:0051704	multi-organism process	42	0.0014
GO:1902905	positive regulation of supramolecular fiber organization	10	0.0014
GO:0017157	regulation of exocytosis	10	0.0015
GO:0051640	organelle localization	16	0.0015
GO:0002763	positive regulation of myeloid leukocyte differentiation	6	0.0017
GO:0031532	actin cytoskeleton reorganization	6	0.0017
GO:0043491	protein kinase B signaling	5	0.0017
GO:0048523	negative regulation of cellular process	80	0.0017
GO:2000107	negative regulation of leukocyte apoptotic process	6	0.0017
GO:2000514	regulation of CD4-positive, alpha-beta T cell activation	6	0.0017
GO:0050708	regulation of protein secretion	16	0.0018
GO:1903305	regulation of regulated secretory pathway	8	0.0018
GO:0001820	serotonin secretion	3	0.0019
GO:0030050	vesicle transport along actin filament	3	0.0019
GO:0032507	maintenance of protein location in cell	6	0.0019
GO:0001961	positive regulation of cytokine-mediated signaling pathway	5	0.002
GO:0042742	defense response to bacterium	11	0.002
GO:0001774	microglial cell activation	4	0.0021
GO:0002532	production of molecular mediator involved in inflammatory response	4	0.0021
GO:0046635	positive regulation of alpha-beta T cell activation	6	0.0021

GO:0072678	T cell migration	4	0.0021
GO:0042325	regulation of phosphorylation	35	0.0022
GO:0019220	regulation of phosphate metabolic process	38	0.0024
GO:0061024	membrane organization	18	0.0024
GO:2000501	regulation of natural killer cell chemotaxis	3	0.0025
GO:0008154	actin polymerization or depolymerization	5	0.0027
GO:0030890	positive regulation of B cell proliferation	5	0.0027
GO:0051345	positive regulation of hydrolase activity	20	0.0027
GO:0042102	positive regulation of T cell proliferation	7	0.0029
GO:0051146	striated muscle cell differentiation	10	0.003
GO:0051641	cellular localization	40	0.003
GO:0007229	integrin-mediated signaling pathway	6	0.0031
GO:0031325	positive regulation of cellular metabolic process	59	0.0031
GO:0010818	T cell chemotaxis	3	0.0032
GO:0030316	osteoclast differentiation	5	0.0032
GO:0032502	developmental process	91	0.0032
GO:1902563	regulation of neutrophil activation	3	0.0032
GO:0033993	response to lipid	23	0.0035
GO:0055002	striated muscle cell development	8	0.0036
GO:1905477	positive regulation of protein localization to membrane	7	0.0036
GO:0046638	positive regulation of alpha-beta T cell differentiation	5	0.0038
GO:0002285	lymphocyte activation involved in immune response	7	0.0039
GO:0070527	platelet aggregation	4	0.004
GO:0007159	leukocyte cell-cell adhesion	5	0.0041
GO:0022604	regulation of cell morphogenesis	16	0.0041
GO:0002440	production of molecular mediator of immune response	6	0.0042
GO:0022607	cellular component assembly	43	0.0042
GO:0050672	negative regulation of lymphocyte proliferation	6	0.0042
GO:1903829	positive regulation of cellular protein localization	12	0.0042
GO:0002381	immunoglobulin production involved in immunoglobulin mediated imı	4	0.0045
GO:0042832	defense response to protozoan	4	0.0045
GO:0048731	system development	74	0.0045
GO:0002704	negative regulation of leukocyte mediated immunity	5	0.0048
GO:0043370	regulation of CD4-positive, alpha-beta T cell differentiation	5	0.0048
GO:0051173	positive regulation of nitrogen compound metabolic process	56	0.0048
GO:0042554	superoxide anion generation	3	0.0049
GO:0032673	regulation of interleukin-4 production	4	0.005
GO:0042060	wound healing	12	0.005
GO:0044403	symbiont process	10	0.005
GO:0051701	interaction with host	6	0.005
GO:0048513	animal organ development	58	0.0053
GO:0002377	immunoglobulin production	5	0.0059
GO:0033004	negative regulation of mast cell activation	3	0.0059
GO:0002825	regulation of T-helper 1 type immune response	4	0.0062
GO:0019221	cytokine-mediated signaling pathway	12	0.0063
GO:0045595	regulation of cell differentiation	38	0.0064
GO:0048856	anatomical structure development	85	0.0068
GO:0001788	antibody-dependent cellular cytotoxicity	2	0.0069
GO:0002344	B cell affinity maturation	2	0.0069
GO:0032796	uropod organization	2	0.0069

GO:0038178	complement component C5a signaling pathway	2	0.0069
GO:0043087	regulation of GTPase activity	14	0.0069
GO:0051127	positive regulation of actin nucleation	3	0.0069
GO:0060101	negative regulation of phagocytosis, engulfment	2	0.0069
GO:0061502	early endosome to recycling endosome transport	2	0.0069
GO:0098885	modification of postsynaptic actin cytoskeleton	2	0.0069
GO:1903923	positive regulation of protein processing in phagocytic vesicle	2	0.0069
GO:2000588	positive regulation of platelet-derived growth factor receptor-beta sig	2	0.0069
GO:1903078	positive regulation of protein localization to plasma membrane	5	0.0071
GO:0007596	blood coagulation	7	0.0072
GO:0050853	B cell receptor signaling pathway	4	0.0074
GO:0050851	antigen receptor-mediated signaling pathway	6	0.0075
GO:0010604	positive regulation of macromolecule metabolic process	57	0.0078
GO:0051129	negative regulation of cellular component organization	19	0.0078
GO:0042127	regulation of cell population proliferation	35	0.0081
GO:0009109	coenzyme catabolic process	3	0.0082
GO:0034142	toll-like receptor 4 signaling pathway	3	0.0082
GO:0009893	positive regulation of metabolic process	60	0.0083
GO:0002286	T cell activation involved in immune response	5	0.0092
GO:0031399	regulation of protein modification process	37	0.0094
GO:0048872	homeostasis of number of cells	10	0.0094
GO:0050855	regulation of B cell receptor signaling pathway	3	0.0095
GO:0051883	killing of cells in other organism involved in symbiotic interaction	3	0.0095
GO:0046718	viral entry into host cell	4	0.0096
GO:0050790	regulation of catalytic activity	42	0.01
GO:0051048	negative regulation of secretion	10	0.0101
GO:0042360	vitamin E metabolic process	2	0.0104
GO:0050704	regulation of interleukin-1 secretion	4	0.0104
GO:0071226	cellular response to molecule of fungal origin	2	0.0104
GO:0042108	positive regulation of cytokine biosynthetic process	5	0.0109
GO:0032814	regulation of natural killer cell activation	4	0.0113
GO:1901224	positive regulation of NIK/NF-kappaB signaling	5	0.0115
GO:0030865	cortical cytoskeleton organization	4	0.0123
GO:0140029	exocytic process	5	0.0123
GO:0001959	regulation of cytokine-mediated signaling pathway	6	0.0124
GO:0032611	interleukin-1 beta production	3	0.0125
GO:0051085	chaperone cofactor-dependent protein refolding	3	0.0125
GO:0009611	response to wounding	13	0.0127
GO:0033628	regulation of cell adhesion mediated by integrin	4	0.0133
GO:0032652	regulation of interleukin-1 production	5	0.0136
GO:0043269	regulation of ion transport	18	0.0136
GO:0007200	phospholipase C-activating G protein-coupled receptor signaling pathv	6	0.0141
GO:0002643	regulation of tolerance induction	3	0.0142
GO:0006691	leukotriene metabolic process	3	0.0142
GO:0032732	positive regulation of interleukin-1 production	4	0.0142
GO:0043030	regulation of macrophage activation	4	0.0142
GO:0051905	establishment of pigment granule localization	3	0.0142
GO:0071801	regulation of podosome assembly	3	0.0142
GO:0043547	positive regulation of GTPase activity	12	0.0144
GO:0010638	positive regulation of organelle organization	16	0.0145

GO:1902106	negative regulation of leukocyte differentiation	6	0.0145
GO:0002442	serotonin secretion involved in inflammatory response	2	0.0146
GO:0006742	NADP catabolic process	2	0.0146
GO:0043315	positive regulation of neutrophil degranulation	2	0.0146
GO:0046469	platelet activating factor metabolic process	2	0.0146
GO:0048549	positive regulation of pinocytosis	2	0.0146
GO:0002718	regulation of cytokine production involved in immune response	5	0.0147
GO:0002720	positive regulation of cytokine production involved in immune respons	4	0.0149
GO:0006936	muscle contraction	8	0.015
GO:0120032	regulation of plasma membrane bounded cell projection assembly	8	0.015
GO:0030852	regulation of granulocyte differentiation	3	0.0156
GO:0033033	negative regulation of myeloid cell apoptotic process	3	0.0156
GO:0045649	regulation of macrophage differentiation	3	0.0156
GO:0045652	regulation of megakaryocyte differentiation	3	0.0156
GO:0050765	negative regulation of phagocytosis	3	0.0156
GO:0072112	glomerular visceral epithelial cell differentiation	3	0.0156
GO:0051051	negative regulation of transport	15	0.0157
GO:0008347	glial cell migration	4	0.0158
GO:0034612	response to tumor necrosis factor	8	0.0158
GO:0046636	negative regulation of alpha-beta T cell activation	4	0.0158
GO:0051336	regulation of hydrolase activity	26	0.0164
GO:1903707	negative regulation of hemopoiesis	7	0.0172
GO:0051235	maintenance of location	7	0.0178
GO:0051271	negative regulation of cellular component movement	10	0.0181
GO:0032715	negative regulation of interleukin-6 production	4	0.0182
GO:0002431	Fc receptor mediated stimulatory signaling pathway	2	0.019
GO:0002636	positive regulation of germinal center formation	2	0.019
GO:0043320	natural killer cell degranulation	2	0.019
GO:0061061	muscle structure development	14	0.0192
GO:0030101	natural killer cell activation	4	0.0193
GO:0032653	regulation of interleukin-10 production	4	0.0193
GO:0043583	ear development	9	0.0201
GO:0048468	cell development	33	0.0202
GO:0003012	muscle system process	9	0.0212
GO:1905523	positive regulation of macrophage migration	3	0.0214
GO:0031341	regulation of cell killing	5	0.0215
GO:0051607	defense response to virus	7	0.0229
GO:0002827	positive regulation of T-helper 1 type immune response	3	0.0238
GO:0019058	viral life cycle	5	0.0238
GO:0030041	actin filament polymerization	3	0.0238
GO:0032753	positive regulation of interleukin-4 production	3	0.0238
GO:0021814	cell motility involved in cerebral cortex radial glia guided migration	2	0.0242
GO:0030217	T cell differentiation	7	0.0242
GO:0034162	toll-like receptor 9 signaling pathway	2	0.0242
GO:0035509	negative regulation of myosin-light-chain-phosphatase activity	2	0.0242
GO:0038095	Fc-epsilon receptor signaling pathway	2	0.0242
GO:0042989	sequestering of actin monomers	2	0.0242
GO:0043305	negative regulation of mast cell degranulation	2	0.0242
GO:0048305	immunoglobulin secretion	2	0.0242
GO:0050705	regulation of interleukin-1 alpha secretion	2	0.0242

GO:0050859	negative regulation of B cell receptor signaling pathway	2	0.0242
GO:0071800	podosome assembly	2	0.0242
GO:2000503	positive regulation of natural killer cell chemotaxis	2	0.0242
GO:0071675	regulation of mononuclear cell migration	4	0.0245
GO:1900076	regulation of cellular response to insulin stimulus	5	0.0254
GO:0002762	negative regulation of myeloid leukocyte differentiation	4	0.0261
GO:1905954	positive regulation of lipid localization	5	0.0279
GO:0010575	positive regulation of vascular endothelial growth factor production	3	0.0284
GO:0071276	cellular response to cadmium ion	3	0.0284
GO:0071356	cellular response to tumor necrosis factor	7	0.0287
GO:0048839	inner ear development	8	0.0289
GO:0051817	modification of morphology or physiology of other organism involved	5	0.0291
GO:0032720	negative regulation of tumor necrosis factor production	4	0.0295
GO:0051924	regulation of calcium ion transport	9	0.0295
GO:0051930	regulation of sensory perception of pain	4	0.0295
GO:0002576	platelet degranulation	2	0.0296
GO:0002667	regulation of T cell anergy	2	0.0296
GO:0014808	release of sequestered calcium ion into cytosol by sarcoplasmic reticul	2	0.0296
GO:0032252	secretory granule localization	2	0.0296
GO:0050665	hydrogen peroxide biosynthetic process	2	0.0296
GO:0051126	negative regulation of actin nucleation	2	0.0296
GO:0098609	cell-cell adhesion	11	0.0296
GO:0007097	nuclear migration	3	0.0305
GO:0050718	positive regulation of interleukin-1 beta secretion	3	0.0305
GO:0071248	cellular response to metal ion	7	0.0316
GO:2000026	regulation of multicellular organismal development	38	0.0319
GO:0000904	cell morphogenesis involved in differentiation	14	0.0324
GO:0035904	aorta development	4	0.0324
GO:0045638	negative regulation of myeloid cell differentiation	5	0.0324
GO:1903307	positive regulation of regulated secretory pathway	4	0.0324
GO:0002724	regulation of T cell cytokine production	3	0.033
GO:0045010	actin nucleation	3	0.033
GO:0007204	positive regulation of cytosolic calcium ion concentration	9	0.0344
GO:0007275	multicellular organism development	76	0.0347
GO:0045766	positive regulation of angiogenesis	7	0.0349
GO:0006468	protein phosphorylation	19	0.0351
GO:0006930	substrate-dependent cell migration, cell extension	2	0.0351
GO:0010829	negative regulation of glucose transmembrane transport	3	0.0351
GO:0032268	regulation of cellular protein metabolic process	45	0.0351
GO:0033625	positive regulation of integrin activation	2	0.0351
GO:0034144	negative regulation of toll-like receptor 4 signaling pathway	2	0.0351
GO:0043302	positive regulation of leukocyte degranulation	3	0.0351
GO:0043615	astrocyte cell migration	2	0.0351
GO:0045654	positive regulation of megakaryocyte differentiation	2	0.0351
GO:0048015	phosphatidylinositol-mediated signaling	4	0.0351
GO:0050957	equilibrioception	2	0.0351
GO:0051014	actin filament severing	2	0.0351
GO:0072503	cellular divalent inorganic cation homeostasis	12	0.0352
GO:0071347	cellular response to interleukin-1	5	0.0353
GO:0045921	positive regulation of exocytosis	5	0.0369

GO:0035458	cellular response to interferon-beta	3	0.0379
GO:0043368	positive T cell selection	3	0.0379
GO:0045742	positive regulation of epidermal growth factor receptor signaling path	3	0.0379
GO:0042981	regulation of apoptotic process	30	0.0389
GO:0008104	protein localization	34	0.039
GO:0070925	organelle assembly	15	0.039
GO:0032386	regulation of intracellular transport	11	0.0397
GO:0002606	positive regulation of dendritic cell antigen processing and presentatic	2	0.0408
GO:0002709	regulation of T cell mediated immunity	4	0.0408
GO:0030866	cortical actin cytoskeleton organization	3	0.0408
GO:0031622	positive regulation of fever generation	2	0.0408
GO:0032623	interleukin-2 production	2	0.0408
GO:0032677	regulation of interleukin-8 production	4	0.0408
GO:0034138	toll-like receptor 3 signaling pathway	2	0.0408
GO:0043372	positive regulation of CD4-positive, alpha-beta T cell differentiation	3	0.0408
GO:0070493	thrombin-activated receptor signaling pathway	2	0.0408
GO:0071705	nitrogen compound transport	29	0.0408
GO:1900165	negative regulation of interleukin-6 secretion	2	0.0408
GO:0009615	response to virus	8	0.0415
GO:0051246	regulation of protein metabolic process	47	0.0421
GO:0055013	cardiac muscle cell development	4	0.0421
GO:0043900	regulation of multi-organism process	11	0.0424
GO:0032733	positive regulation of interleukin-10 production	3	0.0432
GO:2000515	negative regulation of CD4-positive, alpha-beta T cell activation	3	0.0432
GO:0032231	regulation of actin filament bundle assembly	5	0.0433
GO:0015696	ammonium transport	4	0.0439
GO:0042130	negative regulation of T cell proliferation	4	0.0439
GO:0030183	B cell differentiation	5	0.0449
GO:0009306	protein secretion	6	0.0453
GO:0001910	regulation of leukocyte mediated cytotoxicity	4	0.0459
GO:0001782	B cell homeostasis	3	0.0461
GO:0009653	anatomical structure morphogenesis	39	0.0461
GO:0030853	negative regulation of granulocyte differentiation	2	0.0462
GO:0030854	positive regulation of granulocyte differentiation	2	0.0462
GO:0045410	positive regulation of interleukin-6 biosynthetic process	2	0.0462
GO:0048245	eosinophil chemotaxis	2	0.0462
GO:0070486	leukocyte aggregation	2	0.0462
GO:0071223	cellular response to lipoteichoic acid	2	0.0462
GO:0043066	negative regulation of apoptotic process	20	0.0468
GO:0002260	lymphocyte homeostasis	4	0.0472
GO:0032370	positive regulation of lipid transport	4	0.0472
GO:0050885	neuromuscular process controlling balance	4	0.0472
GO:0010591	regulation of lamellipodium assembly	3	0.0484
GO:0016032	viral process	6	0.0484
GO:0031644	regulation of neurological system process	6	0.0484
GO:2001240	negative regulation of extrinsic apoptotic signaling pathway in absence	3	0.0484
GO:0051056	regulation of small GTPase mediated signal transduction	8	0.0498
GO:0022411	cellular component disassembly	7	0.0499
GO:0051649	establishment of localization in cell	26	0.0499

Sup. Table 10

Primer	Sequence
αSMA	Sense: 5’-GTCCCAGACATCAGGGAGTAA-3´<br><br>Antisense: 5’-TCGGATACTTCAGCGTCAGGA-3´
Col1α1	Sense: 5’-GCTCCTCTTAGGGGCCAT-3´<br><br>Antisense: 5’-CCACGTCTCACCATTGGGG-3´
Pdgfrβ	Sense: 5’-TTCCAGGAGTGATACCAGCTT-3´<br><br>Antisense: 5’-AGGGGGCGTGATGACTAGG-3´
Timp1	Sense: 5’-TGCAACTCGGACCTGGTCATA-3´<br><br>Antisense: 5’-CGCTGGTATAAGGTGGTCTC-3´G
Adgre1 (F4/80)	Sense: 5’-TGACTCACCTTGTGGTCCTAA-3’<br><br>Antisense: 5’-CTTCCCAGAATCCAGTCTTTCC-3´
GFP	Sense: 5’-ACTACAACAGCCACAACGTCTATATCA-3´<br><br>Antisense: 5’-GGCGGATCTTGAAGTTCACC-3´
Gapdh	Sense: 5’-CAATGAATACGGCTACAGCAA-3´<br><br>Antisense: 5’-AGGGAGATGCTCAGTGTTGG-3´
Csf1r	Sense: 5’-: TGTCATCGAGCCTAGTGGC-3´<br><br>Antisense: 5’-CGGGAGATTCAGGGTCCAAG-3´
Cd68	Sense: 5’- TGTCTGATCTTGCTAGGACCG-3´<br><br>GAGAGTAACGGCCTTTTTGTGA-3´
Mpeg1	Sense: 5’- CTGGATGATAATAGCGTGTGCT-3´<br><br>Antisense: 5’-CAAGACAGGTAGTTTCAGGGC-3´

Sup. Table 10: Primers used for RT-qPCR
